# Abstracts of Papers Presented at the 2005 Pittsburgh Conference

**DOI:** 10.1155/JAMMC.2005.59

**Published:** 2005

**Authors:** Peter B. Stockwell

**Affiliations:** 1 P S Analytical Ltd Arthur House, Unit 3 Crayfields Industrial Estate Main Road, St Pauls Cray, Orpington, Kent BR5 3HP UK

## Abstract

To attend or not to attend, that is the question. The Pittsburgh Conference continues to pose this conundrum to conferees and exhibitors alike. This year's conference was the first to be presented without a set of paper abstracts—a good thing some would say but this old codger always used the paper abstracts to select papers of interest to our readership and to seek a full publication. The exhibit took its usual format but it seemed that there were less manufacturers present.  The information presented to the attendees was also lacking and many companies' details were missing from the final program book, an omission no doubt on their behalf—my company was one of these—however I feel sure that past Pittcon organizers would have been more persistent in getting the required details for the audience. As is now the norm, many of the presentations take the form of posters displayed within the exhibition area.  Without a driver to get the audience there, the traffic was slow, to say the least. Lecture presentations were also attended in a mixed fashion. So the Pittsburgh Conference show moves on, and again next year it will be held in Orlando from 12 March to 17 March 2006.  No doubt I will be there making it a straight 31 in a row; in Pittsburgh Conference terms I am just a beginner with many of the attendees making more shows in a run than that. Selected abstracts dealing with topics of interest to the readers of this journal follow—hopefully many of these groups will be willing to publish their work either within this journal or elsewhere.


Journal of Automated Methods & Management in Chemistry, **2005** (2005), no. 3, 59–209
© 2005 Hindawi Publishing Corporation

### Abstracts of Papers Presented at the 2005 Pittsburgh Conference

Peter B. Stockwell

*P S Analytical Ltd, Arthur House, Unit 3, Crayfields Industrial Estate, Main Road, St Pauls Cray, Orpington, Kent BR5 3HP, UK*

To attend or not to attend, that is the question. The Pittsburgh Conference continues to pose this conundrum to conferees and exhibitors alike. This year's conference was the first to be presented without a set of paper abstracts—a good thing some would say but this old codger always used the paper abstracts to select papers of interest to our readership and to seek a full publication. The exhibit took its usual format but it seemed that there were less manufacturers present. The information presented to the attendees was also lacking and many companies' details were missing from the final program book, an omission no doubt on their behalf—my company was one of these—however I feel sure that past Pittcon organizers would have been more persistent in getting the required details for the audience. As is now the norm, many of the presentations take the form of posters displayed within the exhibition area. Without a driver to get the audience there, the traffic was slow, to say the least. Lecture presentations were also attended in a mixed fashion. So the Pittsburgh Conference show moves on, and again next year it will be held in Orlando from 12 March to 17 March 2006. No doubt I will be there making it a straight 31 in a row; in Pittsburgh Conference terms I am just a beginner with many of the attendees making more shows in a run than that. Selected abstracts dealing with topics of interest to the readers of this journal follow—hopefully many of these groups will be willing to publish their work either within this journal or elsewhere.

### AUTOMATING LOWRY PROTEIN AND OTHER ASSAYS USING A PERSONAL AUTOMATED PIPETTING SYSTEM

***Michael N. Sevigny***

*Bio-Tek Instruments, Inc, Highland Park, PO Box 998, Winooski, VT 05404*

The most time-consuming aspect of conducting assays, such as the Lowry protein assay, is the pipetting required to create standard curves and sample dilutions. We will show how to utilize a "personal" precision TM XS automated pipetting system to mechanize the pipetting aspects, saving time and lab resources. The application of a small footprint multichannel pipetting system that has a level-sensing single-channel pipette can greatly increase throughput in the typical laboratory. Pipetting can be conducted to and from single tubes, bottles reagent troughs, 96- and 384-well microplates, or a variety of other labware. The device's flexible platform layout with 1- and 8-channel pipette heads accommodates applications from single-well-hit picking to multiwell serial dilutions. Using carbon-filled tips the single-channel pipette is capable of liquid level sensing, allowing for transfer of sample from unevenly filled sample tubes. Pipetting is highly accurate and precise. Dispense accuracy at 100 $\mu$L is within 2% with 2% CVs. We will describe the accuracy and precision of the single- and eight-channel dispensers. The accuracy and precision of samples and standards in a typical Lowry protein assay will also be shown. Customized software provides complete control of experimental design through an intuitive user interface. The use of a plate stacker, and more complete automation robotics, and interface software will also be outlined. The paper will lead the user through the programing of sample preparation for a Lowry assay, in order to show how straightforward it is on the system.

*Keywords:* sample handling/automation, sample preparation
*Application code:* bioanalytical
*Methodology code:* sampling and sample preparation

### OPTICAL BIOSNIFFER FOR METHYL MERCAPTAN VAPOR

***Kohji Mitsubayashi,* Takeshi Minamide, and Hirokazu Saito***

**Institute of Biomaterials and Bioengineering, Tokyo Medical and Dental University, 2-3-10 Kanda-Surugadai, Tokyo 101-0062, Japan*

Halitosis evaluation is important in the medical and dental fields, but there are no convenient devices with high gas selectivity for their diagnoses. Methyl mercaptan (MM) is one of typical causations for halitosis. But it has not been reported a gas sensor, that is, simple and convenient to use and could selectively detect the MM. On the other hand, a monoamine oxidase type A (MAO-A, one of xenobiotic-metabolizing enzymes) is reported to catalyze the oxidation of the organic compounds which has thiol and amino in human liver. In this study, MAO-A biosensors that could detect the MM in the liquid and gas phases were developed. A biochemical electrode was constructed using a Clark-type oxygen electrode with MAO-A immobilized membrane. The enzyme electrode was calibrated by using the MM solution from 0.004 to 4.0 mmol/L. As the next step, a biochemical gas sensor (biosniffer) for MM vapor was fabricated with the MAO-A electrode and a reaction unit with gas and liquid cells separated with porous diaphragm, and the characteristics were evaluated. The biosniffer could be used to measure the MM vapor from 0.15 to 3.7 ppm. Consequently it was suggested that the biosniffer would be applicable to quantify the halitosis.

*Keywords:* bioanalytical, biomedical, fiber optics, fluorescence
*Application code:* biomedical
*Methodology code:* sensors

### NEW ONLINE MONITORING SYSTEM FOR DISSOLVED GASES IN ELECTRICAL INSULATING FLUIDS

***John V. Hinshaw,[*] Dan Morgan, and Thomas Waters***

*[*]Serveron Corporation, 3305 NW Aloclek Drive, Hillsboro, OR 97124, USA*

Routine monitoring of fault gases in electrical insulating fluids helps identify and track problems in electrical generation and distribution systems before potentially catastrophic failures occur. This presentation discusses a new dedicated monitoring system that utilizes an online membrane extraction system and miniaturized gas chromatograph to quantify dissolved transformer gas concentrations every 1–4 hours. The monitoring system comprises three major components: a membrane gas extractor, a miniaturized gas chromatograph, and a microprocessor control subsystem, which are contained in a temperature-controlled enclosure that maintains specified performance levels across external temperatures from $-40^{\circ}$C to $+55^{\circ}$C. The gas chromatograph is a new modular application-specific design that uses a series of micropacked columns to separate fixed gases plus C1-C2 hydrocarbons in under 10 minutes, and optionally propane. The chromatograph is designed for unattended operation in remote outdoor installations with extended routine service intervals. The gas extractor circulates active insulating fluid across a semipermeable membrane that admits dissolved gases into a sampling space. The fluid returns to its source, and a gas pump circulates the extracted gases through a sample loop for GC analysis of equilibrium gas concentrations. Dissolved gas-in-oil concentrations are derived via their gas/oil partition coefficients, temperatures, and pressures. The microprocessor system controls the instrumentation, processes chromatograms, schedules analyses, as well as calibrations from an onboard gas source, and stores and delivers data on demand through multiple communication channels. It continuously monitors the levels and rates of change of up to nine gases and pushes communications to specific destinations in the event of alarm conditions. Ancillary monitoring and trending software takes a variety of configurations that depend upon end-user requirements.

This presentation gives an overview of the monitoring system with emphasis on subsystem integration, application scope, field ruggedness, and interoperability with external data destinations.

*Keywords:* fuels energy, petrochemical, gas chromatography, monitoring, process analytical chemistry
*Application code:* fuels, energy and petrochemical
*Methodology code:* gas chromatography

### FAST RESIN SCREENING AND METHOD DEVELOPMENT WITH PREPACKED PROCESS DEVELOPMENT COLUMNS

***Thomas J. Higley***

*Tosoh Bioscience LLC, 156 Keystone Drive, Montgomeryville, PA 18936, USA*

In the large-scale purification of proteins relevant for therapeutic or diagnostic use, liquid chromatography plays the most important role. In general, LC performance parameters such as selectivity, binding capacity, recovery, and so forth. are mainly influenced by the properties of the chromatographic medium. Therefore, selection of the most suitable medium is the significant key point to succeed in purification. This resin screening historically was accomplished by packing various bulk resins into small columns by hand, which required significant investments in time and cost.

In order to improve the efficiency of these resin-screening experiments, recently new cartridge-type prepacked scouting columns were introduced by Tosoh Bioscience (Montgomeryville, Pa). The 1 mL and 5 mL ToyoScreen columns are packed with various Toyopearl process resins and are a convenient and affordable alternative to self-packing. In this work, the utilizability of the ToyoScreen columns was evaluated on the purification of a monoclonal antibody, antithyroid stimulating hormone (anti-TSH) lgG from a cell culture supernatant.

*Keywords:* HPLC columns, liquid chromatography, method development, prep chromatography
*Application code:* process analytical chemistry
*Methodology code:* liquid chromatography

### MERCURY ANALYSIS OF ANY SAMPLE TYPE: NO COMPRESSED GASES REQUIRED

***Jason P. Gray,[*] Alvin Chua, and Koji Tanida***

*[*]AGS Scientific, Inc, 1511 Texas Avenue S, Suite 270, College Station, TX 77840, USA*

Mercury (Hg) is a common environmental pollutant that is being measured and monitored on a daily basis by industrial, governmental, and environmental contract laboratories around the world. With such an elevated and increasing level of monitoring, there are automated mercury analyzers in use at most such laboratories. The vast majority of these analyzers require high-purity compressed gases such as nitrogen, helium, and even argon for operation. Such gases are consumed by these instruments at a very high rate, typically costing these laboratories thousands of dollars per year in gas consumption, not to mention the associated labor costs. In today's laboratory economy, reduction of such unnecessary costs is a must. In this presentation, a suite of fully capable mercury analyzers that do not require any compressed gases for operation is described. These mercury analyzers function on the principles of reducing vaporization or thermal decomposition, optional gold amalgamation, and cold vapor atomic absorption spectroscopy (CVAAS). Illustrations and supporting data will be provided.

*Keywords:* atomic spectroscopy, environmental analysis, mercury, trace analysis
*Application code:* environmental
*Methodology code:* atomic spectroscopy/elemental analysis

### EVALUATION OF A NEW PURGE-AND-TRAP ON-LINE INTERFACE FOR THE REAL-TIME ANALYSIS OF VOCS IN AQUEOUS STREAMS

***Mark E. Krigbaum***

*Teledyne Instruments - Tekmar, 4736 Socialville Foster Road, Mason, OH 45040, USA*

The new on-line purge-and-trap Interface provides a solution for homeland defense drinking water protection and spill detection by sampling water intakes on public water supplies such as rivers, lakes, and reservoirs. In addition, water treatment facilities can monitor various stages of the water treatment process for the generation of disinfection by-products (DBPs) such as trihalomethanes automatically. The interface delivers water samples from up to six separate streams to a purge-and-trap concentrator for volatile organic compound (VOC) analysis. Standard solutions are automatically added to the 5 or 25 mL sample aliquot. The interface can also be configured in conjunction with a vial autosampler to run continuing calibration checks from vials intermixed with stream samples. The entire system is controlled using a special PC software allowing for unattended sampling and analysis at prearranged times. This new capability will allow water treatment facilities to configure alarm levels for continuous and unattended sampling and analysis.

*Keywords:* environmental/water, on-line, process monitoring, purge and trap
*Application code:* homeland security/forensics
*Methodology code:* sampling and sample preparation

### IDENTIFICATION OF CHEMICAL UNKNOWNS IN MICRO QUANTITIES

***Martin M. Szczesniak,* Michael Beecroft, and Jamie Ferguson***

**Surface Optics Corporation, 11555 Rancho Bernardo Road, San Diego, CA 92127, USA*

Identification of chemical unknowns in micro quantities remains a challenge and usually calls for infrared microscope, or GC/MS technology. This paper will present a method based on FTIR spectrometery with a VSphere accessory applied for detection of fractions of micrograms of chemicals. The VSphere sampling accessory will be described and its principle explained. The simplicity of the method will be compared with that of ATR. The quality of the VSphere-generated spectra will be compared with transmission, diffuse reflectance, and ATR methods. The sample used in analysis remains preserved and can be used again in other analytical methods. The effectiveness of library searches with VSphere-generated spectra will be presented. The paper will discuss the advantages of the VSphere method over the traditional methods.

*Keywords:* contamination, forensic, industrial hygiene, quality control
*Application code:* homeland security/forensics
*Methodology code:* vibrational spectroscopy

### ULTRASONIC AUTOSAMPLER FOR REDUCTION OF CARRYOVER IN HIGH-SENSITIVITY ANALYSIS

***Osamu Shirota,* Masashi Mita, and Xiaomi Xin***

**Shiseido Company, 2-2-1 Hayabuchi Tsuzuki, Yokohama 224-8558, Japan*

An attempt to install an ultrasonic cleaning device to an autosampler for HPLC is presented in this paper. Carryover, or a memory effect, from previous sample runs is becoming a serious problem in LC or LC-MS, especially in the field of pharmacokinetics and clinical analysis, where concentrations of unknown samples often range widely. While various practical strategies to clean an autosampler have been attempted in the real field, a problem common to them is time required to decrease a carryover peak. Ultrasonic cleaning is one of the most common cleaning techniques, used extensively, in laboratory experiments for industrial production. It will be discussed how quickly and efficiently the autosampler will be cleaned by the ultrasonic cleaning device through carryover-evaluating experiments with many compounds supposed to be "difficult."

*Keywords:* HPLC, liquid chromatography/mass spectroscopy, sample introduction
*Application code:* bioanalytical
*Methodology code:* liquid chromatography

### DEVELOPMENTS IN PURGE-AND-TRAP AUTOMATION

***Laura Chambers*** ***and Michael L. Duffy***

**OI Analytical, 151 Graham Road, College Station, TX 77842, USA*

Through the last twenty years, the development of purge-and-trap (P&T) instrumentation has seen continuous improvements including introduction of a sophisticated, third-generation sample concentrator two years ago at Pittcon 2003. This poster will introduce additional new developments in P&T instrument design that facilitate more complete automation, minimize downtime, and further shorten overall cycle time. The OI Analytical model 4660 eclipse purge-and-trap sample concentrator now includes features that more fully automate the entire VOC analysis procedure, minimizing manual labor and increasing laboratory revenue.

Instrumentation developments include complete automation of all pH measurements in water samples, a step that up until now had to be done manually. Automating this otherwise labor-intensive pH measurement reduces the need to collect replicate sample aliquots, eliminates the need to check the pH of each sample individually and manually record the results in a log, and improves reliability and quality of the data. The reduction in labor results in a direct increase in laboratory revenue. A full description of the system, including its LAN/LIMS reporting capability, will be included.

Other improvements in automation include eliminating idle downtime while the autosampler waits for the P&T to cool, shortening cycle times by as much as one to two minutes, a fully integrated service and maintenance log, improved leak checking, and full LAN capability for off-site monitoring and integration into LIMS systems. A complete description of the newly automated features and benefits to the user will be described.

*Keywords:* environmental analysis, GC, purge and trap, volatile organic compounds
*Application code:* environmental
*Methodology code:* gas chromatography/mass spectrometry

### AUTOMATED CHARACTERIZATION, PURIFICATION, AND FRACTION ANALYSIS

***Paul M. Lefebvre,**** ***Ronan Cleary, and Warren Potts***

**Waters Corporation, 34 Maple Street Mailstop Gc, Milford, MA 01757, USA*

A standard requirement for drug discovery screening of synthetic libraries is for the test compounds to have a minimum purity. The typical requirement is greater than 85% purity based on the area % of an LC chromatogram with a generic detector like UV, ELSD, MS TIC, or a combination of multiple detectors. If the screening compounds do not meet this requirement, purification and reanalysis are required. Managing the flow samples, subsequent fractions, and all the associated data through this process can often be difficult and time consuming. This poster will illustrate how a compound set is efficiently taken through the purification process with Waters AutoPurify Application Manager. This software allows for automation from the initial QC, through the purification, to fraction analysis. Using the target's data from the analytical results, the appropriate purification method, if necessary, is selected based on the user-defined purification strategy. Additionally, the selected purification method can be a narrow gradient, identified based on the analytical retention time. This provides optimal target separation of closely eluting impurities, thus improving the resulting fraction purity.

Another useful tool to streamline the process is automatic fraction analysis. This allows fractions to be injected immediately after purification directly from the fraction bed. This feature improves the overall efficiency of the process by eliminating the user's intervention and improving the system's throughput, while giving high-quality data. This poster will also show the results of the automatic fraction analysis are equivalent to those obtained after drying the fractions, reconstituting, and then analyzing.

*Keywords:* high-throughput chemical analysis, isolation/purification, liquid chromatography/mass spectroscopy, prep chromatography
*Application code:* high-throughput chemical analysis
*Methodology code:* liquid chromatography/mass spectrometry

### THE USE OF PARALLEL HPLC TO SPEED UP CHIRAL COLUMN SCREENING

***Holger Gumm*** ***and Ariane Bredebusch***

**Sepiatec GmbH, Louis-Bleriot-Street 5, Berlin 12487, Germany*

The process of finding the optimal separation technique for a given chiral sample is mostly done by screening a given set of methods on several different columns. For about 90% of the samples a satisfactory method can be found this way. The methods for the rest of the samples undergo manual optimization by a skilled scientist. To reduce the workload for the operator and give him more time to tackle the challenging problems the screening should be faster and more efficient.

A way to speed up the chiral screening process is the parallelization of the process. With the Sepmatix system, eight columns can be used simultaneously with only one pump. Therefore the flow of the pump has to be split to eight HPLC lines. If a passive splitter is used, the flow in each line is a function of the back pressure of the column in this line. Even if this might be considered acceptable for applications where columns of a single batch and therefore with a comparable back pressure are used, it is not acceptable if different columns should be used for measurements to compare the separation. Out of this reason, the Sepmatix system contains an active splitter for the flow of the pump, which ensures the same flow rate in each channel even if the back pressure in the columns differs as much as up to 100 bars. A special setup for the chiral column screening is available for the Sepmatix system. The parallel Sepmatix autosampler is modified in a way that the same sample can be injected into eight loops simultaneously and then be separated on the different columns. Therefore one method is checked with up to eight different columns simultaneously. If the optional column oven is used, the influence of the temperature on the separation can be screened as well. Since each single column compartment has its own temperature, temperature screening can be parallelized as well.

*Keywords:* automation, chiral separations, chromatography, drug discovery
*Application code:* pharmaceutical
*Methodology code:* liquid chromatography

### IN-PROCESS PARTICLE MEASUREMENT UNDER IN-SITU CONDITIONS IN ORIGINAL DISPERSED PHASES UP TO CV 70 VOL%

***Larry R. Unger***

*Particle Sizing Systems, 2490 Sandstone Court, Wellington, FL 33414-7785, USA*

Techniques exist for measuring particle size in dispersed systems, but they require sample preparation. A common wish is to work under real process conditions, where the need is for a system that will not change the characteristics of the product, such as those that occur during dilution. This would be ideally suited to process control in chemical/biochemical processes, where results can be obtained rapidly allowing operators to make adjustments for efficient production. Efforts to develop such a system involved studies with a "self-selecting scanning laser system with an automatic and dynamic focus system." This unique method showed restrictions of size range and resolution. Further research led to a scanning laser system, named 3D ORM (3-dimensional optical reflectance measurement) to differentiate it from existing techniques. Patents have been issued in both the US (1999) and UK (2000) for MTS 3D ORM technology.

In operation, light from a laser diode is transmitted to the sensor by a single optical fiber that also receives size information for the particles in the self-selecting and moving focus (Figure 1). The sensor was designed so that the focal point traces an elliptical path at an angle in front of the window. This movement of this point in 3-dimensional space is the origin of the term "3D ORM." Data from concentrated dispersions, emulsions and pigments from the 3D ORM system will be compared with data from instrumentation operating in 2D mode or digital microscopy, highlighting advantages of this new technique. Measurements can be made up to 4000 microns and are presented in each of 1024 channels. 3D ORM MTS particle analysis systems have been proven in solid-in-gas, solid-in-liquid, and liquid-in-liquid applications; pharmaceutical application per CFR21 part 11, hazardous environment with ATEX Ex proof versions, and under wide range of media, temperatures, and pressures through custom sensor construction with a variety of specialized components.

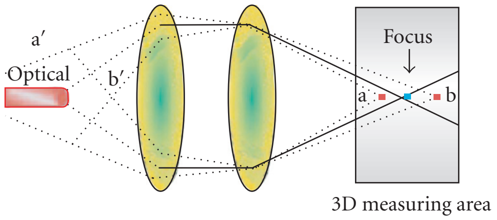

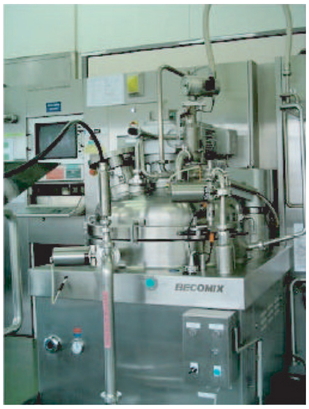

Figure 1: MTS 3D ORD dynamic focus system.

*Keywords:* instrumentation, particle size and distribution, process control, quality control
*Application code:* general interest
*Methodology code:* others

### INTELLIGENT AUTOMATION IN RAMAN MICROSCOPY

***Fran Adar,* Emmanuel Froigneux, Adrian Knowles, Hans Juergen Reich, Bernard Rousseau, and Andrew Whitley***

**Jobin Yvon Horiba, 3880 Park Avenue, Edison, NJ 08820, USA*

Raman microscopy has progressed and evolved over the last decade from a research instrument into a full analytical tool. The development of specialized CCD detectors, of new-generation laser rejection filters, and of small and compact solid-state lasers have meant that the new generation of Raman instruments can really fulfill the role of both a research and routine analytical instrument. The advances in the performance, stability, and robustness of the Raman instrument have also required a new level of automation to be considered so that the flexibility and advantages of the technique can be explored without compromising the all-important convenience and ease of use of the equipment. The faster one can acquire a spectrum, one can operate with various laser wavelengths, one can optimize or exploit different spectroscopic methods ultimately the more time and money will be saved in analysis. In this talk we outline the requirements of the modern Raman microscope and explore the various aspects of the new analytical Raman system design. We discuss the developments made in the components such as lasers and detectors and the work undertaken by the design team at Jobin Yvon (NJ) to develop a Raman microscope to match intelligent automation with high performance and flexibility. We introduce the concepts and implementation of the QUAD grating system, the LRF-4 notch filter system, and the CREST scanning acquisition that has enabled the next generation of analytical Raman microscope.We look into how this can be taken further with specialized sampling arrangments for dedicated analyzers and screening systems.

*Keywords:* instrumentation, materials characterization, microspectroscopy, Raman
*Application code:* others
*Methodology code:* vibrational spectroscopy

### AN ELECTRONIC NOSE FROM ARRAYS OF NANOPARTICLE-BASED CHEMIRESISTIVE VAPOR SENSORS

***Nathan S. Lewis***

*Caltech, 1200 E California Boulevard M/C 127-72, 210 Noyes, Pasadena, CA 91125-7200, USA*

A method is described for generating a variety of chemically diverse, broadly responsive, low-power vapor sensors. A key to our ability to fabricate chemically diverse sensing elements is the preparation of processable, air stable films of electrically conductive nanoparticle composites. An array of such chemiresistive elements produces a chemically reversible, diagnostic pattern of electrical resistance changes upon exposure to different odorants. Such conducting polymer elements are simply prepared and are readily modified chemically to respond to a broad range of analytes. In addition, these sensors yield a fairly rapid, low-power, DC electrical signal in response to the vapor of interest, and their signals are readily integrated with software- or hardware-based neural networks for purposes of analyte identification.

Principal components analysis has demonstrated that such sensors can identify and quantify different airborne organic solvents and can yield information on the components of gas mixtures.

*Keywords:* detection, sensors
*Application code:* homeland security/forensics
*Methodology code:* sensors

### INFORMATICS FOR LARGE-SCALE PROTEOMICS

***John Yates,* Daniel Cociorva, Rovshan Sadygov, and David Tabb***

**Department of Cell Biology, The Scripps Research Institute, 10550 North Torrey Pines Road, LaJolla, CA 92037, USA*

New methodologies to identify proteins such as tandem mass spectrometry have increased the potential scale of proteomics experiments. These new strategies are heavily dependent on informatics to process data and identify proteins. High-resolution separation methods together with faster scanning tandem mass spectrometers have increased the volume of data to be processed. We describe informatic approaches to handle large volumes of data and methods to extract more information from the datasets.

*Keywords:* bioinformatics, liquid chromatography/mass spectroscopy, mass spectrometry, proteomics
*Application code:* proteomics and genomics
*Methodology code:* mass spectrometry

### PORTABLE MICROPLASMA DEVICES AND MICROFLAME DEVICES FOR OPTICAL AND MASS SPECTROMETRIC DETECTION

***Vassili Karanassios***

*Department of Chemistry, University of Waterloo, Waterloo, Ontario, Canada N2L 3G1*

In classical elemental analysis, samples are collected in the field and are brought to the lab for analysis. But there are many cases in which analytical results must be obtained on site (ie, in the field) to enable rapid decision-making. In other cases, samples may be screened in the field and only the "suspect ones" may be shipped to a laboratory for thorough analysis. Toward this ideal we are developing and characterizing portable, battery-operated microplasma devices (MPDs) (Karanassios V. "Microplasmas for chemical analysis: analytical tools or research toys?" *Spectrochimica Acta,* Part B, vol. 59, no.7, pp. 909–928, 2004) that can be used on site. And, to eliminate the need for use of inert gases (such as Ar or He), we are also developing and characterizing microflame devices (MFDs) that may be used in the field. Sample introduction is a key issue when using MPDs or MFDs. For this, we are using a small-size (eg, mini) in-torch vaporization (ITV) sample introduction system. The mini-ITV system was originally developed for use with ICPs (Smith AT, Badiei HR, Evans JC, Karanassios V. "Simultaneous determination of the Cd and Zn total body burden of individual, nearly microscopic, nanoliter-volume aquatic organisms (*Hyalella azteca*) by rhenium-cup in-torch vaporization (ITV) sample introduction and axially viewed ICP-AES." *Anal Bioanal* Chem. vol. 380, no. 2, pp. 212–217, 2004) and its "mini" version proved to be the Achilles' tendon (rather than the Achilles' heel) for both the MFD and the MPD.

In this presentation, development, characterization, and analytical performance characteristics of MPDs and MFDs with optical emission and mass spectrometry will be described in detail.

*Keywords:* atomic spectroscopy, instrumentation, small samples
*Application code:* environmental
*Methodology code:* others

### A NEW PREPARATIVE COLUMN THAT BRIDGES THE GAP FROM ANALYTICAL TO PREPARATIVE HPLC

***Fang Xia,* Jie Y. Cavanaugh, Diane M. Diehl, Uwe D. Neue, John O'Gara, and Yuehong Xu***

**Waters Corporation, 34 Maple Street Mail Stop Cat, Milford, MA 01757, USA*

Maximizing column loading is a final goal of preparative chromatography. However, in practice, tailing peaks limit the resolution and hence mass loading of a column. High silanol activity contributes significantly to peak tailing in RPLC columns. A physical understanding of the nature of surface silanol is first discussed in this presentation. An innovative synthesis process enables higher surface coverage and end capping to dramatically reduce silanol activity. Therefore, this silica-based column provides high efficiencies, more symmetric peak shapes, and extremely high mass loading for acids, neutrals, and bases. The lifetime testing was performed under aggressive conditions. The results suggest that this column exhibits superior stability under low pH conditions. Several applications including pharmaceutical molecules, peptides, and natural products are also discussed in this presentation to demonstrate the wide application of this material to various purification areas. Our data indicate that this new silica-based reversed-phase LC column has excellent peak shapes, good stability, and very high mass loading. This material bridges reproducible purification of a variety of products from milligrams to grams.

*Keywords:* modified silica, prep chromatography, surface analysis
*Application code:* pharmaceutical
*Methodology code:* separation sciences

### ULTRAFAST, HIGH-RESOLUTION STABILITY-INDICATING METHOD DEVELOPMENT USING 1.7 MICROMETER PARTICULATE COLUMNS

***Eric S. Grumbach,* Diane M. Diehl, and Jeffrey R. Mazzeo***

**Waters Corporation, 34 Maple Street, Milford, MA 01757, USA*

Many LC/UV/MS assays run today use 2 mm id columns packed with 5 $\mu$m particles. In theory, utilizing 1.7$\mu$m particles and 1 mm id columns should lead to an 8-fold improvement in sensitivity leading to lower limits of detection. These improvements are dependant on having an LC system that operates at higher pressures and has minimal system volume. The resolution and sensitivity of 5 $\mu$m (2.1 mm id) and 1.7 $\mu$m (1.0 mm id) particles on a hybrid inorganic-organic stationary phase were compared to establish the benefit of using sub-2 $\mu$m particles for improving limits of detection for stability-indicating assays. An active pharmaceutical ingredient was degraded using acid, base, and peroxide then analyzed by LC/UV/MS. The degraded drug product was analyzed at both low (pH 2.5) and high (pH 10.0) pH to optimize the selectivity of the separation so that all degradants were resolved from the parent compound. Ultrafast, high-resolution stability-indicating methods can be achieved by utilizing narrow 1.0 mm id columns packed with 1.7 $\mu$m particles.

*Keywords:* method development, pharmaceutical
*Application code:* pharmaceutical
*Methodology code:* liquid chromatography

### A DUAL-SOURCE INDUCTIVELY COUPLED PLASMA/ ELECTROSPRAY IONIZATION TIME-OF-FLIGHT MASS SPECTROMETER FOR RAPID SPECIATION AND METABOLIC ANALYSIS

***Duane A. Rogers,* Gary M. Hieftje, and Steven J. Ray***

**Chemistry Department, Indiana University, Bloomington, IN 47405, USA*

Atomic spectrometry is having an ever-greater impact upon bioscience. The combination of these disciplines is critical to elucidate the role of metals in metabolic pathways, toxicity, and bioavailability. Ordinarily, the metal "speciation," which includes its chemical environment, valence state, and noncovalent binding, is determined by utilizing one or more separation procedures followed by an element-specific detector. As a result, identification of the metal species depends on the reproducibility of the separation techniques being employed, the availability of suitable standards, or employing a second, separate, molecular detector. In order to improve speciation analysis, we have developed a novel dual-source time-of-flight mass spectrometer (TOFMS). The instrument utilizes a single TOFMS that samples two separate ionization sources simultaneously. In the current arrangement, atomic information is derived after a chromatographic separation by means of an ICP source. Simultaneously, molecular species are identified by an electrospray ionization source. Both kinds of information can then be exploited to identify the metal species within a chromatographic peak, even when suitable standards are not available.

Because a single chromatographic system and a single TOFMS are used for both ionization sources, many redundant and costly components are eliminated, while time and sample requirements are reduced. Figures of merit for the dual-source TOFMS will be presented along with a preliminary characterization of the instrument.

*Keywords:* atomic spectroscopy, ICP-MS, speciation, time-of-flight MS
*Application code:* others
*Methodology code:* atomic spectroscopy/elemental analysis

### HIGH-THROUGHPUT OXYGEN SENSOR FOR MONITORING BACTERIAL PATHOGENS

***Jason E. Karasinski,[*] Silvana Andreescu, and Omowunmi A. Sadik***

[*]*Department of Chemistry State University of New York at Binghamton, PO Box 6000, Binghamton, NY 13902, USA*

Conventional culture methods allow for the detection of bacteria with adequate sensitivity and selectivity required in many practical applications. However, in spite of their advantages these methods are extremely slow, requiring high-skill personnel and a single assay could require up to seven days. In this context, novel biosensing technologies are being developed to address specific issues for faster and simpler detection of bacterial pathogens. Current trends in this direction are oriented towards the development of novel sensors, autonomous cell-based toxicity monitoring or microsensors. This presentation will focus on the development and optimization of a high-throughput electrochemical oxygen sensor for (i) monitoring and differentiation of bacteria populations and for (ii) studying the interaction of bacteria with various analytes (drugs, chemical toxins). The system provides continuous information on metabolic activity of bacteria for more than 12 hours without any additional incubation or preparation steps. A comparison with classical culture methodology used for monitoring bacteria and their interactions with toxins will be also presented.

*Keywords:* bioanalytical, biosensors, high-throughput chemical analysis
*Application code:* bioanalytical
*Methodology code:* sensors

### REAL-TIME DETECTION OF NITRIC OXIDE (NO) RELEASED FROM CELLS USING MICROPHYSIOMETER TYPE ARRANGEMENT WITH CELLS ADHERED TO SURFACE OF ELECTROCHEMICAL NO SENSOR

***Wansik Cha,[*] Anjali Desai, Mark E. Meyerhoff, and Jeffrey S.Warren***

[*]*University of Michigan, Chemistry Building, Room 3316, 930 N University Avenue, Ann Arbor, MI 48109, USA*

To date, mechanistic studies related to nitric oxide (NO) production from living cells have been carried out by estimating the amount of NO released via measurements of total $NO_2$-/$NO_3$-levels and/or L-citrulline in the bathing media, or by assessing the levels of nitric oxide synthase (NOS) or NOS-mRNA expression, within the cells (Ganster et al., in *Nitric Oxide: Biology and Pathobiology,* In Ignarro LJ., Ed., vol. 2000, pp. 129–156, Academic Press, San Diego.). Herein, we describe a method in which NO produced by cells is directly measured at or near the surface of an improved amperometric NO sensor (Lee et al., *Anal. Chem.,* vol. 76, no. 3, pp. 536–544, 2004). Inspired by the previous research on the direct and real-time detection of NO generation from animal tissue slices (Lee et al., *Anal. Chem.,* vol. 76, no. 3, pp. 545–551, 2004), an integrated NO sensor platform with immobilized living cell layers and a flow-through microphysiometer set-up (McConnell et al., *Science*, vol. 257, no. 5078, pp. 1906–1912, 1992), has been developed. This arrangement can be used to investigate physiological responses of cells by measuring the extracellular NO production in real time, and in the presence of various activators or inhibitors of cellular NOS activity flowing within the media perfusing the cells. For example, erythropoietin (EPO) is one of primary targets in this study due to its effects on NOS expression and NO production by endothelial cells (ECs) (Wu et al., "Role of erythopoietin and nitric oxide in modulating tone of interlobular and uraemic subcutaneous arteries in man," *Clinical Science,* vol. 97, pp. 639–647, 1999, and Wang et al. "Hypertension," vol. 33, no. 3, pp. 894–899, 1999). The influence of EPO in the media on ECs is determined by observing both the basal levels of NO production from cell layers as well as the NO build-up rate while the flow of media is stopped over the cultured EC layer. Similar experiments can be carried out with other types of cells, including smooth muscle cells, where iNOS activation by cytokinin can be followed via amperometric NO detection in real time. Other enhancers or suppressors of NOS expression, receptor-binding hormones and known NOS inhibitors can also be tested in this system to obtain their dose-response patterns, and to help understand the signal transduction pathways or the regulation of NOS enzyme expression which is accompanied by NOS activation or inhibition.

*Keywords:* biosensors
*Application code:* bioanalytical
*Methodology code:* sensors

### ON-LINE COUPLING OF SOLID-PHASE MICROEXTRACTION AND CAPILLARY ISOELECTRIC FOCUSING WITH LASER-INDUCED FLUORESCENCE WHOLE-COLUMN IMAGING DETECTION FOR PROTEIN ANALYSIS

***Zhen Liu[*] and Janusz B. Pawliszyn***

[*]*University of Waterloo, 200 University Avenue West, Waterloo, Ontario, Canada N2L 3G1*

An on-line coupling method of solid-phase microextraction (SPME) and capillary isoelectric focusing (CIEF) with laser-induced fluorescence (LIF) whole-column imaging detection (WCID) was developed for the analysis of proteins. SPME

is a novel sampling and sample preparation technology, while CIEF is a high-resolution electrophoretic separation method for the analysis of amphoteric molecules particularly proteins. Unlike in other liquid-phase separation methods and conventional CIEF, proteins are focused into stationary bands within a pH gradient in CIEF-WCID. CIEF-WCID is therefore the most compatible liquid-phase separation method for coupling with SPME, which can effectively resolve the problems associated with the slow desorption kinetics of SPME in a liquid phase. By combining SPME and CIEF-WCID, the desorption time can be as long as necessary, allowing for complete desorption without any band broadening and analyte carryover. By using this method, R-phycoerythrin in water can be extracted by SPME in 10 minutes, and subsequently analyzed by CIEF-LIF-WCID within 20 minutes, providing a limit of detection (LOD) of 3.5 pM (S/N = 3). The feasibility of the SPME-CIEF-LIF-WCID method was demonstrated by extracting and analyzing extracellular phycoerythrins in cultured cyanobacteria samples. Extracellular phycoerythrins at the nM level were extracted and analyzed in 30 minutes, while avoiding the interference of the cyanobacteria cells.

*Keywords:* bioanalytical, capillary electrophoresis, protein, SPME
*Application code:* bioanalytical
*Methodology code:* capillary electrophoresis

### DETERMINATION OF LOW-MOLECULAR-MASS ALDEHYDE BY AUTOMATED HEADSPACE SOLID-PHASE MICROEXTRACTION WITH IN-FIBER DERIVATIZATION

***Qing Wang***

*Department of Chemistry, University of Waterloo, Waterloo, Ontario, Canada N2L3G1*

Headspace solid-phase microextraction (HS-SPME) analysis of low-molecular-weight (C1-C10) aldehydes in aqueous solutions was investigated using pentafluorophenylhydrazine (PFPH) and O-2, 3, 4, 5, 6-(pentafluorobenzyl)-hydroxylamine hydrochloride (PFBHA) as in-fiber derivatization reagents. Analysis of the derivatives was achieved using GC-FID. A comparison of the two reagents showed that PFBHA was superior to PFPH using this technique under the investigated conditions. Fundamental studies of the PFBHA and PFPH reactions showed that the kinetics of the process was limited by diffusion of the analytes to the fiber. The developed PFBHA method gave detection limits in the low to submicrogram per liter range for most of the aldehydes tested. The method was applied successfully to the analysis of particle board, wine, and fish samples.

*Keywords:* automation, derivatization, SPME
*Application code:* food science
*Methodology code:* sampling and sample preparation

### CHARACTERIZATION OF POLYMERS AND PLASTICS BY PYROLYSIS-GC/MS

***Inger Ericsson***

*Pyrol AB, PO Box 766, Lund 220 07, Sweden*

pyrolysis-gas chromatography-mass spectrometry (Py-GC/MS) has been used for a long time to characterize nonvolatile samples. The technique has improved by more sophisticated pyrolysis instruments, more efficient separation columns, and cheaper mass spectrometers. The possibility to analyze polymers and plastics can be useful in many different circumstances, for example, product control, new products, reclamations, and in the forensic field. The reasons are that the technique can handle very small sample sizes, identify volatile and relatively volatile samples first by thermal desorption without extraction before pyrolysis. The sample handling is thus very simple. It can also find small differences and identify the polymers by specific pyrolysis products or reference materials.

To be able to characterize complex samples in detail, different pyrolysis methods are used like sequential and fractionated pyrolysis as well as pyrotomy. Sequential pyrolysis is used to find the thermal degradation rate and behavior, fractionated pyrolysis to more easily find the different substances and pyrotomy to study the surface and different layers in a sample.

*Keywords:* materials characterization, polymers and plastics, rubber, surface analysis
*Application code:* polymers and plastics
*Methodology code:* gas chromatography/mass spectrometry

### INTEGRATION OF AN IMMOBILIZED PC 12 CELL REACTOR WITH A MICROCHIP-BASED FLOW ANALYSIS SYSTEM

***Michelle W. Li*** **[*]** ***and R. Scott Martin***

[*]*Department of Chemistry, Saint Louis University, 3501 Laclede Avenue, Saint Louis, MO 63103, USA*

Recent studies have shown that nitric oxide may play a role in degeneration of dopaminergic neurons. New analytical tools are needed to gain insight into the mechanisms of this degeneration. In this paper, we will describe the development of such tools, specifically a new method for detecting dopamine release from rat pheochromocytoma (PC12) cells using microchip-based flow analysis and amperometric detection. The cells are immobilized in poly (dimethylsiloxane)-based microchannels using a new cell culturing technique. Various coatings for immobilizing the cells were studied. A new method for introducing cells into the PDMS channels was also developed. This technique involves selectively coating the microchannels with collagen, followed by pipetting the cells over the PDMS structure with the cells only being immobilized on the coated channels. The cell-coated microchannels can then be reversibly sealed to a glass plate that contains electrodes for amperometric detection. The nafion-coated electrodes were made by micromolding carbon inks and used to measure dopamine release from the cells upon stimulation with a calcium solution. Varying concentrations of PC 12 cells placed in the channels produced dopamine release ranging from 31 to 160 micromolars. Channels with microtextured posts were also utilized to create a more even distribution of cells in the channel network. Studies using the described methodology to investigate the effect of nitric oxide on the release of dopamine from the cells will also be presented.

*Keywords:* bioanalytical, biological samples, lab on a chip/microfluidics, microelectrode
*Application code:* bioanalytical
*Methodology code:* microfluidics/lab on a chip

### A MICROFLUIDIC CHIP COUPLED TO MICRODIALYSIS FOR IN VIVO MONITORING OF PRIMARY AMINE NEUROTRANSMITTERS BY CAPILLARY ELECTROPHORESIS

***Zechariah D. Sandlin,[*] Robert T. Kennedy, and Jonathan G. Shackman***

[*]*Department of Chemistry University of Michigan, 930 North University Avenue, Ann Arbor, MI 48109, USA*

The ability to monitor neurotransmitters in vivo in the extracellular space of the brain can aid in the elucidation of their pathological role in neurological diseases. Previously we have developed an online microdialysis-based capillary electrophoresis (CE) system that utilizes laser-induced fluorescence (LIF) with a sheath-flow cuvette detector capable of measuring in vivo basal and stimulated levels of primary amine neurotransmitters with high temporal and spatial resolution. However, dissemination of the CE-LIF methodology is impeded by the operational complexity of the system. In response, we have developed a $1'' \times 3''$ disposable microfluidic chip to replace the mixing tee, reaction chamber, flow-gate interface, and separation capillary of the CELIF system. Utilizing LIF with a confocal epifluorescence detector, the microfluidic chip is capable of sampling from a microdialysis probe, online derivatization, injection, and separation of primary amine neurotransmitters. In the future, the microfluidic chip could be readily expandable to an array format to allow for multiple analyses from a single run.

*Keywords:* bioanalytical, capillary electrophoresis, lab on a chip/microfluidics
*Application code:* bioanalytical
*Methodology code:* microfluidics/lab on a chip

### MICROCHIP-BASED RESISTANCE VESSEL MIMICS FOR THE DETECTION OF DEFORMATION-INDUCED ATP FROM ERYTHROCYTES

***Alexander K. Price,[*] R. Scott Martin, and Dana M. Spence***

[*]*Department of Chemistry, Saint Louis University, 3501 Laclede Avenue, Saint Louis, MO 63103, USA*

The fabrication of microfluidic devices for use as mimics of resistance vessels in the microcirculation will be described. Soft-lithographic methods facilitate the fabrication of poly(dimethylsiloxane)-based microchannels with dimensions similar to those of arterioles in vivo. When red blood cells (RBCs) are mechanically pumped through these microstructures, micromolar amounts of adenosine triphosphate (ATP) have been measured via luciferin/luciferase-mediated chemiluminescence. ATP is known to stimulate nitric oxide (NO) production in endothelial cells that line the lumen of vessels in vivo. Nitric oxide is well known for its ability to activate guanylyl cyclase, resulting in relaxation of smooth muscle cells and eventual vasodilation. With these microchip devices, we have been able to show that this deformation-induced release of ATP changes accordingly from lower to higher concentrations when the cross-sectional area of the channel is decreased. Microscopic images of RBC flow through a channel indicate that this model constitutes an adequate mimic due to the presence of a “cell-free” layer at the wall of the channel. ATP release from RBCs flowing through microchannels and microbore tubing that have similar cross-sectional areas is statistically equivalent, giving further evidence that microchip-based vessel mimics can operate just as effectively as previously identified mimics. A more complex chip-based mimic having channels that scale down uniformly (similar to vessels in vivo) has also been implemented, enabling the investigation of numerous channel cross-sections on the same device. Results suggest the combined decrease in channel cross-section and increase in linear flow velocity result in increased ATP release. In addition, we have designed a device for investigating ATP release from RBCs where the RBC stream is focused and deformed by two parallel luciferin/luciferase streams. In this design, the width of the RBC stream, and thus the amount of ATP released, is controlled by only changing the flow rates of the introduction channels so that one uniform channel design can be used to deform RBCs in dimensions ranging between those found in arterioles and capillaries. Preliminary data from this study will also be presented.

*Keywords:* bioanalytical, biological samples, chemiluminescence, lab on a chip/microfluidics
*Application code:* bioanalytical
*Methodology code:* microfluidics/lab on a chip

### DESIGN OF INTERFACIAL SELECTIVITY OF DNA PROBES FOR DEVELOPMENT OF QUANTITATIVE ELECTROKINETIC BIOCHIPS

***Ulrich J. Krull,[*] David Erckison, Dongqing Li, Ying Lim, Xuezhu Liu, Paul Piunno, and April Wong***

*[*]The University of Toronto, Mississauga, Ontario, Canada L5L 1C6*

A new strategy will be reported for covalent immobilization of single-stranded DNA (ssDNA) probes that provides for improved selectivity and for control of melt temperature. The goal is to achieve substantially increased selectivity through control of the environment of the ssDNA probes that are immobilized onto a glass or fused silica surface. One approach is to use a “matrix isolation” method to achieve the desired environment for the probe molecules. The DNA oligonucleotide probes are polyelectrolytes with charged backbones and significant flexibility. The probe molecules are isolated by surrounding each on average with a sheath of immobilized polyelectrolyte, that is, not based on complementary nucleic acid. A more reproducible environment would reduce degrees of freedom and therefore would provide for sharpening of melt curves. Control of the density and organization of the surface chemistry can be used to adjust the duplex melting temperature so that combinations or arrays of immobilized nucleic acid films in a system can be made to have similar melt temperature, largely overcoming limitations that are often associated with oligonucleotide length and sequence. An electrokinetically controlled poly (dimethylsiloxane) microfluidic chip has been used to examine strategies for development of quantitative DNA hybridization methods. Steep shear gradients near the wall make electroosmotic flow (EOF) a particularly good technique for removal of nonspecific adsorption. The EOF-driven delivery provides for improved kinetics based on control of convective flow. Quantitative sample delivery with an on-line detection process that can be conducted simultaneously can reduce the total analysis time to a few minutes. Several other important features of the device include the use of Joule heating for collection of melt curves and control of selectivity, and for rapid on-line regeneration of the hybridization chemistry.

### ACKNOWLEDGEMENT

We are grateful to the Natural Sciences and Engineering Research Council of Canada for support of this research work.

*Keywords:* biosensors, fluorescence, immobilization, lab on a chip/microfluidics
*Application code:* bioanalytical
*Methodology code:* microfluidics/lab on a chip

### DETERMINATION OF LEAD BASED ON CATALYTIC DNA IN A CAPILLARY ELECTROPHORESIS MICROCHIP

***Donald M. Cropek,[*] Paul W. Bohn, In-Hyoung Chang, Yi Lu, and Jonathan V. Sweedler***

*[*]Construction Engineering Research Laboratory, US Army Engineer Research and Development Center, Champaign, IL 61822, USA*

A microchip-based lead sensor was developed that employs lead-specific catalytic DNA as the recognition element. Lead-specific catalytic DNA (DNAzyme) cleaves its complementary substrate DNA strand in the presence of only cationic lead ($Pb^{2+}$). Fluorescent tags on the substrate DNA transduce the $Pb^{2+}$ concentration to a measurable, optical signal. The DNAzyme responds sensitively and selectively to $Pb^{2+}$ but lacks activity toward other divalent cations. The on-chip preparative separation of $Pb^{2+}$ is achieved by a nano/microfluidic system. It has a hybrid three-dimensional fluidic structure that incorporates a nuclear track-etched nanocapillary membrane between two crossed, spatially separated, poly (dimethylsiloxane)(PDMS) microfluidic channels. This allows for reliable sample injection, electrophoretic separation, and isolated delivery of $Pb^{2+}$ to the DNAzyme in a spatially confined detection window where the fluorescent substrate fragments are interrogated using laser-induced fluorescence (LIF).

In addition to $Pb^{2+}$, the migration times of individual heavy metal ions $Mn^{2+}$, $Co^{2+}$, $Cu^{2+}$, $Cd^{2+}$, $Zn^{2+}$, and $Ni^{2+}$ were investigated using on-chip contactless conductivity detection in a nano/microfluidic device. The ability to separate and deliver desired analytes to the DNAzymes will be shown. This method provides a new means for rapid and reliable determination of $Pb^{2+}$ in a microchip format. Successful applications to real environmental samples are demonstrated. This general approach can be extended to further applications including creation of field sensors for additional compounds of environmental concern including other heavy metals, explosives, and depleted uranium.

### ACKNOWLEDGEMENTS

This work was funded by the Department of Defense SERDP program and the Long-Term Monitoring Program.

*Keywords:* biosensors, fluorescence, lab on a chip/microfluidics, metals
*Application code:* environmental
*Methodology code:* microfluidics/lab on a chip

### METAL-POLYMER NANOCOMPOSITES FOR INTEGRATED MICROFLUIDIC SEPARATIONS AND SURFACE-ENHANCED RAMAN SPECTROSCOPIC DETECTION

***R. Maggie Connatser*** ***[*]*** ***and Michael J. Sepaniak***

[*]*Department of Chemistry, University of Tennessee at Knoxville, 552 Buehler Hall, Knoxville, TN 37996-1600, USA*

The widespread development of microfluidics (microfluidics) has allowed for the extension of efficient separations, fluid handling, and hyphenation with many detection modes to a small, portable, highly controllable platform. Surface-enhanced Raman spectroscopy (SERS) offers the advantage of obtaining vibrational spectroscopic information about analytes in aqueous matrices with negligible background. The mating of electrophoretic separations with vibrational spectroscopy on microfluidic devices will combine the chromatographic efficiency of capillary electrophoresis (CE) with the analyte "fingerprinting" capability of detailed structural information. By utilizing SERS as a means of detection, this work will help yield redress for the hindrances of electrophoretic separations, including uncertainty in analyte band identification due to changing migration times as well as compromised detection sensitivity for nonfluorescent analytes. Our work represents the first steps toward developing CE-SERS on a microfluidic platform with a region of novel metal-pliable polymer nanocomposite SERS substrate fabricated directly into the device. Via this integration of the detection region directly into the portable separation platform, we build upon the vast development of architectures and separations methods already established in PDMS devices by other researchers. Neither chromatographic nor spectroscopic performance substantially suffers with nanocomposite integration; our systems show efficiencies of 350 000 plates/meter and spectral reproducibility showing less than 10% RSD among devices. This work thereby adds a sensing method with detection of femtomoles of analyte to the arsenal for polymeric microfluidics. Considerable investigation has led to identification of solvent conditions amenable to both separation and SERS sensing, as partitioning of analytes onto the nanocomposite surface is crucial to detection.

*Keywords:* capillary electrophoresis, lab on a chip/microfluidics, polymers and plastics, surface-enhanced Raman
*Application code:* general interest
*Methodology code:* microfluidics/lab on a chip

### A NOVEL APPROACH TO HIGH-EFFICIENCY MULTIDIMENSIONAL ELECTROPHORESIS OF SDS-DENATURED, DYE-LABELED PROTEINS IN A DISPOSABLE POLY (METHYL METHACRYLATE)-BASED MICRODEVICE

***Hamed Shadpour*** ***[*]*** ***and Steven A. Soper***

[*]*Department of Chemistry, Louisiana State University, Baton Rouge, LA 70803-8413, USA*

Reducing the size of bioanalytical devices through microfabrication techniques allows for shorter analysis times and reduced reagent demand. This work focuses on the design, fabrication, and application of polymer-based microdevices for two-dimensional separation of proteins. Poly (methyl methacrylate) (PMMA) was chosen as the polymer substrate material due to its low fluorescence background levels, low adsorption of biomolecules, and low assembly temperatures. A two-dimensional microfluidic device was produced by a molding die fabricated using micromilling techniques. The separation length of each dimension was 3.0 cm and 1.0 cm for gel electrophoresis (GE) and micellar electrokinetic chromatography (MEKC), respectively. Channels were 50 $\mu$m deep and 20 $\mu$m wide. Electrokinetic injection and separation were used with field strengths ranging from 100 to 500 V/cm. Gel electrophoresis of denatured proteins (30 kd to 104 kd) was conducted in the PMMA microdevice followed by MEKC as the second dimension. A mixture of proteins was labeled with Alexa Fluor 633 and purified by HPLC prior to use. LIF was accomplished with excitation at 633 nm. Denaturing of the proteins was performed before loading into the microchip. Some reagents were investigated to suppress the electroosmotic flow (EOF) and protein adsorption or agglomeration during separation to obtain better reproducibility. Both continuous- and pulsed-sample transferring from gel to MEKC was studied and optimized. The total separation time was less than 1 minute in the MEKC domain, and a few minutes for the gel dimension. A novel programed matrix-assisted optimization technique was applied during the process to optimize the separation. With combination of GE with MEKC in PMMA microdevice, all proteins in mixture were separated in a few minutes.

*Keywords:* electrophoresis, lab on a chip/microfluidics, nanotechnology, protein
*Application code:* bioanalytical
*Methodology code:* microfluidics/lab on a chip

### A PORTABLE POSTCOLUMN ION CHROMATOGRAPHY-BASED ANALYZER FOR MONITORING HALOACETIC ACID CONCENTRATIONS IN DRINKING WATER

***Greg T. Anderson,*** ***[*]*** ***Gary L. Emmert, Gija Geme, and Paul S. Simone***

[*]*Department of Chemistry, The University of Memphis, 213 Smith Chemistry Building, Memphis, TN 38152, USA*

Currently, the USEPA approves three methods for the determination of haloacetic acids (HAA) in drinking water. There are five haloacetic acids currently regulated by the EPA. They are monochloroacetic acid (MCAA), dichloroacetic acid (DCAA), trichloroacetic acid (TCAA), monobromoacetic acid (MBAA), and dibromoacetic acid (DBAA). EPA 552.1 uses liquid-solid extraction followed by derivatization to the corresponding methyl esters with GC-ECD detection. EPA 552.2 and EPA 552.3 use liquid-liquid extraction followed

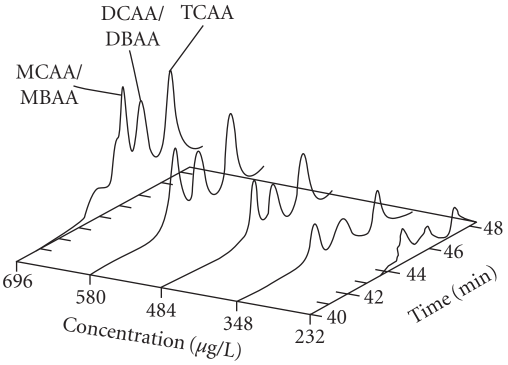

Figure 2: A 3D figure of five PCIC chromatograms with varying concentrations.

by derivatization and analysis on a GC-ECD. A sample set with calibration standards, check standard and samples takes approximately an hour for preparation, two hours for the derivatization step, and an hour for sample cleanup and then injection. These EPA methods work very well for quarterly or weekly samples, but not for faster, perhaps hourly, sampling rates. An instrument was constructed to analyze HAAs using ion chromatography (IC) separating the HAAs in time, and a modified Fujiwara chemistry using nicotinamide and sodium hydroxide to derivatize the HAAs to a fluorescent product. A key advantage of the postcolumn IC method is that the sample preparation time is minimal, and preliminary data indicates that common drinking water ions such as fluoride ion, bromide ion, chloride ion, sulfate ion, bromate ion, nitrite ion, nitrate ion, and orthophosphate do not interfere with postcolumn derivatization. This means that the PCIC chromatograms are much simpler than those using more traditional approaches such as IC with membrane suppressed conductivity detection. The portable analyzer can be built from commercially available parts, including the IC pump, injection valve, column, and postcolumn FIA techniques.

Figure 2 is a 3D plot of the five stopped flow PCIC chromatograms. The unresolved peaks of MCAA/MBAA and DCAA/DBAA along with TCAA are the three peaks shown. The total concentration of each run is listed. The farthest right chromatogram is of the following concentrations: 73 μg/L MCAA/MBAA combined, 120 μg/L DCAA/DBAA combined, and 38 μg/L TCAA. Though the current setup has resolution issues, concentrations as low as 11 μg/L TCAA and 21 μg/L of MCAA/MBAA combined have been detected. Additional studies aim at resolving individual HAA species and lowering the MDL values to the single μg/L range.

*Keywords:* derivatization, environmental/water, fluorescence, ion chromatography

*Application code:* environmental

*Methodology code:* liquid chromatography

### FAST ION CHROMATOGRAPHY USING SHORT COLUMNS

***Charles A. Lucy*** [*] ***and Sarah Pelletier***

[*] *Department of Chemistry, Gunning/Lemieux Chemistry Centre, University of Alberta, Edmonton, Alberta, Canada T6G 2G2*

Laboratories are under constant pressure to improve sample throughput and boost productivity. Further, applications such as on-line monitoring of chemical processes require techniques with fast analysis response times. These pressures have lead to substantial advances in reversed-phase liquid chromatography that shorten analysis times. The primary thrusts have been in the use of high-permeability monolithic columns and the use of short particles packed with small (less than 2μm) particulate packings.

In contrast, ion chromatography (IC) columns are generally 15–25 cm long and packed with rather large (greater than 5μm) particles. This results in typical IC separations being 10–15 minutes long. Previously it was demonstrated that 5 cm ×4.6 mm i.d. silica monoliths (Chromolith) could separate 7 inorganic anions in less than 30 seconds (P. Hatsis and C. A. Lucy, *Anal. Chem.*, vol. 75, pp. 995, 2003). However, such separations required flow rates greater than 10 ml/min, and so are not practical in most laboratories. This presentation discusses the use of short monolithic and small particulate columns for ion chromatographic analyses. Short (0.5–1.0 cm) monolithic columns generate sufficient chromatographic efficiency so that 7-component separations can be performed in less than 2 minutes at 3 ml/min. Efficiencies and detection limits are in the range of 70 000 plates/m and 1 μm.

Further, the pressure drop of such columns is so low that low-pressure syringe pumps can be used.

Alternatively, 3 cm columns of 1.8 μm silica reversed-phase packing enable full anion separations to be performed in a few minutes under conventional flow conditions. The enhanced base stability of bidentate C-18 silica phases enables fast anion separations to be performed with typical IC eluents.

*Keywords:* high-throughput chemical analysis, ion chromatography, liquid chromatography

*Application code:* high-throughput chemical analysis

*Methodology code:* liquid chromatography

### REAL-TIME PROBING OF MEMBRANE TRANSPORT IN LIVING MICROBIAL CELLS USING SINGLE NANOPARTICLE OPTICS AND LIVING CELL IMAGING

***X. Nancy Xu***

*Old Dominion University, 201 Alfriend Chemistry Building, 4541 Hampton Boulervard, Norfolk, VA 23529, USA*

All living organisms appear to be equipped with unique membrane transport apparatus that protects the cells from hazardous and noxious compounds. Membrane permeability and active extrusion systems in many living organisms, such as bacteria, yeast, molds, and mammalian cells, play a crucial role in controlling the accumulation of specific intracellular substances, leading to the cellular self-defense mechanism that can resist incoming noxious compounds. Despite extensive studies, the mechanisms of membrane transport in living cells still remain incompletely understood. Currently, the sizes of membrane pores rely upon X-ray crystallography measurements, which are limited by the difficulties of crystallization of membrane proteins and are unable to provide real-time kinetic information of sized change of membrane pores in living cells. To address this technique challenge, we have developed a new tool that uses silver (Ag) nanoparticles as nanometer probes to determine the sizes of substrates that can be transported through membrane of living microbial cells and to measure accumulation kinetics of the substrates in real time at the single-cell resolution. In this study, we directly measure real-time sized change of membrane porosity and permeability of single living cells at the nanometer scale using the intrinsic color index (surface plasmon resonance spectra) of silver (Ag) nanoparticles as the nanometer-sized index. The result shows that Ag nanoparticles with size up to 80 nm are accumulated in living microbial cells, demonstrating that such larger nanoparticles are transported through the inner and outer membranes of the cells. This permeable size is about 50 times larger than conventional antibiotics.

*Keywords:* bioanalytical, biotechnology, imaging, nanotechnology
*Application code:* nanotechnology
*Methodology code:* microscopy

### ETHERNET-CONTROLLED VISION-BASED PARTICLE ANALYZERS

***Bruno C. Albano***

*Canty Inc, 6100 Donner Road, Lockport, NY 14094, USA*

On-line vision-based particle analyzer for solids and liquids controlled via ethernet for real-time analysis is tackled in this paper. Traditional systems have been limited to taking samples to a lab where timely analysis is very difficult. The lag time in obtaining results is often too long for the requirements of process control. New ethernet-controlled systems measure true 2D size and shape of crystals and cells from one micron to several millimeters. Cell viability can now be done on line or at line. All of the vision-based systems discussed here contain a fused glass to metal seal which is sanitary and can be cleaned in place. The fiber-optic lighting systems use "cold" light so no heat is transferred into the process causing "bake-on" or contamination. In the past similar vision systems have been limited to smaller size ranges due to having only a single calibration. If the lens settings were changed, the system would need to be recalibrated. The ethernet systems here have the ability to store many calibrations with different optical settings which can be repeated accurately time and time again. These calibrations are stored on the image processor where they can be selected through the software. Results of the full particle size distribution along with shape information such as aspect ratios, circularity, and more can be outputted to a control system to automatically adjust process parameters. All data can be stored digitally for a historical record along with operators viewing the process in real time for a visual verification.

*Keywords:* bioanalytical, laser, microspectroscopy, process control
*Application code:* process analytical chemistry
*Methodology code:* microscopy

### AN AUTOMATED AND SIMPLE METHOD FOR THE DETERMINATION OF SULFUR COMPOUNDS IN BEVERAGE-GRADE CARBON DIOXIDE

***Robert M. Hillard,[*] Robert J. Mathieu, and Peter S. Mathieu***

[*]*GOW-MAC Instrument Company, 277 Brodhead Road, Bethlehem, PA 18020, USA*

The identification and quantitation of sulfur impurities is of vital importance to many industrial processes. Specialty, electronic and medical gases, catalysts, and petroleum products represent but a few of the many areas of worldwide commerce detrimentally affected by sulfur contamination. None of these, however, touches the pulse of the human population as much as the beverage industry.

Carbon dioxide is a major ingredient in the soft drinks enjoyed throughout the world. Sulfur contamination of raw carbon dioxide is of tremendous concern to beverage bottlers because of the obvious taste and considerations, as well as the enormous fallout of a publicized product recall. The industry is in need of a definitive method of sulfur speciation and quantitation one that is reliable, yet fast and easy to use.

A method and device will be presented that combines the speciating power of a gas chromatograph with the user friendliness of a process analyzer. The method utilizes a newly designed flame photometric detector, coupled with a proprietary speciation system capable of detecting and reporting COS, $H_2S$, $CH_3SH$, $SO_2$, and more, and is free from carbon dioxide interference. Detection limits are well below ISBT specifications. The device is designed for hands-free operation via the incorporation of a computerized user interface. Calibration and operation are automatic, resulting in fast and easy process monitoring and reporting of sulfur impurity data.

*Keywords:* beverage, process analytical chemistry, process control, quality
*Application code:* process analytical chemistry
*Methodology code:* sensors

### STREAMING GAS PROCESS DATA TO THE OUTSIDE WORLD: APPLICATIONS OF OLE FOR PROCESS CONTROL TO FTIR GAS ANALYSIS

***Jay P. Roberts***

*Thermo Electron Corporation, 5225 Verona Road, Madison, WI 53711, USA*

FTIR spectroscopy is well established for gas-phase research in such diverse applications as automobile exhaust emissions, contaminant detection in breathing oxygen or semiconductor gases, and combustion science. More recently, dedicated FTIR gas analyzers are finding widespread use monitoring gas streams in industrial chemical plants for online or near-line process control applications. In these applications, the FTIR unit must communicate extensively with a networked computer system controlling the chemical process for instrument control, synchronizing the FTIR data to external sensors, and to integrate the resulting data into a common database. OPC (OLE for process control), the computer communications standard, can simplify and facilitate this implementation. The current study will discuss instrument control, synchronized data collection, and real-time data output to a remote control network.

*Keywords:* environmental air, FTIR, gasoline, monitoring
*Application code:* process analytical chemistry
*Methodology code:* vibrational spectroscopy

### CONTINUOUS ON-SITE MONITORING OF THE FERMENTATION OF BEER BY ION MOBILITY SPECTROMETRY

***Joerg Ingo Baumbach,* Joachim Jung, and Wolfgang Vautz***

**ISAS Institute for Analytical Sciences, Bunsen-Kirchhoff-Street 11, Dortmund 44139, Germany*

The fermentation process of beer is the time-consuming part of the complete beer production process and takes 5 to 6 days. The end of the process is determined by fixed concentrations of diacetyle and 2, 3-pentandion. Because of the fact that both substances smell like rancid butter, their concentrations have to be decreased consequently below the human odor threshold. Furthermore their ratio is a measure for potential microbiotic contaminations which can occur during the fermentation process. In a brewery, such concentration values are determined by taking and preparing samples manually before a gas chromatographic headspace analysis. The full procedure takes about 3 hours. Therefore, this kind of analysis is made only once a day and no continuous concentration data are available.

We present the results of examinations using a 10.6 eV UV ion mobility spectrometer equipped with a gas chromatographic column for preseparation purpose. The characterization of the analytes selected in the matrix of different beer types will be described. Especially, the on-line determination of diacetyle and 2, 3-pentandion will be considered in detail. Using the method developed, the fermentation process can be stopped immediately at the time when the concentration threshold is reached. Thus, the beer production process can be optimized using ion mobility spectrometers on the process line directly.

*Keywords:* process analytical chemistry, process control, sensors, spectroscopy
*Application code:* process analytical chemistry
*Methodology code:* sensors

### ONLINE CHEMICAL COMPONENT CHARACTERIZATION AND CONCENTRATION MEASUREMENT FOR PROCESS CONTROL USING RAMAN SPECTROSCOPY

***Hongqi Yuan,* Theodore M. Garver, and George Sedgwick***

**Alberta Research Council, 250 Karl Clark Road, Edmonton, Alberta, Canada T6N 1E4*

The online measurement of Raman spectra to identify and quantify chemical species has been investigated for several industrial processes. The Raman method is particularly suited to the measurement of small, oxygenated molecules such as $ClO_2$, hydrogen peroxide and sulfate that have unique well-defined Raman spectral signatures. We have used Raman spectroscopy to characterize and measure an array of chemical species that are used or formed during different industrial processes. The technique offers particular promise for unraveling speciation in a redox series such as hydrosulfite, sulfite and sulfate ions, as well as the species like chlorite, chlorate and chlorine dioxide. The potential of an oxidative-reductive process is conveniently determined using Raman peak intensity ratios. This technique has been used to accurately measure the residual hydrogen peroxide in pulp bleaching processes. Measurements of the various chemical species have provided new information, new process variables, for control of the industrial process. Successful implementation of on-line Raman analyzers in industrial settings requires sample preparation and data extraction/reduction techniques that will be discussed.

*Keywords:* characterization, on-line, process control, Raman
*Application code:* process analytical chemistry
*Methodology code:* vibrational spectroscopy

### NOVEL INTERLEAVED DATA COLLECTION METHOD FOR IMPROVED CHEMICAL IMAGING

***Joseph W. Schoppelrei,* Haber Ken, Eunah Lee, and E. Neil Lewis***

**Spectral Dimensions, Inc, 3416 Olandwood Court, Suite 210, Olney, MD 20832, USA*

Near-infrared chemical imaging instruments incorporating a solid-state tunable filter and focal plane array (FPA)

detector collect data by tuning the filter and capturing the response over a set of sequential wavelengths. Raw datasets are typically corrected to remove the instrument response function from the sample spectra. Traditionally, the sample, background, and dark datasets are collected via sequential data collections. Environmental changes between these collections may contribute nonrandom noise to the corrected sample spectra. While negligible in most experiments, this component may become dominant for more challenging measurements. In such cases, increasing signal averaging may actually result in degraded spectral quality. An interleaved collection approach can be automated by use of a mechanical device to move the background and dark references into the optical path of the instrument without disturbing the sample. All three measurements are constructed concurrently as the filter tunes through a single wavelength, effectively eliminating instrumental drift effects and improving the sensitivity of the measurement. Using this method, signal-to-noise values of 30 000 : 1 have been achieved for individual spectra, and the remaining noise is predominantly random in nature, even over protracted data acquisition times. Additionally, by reducing the number of filter-tuning sequences the interleaved collection scheme increases the duty cycle of the instrument and reduces the time required to produce a corrected spectral image cube relative to the standard approach. Data will be presented highlighting the performance improvements achievable using this approach, and demonstrating how it is particularly well suited to improving process analytical measurements.

*Keywords:* automation, imaging, instrumentation, near infrared
*Application code:* general interest
*Methodology code:* surface analysis/imaging

### OPTIMIZATION OF EXPERIMENTAL CONSTRAINTS WHEN COUPLING AUTOMATED POLARIZED LIGHT MICROSCOPY WITH RAMAN MICROSCOPY

***Carrie A. Lendon,* Douglas L. Elmore, Chad L. Leverette, John T. McDonald, and Sean A. Smith***

**Cargill Scientific Resources, 7101 Goodlett Farms Parkway, Cordova, TN 38016, USA*

Automated polarized light microscopy coupled with Raman microscopy on the same platform is a powerful analysis strategy for monitoring chemical systems. The benefits of having these technologies coupled onto one platform are the following. (1) The same spatial region of a given sample can be observed and analyzed by two independent, complementary techniques. (2) Auto-PLM images can quickly reveal areas of interest amenable to Raman analysis reducing the amount of time spent in the undesirable "trial and error" hunt for a useful sample location. The auto-PLM image can also be used to ensure representative sampling. (3) Fast identification of unknowns or contaminants is possible due to the improved chemical contrast of the auto-PLM image. (4) The combined technique validates the results from each independent technique. (5) The corresponding data from both techniques can be correlated against each other. Auto-PLM provides birefringence information as a function of retardance, which is calculated as the birefringence of the sample multiplied by the sample thickness (more accurately, the penetration depth of the polarized radiation through the sample). Birefringence is an intrinsic property of each of the individual sample components that can be interpreted as a given component's degree of structural order. For qualitative applications, the auto-PLM component provides high resolution, colored images of the sample that represents spatial variations in birefringence. This chemical contrast greatly improves the subsequent Raman analysis through the benefits already mentioned. The dependence on sample thickness for the retardance information obtained is an experimental constraint that must be addressed if true quantitative applications of this technology are to be realized. This talk will discuss this experimental constraint and provide solutions to dealing with this issue for quantitative applications. Applications covering reflection and transmission mode of the microscope will be provided. Examples of chemical imaging quantitation of chemical components in heterogeneous systems will be shown and discussed.

An application of PCA and MCR chemometric techniques will be demonstrated and discussed.

*Keywords:* imaging, microscopy, microspectroscopy, Raman
*Application code:* others
*Methodology code:* microscopy

### INSPECTING METALS FOR CONTAMINATION AND CORROSION USING FTIR REFLECTANCE SPECTROSCOPY

***George L. Powell,* Michael Beecroft, Richard L. Cox, Phillip Mattison, and Martin M. Szczesniak***

**Y-12 National Security Complex, PO Box 2009, Bear Creek Road, Oak Ridge, TN 37831-8096, USA*

In the process of measuring the growth of uranium oxide films on uranium metal, and the thickness of organic films on metals, using specular reflection at 75° and 45° angles of incidence with and without polarization of the incident light, to measure the film thickness with subnanometer resolution, the inadvertent contamination of specimens and gold reference mirrors by particulate matter was detected in the spectra. The sources of this particulate matter included powder from laboratory gloves, dust from the air, and pitting corrosion of the metal substrates. This paper describes the application of remote-sensing specular and diffuse reflectance heads for a surface inspection machine to the characterization of metals having particulates on their surfaces as a result of dust or pitting corrosion. Applications of these techniques to the discrimination between particulate contamination and coherent film growth are described using applications such as powdered glove contamination by organic and inorganic compounds, and the pitting corrosion (rusting) of iron is described.

**ACKNOWLEDGEMENT**

This work was managed by BWXT Y-12, L. L. C. for the US Department of energy under contract DE-AC05-00OR22800.

*Keywords:* FTIR, infrared and Raman, materials characterization, surface analysis
*Application code:* materials science
*Methodology code:* vibrational spectroscopy

### CHARACTERIZATION OF ORGANIC HYDROGEN GETTERS BY HYDROGEN GAS TITRATION, FT-MS, DYNAMIC MS, AND UHV FTIR GAS ANALYSIS

***Norman R. Smyrl,** *Dean Davis, Quirinus G. Grindstaff, Kenneth D. Nicklas, and George L. Powell***

**Y-12 National Security Complex, Bear Creek Road, PO Box 2003, Oak Ridge, TN 37831-8096, USA*

DEB (1, 4 bis (phenyl ethynyl) benzene) or DPB (diphenyl butadiene), typically mixed with 25 wt% C - 1 wt% Pd, is made into organic hydrogen getters in porous pellet form. The hydrogen gettering reactions are effective and irreversible since the hydrogen is converted into a hydrocarbon. The hydrocarbon products formed by hydrogen addition to pairs of triple bonds by this heterogeneous catalysis are many and complex and influence the rate and capacity of these getters through eutectic formation and volatility of reduction products. This paper describes the measurement of the extent and rate of hydrogen consumption by DEB and DPB organic hydrogen getters using isobaric and pressure drop gas burette methods, and the integration of this technique with a Fourier transform mass spectrometer (FTMS) for gas analysis as modified by Siemens applied automation to detect masses as low as mass 2, dynamical measurements with a residual gas analyzer MS, and a vacuum FTIR having an ultrahigh-vacuum (UHV) white cell (2 m, 200 mL) for the determination and characterization of volatile reaction products as a function of reaction extent.

**ACKNOWLEDGEMENT**

This work was managed by BWXT Y-12, L. L. C. for the US Department of energy under contract DE-AC05-00OR22800.

*Keywords:* FTIR, gas, mass spectrometry, trace analysis
*Application code:* others
*Methodology code:* vibrational spectroscopy

### MONITORING OF SULFURIC ACID DECOMPOSITION BY FOURIER TRANSFORM INFRARED SPECTROSCOPY IN THE SULFUR IODINE THERMOCHEMICAL REACTION FOR THE PRODUCTION OF HYDROGEN

***Dion Rivera***

*Sandia National Laboratories, PO Box 5800, Albuquerque, NM 87185-0886, USA*

A potential way to produce large amounts of hydrogen for energy needs is the thermal breakdown of sulfuric acid ($H_2SO_4$) to oxygen, water, and sulfur dioxide ($SO_2$). The sulfur dioxide can then be reacted with iodide to produce hydrogen iodide and ultimately hydrogen. Strategies for the breakdown of sulfuric acid vary and sulfur trioxide ($SO_3$) is also present as a byproduct. In order to insure the maximum amount of hydrogen is being produced, the amounts of $SO_3$, $SO_2$, $H_2SO_4$, and water in the process stream need to be monitored periodically. Fourier transform infrared (FT-IR) spectroscopy is well suited to detection of these gas-phase species as they all contain strong infrared modes in the 1700 to 1200 wavenumber region. However, the reactive nature of the gases and the high temperatures at which the reactions are run, greater than 600°C, make implementation of FT-IR process monitoring challenging, particularly in regard to infrared window materials. This talk will focus on modifications to typically FT-IR window materials to allow them to be more robust in the environment of interest. One modification that is being pursued is evaporation of a thin gold coating, ~ 20 nm, onto $CaF_2$ windows to make them less susceptible to chemical attack but sill allows for transmission of infrared radiation. In addition, seal materials and cell body materials that can withstand both high temperatures and oxidative conditions will be discussed. This talk will also discuss the chemometric techniques used to calibrate the system and maintain the calibration as instrument drift or degradation of the window materials causes the original calibration to become invalid. Maintenance of calibration is crucial for this system to both maximize the life of the window materials and prevent down time of the system for recalibration.

*Keywords:* chemometrics, process monitoring, vibrational spectroscopy
*Application code:* process analytical chemistry
*Methodology code:* vibrational spectroscopy

### FTIR VALIDATION USING EPA METHOD 301 AND 320 FOR HCL AND HF AT A MUNICIPAL SOLID WASTE INCINERATOR

***Peter G. Zemek***

*MIDAC, 130 Mccormick Avenue, Costa Mesa, CA 92626, USA*

Matrix specific effects require EPA Method 320 compliance testing for validation of the FTIR sampling system, instrumentation and analysis method. EPA Method 301 is an important statistical validation technique for validating a sampling methodology to a specific matrix type.

A municipal solid waste incinerator was tested for HCl and HF using extractive FTIR for compliance testing. Additionally, a spiking regimen according to EPA Method 320 was expanded to include EPA Method 301 validation using spike studies. Two FTIRs will be used to measure baseline HCl and HF levels and cumulative spiked materials. Recoveries, statistical data, equipment, and sampling techniques will de discussed.

*Keywords:* environmental air, FTIR, sampling, speciation
*Application code:* environmental
*Methodology code:* vibrational spectroscopy

### SCANNING FOR EXTINCT ASTROBIOLOGICAL RESIDUES AND CURRENT HABITATS (SEARCH) USING INTEGRATED COMPUTATIONAL IMAGING

***Robert A. Lodder*[*] *and Clay Harris***

[*]*Department of Chemistry, University of Kentucky, A123 Astecc Building, Lexington, KY 40506-0286, USA*

SEARCH uses Hadamard encoded active-excitation remote sensing to seek evidence of extinct life and potential habitats. Combining UV, visible, and near-IR spectroscopic integrated sensing and processing with parallel data-analysis algorithms, SEARCH is designed to explore and quantitatively assess a local region on the surface of a planet or moon as a potential habitat for life, past or present. In the course of collecting geological data, SEARCH spectrometry can investigate planetary processes of relevance to past habitability, including the role of water. In addition to its own investigations, SEARCH can guide a rover to areas of interest for application of the full suite of analyses. A sufficient number of discrete reflections at different wavelengths from a target provide a unique profile. SEARCH uses an array of laser diodes to obtain profiles of a wide variety of organisms and of fossils or other remnants of once-living organisms. In addition, amino acids, carbohydrates, PAHS, and more complex organic compounds can be identified. Minerals, such as amphiboles, silicates, limestone, jarosite, hematite, oxides, feldspars, plagioclase, smectite, halites, apatite, hydroxyapatite, sulfides, sulfates, and others are detectable. Water can be found in liquid or solid phase, enabling a rover to "follow the water" in its search for life. The data collected by SEARCH are compared to an extensive data bank library of responses from selected terrestrial materials assembled prior to launch. By "tokenizing" the data, SEARCH can obtain the greatest significance from the data and greatly reduce the traffic and bandwidth required for its transmission to Earth for comparison with the library bank. A diagram of the SEARCH instrument is attached.

*Keywords:* computers, laser, spectrometer, UV-VIS absorbance/luminescence
*Application code:* environmental
*Methodology code:* vibrational spectroscopy

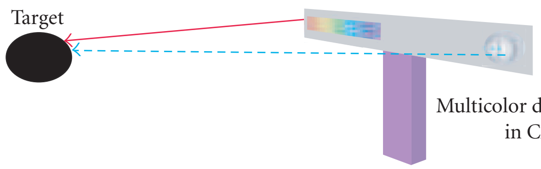

Figure 3

### CALLI 2002: NEW AUTOMATED WEIGHING AND LIQUID HANDLING SYSTEM

***Jim Schools*[*] *and Clifford A. Olson***

[*]*Zinsser Analytic, 19145 Parthenia Street, Suite C, Northridge, CA 91324, USA*

CALLI 2002 is a new robotic calibrating, weighing, and liquid handling system with a built-in robotic arm. It reads barcodes on vials, microtubes, and plates, determines the tare weight, the weight of the contents and then calculates the volume of the solvent required for concentration normalization.

The user can choose whether they require an integrated weighing cell set into the workbench or a balance located to the side of the workbench still with full access from the handler for complete automation of the weighing process. Eight pipetting probes offer a full range of liquid handling functions. Powder handling is available as an option as are other options including vortexing, sonication, automated capping and decapping, cherry picking software, and more. The CALLI 2002 is available with the CALLI Windows 2000/XP software which controls all robotic functions and keeps its own database for weighing data, compound codes, and so forth. CALLI 2002 can be linked into existing networks and communicate with most commercial databases.

*Keywords:* automation, robotics, sample handling/automation, sample preparation
*Application code:* pharmaceutical
*Methodology code:* sampling and sample preparation

### APPLICATION OF A RECENTLY DESIGNED ROBOTIC LIQUID AUTOSAMPLER FOR THE ANALYSIS OF TRACES OF CONTAMINANTS IN WATER BY LVI-GC-MS

***Fausto Munari,*[*] *Stefano Pelagatti, and Fausto Pigozzo***

[*]*Thermo Electron Corporation, Strada Rivoltana, Km 4, Rodano, Milano 20090, Italy*

Recent European legislation for the regulation of the maximum tolerable amount of contaminants in deep water in industrial areas has strongly increased the demand for sensitivity and speed in the analytical methods. The sensitivities normally required call for laborious and time-consuming sample preparation prior the injection into the analytical system. The possibility of injecting larger amounts of sample would require shorter analytical cycle. Various sample preparation methods are normally used in this application, among which liquid-liquid extraction and SPE. Large volume injection in combination with in-vial liquid-liquid extraction provides the advantage of an easy-to-automate sample-preparation step, with automatic transfer to the analytical system (GC or GC/MS). This combination, automated by a robotic autosampler, delivers the most powerful solution in terms of simplicity and level of sensitivity achievable. By means of automated large sample volume injection, the solvent desolvation step completely replaces the sample reconcentration step, otherwise required, without compromising the level of sensitivity achievable. Sample preparation is hence made easier, more precise and reliable. With adequate instrumentation and injection procedure, the technique is also compatible with MS systems, despite the large amount of solvents injected in the chromatographic system. This paper shows results obtained using automatic in-vial extraction technique followed by large volume injection, applied to various injection systems for the analysis of pollutants in water. The ability of this new-generation autosampler to completely use the material available after liquid-liquid extraction while permitting advanced large volume injection techniques is also described. The resulting analytical methods are proved to be comparable with standard official methods, as far as sensitivity is concerned, but offer the advantage of a shorter sample prep time and full automation.

*Keywords:* capillary GC, instrumentation, sample preparation, trace analysis
*Application code:* environmental
*Methodology code:* gas chromatography

### NEW PORTABLE SPME PASSIVE AIR MONITORING AND FIELD SAMPLING DEVICE

***Robert E. Shirey* and Leonard M. Sidisky***

**Supelco, Inc, 595 North Harrison Road, Bellefonte, PA 16823, USA*

A new solid-phase microextraction device has been developed for field sampling and passive air monitoring. This device is about the size of a pen and contains a PTFE sealing unit. The cap containing the sealing unit also can be used as a shield for air sampling or completely removed to sample in water. After extraction, the cap with the spring-loaded PTFE seal can be pushed into the lock position to store the sample for analysis at a later time. The unit contains an easy-moving mechanism to adjust the fiber in multiple positions for passive air monitoring using time-weighted averaging (TWA). The positions are set to put the fiber at a fixed position from the needle opening. The distance from the opening and the time of sampling determines the amount of air that contacts the fiber. The amount of the analytes adsorbed can be compared with the amount of air that contacted the fiber to obtain the average concentration of analytes in the air over the given time period.

The sampling device uses standard SPME fiber assemblies, and fiber assemblies can be exchanged in the unit. The design will allow this device to be compatible with the CTC analytics autosampler in the near future.

This presentation will provide a detailed look at the portable sampling device. Applications of the device as a passive air monitoring device and as a field sampling device will be presented.

*Keywords:* air, environmental analysis, extraction, solid-phase extraction
*Application code:* environmental
*Methodology code:* sampling and sample preparation

### PREVENT DAMAGE TO YOUR PURGE-AND-TRAP SYSTEM BY PRESCREENING WITH STATIC HEADSPACE

***Mark E. Krigbaum* and Brian T. Wallace***

**Teledyne Instruments - Tekmar, 4736 Socialville Foster Road, Mason, OH 45040, USA*

Much time and money is lost in environmental laboratories bringing purge-and-trap gas chromatography mass spectrometer (PT-GC/MS) systems back in operation from a sample containing dangerously high levels of volatile organic contaminants. This is often a problem where water and soil samples originate from the leaking underground storage tank (LUST) remediation. This EPA program requires testing of soil and water for benzene, toluene, ethylbenzene, and xylenes (BTEX), as well as other gas range organic (GRO) compounds. These compounds are often markers of a fuel leak from underground storage tanks and need to be monitored. But often the levels are much higher than the normal operating range of purge and trap. EPA method 5021, a static headspace gas chromatography flame ionization (GC/FID) method, is recommended for screening of volatile organic analytes prior to PT-GC/MS analysis because of its speed, simplicity, and minimal carryover. This method can be used to quickly predetermine dilution levels or calibration ranges necessary for PT-GC/MS analysis, saving time and preventing costly damage to the volatiles system.

*Keywords:* gas chromatography, headspace, purge and trap, volatile organic compounds
*Application code:* environmental
*Methodology code:* gas chromatography

Table 1

| Averaging time | Detection limit |
| --- | --- |
| 1 | 1.6 |
| 5 | 0.8 |
| 16 | 0.4 |
| 30 | 0.3 |
| 60 | 0.24 |
| 120 | 0.22 |

### REAL-TIME BACKGROUND MERCURY MONITORING IN AIR USING PORTABLE ZEEMAN ATOMIC ABSORPTION SPECTROMETER

***Nikolay R. Mashyanov,* Vladimir V. Ryzhov, Serguei E. Sholupov, Joseph Siperstein, and Akira Yasutake***

**Lumex Ltd, 19 Moskovsky pr, Saint Petersburg 190005, Russia*

A technique of the direct continuous analysis of the background mercury concentration in air without any preconcentration, amalgamation, or trapping options using a Zeeman atomic absorption spectrometer RA-915+ is presented. The RA-915+ Zeeman analyzer provides continuous determination of the mercury concentration with a detection limit (DL) of 1.5 $\approx$ 2 ng/m$^3$, the response time being 1 second. The DL is calculated as threefold standard error and depends on averaging time (tav) as DL(tav) = DL1/[root](tav), where DL1 is determined for the averaging time of 1 second.

Statistical accumulation of the analytical signal by the RA-919P software enables reducing the real DL of measurements below 1 ng/m$^3$ (see Table 1).

The examples of real-time background mercury monitoring in air and continuous automobile survey in different regions are given.

*Keywords:* elemental analysis, environmental air, mercury
*Application code:* environmental
*Methodology code:* atomic spectroscopy/elemental analysis

### A PORTABLE GC FOR INDOOR VOC DETERMINATIONS

***Qiongyan Zhong,* Chunguang Jin, and Edward T. Zellers***

**University of Michigan, 1843 Lake Lila Lane, Apartment B8, Ann Arbor, MI 48105, USA*

The development of a portable GC for determination of complex VOC mixtures at concentrations relevant to indoor air quality (IAQ) investigations is described. The key features of the instrument, which is about the size of a laptop computer, are a 3-stage adsorbent preconcentrator/focuser, series-coupled GC columns with pressure and temperature tunable retention, and a detector consisting of an integrated array of 3 chemiresistor microsensors coated with different gold-thiolate nanoclusters whose responses provide a crude "spectrum" of responses for the eluting vapors. The instrument was calibrated with 20 common IAQ contaminant vapors. These compounds were divided into 3 groups for initial testing of retention times and response patterns. Compounds were calibrated from 0.4–30 ppb, assuming a 1-L preconcentrated air sample. Except for two low-volatility compounds, which had residual carryover problem with the system, calibration curves were linear ($r2 > 0.99$) and response patterns from the three sensors were reproducible. LODs were in the low or sub-ppb range in all cases, with a minimum value of 50 parts per trillion (for d-limonene). Preliminary field surveys have been conducted in several office buildings. With GC-MS standard method as a reference, peaks were identified by combining information of peak profile, retention time, and sensor recognition patterns. Results indicated that the detection limits and chromatographic resolution of the instrument are promising for many IAQ applications.

*Keywords:* environmental air, gas chromatography/mass spectrometry
*Application code:* environmental
*Methodology code:* gas chromatography

### A NOVEL AUTOMATIC LUMINOUS BACTERIA-BASED EARLY WARNING ONLINE MONITOR FOR WATER TOXICITY MEASUREMENTS

***Luca Sanfilippo,* Pompeo Moscetta, Shimon Ulitzur, and Nirit Ulitzur***

**SYSTEA Srl, Loc Paduni, Anagni (FR) 703012, Italy*

An innovative automated online water quality monitoring system to detect $\mu$g/L concentrations of toxic organic and inorganic chemical agents in surface, ground water, or treated drinking water is presented. The analyzer utilizes a renewable luminescent bacteria bioassay (Toxscreen, Barcelona, Spain) recently verified by EPA under the ETV program. When the bacteria are mixed with a water sample automatically collected by the analyzer, their light production, which is directly tied to cell respiration and other critical metabolic pathways, decreases in proportion to the toxicity (concentration of toxic chemicals) in the sample.

At 14-day intervals, the instrument is resupplied with a fresh inventory of liquid assay buffers and a freshly hydrated suspension of the freeze-dried luminescent bacteria. Automatic safeguards have been engineered into the system to assure reagent and data quality and appropriate instrument functioning. The analyzer was tested in lab and under field conditions in Italy and Israel, using a strict data validation procedure. The online analyzer is particularly suited for raw drinking water monitoring, to provide early warning of dangerous spills, accidents, sabotage, and bioterrorism.

*Keywords:* luminescence, monitoring, on-line, toxicology
*Application code:* environmental
*Methodology code:* fluorescence/luminescence

### MONITORING BIOLOGICAL CONTAMINATION (PYROGENS AND NUCLEASES) IN WATER

***Julien Bole,* Ichiro Kano, and Stéphane Mabic***

**R&D Department, Millipore Lab Water Division, Bp307 Saint Quentin-Yvelines 78054, France*

Regulatory bodies have enforced the monitoring of several water quality parameters, including ionic, organic, and bacteria levels. Pharmaceutical companies, as well as environmental and clinical laboratories, therefore have to comply with the strict norms and guidelines issued by these regulatory agencies. In addition to the standard quality parameters (resistivity, bacteria, and total organic carbon), other biological contaminants have to be monitored. Norms and guidelines focus on the levels of two major bacterial by-products: endotoxins (or pyrogens) and ribonucleases (RNases). Monitoring the level of pyrogens is particularly critical for pharmaceutical preparations, as endotoxins have a direct effect on human health. Controlling the concentration of RNase in water used in molecular biology laboratories has prompted a number of regulatory bodies to recommend maximum levels of nucleases in high-purity water. Nucleases have been monitored using a cleavable, RNase substrate standard labeled with a green fluorescent probe. Fluorescence generated is directly proportional to the concentration of nucleases present. Endotoxins were monitored using two methods, both based on an end-point chromogenic reaction. The first method is a classical enzyme spectrophotometric test, while the second is an automated microfluidic method. Levels of RNases and endotoxins in various qualities of water were assessed on a routine basis in our laboratories. Data are presented on the reproducibility, the sensitivity, and the ease of use of the assays. The two methods evaluated for endotoxins monitoring were compared in terms of precision and accuracy.

*Keywords:* contamination, monitoring, spectrophotometry, water
*Application code:* regulatory
*Methodology code:* UV/VIS

### MINIATURIZED PORTABLE DEVICES FOR WATER MONITORING

***Leanne Marle* and Gillian M. Greenway***

**Department of Chemistry, University of Hull, Cottingham Road, Hull HU6 7RX, UK*

A chemiluminescence immunoassay for the determination of *E coli* in seawater within a microfluidic device is presented as a highly sensitive portable method for rapid in-situ analysis. *E coli* in seawater is an indicator of fecal contamination; current analysis is time consuming presenting a need for in-situ real-time measurements, enabling faster results, at a low cost and at a minimal risk of contamination as well as providing high temporal and spatial resolution data. The advantages of miniaturization, which includes speed of analysis, low reagent consumption, low waste production, portability and remote operation lead to ideal properties for portable analytical techniques for monitoring *E coli* in seawater. Chemiluminescence (CL) as a method of detection for miniaturized total analytical systems ($\mu$TAS) has the advantage of high sensitivity and simple instrumentation. The combination CL $\mu$TAS with the high specificity of immunoassays is ideal for determining pathogens in the environment.

The analysis comprises of an *E coli* specific antibody immobilized to controlled pore glass packed within a microfluidic device. The immobilization is based on the covalent attachment of the antibody via a mercapto-terminal silane and a heterobifunctional cross-linker. A second *E coli* specific antibody labeled with horseradish peroxidase (HRP) is used to sandwich the antigen. The HRP is then detected using the luminol-hydrogen peroxide chemiluminescence system. The immobilized antibody is then regenerated for each sample. The microfluidic device consists of a network of micronsized channels etched into glass. The reagents are brought together within the device using a miniaturised peristaltic pump situated within a custom built, battery-operated light tight box containing a miniaturised PMT for detection. Optimization of the chemiluminescence and the effects of enhancers have been investigated to produce a sensitive method for the detection of HRP. The specificity of the *E coli* antibody has been studied using an enzyme-linked immunosorbent assay (ELISA) protocol. The optimum loading and regeneration step have also been considered. Initial results present a promising chemiluminescence immunoassay for the fast analysis of *E coli* with high sensitivity. The EPSRC is acknowledged for funding the studentship.

*Keywords:* chemiluminescence, environmental/water, immunoassay, lab on a chip/microfluidics
*Application code:* environmental
*Methodology code:* microfluidics/lab on a chip

### USE OF TISSUE SAMPLES FROM THE MARINE ENVIRONMENTAL SPECIMEN BANK FOR ANALYTICAL RESEARCH AND MONITORING

***Rebecca S. Pugh,* Paul Becker, Steven J. Christopher, Clay Davis, Michael Ellisor, John Kucklick, Elizabeth Mackey, Barbara Porter, Heather Stapleton, Stacy Vander Pol, and Stephen A. Wise***

**National Institute of Standards and Technology, 331 Ft Johnson Road, Charleston, SC 29412, USA*

The Marine Environmental Specimen Bank (MESB) is maintained by the National Institute of Standards and Technology (NIST) as part of its National Biomonitoring Specimen Bank Program. Marine animal tissues are collected from several projects, including the Marine Mammal Health and Stranding Response Program (MMHSRP) and the Seabird Tissue Archival and Monitoring Project (STAMP). The goal of the MESB is to provide samples for future retrospective analyses for new analytes of interest; provide samples for future analyses using improved analytical techniques; and provide a resource of samples that have been collected and stored in a systematic and well-documented manner for comparing results over time to identify whether environmental trends exist. Some of the many uses of MESB samples are described, including quantifying organometallic compounds and newly emerging accumulative compounds, such as polybrominated diphenyl ethers, evaluating trophic transfer of persistent organic pollutants, determining effects of animal life history on concentrations of persistent organic pollutants, and determining trends in contaminant concentrations in seabirds, including polychlorinated biphenyls and mercury.

*Keywords:* biological samples, environmental analysis, sampling
*Application code:* environmental
*Methodology code:* sampling and sample preparation

### DETERMINATION OF ARSENIC IN PRESSURE-TREATED WOOD STRUCTURES AND CONTAMINATED ENVIRONS BY FLOW INJECTION PERVAPORATION AND INDIRECT SPECTROPHOTOMETRIC DETECTION

***Richmond J. Ampiah-Bonney* and Julian F. Tyson***

**Department of Chemistry, Lederle Graduate Research Center, University of Massachusetts Amherst, Room 701, MA 01003, USA*

Many wooden structures, especially those designed for outdoor use, are made from wood pressure treated with chromated copper arsenate (CCA), which leaches out into the immediate environs with time. To support research activities by middle school students in a major outreach program, methodology capable of detecting arsenic at realistic concentrations, without atomic spectrometry, was needed. Such a method has been developed for the determination of arsenic in the wood, and in the soil, plants and water in close proximity to the pressure-treated structure. The arsenic was extracted by various reagents and the filtered, acidified filtrate injected into a 0.3 M hydrochloric acid carrier and merged with a stream of sodium tetrahydroborate to generate arsine in the donor chamber of a pervaporation cell. The arsine gas is selectively transported through a 1.5 mm thick hydrophobic PTFE membrane into the acceptor chamber of the cell, where it dissolves in an acidified solution of $10^{-4}$ M potassium permanganate in 0.5 M $H_2SO_4$ and 0.002 M $KIO_3$ while the acceptor flow is stopped for 6.5 minutes. The reduction in absorbance of the $KMnO_4$ solution at 528 nm is measured. The sampling frequency was 7 per hour. Arsenic (III) spikes were over 90% recovered. The method gave a linear response over the concentration range 0–100 $ngmL^{-1}$ with a detection limit of 0.15 $ngmL^{-1}$. The advantages over the conventional molybdenum-blue procedure are that fewer reagents are used and heating is not necessary. In addition, the hydrophobic pervaporation membrane transfers only arsine and not water vapor.

*Keywords:* education, environmental, flow injection analysis, UV-VIS absorbance/luminescence
*Application code:* environmental
*Methodology code:* UV/VIS

### DEVELOPMENT OF A FLUOROMETRIC METHOD TO MONITOR AGING OF TRANSFORMER OIL

***Subbiah Deepa,* Ashok M. Mishra, and R. Sarathi***

**Department of Chemistry, Indian Institute of Technology, Madras, Chennai, Tamil Nadu 600 036, India*

An insulation system of a power transformer consists mostly of hydrocarbon oil and paper. Transformer oil contains high proportions of polychlorinated biphenyls (PCB) and polycyclic aromatic hydrocarbons (PAHs). However, oil and the insulating material suffer continuous deterioration and degradation due to the sustained application of the electric and cyclic thermal stresses. A standard method of analysis of PAHs like GC-MS involving lengthy extraction procedures is time consuming and expensive. Since PAHs are generally fluorescent, spectrofluorometry is widely used in their characterization in environmental samples. In application to complex natural systems, the selectivity of conventional fluorescence technique appears to be insufficient. Instead, multidimensional fluorescence techniques like synchronous fluorescence spectroscopy (SFS) and total luminescence spectroscopy (TLS) have been employed. Oil was subjected to accelerated thermal degradation under laboratory conditions. Insulating material (kraft paper and polypropylene) were impregnated in the oil maintained at 100° C kept in an air-circulated oven for 30 days. Fluorescence was monitored every 2 days. For SFS, from an optimization study 5 nm was chosen. A prominent SFS band at 335 nm was found to decrease substantially with aging of transformer oil. Changes of this band with regard to the presence of the insulating material could be rationalized in terms of oil aging. In the EEMF spectra, it was observed that there was a longer wavelength shift of (ex, em) with a progressive decrease in fluorescence intensity. This shift of SFS band and the contour maximum could be explained due to the oxidation of the conjugated double bonds and the aromatic species present in oil-form hydroperoxides, which in turn oxidizes to aldehydes and ketones. The aging process in the transformer oil is delayed in presence of polypropylene and paper insulation.

Evidence of carbonyl compound formation on aging was supported by Fourier transform infrared spectroscopy. A vibrational band corresponding to carbonyl stretching frequency appeared with aging of the oil. This is in agreement with the shift of SFS and EEMF contour maximum due to the formation of a new specie on aging. Thus, this study indicates the possibility of using fluorometry for in-situ online monitoring of the aging of transformer oil.

*Keywords:* fluorescence, PAH
*Application code:* environmental
*Methodology code:* fluorescence/luminescence

### SIMULTANEOUS DETERMINATION OF INORGANIC ANIONS AND ORGANIC ACIDS IN REFINERY PROCESS AND WASTE WATERS BY CAPILLARY ELECTROPHORESIS WITH INDIRECT UV DETECTION

***Nicolas Bord,* Carole Bailly, Gérard Cretier, Jean-Louis Rocca, and Jean-Paul Souchez***

**Laboratoire Des Sciences Analytiques Equipe Séparation, CNRS UMR 5180, Université Claude Bernard Lyon 1, Villeurbanne Cedex 69622, France*

A new application of capillary electrophoresis (CE) was developed for the simultaneous determination of inorganic anions such as bromide, chloride, thiosulfate, nitrite, nitrate, sulfate, thiocyanate, fluoride, phosphate, and organic acids such as oxalic, malonic, formic, tartaric, acetic, glycolic, propionic, butyric, and cyclohexanoic acids. Indeed, for the separation of the totality of these compounds in a single run, ion-exchange chromatography showed some limits in terms of selectivity. Trimellitic acid was used as the probe to allow for indirect UV detection. The background electrolyte was constituted of 10 mM trimellitic acid, 200 mM TRIS, and 0.1% polyvinyl alcohol (PVA) at pH 9.0. Because of adsorption phenomena of some compounds to the capillary wall, a PVA-coated capillary was employed for the separation. Baseline separation of the eighteen compounds was achieved in 9 minutes with indirect detection at 240 nm. Comigration problem for acetic and glycolic acids could be solved using 0.1% PVA as BGE additive.

The quantification was performed using internal standardization, by which molybdate was used as an internal standard. The results indicate excellent repeatability for relative migration times (RSD, 0.02%–0.35%, $n = 25$) that allows for a very powerful identification of peaks. The method is simple and rapid and can be applied to the analysis of refinery process and wastewaters. Under optimized conditions and using corrected peak area ratios for quantification, analytical performances including linear dynamic range, limits of detection, limits of quantification and recoveries met the need of the method. An acceptable correlation was observed between results obtained by the CE method and the ion-exchange chromatography method in real samples.

### ACKNOWLEDGEMENT

Financial support from the Total France Group is gratefully acknowledged.

*Keywords:* capillary electrophoresis, environmental/water
*Application code:* environmental
*Methodology code:* capillary electrophoresis

### RAPID ANALYSIS OF SOIL SAMPLES FOR ORGANIC ENVIRONMENTAL POLLUTANTS USING GAS CHROMATOGRAPHY TIME-OF-FLIGHT MASS SPECTROMETRY

***Lawrence D. Clark***

*Chromatographic Analysis Division, US Army Center for Health Promotion and Prevention, Building E-2100, 5158 Blackhawk Road, Aberdeen Proving Ground, MD 21010, USA*

Traditionally it has been necessary to analyze for multiple classes of environmental organic compounds in soils using several column and detector combinations. Our laboratory developed a method to quickly screen soil samples for semivolatiles, pesticides, and herbicides to support our deployment-oriented mission. With the developed method, over 180 organic pollutants in soil are analyzed in a single chromatographic run using the power of time-of-flight mass spectrometry and its associated spectral deconvolution software. The extraction is performed using SW-846 Method 3545. A 30-gram soil sample is extracted using this elevated-temperature, pressurized fluid extraction. Extracts are then concentrated to 10 mL for analysis. The extracts are analyzed using GC/MSTOF in a 15-minute analytical run. A significant reduction in both analytical and interpretation time is gained over the multiple column/detector combinations normally used. This reduction in turnaround time facilitates a quick-response, deployment mission where decision-makers need analytical results to perform environmental assessments.

*Keywords:* accelerated solvent extraction, environmental/soils, gas chromatography/mass spectrometry, time-of-flight MS
*Application code:* environmental
*Methodology code:* gas chromatography/mass spectrometry

### MONITORING OF INSECTICIDE AND HEAVY METAL LEVELS IN SEDIMENT CORES FROM LAKE ABAYA/SOUTH ETHIOPIA

***Bernd W. Wenclawiak,* Ulrike Koch, Thorsten Schmeck, and Brigitta Schuett***

**University of Siegen, Adolf Reichwein Street, Siegen 57068, Germany*

Lake Abaya is a part of the Lake Abaya-Lake Chamo system, a graben fill in the southern section of the Main Ethiopian Rift Valley. Drainage of Lake Abaya-Lake Chamo system covers a watershed of approximately 18 600 km$^2$. The area of Lake Abaya itself is about 80 km in length and 20–40 km in width and shows a maximum depth of 26 m (S. B. Awulachew, Dissertation, Technische Universität Dresden, 2001). Lake Abaya is surrounded mainly by farmland growing cotton, tobacco, bananas, and cereals,and by a couple of settlements in which the city Arba Minch with approximately 40 000 inhabitants is the largest. The aim of our study was to determine the amount of insecticides and heavy metals stored at different depths in the sediment of Lake Abaya. Sediment cores were sampled from river estuaries and the lake center. For monitoring purposes of many samples we developed measurement methods using GC/MS (T. Schmeck and B. W. Wenclawiak, *In: 1st Ethiopia Proceedings,* Sedimentary Studies in Tropics and Subtropics, Book of Abstracts, pp. 60–67, Siegen, Dresden, 2002/2003) and ICP-OES (D. Florian, R. M. Barnes, G. Knapp, J. Fresenius, *Anal. Chem.* vol. 362, pp. 558–565, 1998). Solvent extraction methods and acid digestion techniques were optimized. p,p-DDT and p,p-DDE are found as contaminants. Lead, chromium, nickel, copper, cobalt and zinc are on a geogenic background level. Depth profiles of the contaminants and metals were measured and showed significant variations. Also significant lateral variations were found comparing the results from different estuaries and the lake center.

### ACKNOWLEDGEMENT

This project is supported by the Deutsche Forschungsgemeinschaft under WE 1073/17-1.

*Keywords:* environmental analysis, GC-MS, plasma emission (ICP/MIP/DCP/etc), sample preparation
*Application code:* environmental
*Methodology code:* sampling and sample preparation

### ANALYTICAL SCREENING ACCORDING TO ROHS AND ELV DIRECTIVES USING BENCHTOP EDXRF

***Dirk Wissmann** and Oliver Buettel***

**Spectro Analytical Instruments GmbH & Co KG, Boschstr 10, Kleve 47533, Germany*

One of the actual challenges of industrial elemental analysis is the analysis of hazardous substances in electric and electronic equipment. Using EDXRF as an analytical method allows for a fast screening with small sample preparation. The purpose of this screening should be to monitor the threshold values safely. Benchtop EDXRF with excitation using a 50 kV X-ray tube and polarized radiation allows for the analysis of Cd in electronic products with the required sensitivity. By means of secondary targets the required sensitivity for the concerned elements Cr, Hg, Br, and Pb is easily achieved as well. For the analysis of hexavalent chromium, the polybrominated biphenyl (PBB), and polybrominated diphenyl ethers (PBDE), only the complete content of Cr or Br, respectively, can be monitored. Analyzing the overall content of Cr and Br is an accepted alternative to specific determination of these compounds as long as the threshold value is not exceeded. XRF in general is the preferred analytical technique for screening of these samples. With its ease of use and simple sample preparation it fulfills important requirements for routine analysis. The paper describes the background of the legislation (RoHS, ELV) and the impact on the analytical technique. It also gives some information on comparison studies, which had been performed at the Europa Fachhochschule Fresenius.

*Keywords:* elemental analysis, X-ray fluorescence
*Application code:* environmental
*Methodology code:* X-ray techniques

### ADVANTAGE AND DISADVANTAGE USING DISCRETE VERSUS FIA/SFA TECHNOLOGY IN AUTOMATED MONITORING OF IONS IN WATER

***Ninglan Liao,** Michael L. Duffy, William Lipps, and Craig Marvin***

**OI Analytical, PO Box 9010, College Station, TX 77845, USA*

The use of flow injection analysis (FIA) or segmented flow analysis (SFA) to automate wet chemistry has been mainstream in environmental laboratories since 1970s due to its flexible configuration, lower detection limit, and capability for in-line sample pretreatment. Discrete analyzers were successfully introduced into the environmental marketplace to automate wet chemistry approximately 4 years ago. Just as discrete analyzers had replaced FIA and SFA systems in the clinical market over a decade ago; the same is beginning to occur for environmental applications. Discrete analyzers offer greater sample capacity per hour, are significantly easier to use, and significantly lower reagent consumption, a key point with increasing costs of waste disposal for labs. Both technologies are currently accepted in environmental laboratories even though discrete methods are still in the process of obtaining full methods approval by the various regulatory agencies. Each technology has its own advantages and disadvantages. We will compare in detail the advantages and disadvantages of each technology and present data performance comparisons for discrete versus FIA/SFA analyzers. In addition we will discuss new trends in automated wet chemistry and describe how to select the right technology for many individual applications.

*Keywords:* automation, environmental analysis, spectrophotometry, wet chemical methods
*Application code:* environmental
*Methodology code:* chemical methods

### USING A DISCRETE ANALYZER FOR NITRATE DETERMINATION BY CADMIUM REDUCTION

***Angela D. Serra***

*STL, Inc, 4101 Shuffel Drive NW, North Canton, OH 44720, USA*

The use of discrete autoanalyzers has increased dramatically in recent years as a new technology that provides laboratories with a means of reducing labor and waste disposal costs while increasing productivity. A discrete autoanalyzer has been used to determine nitrate concentration in aqueous samples through cadmium reduction. Nitrate is reduced to nitrite in the presence of cadmium. The nitrite is determined by diazotizing with sulfanilamide and coupling with N-(1-naphthyl)-ethylenediamine dihydrochloride to form a highly colored azo dye that is measured colorimetrically. A correction may be made for any nitrite present in the sample by analyzing without the reduction step. The initial reduction efficiency of the cadmium column was evaluated along with the column's expected longevity. Residual chlorine interferes by oxidizing the cadmium column, thereby reducing column efficiency. Column degradation due to such sample interference was evaluated. Common method performance measures such as percentage recovery, percent RSD, and method detection limits will also be presented.

*Keywords:* process analytical chemistry
*Application code:* environmental
*Methodology code:* physical measurements

### CHALLENGE TO LOWER DETECTION LIMIT BY DISCRETE ANALYZER TO MEET EPA REGULATED REQUIREMENTS FOR AUTOMATED WET CHEMISTRY IN NUTRIENT ANALYSIS

***William Lipps*[*] *and Ninglan Liao***

[*]*OI Analytical, PO Box 9010, College Station, TX 77842, USA*

Automating wet chemistry with discrete analyzers is a new technology for environmental testing laboratories. Analytical methods that are adapted from existing approved methodology are slowly gaining acceptance by regulatory agencies. Meeting regulatory accuracy and precision requirements and achieving low detection limits are a challenge to existing discrete technology. We will discuss EPA methods and their requirements, present detailed data performance using a new OI Analytical discrete analyzer for nutrient analysis, and describe the strengths and weaknesses for obtaining lower method detection limits by using discrete chemistry analyzers.

*Keywords:* automation, environmental analysis, spectrophotometry, wet chemical methods
*Application code:* environmental
*Methodology code:* chemical methods

### ULTRAFAST GC ANALYSIS OF ESSENTIAL OILS AND PETROLEUM FRACTIONS USING AUTOMATIC NANOVOLUMES INJECTION

***Thomas Porzano,*[*] *Flavio Bedini, Andrea Cadoppi, Riccardo Facchetti, and Fausto Pigozzo***

[*]*Thermo Electron Corporation, Strada Rivoltana Km 4, Rodano, Milano 20090, Italy*

The combination of nanovolume sampling with ultrafast separation capabilities is particularly attractive, in capillary gas chromatography, for those application fields where information over very concentrated or pure samples is required in very short times. The control over such small volume injections permits to avoid dilution with solvent still preventing the main constituents from exceeding even the low capacities of the short narrow bore columns used in ultrafast GC. Ordinary GC syringes of 5–10 $\mu$L are found to be not suitable for sample volumes smaller than 0.1 $\mu$L due to insufficient volume accuracy, whereas the use of special plunger-in-needle syringes allows for an accurate measurement of volumes 10 times smaller.

In this work these syringes are routinely used on an autosampler requiring no modification. Sample introduction through a hot inlet is performed exploiting the liquid band formation technique (cold needle). This combination allows to overcome the problems related to sample evaporation from the needle. Volumes as low as 10–20 nL were injected with good accuracy and precision. Ultrafast GC analysis showed perfect peak shape, with peak widths around 200 milliseconds and no broadening due to column overloading. Excellent linearity versus injection volume and split ratio, as well as high-quality repeatability and sample recovery were obtained.

Application to the analysis of pure essential oils and petroleum fractions are illustrated. Comparison of the results with those obtained with conventional 1 $\mu$L injection of the diluted samples shows a perfect agreement.

*Keywords:* capillary GC, flavor/essential oil, petroleum, sample introduction
*Application code:* food science
*Methodology code:* gas chromatography

### FAST SEPARATIONS WITH STANDARD CAPILLARY COLUMNS USING ACCESSORY HEATERS IN CONVENTIONAL GC OVENS

***Stephen J. MacDonald***

*Zip Sceintific, 1 Gates Lane, Hudson, NH 03051, USA*

Many techniques including fast temperature programing, short/microbore columns, and pressure programing have been used to improve gas chromatographic throughput. Fast temperature programing can be achieved with specialized low-thermal mass systems that replace the conventional GC oven with resistively heated column housings that can ramp up to 1200°C/min; however, such rates are applicable to a small percentage of GC applications. Another approach is to use an accessory heater placed inside the GC oven that operates under complete control of the host GC microprocessor. Fast practical temperature programing rates up to 120°C/min can be achieved to reduce run times by factors of 2–5. Accessory heaters do not require specialized columns and heated interfaces and can be used for single or dual column applications. Fast temperature programing with accessory heaters has been demonstrated to be a precise and reliable technique for demanding test lab applications that run 24/7 throughout the year. Furthermore, simple design and low cost make accessory oven heaters an attractive means of reducing run times and achieving high sample throughput. This presentation will review the design and implementation of accessory heaters for conventional GC ovens and present data showing fast GC separations for routine applications in petrochemical, environmental, and other fields of interest.

*Keywords:* environmental analysis, gas chromatography, gas chromatography/mass spectrometry, instrumentation
*Application code:* high-throughput chemical analysis
*Methodology code:* gas chromatography

### FAST GAS CHROMATOGRAPHY, GUIDELINES AND APPLICATIONS

***Leonard M. Sidisky,* Greg L. Baney, Jamie Desorcie, and Katherine L. Stenerson***

**Supelco Inc, 595 North Harrison Road, Bellefonte, PA 16823, USA*

The current trend in GC analysis has been towards decreasing analysis time. This must be achieved while still maintaining the resolution necessary to adequately perform the analysis. This presentation covers the theoretical and practical aspects of fast gas chromatography. The specifics of fast GC will be defined, including several different instrumentation options. The implications of modifying specific method parameters to decrease analysis time will also be discussed. Finally, relevant applications in the environmental, petrochemical, and food and beverage fields will be presented.

*Keywords:* capillary GC, environmental, flavor/essential oil, petroleum
*Application code:* general interest
*Methodology code:* gas chromatography

### OPTIMIZATION OF THE EFFECTIVE SEPARATIONS FOR PEPTIDES AND PROTEINS USING HIGHLY DURABLE PACKING MATERIALS FOR HIGH-PERFORMANCE LIQUID CHROMATOGRAPHY

***Naohiro Kuriyama,* Masako Moriyama, Masakatsu Omote, and Noriko Shoji***

**YMC Co, Ltd, 5-28, Kokufudai, Komatsu, Ishikawa 923-8557, Japan*

Reverse-phase high-performance liquid chromatography is an effective tool for the analytical and preparative separation of peptides and proteins, due to the high-resolution separation. Recently the large-scale purification of peptides or proteins using RPHPLC has been carried out in the industrial production. Most of the separations are achieved by using gradient elution with trifluoroacetic acid as an acidic mobile phase modifier. However, the bonded phase bleedings by acid-catalyzed hydrolysis occurs under such acidic conditions. In this study, we present the developing of highly durable packing materials under acidic conditions and compare them with commercially available packing materials. In addition, we describe the optimization of the separations for peptides and proteins concerning the differences for pore size of silica and bonded alkyl group. The novel packing materials with short alkyl chain group based on the wide pore size silica showed specific separation profiles of peptides or proteins and high durability under acidic conditions.

*Keywords:* HPLC columns, peptides, protein, proteomics
*Application code:* proteomics and genomics
*Methodology code:* liquid chromatography

### MOLECULAR IDENTIFICATION AT THE SINGLE-MOLECULE LEVEL FOR DISEASE DIAGNOSIS

***Edward S. Yeung,* Ji-Young Lee, and Hung-Wing Li***

**Department of Chemistry and US DOE Ames Laboratory, Iowa State University, Ames, IA 50011, USA*

The electrophoretic mobilities of individual DNA molecules can be determined at the rate of up to 2500 every 25 milliseconds by using fluorescence imaging. The average mobility agreed well with that obtained in capillary electrophoresis (CE). The signal-to-noise ratio (S/N) did not decrease in the presence of up to 8% plasma or 8% raw blood. Single-molecule detection was still possible in the presence of 50% raw blood. Single-molecule CE of two differently labeled molecules was carried out in the presence of a transmission grating. Even when the mobility difference is not sufficient because of low S/N, identification using different fluorescence wavelengths can be performed at more than 99% accuracy. So, when small differences in DNA sequence due to disease or mutation can lead to hybridization to labels with different dyes, the screening of the mutated DNA will be facilitated by on-line spectroscopy in addition to the electrophoretic information from CE.

*Keywords:* bioanalytical, electrophoresis, spectroscopy
*Application code:* clinical/toxicology
*Methodology code:* separation sciences

### MONITORING SYNAPTIC AND EXTRASYNAPTIC NEUROTRANSMISSION IN THE BRAIN

***Adrian C. Michael,* Laura M. Borland, and Bridget M. Willoughby***

**Department of Chemistry, University of Pittsburgh, Pittsburgh, PA 15260, USA*

The synapse represents a site of contact between neurons that brings the membrane of two cells into nanometer-scale proximity with one another. Efforts to monitor chemical transmission across these contacts have included the implantation of microelectrodes into living central nervous system tissues such that the electrode closely approaches the synapse. In the case of dopamine and glutamate, this approach has been highly productive in revealing new information about the interactions between these neurotransmitters in a specific region of the rodent brain called the striatum. However, these measurements have produced results to suggest that communication is not just limited to synaptic contacts but rather that some communication processes involve diffusion over longer distance ranges. This leads to questions as to how communication occurring on the nanometer and longer distance scales function, presumably in concert, to relay information in the central nervous system.

*Keywords:* biological samples, biosensors, microelectrode, voltammetry
*Application code:* neurochemistry
*Methodology code:* sensors

### DYNAMIC MONITORING OF LIVE CELLS IN A MICROFLUIDIC ENVIRONMENT

***Robert T. Kennedy***

*Department of Chemistry, University of Michigan, 930 N University, Ann Arbor, MI 48109-1055 USA*

Understanding the biochemical mechanism of cellular function requires the ability to monitor dynamic changes with minimal perturbation of the cell. We are exploring combination of microfluidic assays, nanoscale sensors, and imaging to study living cells.

As a model system we are studying stimulus secretion coupling in single islets of Langerhans. Islets are microorgans, containing about 3000 cells, found within the pancreas that secretes hormones, including insulin, to regulate metabolism. Secretion of insulin is controlled by an interaction of metabolism and intracellular Ca ions. We have developed a microfluidic chip that can house a single islet and monitor insulin secretion in real time using a sampling and electrophoresis-based immunoassay. We have developed sensors, including a nanoscale optical oxygen sensor to monitor metabolism in the islets. Finally, we have developed approaches to image secretion events at the islets. Using these tools we have characterized novel transgenic islets, biochemical oscillations that occur in the islets, and chemical synchrony among cells. These tools promise to be useful for a wide variety of systems.

*Keywords:* biomedical, biosensors, biotechnology, capillary electrophoresis
*Application code:* bioanalytical
*Methodology code:* microfluidics/lab on a chip

### PORTABLE FAST GC WITH PFPD FOR CHEMICAL WARFARE AGENT ANALYSIS AND ELECTROLYZER FID FOR BREATH ANALYSIS

***Aviv Amirav***

*School of Chemistry, Tel Aviv University, Tel Aviv 69978, Israel*

A pulsed flame photometric detector (PFPD), coupled with a short-column fast GC, was designed, constructed, and tested for low-level field detection and identification of chemical warfare agents (CWA). Airborne pollutants were adsorbed on a short trapping column, thermally desorbed and separated in time through a temperature-programed, short capillary column using resistively heated external heating tube. Hydrogen served as purge, carrier, and combustion gas with 4 mL/min average total flow rate and air was provided with a small pump from filtered environmental air. The PFPD separation is fully orthogonal to that of the GC; thus, false alarms are practically eliminated combined with agent identification on the molecular level for optimal decision-making and minimizing mask wearing time.

The same GC was also separately equipped with an electrolyzer FID (EFID) in place of the PFPD for its application in breath analysis for medical diagnostics. In this gas-cylinder-free GC, a small water electrolyzer produces premixed hydrogen and oxygen gas mixture, which serves consecutively as a purge-and-trap gas, column carrier gas, and as the FID combustion gas mixture. The EFID is based upon the combustion of a premixed, stochiometric, hydrogen and oxygen gas mixture, at a low flow rate, without the make-up gas and without gas separation or compression. The gas-cylinder-free GC-EFID was tested and evaluated in human breath analysis. One-minute noninvasive point-of-care analysis of volatile organic compounds in breath will be demonstrated, with sub-ppb detection limits and with no gas maintenance.

*Keywords:* capillary GC, environmental air, GC detectors, instrumentation
*Application code:* homeland security/forensics
*Methodology code:* gas chromatography

### ON-SITE MEASUREMENTS AND IN-FIELD MONITORING USING GAS CHROMATOGRAPHS, ION MOBILITY SPECTROMETERS, AND COMBINATIONS OF THE SAME

***Gary A. Eiceman***

*Department of Chemistry and Biochemistry, New Mexico State University, Las Cruces, NM 88003, USA*

Mobility spectrometry has become a central measurement science and technology for military preparedness and commercial aviation security. The features of mobility spectrometers that have favored on-site or in-field use include portability, comparatively cost of ownership or operation, low detection limits for certain important chemicals, and fast response. These have been developed further with new generations of small analyzers including drift tubes that are made through microfabrication techniques. The analytical response is grounded in gas-phase ion chemistry at ambient pressure which establishes advantages of sensitivity and disadvantages, on occasions, from matrix interferences. Small or hand-held gas chromatographs can provide improved analytical integrity with some additional complexity and these will be described. Alternative solutions to chromatographic inlets will be illustrated with environmental monitoring studies with mobility spectrometers or differential mobility spectrometers.

*Keywords:* analysis, gas chromatography, volatile organic compounds
*Application code:* environmental
*Methodology code:* others

### IN-SITU SAMPLING/SAMPLE PREPARATION COUPLED TO PORTABLE GC FOR RAPID AIR INVESTIGATIONS

***Janusz B. Pawliszyn***

*Department of Chemistry, University of Waterloo, Waterloo, Canada ON N2L 3G1*

New technologies allowing for integration of sampling and sample preparation will be discussed. The focus will be placed on two approaches based on modified needles—needle traps (NT device) based on packed needles and coated fibers in the needle (SPME device). NT device is used to obtain the value for total concentration of analytes in air, while SPME device provides gas-dissolved fraction. To facilitate the use of these approaches for on-site implementation, a new field sampler was designed and tested. The sampler is versatile and convenient to deploy. It can be adopted for use with packed needles and coated fibers for both time-weighted average (TWA) and spot sampling. The SPME fiber can be positioned precisely inside the needle for time-weighted average (TWA) sampling, or exposed completely outside the needle for rapid sampling. The needle is protected within a shield at all times hereby eliminating the risk of operator injury and fiber/needle damage. A replaceable Teflon cap is used to seal the needle to preserve sample integrity. Factors that affect the preservation of sample integrity (sorbent efficiency, temperature, and sealing materials) are studied. The use of a highly efficient sorbent is recommended as the first choice for the preservation of sample integrity. Teflon was a good material for sealing the fiber needle, had little memory effect, and could be used repeatedly. To address adsorption of high boiling point compounds on fiber needles, several kinds of deactivated needles were evaluated. On-site investigations demonstrate validity of the new device for field applications.

*Keywords:* aerosols/particulates, on-line, SPME, volatile organic compounds
*Application code:* environmental
*Methodology code:* sampling and sample preparation

### FAST AND SENSITIVE ENVIRONMENTAL ANALYSIS UTILIZING MICROEXTRACTION IN PACKED SYRINGE ON LINE WITH GC-MS-MS

***Mohamed Abdel-Rehim,[*] Lars Blomberg, and Aziza Elbeqqali***

*[*]Department of DMPK & BAC, AstraZeneca R&D, Södertälje 15185, Sweden*

A new technique for sample preparation on line with LC and GS-MS assays was developed. Microextraction in a packed syringe (MEPS) is a new miniaturized, solid-phase extraction technique that can be connected on line to GC or LC without any modifications.

In MEPS approximately 1 mg of the solid packing material is inserted into a syringe (100–250 mL) as a plug. Sample preparation takes place on the packed bed. The bed can be coated to provide selective and suitable sampling conditions. The new method is very promising. It is very easy to use, fully automated, of low cost, and rapid in comparison to previously used methods. This paper presents the development and validation of a method for MEPS on line with GC-MS to determine naphthalene, fluorene, anthracene, fluoranthene, pyrene, chrysene and benZo (a) pyrene in water. Due to their widespread use over the last 100 years, PAH can be found in polluted waters and sediments. Some polyaromatic hydrocarbons have turned out to show mutagenic and carcinogenic effects. The application of a new, rapid analysis method, MEPS on line with GC/MS, is demonstrated for these analytes.

MEPS is as accurate as SBR and SPME, the same for the precision; the limit of detection (1–5 ng/L) is far better than the SPME method (40 ng/L). It took only 3 minutes for the extraction compared to 3–5 hours in the SBR case, and 15–50 minutes in the SPME.

*Keywords:* environmental analysis
*Application code:* environmental
*Methodology code:* sampling and sample preparation

### ON-LINE MONITORING OF GLASS MELTER SLURRY USING LASER-INDUCED BREAKDOWN SPECTROSCOPY

***Lazarus Solomon,[*] John W. Branch, Tracey Miller, Jagdish P. Singh, Fang-Yu Yueh, and Hongbo Zheng***

*[*]Mississippi State University, 205 Research Boulevard, Starkville, MS 39759, USA*

Laser-induced breakdown spectroscopy (LIBS) is a laser-based diagnostic tool used in the study of atomic emission from various solid, liquid, and gas samples. It is a sensitive optical technique with high resolution. LIBS utilizes high-intensity laser to generate luminous microplasma from different types of targets to facilitate the study of the targets' optical emission properties. It is regarded as a more versatile useful means of monitoring the chemical composition of process flow material, both on line and in real time with minimal sample preparation, even under hostile environmental conditions. Though slurry application is unique and very challenging when compared to solids, it is not altogether impossible. The problems encountered with the slurry were loss of focus due to splattering of target material, attenuation of emission signal due to splattering, and surface cavitations. Appropriate steps were taken to overcome these problems by periodically monitoring the focus of the beam, sending a stream of air over the bottle opening to clear the emission path of splattered particles, and rotating the sample to provide new surface for ablation, respectively. A Q-switched Nd: YAG laser was used in the study of the above material. System parameters such as gate width, gate delay, laser energy, diameter of the opening of the bottle containing the material, and the focus point of the beam were optimized to achieve the best signal-noise ratio. By fine-tuning the above parameters, a significant improved signal-to-noise ratio was obtained. Detailed studies of the results will be presented in the conference.

*Keywords:* elemental analysis, laser, plasma, spectroscopy
*Application code:* environmental
*Methodology code:* atomic spectroscopy/elemental analysis

### A SIMPLE METHOD FOR SELECTIVELY MONITORING THE CONCENTRATIONS OF TOTAL TRIHALOMETHANES AND TOTAL HALOACETIC CONCENTRATIONS IN DRINKING WATER

***Gija Geme,* Michael A. Brown, Gary L. Emmert, Kyoo Dong Jo, and Paul S. Simone***

**Department of Chemistry, The University of Memphis, Smith Chemistry Building, Room 213, Memphis, TN 38152, USA*

Water chlorination is the most widely used drinking water disinfection process in the United States. Unfortunately, it produces two classes of halogenated disinfection by-products: the trihalomethanes (THMs) and the haloacetic acids (HAAs). A relatively simple method using capillary membrane sampler (CMS) flow injection analysis (FIA) has been developed for selectively measuring the concentrations of total THMs and total HAAs in drinking water. The method is based on a modified Fujiwara chemistry where the THM or the HAA species react with nicotinamide in basic solution to form a fluorescent product. Two configurations of CMS were used, one selective for total THMs and another selective for total HAAs. The CMS device was constructed in the laboratory with commercially available materials. The preliminary results show a linear working range from 10 ppb to 100 ppb for THMs and from 10 ppb to 70 ppb for HAAs. The method detection limit (MDL) for total THMs is 4.1 ppb and for total HAAs 2.1 ppb. The mean percent recovery for THMs is 106 percent and for HAAs 121 percent. The relative percentage standard deviation for THMs and HAAs is 8.1 and 3.6 percent, respectively. The total analysis time is about 25 minutes per sample. The effects of ionic strength changes and water temperature of solution on the analytical signal have been investigated. Interference, selectivity, and linearity studies were performed. Drinking water samples were analyzed by each proposed method and the results were compared to United States Environmental Protection Agency (USEPA) Method 502.2 for THMs and to USEPA Method 552.3 for HAAs. In the study, water samples were collected from locations in Arkansas, Louisiana, Maryland, Wisconsin, and seven different locations in Tennessee. Student *t* test was used to evaluate if the results from the methods were statistically different at the 95% confidence level. The results of this statistical comparison indicated that these methods did not give statistically different results. In addition to the statistical evaluation of the data, a practical evaluation of the data will be presented.

*Keywords:* environmental/water, flow injection analysis, fluorescence, water
*Application code:* environmental
*Methodology code:* fluorescence/luminescence

### A SIMPLE, INEXPENSIVE LED SPECTROMETER FOR MONITORING DISINFECTANTS IN DRINKING WATER

***Lucy J. Thurston,* James J. Aschberger, and Gary L. Emmert***

**Department of Chemistry Smith Chemistry Building., University of Memphis, Room 213, Memphis, TN 38152, USA*

One way to increase the sensitivity of analytical measurements of disinfectant concentrations is to take advantage of their bleaching capabilities when reacted with colored organic dyes. The chemical basis of using these dyes as disinfectant reagents is that the disinfectant will react to decolorize the dye. By monitoring the decrease in the absorbance of the dye as it is bleached by the disinfectant (more appropriately decolorized), the analytical signal is enhanced because the molar absorptivity of the dye is much greater than the molar absorptivity of the disinfectant. The decrease in the absorbance of the dye at the wavelength of maximum absorbance is proportional to the disinfectant concentration. A simple LED spectrometer was adapted from a design by McClain (University of Wisconsin-Madison) and used in conjunction with an inexpensive flow cell prepared from PDMS, two ports, and a microscope slide. Using this system, a simple miniaturized analyzer was constructed to measure the concentration of disinfectants in drinking water. The LED spectrophotometer was constructed for under $50. By simply switching the LED, different dyes can be investigated at different wavelengths, thus demonstrating the versatility of the instrument. The system is relatively simple being comprised of a syringe pump, a six-port sample injection valve, a superserpentine mixing coil, and a laboratory-constructed LED spectrophotometer with flow cell. The results of preliminary studies using congo red gave MDL estimates of 0.7 mg/L. Percentage mean recoveries, a measure of method accuracy, averaged 107%. The relative percentage standard deviation

(RSD%), a measure of precision, averaged 15%. The use of amaranth, bromothymol blue, and thymol blue as disinfectant reagents will be investigated. A preliminary monitoring study of chlorine in Memphis drinking water will be presented.

*Keywords:* environmental/water, spectrometer, UV-VIS absorbance/luminescence
*Application code:* environmental
*Methodology code:* UV/VIS

### BOOST PRODUCTIVITY AND LOWER ANALYTICAL COSTS WITH TOC/NITROGEN MONITORING BY HIGH-TEMPERATURE COMBUSTION

***Brian T. Wallace***

*Teledyne Instruments - Tekmar, 4736 Socialville-Fosters Road, Mason, OH 45040, USA*

Nitrogen monitoring is an important element of process control for treatment of wastewater, seawater, and a variety of industrial process water applications. Failure to control nitrogen effluent can cost thousands of dollars and take days to recover. Currently, total Kjeldahl nitrogen (TKN) is the standard method for organic nitrogen analysis and nitrate/nitrite analysis is used for detecting inorganic forms of nitrogen. Drawbacks of these methods include that they are time-consuming (two to three hours analysis time), environmentally unfriendly, and labor-intensive tests for laboratory personnel to perform. Fortunately, new advances in high-temperature combustion (HTC) technology with chemiluminescence detection provide a quick and easy way to monitor nitrogen loading by total nitrogen (TN) analysis. Since this analysis can be performed simultaneously with traditional total organic carbon (TOC) analysis, the analytical benefits can be achieved with minimal labor and capital expenditure, boosting productivity and lowering costs over existing nitrogen analysis. Because the high-temperature combustion (HTC) technology uses a minimal amount of sample without the addition of harsh reagents, the waste from the analysis can be disposed of with nominal treatment.

*Keywords:* chemiluminescence, elemental analysis, environmental/water, total organic carbon
*Application code:* environmental
*Methodology code:* others

### A FULLY AUTOMATED MEMBRANE SAMPLING METHOD FOR MONITORING TRIHALOMETHANES IN DRINKING WATER DISTRIBUTION SYSTEMS

***Michael A. Brown*[*] and Gary L. Emmert***

[*]*Department of Chemistry, The University of Memphis, Smith Chemistry Building, Room 213, Memphis, TN 38152, USA*

Trihalomethanes (THMs) are disinfection by-products (DBPs) that are formed during the water chlorination process and are considered to be possible carcinogens. The USEPA currently regulates four THMs which include chloroform, bromodichloromethane, dibromochloromethane, and bromoform (THM4). Currently, the maximum contaminant level for THM4 in drinking water is 0.080 mg/L. Typical THM methods, even though quite successful, often involve cumbersome sample preparation steps or require expensive equipment to perform and are also difficult to adapt to on-line monitoring. This research aims to develop a fully automated on-line supported capillary membrane sampling-gas chromatograph with an electron capture detector (SCMS-GC-ECD). The SCMS-GC-ECD analyzer can be used to establish a method that will eliminate most of the disadvantages associated with conventional methods. Extraction of THM4 present in a drinking water sample occurs by permeation of the molecules through silicone tubing used with the SCMS. These compounds are then transported to a 10-port electrically actuated injection valve with N2 and continuously load a sample loop. By changing the valve's position, GC carrier gas transports the contents of the loop to a capillary column to be separated and detected by an ECD. A simple device for on-line injection of internal standard(s) is being developed to correct for changes that may occur with the analyzer or within the water matrix.

A final optimized version of the analyzer was connected to an ordinary water tap to conduct a preliminary week long monitoring study for Memphis water. Results from the SCMS-GC-ECD were compared side by side to results obtained by performing USEPA Method 502.2, which uses purge-and-trap gas chromatography with electrolytic conductivity detection (HALL). Experimental set-up, instrumentation details, and optimization studies will be discussed. MDL, accuracy, and precision studies for both sampling methods will also be presented. Practical issues with on-line monitoring using this method will also be discussed.

*Keywords:* environmental/water, gas chromatography, sample introduction, volatile organic compounds
*Application code:* environmental
*Methodology code:* gas chromatography

### NITROGEN AND CARBON DETERMINATION IN WATER BY ELEMENTAL ANALYSIS WITH AUTOMATIC LIQUID INJECTION

***Liliana Krotz,*[*] *Guido Giazzi, Richard Hancock, and Luigi Ragaglia***

[*]*Thermo Electron Corporation, Strada Rivoltana, Rodano, Milano 20090, Italy*

The determination of total nitrogen (TN), total carbon (TC), and total organic carbon (TOC), having a simple and fully automatic system, provides useful information on the characterization of water (waste, sewage, river, lake, and sea), so that the possible contamination source can be easily located and also used to monitor the treatment of wastewater.

In this way the FlashEA 1112 elemental analyzer, based on the dynamic combustion of the sample, provides rapid and automatic nitrogen and carbon determination as well as advantages in terms of accuracy, reproducibility, and low cost per analysis.

Samples are directly injected by means of a syringe via the AS3000 autosampler. A report is automatically generated by the Eager 300 dedicated software and displayed at the end of the analysis. This paper presents data of water samples analyzed several times to show the accuracy and precision for the extremely low concentration of nitrogen and carbon present. Differentiation of TOC from TC was performed by sample pretreatment. For TC the sample is analyzed as it is while for TOC the water sample is acidified with chlorhydric acid to eliminate carbonates, and then analyzed.

*Keywords:* elemental analysis, environmental/water, sample handling/automation, water
*Application code:* environmental
*Methodology code:* atomic spectroscopy/elemental analysis

### EXPERIENCE OF AN AUTOMATIC ON-LINE MEASUREMENT SYSTEM MONITORING PPB-LEVEL VOLATILES IN WATER OVER THE LAST 10 YEARS

***Ulrich K. Gokeler***

*Siemens, 7101 Holister Road, Houston, TX 77040, USA*

To continuously monitor surface water quality taken from a ship channel, an automatic analytical monitoring station was installed more than 10 years ago. The water from the channel is the primary fresh water supply to a major city and is continuously frequented by large vessels with unknown cargo. The monitoring stations purpose is to provide early warning to the water distribution facility downstreams in case of a marine accident and pollution.

The unattended measuring system is automatically monitoring more than 40 different hydrocarbon and halogenated compounds selectively and is interference free at the ppb and ppt levels. Because of frequent rain water run-off from the surrounding hills and increased mud concentration from the channel bottom whenever a vessel passes by, specific attention to the design and automatic cleanup of the filtering and extraction system is necessary. An on-line spin-cleaning filter design with pressure differential measurement automatically triggering cleaning cycle is utilized. To reach the low detection levels and to extract the targeted components from the channel water, a continuous sparging extraction system with self-cleaning capabilities is used. The measurement system consists of isothermal on-line process gas chromatographs equipped with capillary columns, simple and maintenance free column switching, and FID and ECD detectors for the various hydrocarbon and halogenated compounds. Due to the location of the monitoring station and in order to ensure the necessary gas quality, continuous gas utility supply is provided by utilizing gas generators for hydrogen, nitrogen, and combustion air. Automatically, every 30 minutes an analysis cycle is completed, results are determined, and data are collected, stored, and used to generate alarms when exceeding preset concentration thresholds.

This presentation discusses the system and analytical configuration of the measurement station and provides a summary of the analytical performance since installation includes necessary maintenance and failures encountered.

*Keywords:* environmental/water, gas chromatography, purge and trap, water
*Application code:* environmental
*Methodology code:* gas chromatography

### NEAR-LINE AUTOMATED ANALYSIS OF WATER QUALITY PARAMETERS: STUDY COMPLETED BY ENVIRONMENTAL PROTECTION AGENCY EQUIPMENT VERIFICATION PROGRAM

***Robert V. Menegotto* and Tiffany Reid***

**Man-Tech Associates Inc, 600 Main Street, Tonawanda, NY 14150, USA*

As part of homeland security, drinking water safety is an important area of study. Technologies that will detect contamination in a water supply quickly and accurately are of particular interest. Minimizing false alarms is critical. Quick testing is important for effective response in order to protect the safety of citizens; however, accurate results are also essential. In this respect, lab-based instruments have proven to be accurate and precise but require transportation of the sample to the laboratory, increasing potential for contamination. The development of a lab-based instrument that can automatically sample at set time intervals and measure key water quality parameters was developed. Two of these systems were installed at the Environmental Protection Agency's Test Facility in Cincinnati. The test site included a water loop with water taken from the city of cincinnati water supply. The system automatically analyzes pH, conductivity, temperature, alkalinity, residual chlorine, and turbidity. The study consisted of three parts: accuracy of results as compared to lab analysis comparisons, the ability to detect spikes from injection of contaminants into the water supply, and precision of results from two identical instruments. A description of the system as well as results from this study will be presented.

*Keywords:* environmental analysis, environmental/water, monitoring, titration
*Application code:* environmental
*Methodology code:* others

### MONITORING THE EFFECTS OF EDIBLE OIL HYDROGENATION IN A LAB-SCALE HIGH-PRESSURE REACTOR

***Gerald J. DeMenna* and Nancy Gregorio***

**Sacred Heart University, 345 West 58th Street Suite-2K, New York, NY 10019, USA*

Butter ... OKAY! Margarine ... NO WAY! How about ... PARKAY? Do we know what "hydrogenation" is? What comprises an "HVO"? Does it make a difference what "vegetable oil" is used? What happens to convert it to shortening or margarine? Are catalysts used? What is their reaction mechanism? Are "synthetic" saturated fatty acids better or worse than "natural" saturated fatty acids? Is it okay to eat "food" made with it? Let us leave the last two questions to politicians, lobbyists, and health experts, but science can answer the first five! Natural oils and fats ("liquid" are oils and "solid" are fats) are made of triglycerides consisting of glycerol and three fatty acids. While the glycerol is common to all oils, it is the wide variety of the different fatty acid parts that determines the wide variety of characteristics of the oil: melting point, smoke point, oxidation affinity, rancidity, color, saturation, and so on. Most plant-based products tend to be liquid oils with predominantly mono-unsaturated (oleic acid of olive oil) and poly-unsaturated (linoleic and linolenic acids of soy and canola oil), with lower smoke points and shorter stability than the solid fats (palmitic and myristic acids derived from coconut and palm oils). The soybean industry created a virtual "buffalo" where nothing went to waste! If you made too much liquid oil, you could change it into a solid fat with a little chemical magic!

This presentation examines the hydrogenation protocols used in the industry to produce "HVOs," and by duplicating these processes in a high-pressure, controlled laboratory reactor, we can evaluate molecular changes (trans-fatty acid formation, cross-linking, polymerization, etc), in different oil "feedstock" and the effects from variations in catalysts and hydrogen pressures. GC, IR spectroscopy, and UV-Vis Spectrophotometry will be used to track the changes in the original oil over time and conditions.

*Keywords:* food science, method development, process control, sample preparation
*Application code:* food science
*Methodology code:* chemical methods

### BENEFITS OF ISO 17025 ACCREDITATION FOR PROVIDERS AND USERS OF CALIBRATION GASES USED IN ENVIRONMENTAL TESTING

***Douglas C. King***

*Airgas, Inc, 259 North Radnor-Chester Road, Suite 100, Radnor, PA 19087, USA*

Airgas has received ISO/IEC 17025 accreditation from the American Association for Laboratory Accreditation (A2LA) for three specialty gas laboratory facilities, the scope of which covers a range of calibration gas mixtures having well-defined traceability and uncertainty. The processes used in gaining this unique competency and capability assessment, and the hurdles to be overcome, will be discussed. The author will also provide benefits analyses, both internally derived and those available to end users of EPA protocol gases and other traceable calibration products. Airgas' scope of accreditation covers the majority of potential EPA Protocol gases and NIST traceable mixtures, plus natural gas standards. Fully documented and validated analytical procedures.

*Keywords:* gas, quality, quality control, specialty gas analysis
*Application code:* quality
*Methodology code:* gas chromatography

### ELECTROCHEMICALLY MODULATED SEPARATIONS PERFORMED ON LINE WITH ICP TIME-OF-FLIGHT MASS SPECTROMETRY FOR SEPARATION AND CONCENTRATION OF PLUTONIUM

***Sea H. Park,* Debra A. Bostick, and Douglas C. Duckworth***

**Chemical Science Division, Oak Ridge National Laboratory, PO Box 2008, Oak Ridge, TN 37831-6375, USA*

A hyphenated technique employing electrochemically modulated separation on line with inductively coupled plasma mass spectrometry is being developed to improve the speed, sensitivity, and selectivity of a novel separation and analysis process for plutonium (Pu). A flow-by electrochemical cell with an anodized glassy carbon electrode is interfaced on-line with an inductively coupled plasma time-of-flight mass spectrometer (Optimass 8000, GBC Scientific). Applying positive/negative potentials to the glassy carbon electrode controls accumulation/release of Pu resulting in selective separation from other species in solution including those giving rise to chemical interferences. Parametric investigations reported focus on optimizing conditions for deposition and release of Pu-pH, redox potentials, accumulation time. Results from the parametric studies demonstrate that oxidation potential affects the accumulation efficiency which increases as the potential shifts to positive potentials until it reaches a threshold potential (0.7 V versus Ag/AgCl). Any potential greater than 0.7 V shows minimal improvement over the efficiencies. Varying the accumulation time shows negligible effect on the accumulation efficiency; however, the extensive accumulation time results in saturation of electroactive sites on the anodized glassy carbon and lowers effectiveness of Pu accumulation. Results from the stripping potential study reveals that release rate of Pu strongly depends on the reduction potential applied, and the stripping peak intensity increases until the potential reaches 0 V. Effect of solution pH on the Pu accumulation is also reported.

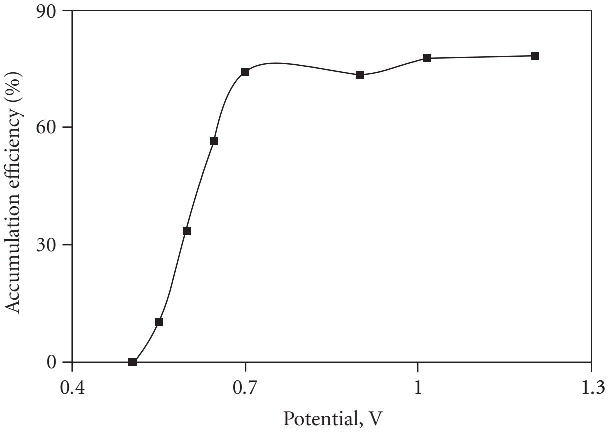

Figure 4

### ACKNOWLEDGEMENTS

This research was sponsored by Office of Research and Development, US DOE, under a contract with Oak Ridge National Lab, managed and operated by UT-Battelle, LLC. The submitted manuscript has been authored by a contractor of the US Government under contract no DE-AC05-00OR22725. Accordingly, the US Government retains a nonexclusive, royalty-free license to publish or reproduce the published form of this contribution, or allow others to do so, for US Government purposes.

*Keywords:* electrochemistry, elemental analysis, ICP-MS
*Application code:* homeland security/forensics
*Methodology code:* mass spectrometry

### ON-LINE PHOTO-IMMOBILIZATION OF PROTEIN INSIDE MICROCHANNEL

***Hizuru Nakajima,* Toshiyuki Hobo, Satomi Ishino, Hironori Masuda, Tatsuro Nakagama, Takuya Shimosaka, and Katsumi Uchiyama***

**Tokyo Metropolitan University, 1-1 Minamiohsawa, Hachioji, Tokyo 192-0397, Japan*

Patterning several kinds of proteins to a specific position on the inner wall of microchannel is very difficult because of the difficulty in making microfluidic device after immobilization of protein which is fragile to heat and organic solvents. We have developed a novel immobilization method to make zones of enzyme inside the microchannel. A photoreactive cross-linker, 4-azido-2,3,5,6-tetrafluorobenzoic acid succinimidyl ester, was incubated with horseradish peroxidase (HRP) beforehand. The solution was introduced into the microchannel, and then UV light irradiated the specific position of the microchannel through a photomask to create the zone of HRP. After washing the microchannel, a zone of alkaline phosphatase (ALP) was similarly created in another place in the same microchannel. The activity of HRP and ALP immobilized on the inner wall of microchannel was investigated using Amplex Red and fluorescein diphosphate (FDP). The microchannel was filled with the solution containing Amplex Red and hydrogen peroxide or FDP. The resulting pattern of immobilized enzymes was imaged under an inverted fluorescence microscope with a CCD camera. Fluorescence was observed only at the position in which the UV light had been irradiated. The result suggests that HRP and ALP are regioselectively immobilized on the inner wall of microchannel with maintaining their activity. The method developed in this study has a wide range of applications because two or more different functional materials, such as antibodies and enzymes, can be immobilized to an arbitrary position inside the microchannel and will become a basic technology of microfabrication for surface functionalization in microchemistry.

*Keywords:* enzyme assays, immobilization, lab on a chip/ microfluidics, protein
*Application code:* high-throughput chemical analysis
*Methodology code:* microfluidics/lab on a chip

### IN-SITU PROBING OF THE BIOTIC-ABIOTIC BOUNDARY OF PLANTS BY LASER DESORPTION/IONIZATION TIME-OF-FLIGHT MASS SPECTROMETRY

***Chanan Sluszny,* Basil J. Nikolau, and Edward S. Yeung***

**Carver Co-Lab, Iowa State University, 35C Roy J, Ames, IA 50011-3650, USA*

Laser desorption/ionization time-of-flight (LDI-TOF) mass spectrometry was applied for the direct analysis of cuticular waxes on intact plant tissues. Cuticular wax compounds were ionized by laser desorption in the presence of colloidal silver. Silver adductions were detected on samples from *Arabidopsis thaliana* and from maize. Good spot-to-spot reproducibility indicated homogeneous coverage of the sample by the fine colloidal material. The results were consistent with GC-MS analyses of cuticular extracts, thus confirming the feasibility of direct analysis based on this protocol. Molecular masses of the adduct ions correspond well with the known composition of cuticular waxes. Moreover, LDI-TOF gave good estimates of the relative local abundances of a given compound. However, bias was found in cases where compounds with different ionization efficiencies were analyzed.

*Keywords:* biological samples, high-throughput chemical analysis, hydrocarbons, mass spectrometry
*Application code:* high-throughput chemical analysis
*Methodology code:* mass spectrometry

### NOVEL CONTINUOUS FREE-FLOW ELECTROPHORESIS INSTRUMENTATION FOR LARGE-SCALE SEPARATION OF MONOLAYER PROTECTED NANOCLUSTERS

***Rachel R. Peterson* and David E. Cliffel***

**Vanderbilt University, PO Box 1822, Nashville, TN 37240-0001, USA*

The use of continuous free-flow electrophoresis (CFE) for isolating individual biological species has been extensively reported in the literature, but has yet to be applied to the isolation of macroscopic quantities of monodisperse nanoparticles. The use of CFE allows for the separation of large-scale amounts (less than 3 grams) in less than 1 hour. In CFE, the sample is flowed through a column while a perpendicular electric field induces the migration of ions towards the column walls. In contrast to typical CFE instrumentation, our novel CFE instrument design for nanoparticle separation eliminates the dependence on the membrane separating the electrodes from the analyte and minimizes the effect of gravity. CFE is a high-resolution analytical technique that can be utilized for the separation of nano-sized materials such as monolayer protected nanoclusters (MPCs). MPCs are metal nanoparticles that have unique optical, chemical, and electrochemical properties resulting from their size and lending them useful in the developing field of nanotechnology. MPC particle size manipulation would allow valuable control of the MPC characteristics for use in industry and academics. CFE separation of tiopronin protected MPCs, using a commercial CFE, results in a visible color gradient that indicates MPC fractionation and proves good anodal migration of the MPCs. This reveals that the use of CFE for MPC separation is a viable solution to isolating macroscopic quantities of monodisperse samples of MPCs for possible use in nanoelectronics.

### ACKNOWLEDGEMENTS

Partial funding was provided by the Boeing Company and the Vanderbilt Institute of Nanoscale Science and Engineering.

*Keywords:* electrophoresis, nanotechnology, particle size and distribution, separation sciences
*Application code:* nanotechnology
*Methodology code:* separation sciences

### ON-LINE AND ON-SITE QUALITY CONTROL OF POLYMERIZATION PROCESSES

***Joerg Ingo Baumbach,[*] Ralf Guesthuisen, and Wolfgang Vautz***

*[*]ISAS Institute for Analytical Sciences, Bunsen-Kirchhoff-Street 11, Dortmund 44139, Germany*

The real value of the trace concentration of the initial monomers remaining in the final product of polymerization processes is characteristic for the quality of the product. Beside unpleasant odor, common monomers (eg, styrol, n-butylacrylate, methylmetacrylate, vinylacetate) are candidates causing allergies and various lung diseases. Therefore, accurate monitoring of the monomere concentration during the run of the polymerization process should be used as a measure to determine the actual state of the process. Thus, the monitoring of the process would guarantee a polymerization as complete as possible. Finally, quality standards could be controlled directly and the quality of the final polymeric product can be safeguarded. Both 63 Ni and 10.6 UV-ion mobility spectrometers are used for on-line detection and quantification of various monomers selected. The challenge of the experiments includes a rather wide concentration range from the start until the end of the polymerization process and high humidity of the analyte. The integration of the ion mobility spectrometer developed into the process and the treatment of the carrier gas containing the analytes will be discussed in detail. The results of laboratory investigations will be shown.

*Keywords:* polymers and plastics, process analytical chemistry, process control, spectrometer
*Application code:* polymers and plastics
*Methodology code:* sensors

### AUTOMATIC CONTINUOUS ONLINE MONITORING OF POLYMERIZATION PROCESSES

***John A. McConville,[*] Stephen O'Donohue, and Wayne Reed***

*[*]Polymer Laboratories Inc, Amherst Fields Research Park, 160 Old Farm Road, Amherst, MA 01002, USA*

Monitoring the progress of polymerization processes as they occur within a reactor has important implications for commercial polymer manufacture. Minimizing reactor down time, optimizing the product and increasing the efficiency of manufacture can have a high impact upon the profitability of polymer production. In this paper, details of a new system for the automatic continuous online monitoring of polymerizations (ACOMP) shall be presented. In ACOMP, the contents of the reactor are continuously extracted and diluted producing a stream which passes through a series or "train" of detectors. The stream of liquid is so dilute that detector signals are dominated by the properties of single polymers, not their interactions.

We describe uses of the ACOMP technique in the discovery and understanding of kinetics and mechanisms.We will show how polymerization reactions have been optimized at both the laboratory and pilot plant level, and present details of how ultimately the technique could be used to monitor and provide full feedback control for full-scale industrial reactors.

Some of the parameters that may be monitored in ACOMP by absolute means include molecular weight, indices of polydispersity, viscosity, and percentage conversion. When ACOMP is applied to copolymerization reactions composition and sequence length distributions can additionally be determined.

*Keywords:* materials characterization, on-line, polymers and plastics, process monitoring
*Application code:* polymers and plastics
*Methodology code:* others

### PERFORMING AUTOMATED DATA REVIEW AND DATA QUALITY ASSESSMENT ON SEDD FILES

***Scott Denzer*** [*] ***and Pamela Wehrmann***

[*] *Laboratory Data Consultants, 7750 El Camino Real, Suite 2L, Carlsbad, CA 92009, USA*

This presentation discusses the use of staged electronic data deliverable (SEDD) files and automated data review and data quality assessment software to meet project specific electronic data management goals. The software applications, automated data review (ADR) and environmental data management system (EDMS), were developed under contract with the Army Corps of Engineers - Sacramento District and assistance with defining project specific electronic data deliverable requirements and automating the review and data quality assessment processes. Project electronic deliverable requirements are developed as a library within ADR and electronically transferred to the laboratory. Requirements include specific measurement quality objectives established for each analyte in the various analytical methods performed for the project. This information is used by the laboratory to ensure that the contents of a SEDD file meet project electronic deliverable requirements and is also used by the data user to perform an automated review of the data. Discussion will center on parsing data from SEDD files, automated systems to verify compliance with project specific data integrity and content rules, automated review of data, and portability of data from SEDD to other applications.

*Keywords:* automation, informatics, laboratory informatics, sample and data management
*Application code:* environmental
*Methodology code:* laboratory informatics

### CUSTOM DATABASE APPLICATION FOR STORAGE, REVIEW, MANAGEMENT

***John Morrow,*** [*] ***Benjamin C. Blount, and Robert Jones***

[*] *Centers for Disease Control and Prevention, 1600 Sifton Rd, Atlanta, GA 30341, USA*

Our laboratory has developed a customized database system to manage analytical results data from several different instruments, based on commercial database software. Instruments from several different vendors provide data in differing, proprietary formats. These data are transferred via network and removable storage devices, and collected in a central network location. Data files are converted to compatible formats as needed. Results data are exported to text or spreadsheet files, again with formats differing widely among vendors. Custom database queries and modules are used to import these results into the laboratory database. The database system facilitates tracking samples, results, and QC data. Furthermore, the database aids in QA/QC review of sample results and reporting of results in differing formats as needed by data users. Throughout this process, we maintain ready access to all laboratory notes and data files at every level. This database system has enabled this lab to manage more data, instruments, analysts, and data end users, while retaining flexibility to import and export data in multiple formats as needed.

*Keywords:* automation, informatics, laboratory informatics, sample and data management
*Application code:* environmental
*Methodology code:* laboratory informatics

### MERCURY MEASUREMENTS: OVERCOMING CONTAMINATION AND INTERFERENCES TO ENABLE THE CORRECT MEASUREMENTS FOR VALUED JUDGEMENTS USING EPA 1631

***Peter B. Stockwell,*** [*] ***Derek W. Bryce, and Warren T. Corns***

[*] *P S Analytical Ltd, Arthur House, Crayfield's Industrial Estate, Main Road, Orpington, Kent BR5 3HP, UK*

Interest and concern over the levels of mercury in its various forms in the environment has been well documented over the last 50 years. Organomercury compounds in particular have received widespread attention, highlighted by the Minamata Bay disaster which sparked off concern over mercury in aqueous ecosystems. Mercury levels in water, soil, petrochemicals, and coal-fired stack emissions have been regulated for many years. Permitted levels of mercury are being lowered by regulatory bodies, placing significant demands on the various standard methods of analysis approved both in the USA (EPS methods 1631, 245.1, 245.7 and SW-846 747770-74-71) and the CEN 13506 (soon to be integrated into the ISO regime). The criterion for fresh water systems is currently set at 12 nanograms/liter (ng/L) and for saltwater the level is 25 ng/L. While there are several instruments on the market which can readily measure such levels, there are still major issues to be addressed in order to obtain accurate measurements. Sampling, storage, pretreatment, contamination, and interferences are all potential sources of error which should be carefully considered before and during analysis using the EPA 1631 approach.

*Keywords:* mercury
*Application code:* environmental
*Methodology code:* atomic spectroscopy/elemental analysis

### MERCURY ANALYSIS IN SOLIDS, LIQUIDS, AND GASES ON A SINGLE INSTRUMENT

***Jason P. Gray,*** [*] ***Alvin Chua, and Koji Tanida***

[*] *AGS Scientific, Inc, 1511 Texas Avenue S, Suite 270, College Station, TX 77840, USA*

Mercury (Hg) is a well-known environmental pollutant that is introduced into our environment through emissions from coal-fired plants, automobiles, and other industrial processes, eventually spreading through all phases of our environment. Mercury contamination levels are monitored in our air, water, and soils in every state within the United States. Most of these measurements are performed at the request of the United States Environmental Protection Agency (EPA), which requires the use of multiple analytical methods to accomplish these tasks. There are Methods 245.1 and 245.2 for drinking and wastewaters, Methods 245.7 and 1631-Rev E for low levels in wastewaters, Methods 245.5 and SW-846 7473 for solids, soils, and sludges, along with a variety of recommendations for air monitoring. With such a wide variety of methods required for compliance, a vast array of analytical instruments has been designed to meet each single requirement. A new mercury analyzer for the analysis of mercury in waters, solids, and gases will be described in this presentation. This analyzer functions on the principles of oxidation, purge and trap, and CVAAS for low-level waters analysis and thermal decomposition, amalgamation, and CVAAS for solids and gases. This presentation includes supporting data and illustrations of this new mercury analyzer's capabilities.

*Keywords:* atomic spectroscopy, environmental analysis, mercury, trace analysis
*Application code:* environmental
*Methodology code:* atomic spectroscopy/elemental analysis

### DETERMINATION OF INORGANIC MERCURY BY FLOW INJECTION COLD VAPOR GENERATION WITH TIN(II) CHLORIDE IMMOBILIZED ON AN ANION-EXCHANGER WITH IN-ATOMIZER TRAPPING BY ELECTROTHERMAL ATOMIC ABSORPTION SPECTROMETRY

***Wipharat Chuachuad*** **[*] *and Julian F. Tyson***

[*]*University of Massachusetts, 710 N Pleasant Street, Amherst, MA 01003-9306, USA*

A new method for the determination of inorganic mercury by flow injection cold vapor generation from tin chloro anion immobilized on a strong anion-exchange resin with in-atomizer trapping by ETAAS has been developed. Both ETAAS and flow injection hydride generation parameters were optimized. The method offers several advantages such as convenience, and smaller amounts of reagents are required. A purer reagent of tin(II) chloride reagent was obtained by passing tin(II) chloride through the anion exchange column without prior purification. The method was based on using an anion-exchange resin (Amberlite IRA-400) to immobilize the anionic tin chloro complex before passing the acidified sample. The effects of column dimensions, types of resins, concentration of tin(II) chloride, loading time, loading flow rate, carrier reagent flow rate, carrier gas flow rate, and acidity of sample by the flow-based system were investigated. The sensitivity of inorganic mercury from suitable metal-coated graphite tubes, for example, Ir (250 mg), and Au (250 mg) were compared. The highest-sensitivity and well-defined peak profiles were obtained from the Au-coated graphite tube. The optimized trapping, pyrolysis and atomization temperatures for the ETAAS were 50°C, 50°C, and 900°C, respectively. Sub-ppb level of limit of detection was obtained from this technique, with good accuracy and precision. The method was applied for the determination of inorganic mercury in some natural waters, seawater, whale liver, and goat blood samples and validated for the determination of inorganic mercury in standard reference materials DORM-2 (dogfish muscle) and DOLT-2 (dogfish liver).

*Keywords:* atomic absorption, environmental/biological samples, mercury
*Application code:* environmental
*Methodology code:* atomic spectroscopy/elemental analysis

### THE USE OF ATOMIC FLUORESCENCE SPECTROMETRY FOR MERCURY DETERMINATION IN THE PETROCHEMICAL INDUSTRY

***Warren T. Corns***

*P S Analytical Ltd, Arthur House, Crayfield's Industrial Estate, Main Road, Orpington, Kent BR5 3HP, UK*

Accurate measurements of mercury in petrochemical feedstocks and resulting products is critical for refining operations since the presence of mercury even at low concentrations can have a detrimental effect on numerous refining operations. These include the poisoning of expensive hydrogenation catalysts, corrosion of aluminium alloys of steam cracker cold boxes, and reducing product quality. There are also environmental aspects that have to be considered, since the combustion of hydrocarbons may contribute to the atmospheric emissions of mercury to the atmosphere. Removal of mercury from petrochemicals is extremely challenging and the optimization of such processes cannot be achieved without knowledge of the mercury species present in the sample and how they might be transformed during refining operations. This paper will describe how atomic fluorescence spectrometry (AFS) can be applied to the measurement of mercury in petrochemical samples. Online and offline measurement of mercury in natural gas was achieved using dual amalgamation—AFS. Data will be presented showing the effectiveness of mercury removal beds. Naphtha and gasoline samples were analyzed using a novel volatilization technique coupled to amalgamation at elevated temperature. This approach allowed part-per-trillion detection limits without the need for sample preparation. Mercury fractionation in crude oil and condensates was established using various selective extractions with subsequent measurement by cold vapor—AFS. Organic and elemental mercury was determined using a specially designed capillary GC-AFS after direct injection of filtered samples. Experiences from working in the field and typical data from refineries around the world will be presented.

*Keywords:* mercury
*Application code:* environmental
*Methodology code:* atomic spectroscopy/elemental analysis

### MERCURY SPECIATION IN FLUE GAS BY CATALYTIC CONVERTERS

***Matthew A. Dexter,* Warren T. Corns, and Peter B. Stockwell***

**P S Analytical Ltd, Arthur House, Crayfields Industrial Estate, Main Road, Orpington, Kent BR5 3HP, UK*

Gas-phase mercury in power plant stack-gas emissions is present as elemental mercury ($Hg^0$) and oxidized mercury ($Hg^{2+}$, principally $HgCl_2$). Current legislative developments are increasing demand for a robust, low maintenance mercury continuous emission monitor (CEM). Existing wet chemical methods for speciating mercury in flue gas and preconditioning the sample for analysis involve the use of large quantities of reagents, producing similar volumes of liquid waste, and require considerable operator skill and attention. The flue gas matrix presents particular challenges. Firstly, representative sampling from the stack, including separation from the reactive flash without this affecting the mercury speciation or level in the flue gas is necessary. Secondly, the composition of the flue gas contains significant levels of a range of acid gases (eg, sulfur dioxide, nitrogen oxides, hydrogen chloride), which can interfere with the determination of mercury. A dry-based mercury speciation module will be described and preliminary results shown. Mercury is measured in two streams, total gas-phase mercury ($Hg^T$) and $Hg^0$; thus speciation by difference can be achieved. A high-temperature catalytic process is used to convert $Hg^{2+}$ to $Hg^0$ for measurement in the $Hg^T$ stream and a $Hg^{2+}$ adsorbent is used to remove $Hg^{2+}$ from the $Hg^0$ stream. A Peltier cooler is used to remove water from the matrix, prior to determination of mercury in the two sample streams by amalgamation of atomic fluorescence spectroscopy (AFS).

*Keywords:* speciation
*Application code:* process analytical chemistry
*Methodology code:* atomic spectroscopy/elemental analysis

### DEVELOPMENT AND APPLICATION OF A FLOW INJECTION HYDRIDE GENERATION ATOMIC FLUORESCENCE SPECTROMETRIC (FI-HGAFS) METHOD FOR DETERMINATION OF TOTAL ARSENIC IN HAIR SAMPLES

***Jon Clark* and Olujide T. Akinbo***

**Butler University, 4600 Sunset Avenue, Indianapolis, IN 46208, USA*

Although there is much debate about the inability to distinguish between exogenously and endogenously incorporated arsenics in hair matrix, there is also a strong incentive to use hair as a biomarker of exposure to arsenic. Determination of arsenic in hair matrix is useful as a screening tool in environmental pollution and occupational exposure studies. Moreover, factors such as ease of transport, storage, relatively noninvasive sample collection, record of long-term exposure, and its noninfectious nature give hair an advantage over other biological tissues (eg, blood and urine). Therefore, it is pertinent to develop and refine methods for determination of arsenic in hair. Several atomic spectrometric methods have been used in arsenic determination in hair but HG-AFS is attractive because of its high sensitivity, wide linear dynamic range, speed of analysis, ease of use, and low costs. In spite of these qualities, HG-AFS suffers a disadvantage with matrices that affect the hydride generation reaction. For example, undiluted acid digestate of hair samples can cause vigorous reaction resulting in poor precision, flame instability or extinguishment. Also, large amount of sample is needed (6–10 ml per replicate) for analysis in addition to large volume of highly concentrated reagents (eg, 30% HCl). To address all these problems, flow injection (FI) sample introduction was investigated. The factors optimized include sample volume required for analysis, concentration and flow rates of the reagents in the carrier stream. The analytical performance (precision, detection limits range for linearity) of the FI-HGAFS will be discussed.

*Keywords:* elemental analysis, environmental/biological samples, flow injection analysis, trace analysis
*Application code:* environmental
*Methodology code:* atomic spectroscopy/elemental analysis

### DEVELOPMENT OF A MICROWAVE SAMPLE PREPARATION METHOD FOR TOTAL ARSENIC DETERMINATION IN EDIBLE OILS USING FLOW INJECTION HYDRIDE GENERATION ATOMIC FLUORESCENCE SPECTROMETRY

***Megan Bergauff* and Olujide T. Akinbo***

**Butler University, 4600 Sunset Avenue, Indianapolis, IN 46208, USA*

Arsenic is a toxic metalloid and is ubiquitous in nature. Human exposure to arsenic is mainly through ingestion of food and water. Although the presence of arsenic in water and composite food has been widely studied and reported, there have been fewer reports on the arsenic level in edible oils and hence its contribution to arsenic found in food composites. The weakest link in many atomic spectrometric methods used for arsenic determination in environmental samples is the sample preparation step. This is very pertinent to the determination of arsenic in edible oils given the complexity and toughness of this matrix. The aim of this study is to develop a method for extracting arsenic from edible oil prior to analysis by flow injection hydride generation atomic fluorescence spectrometry (FI-HG-AFS). For this purpose, various acids and acid combinations have been investigated and nitric acid was found to be most suitable.

However, other problems such as false positive signal from the acid digestion blank even when an ultrapure nitric acid was used, and arsenic signal broadening and suppression in oil digestates have forced the need for acid evaporation and solvent extraction of digestate prior to instrumental analysis.

Among the solvents examined for extraction, chloroform was found to be most suitable. The impact of these sample pretreatments on the analytical performance of the FI-HG-AFS method and results obtained from application of the method to 14 market basket edible oil samples will be presented.

*Keywords:* environmental analysis, flow injection analysis, method development, trace analysis
*Application code:* environmental
*Methodology code:* atomic spectroscopy/elemental analysis

### PRECONCENTRATION AND SPECIATION OF ARSENIC IN ENVIRONMENTAL SAMPLES WITH SOLID-PHASE EXTRACTION AND FI-HGAAS

***Khalid H. Alassaf,[*] Marissa K. Callahan, and Julian F. Tyson***

*[*]Department of Chemistry, University of Massachusetts, 701A Lgrt 710 North Pleasant Street, Amherst, MA 01003-9306, USA*

Arsenic is an analyte of high concern all over the world due to its toxic properties. The toxicity and mobility in the environment are dependent on the chemical form or species in which it exists. Solid-phase extraction offers a number of advantages. It reduces (a) solvent usage and exposure, (b) analysis and disposal costs, and (c) the extraction time for sample preparation. Anion- and cation-exchange, silica-based cartridges were used sequentially in order to quantitatively separate and preconcentrate arsenite [As(III)], arsenate [As(V)], monomethylarsonic acid (MMA), and dimethylarsinic acid (DMA). DMA was retained on a silica-based, strong cation-exchange (SCX) cartridge and eluted with 1.0 M nitric acid. MMA and As(V) were both retained on a silica-based, strong anion-exchange (SAX) cartridge and sequentially eluted with 0.1 M acetic acid for MMA and 1.0 M nitric acid for As (V). As(III) was not retained on either cartridges and remained in the solution. However, at pH $>$ 9.5, As (III) was fully retained on a resin-based, strong-anion exchanger. The sequentially eluted arsenic species were determined by flow injection hydride generation atomic absorption spectrometry (FI-HGAAS). The method was applied to the speciation of arsenic in pond water, plants, and soils. The recoveries of arsenic species in pond water sample spiked with 1 $\mu$g/L of mixture of the four species were 99% for As (V) and 107% for both MMA and DMA. As(III) was difficult to elute from the SAX even when concentrated hydrochloric acid (6 M) was used (45% recovery). However, using a combination of 3:1 nitric acid (3 M) and hydrochloric acid (3 M) as a solvent to elute As (III) was very successful. This combination of acid gives a 98% recovery for As (III).

*Keywords:* atomic absorption, hydride, solid-phase extraction, speciation
*Application code:* environmental
*Methodology code:* atomic spectroscopy/elemental analysis

### APPLICATION OF FACTORIAL DESIGN AND DOEHLERT MATRIX IN OPTIMIZATION PRECONCENTRATION PROCEDURE FOR CADMIUM DETERMINATION IN SEAWATER BY FLAME ATOMIC ABSORPTION SPECTROMETRY

***Adriana C. Ferreira,[*] Sérgio L. Ferreira, and Maria das Graças A. Korn***

*[*]SENAI CETIND, Av Luiz Tarquínio, 938 Aracuí, Lauro De Freitas 42700-000, Brazil*

A method for the determination of trace amounts of cadmium in seawater by flame atomic absorption spectrometry after preconcentration on activated carbon has been developed. It is based on the solid-phase extraction of cadmium (II) ions as a 4-(2-pyridylazo- resorcinol) (PAR) chelate using activated carbon as a sorbent. The optimization of the experimental factors (pH, activated carbon mass, PAR mass and shaking time) was carried out using a two-level full factorial design (24) and two Doehlert matrix designs. The results of the factorial design, considering the analysis of variance (ANOVA), demonstrate that all these factors and the interactions (pH $\times$ PAR mass) and (PAR mass $\times$ activated carbon mass) are statistically significant. The final optimization was carried out using Doehlert matrix designs considering the results of the factorial design. With the application of the Doehlert designs in solid-phase spectrophotometry, the optimization of variables can be performed simply, quickly, and with greater efficiency compared to traditional methodology. The validation process evaluated the following parameters: effect of other metal ions, calibration curve, precision, accuracy, and robustness.

The procedure allows for cadmium determination in seawater samples, with limit of detection (3 s/S) and quantification (10 s/S) of 8.3 $ngL^{-1}$ and 27.7 $ngL^{-1}$, respectively, and an experimental enrichment factor of 149 is easily achievable. The results proved that the procedure is not affected by matrix interferences. The method has been applied to numerous seawater samples collected in Salvador City, Brazil, for the determination of cadmium at ppb levels. The concentration found ranged between 0.035 and 0.17 $mgL^{-1}$ with good precision and accuracy.

*Keywords:* atomic absorption, chemometrics, ultratrace analysis, water
*Application code:* environmental
*Methodology code:* atomic spectroscopy/elemental analysis

### IMPROVING THE DETECTION LIMITS OF ICP-OES FOR SELENIUM BY FI SAMPLE INTRODUCTION PROCEDURES

***Princess Hernandez,[*] Julian F. Tyson, Peter C. Uden, and Dennis Yates***

*[*]Department of Chemistry, University of Massachusetts, 710 North Pleasant Street, Amherst, MA 01003-9306, USA*

The combination of flow injection hydride generation and preconcentration by solid-phase extraction with direct coupling of the eluent to the spectrometer offers the possibility of accurate analyses at concentrations below the normal detection limits of ICPOES. The mass flux is increased by replacing the transfer of material as small droplets isolated at 1%–2% efficiency from the spray generated from liquid introduced at 1 mL $min^{-1}$ with the introduction of gaseous molecules generated from a sample stream introduced at up to 5 mL $min^{-1}$. Secondly, interferences in the solution-phase, hydride generation reaction can be removed by trapping the analyte and discarding the sample matrix. This process also provides solution-phase preconcentration. The possibilities of these approaches have been evaluated for selenium as a test element. The replacement of the nebulizer and spray chamber with a flow injection hydride generation (FI-HG) system, whose gaseous eluent from the gas-liquid separator was connected directly to the end of the injector tube, produced a decrease in detection limit of 10 times. The incorporation of a strong anion-exchange material into the manifold with release into an acid eluent produced a further decrease in detection limit of 30 times. The procedure was applied to the determination of selenium in both environmental and clinical samples.

*Keywords:* environmental analysis, flow injection analysis, plasma emission (ICP/Mip/DCP/etc), sample introduction
*Application code:* environmental
*Methodology code:* atomic spectroscopy/elemental analysis

### ATOMIC ABSORPTION DETECTION OF TIN AND TELLURIUM BASED ON HYDRIDE TECHNIQUE

***Abdumalik Y. Khudayberdiev*** ***and Shahista A. Ishniyazova***

**Samarkand Economy and Service Institute, Usto Kuli Street 35, Samarkand 703021, Uzbekistan*

A principle, which is laid on definition of hydride-forming elements by atomic absorption, consists in making generation of hydrides from water solutions by the help of first group borohydrides and giving obtained gas mixtures to atomizing cell. Metrological characteristics of tin determination by atomic absorption method depends on material from which an atomizer is made. Furnaces which are covered with tungsten have the highest degree of sensitivity coefficient. This can be explained as follows. As a result of gas saturation, which is output from a generator, by water vapors, tin accumulates on the surface of the atomizer as an oxide film. After furnace heating, atoms form as a result of tin oxide reduction by tungsten carbide. As in the case with graphite furnace, also with the furnace that is covered with tungsten, after absorption measuring, there is a residue signal, because of element carbidization.

Residue removing needed to hold furnaces at temperature of atomization for not less than 15 seconds or 2–3 times heat the furnace up to appropriate temperature. It can lead to quick break of furnace. Relative standard deviation by using them is 0, 24–0, 25. When furnaces covered with tantalum are used, the residue signal after heating furnace for 1–2 seconds is not detected, and relative standard deviation not more than 0, 14. It can be done more than 50 element determinations. That is why, during tin determination it is recommended to use furnaces covered with tantalum foil. It is established that tellurium extraction degree (R %) depends on generator material and form. When generators that are made of plexiglass were used, the R was less than 15%. If the generator is made of glass, the degree of extraction increases. For a generator that has cylindrical form, R is 75%–80%, and for a generator with sphere form the extraction degree is near 95%. It turns out to be inadmissible to use polyvinyl chloride pipes for tellurium hydrogen feed, as the extraction degree decreases. It can be concluded that polymers' high molecular surfaces accelerate thermal decomposition of tellurium hydrogen.

*Keywords:* atomic absorption, detection, elemental analysis, hydride
*Application code:* environmental
*Methodology code:* atomic spectroscopy/elemental analysis

### A NEW ANALYTICAL SYSTEM BASED ON ELECTROSTATIC PRECIPITATION IN A FURNACE OF ZEEMAN SPECTROMETER FOR DIRECT AND RAPID DETERMINATION OF ELEMENTS IN AMBIENT AIR AND EXHALED AIR

***Natalija B. Ivanenko*** ***and Alexander A. Ganeev***

**Lumex Ltd, 19 Moskovsky pr, Saint Petersburg 198005, Russia*

Air pollution by some metals absorbed into aerosols is a growing problem. Global pollution is mainly associated with airborne particles and various gases exhausted from industrial facilities. The recognized need of continuous air quality monitoring in sites has stimulated efforts to adapt laboratory analyzers for measurements in situ.Traditional methods for monitoring of airborne particles are both labor-intensive and time-consuming ones. The airborne particulate matter is collected for hours in filters, made from fiberglass, cellulose esters, or other materials with pore sizes ranging from 0.05 to 0.5 mm. For a short precipitation time the level of filter contamination for the great number of elements exceeds the quantity of the precipitated element; thus the precipitation generally takes several hours. Technique of rapid elements determination in air (without aerosols precipitation on filters) is very attractive, since in this case background fluctuations of elements in filters are avoided, laborious filter treatment is removed, and precipitation time is decreased significantly. Moreover analyzers based on this technique allow to determine elements concentration in air automatically in situ. This poster is devoted to the development of novel analytical equipment based on a technique of electrostatic aerosols precipitation from air directly into a graphite furnace. The equipment includes Zeeman spectrometer with

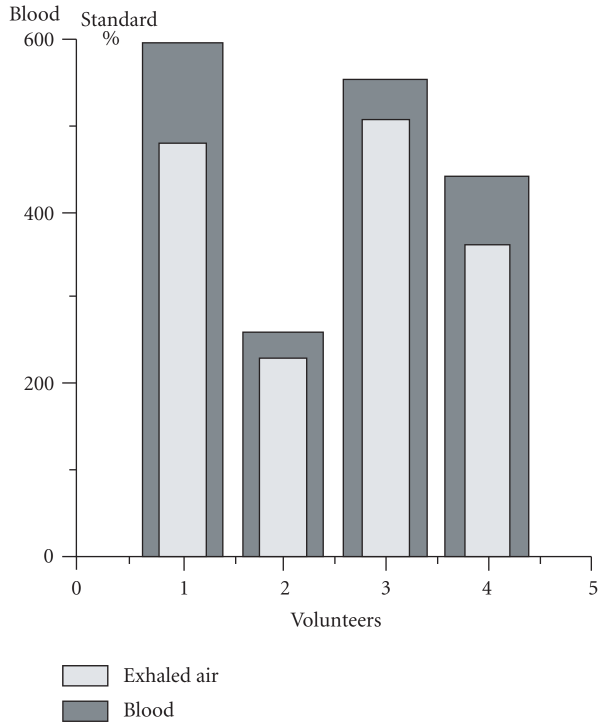

Figure 5

high frequency modulation polarization-MGA-915 and electrostatic precipitation system incorporated in analyzer. Analytical characteristics and optimized parameters of the new system are presented. The optimization procedure is carried out taking into account the theoretical model of electrostatic precipitation efficiency. The efficiency of aerosols electrostatic precipitation process after parameters optimization is estimated to be close to 100%—the results of elements contamination (Pb, Cr, Cd, Mn, and Cu) determined in laboratory air by the new system are in agreement with the elements concentration determined by classical filter precipitation to better than 5%. Low detection limits of the system allow for using it not only for determination of elements concentrations not only in ambient air but also in the air exhaled by humans. Concentration of Se in exhaled air correlates with Se concentration in blood for some volunteers (see Figure 5).

*Keywords:* air, atomic absorption
*Application code:* environmental
*Methodology code:* atomic spectroscopy/elemental analysis

### DEVELOPING STANDARD METHODS FOR ANALYSIS FOR ARSENIC, SELENIUM, AND ANTIMONY

***Peter B. Stockwell***

*P S Analytical Ltd, Arthur House, Crayfield's Industrial Estate, Main Road, Orpington, Kent BR5 3HP, UK*

As the regulation requirements for the levels of arsenic, selenium, and antimony are reduced, there becomes an urgent requirement for standard methods of analysis that is suitable for a global market. As part of its opening commitment to the improvement in the environment, P S Analytical has devoted time to working with clients in the European market and globally to develop standard methods of analysis that are suitable for use worldwide and which are fit for purpose of that making use of both atomic absorption spectrometry and atomic fluorescence spectrometry.

A template for these standards is fast established for the determination of antimony by AAS and AFS. Following consultation, further standard methods for arsenic and selenium have been established. Currently these standards are being circulated as a committee draft, having been discussed and appraised by the technical working group. Once completed, the methods will need to be validated. An interlaboratory trial will be coordinated so that these standard methods can be validated. When resources are so stretched to measure environmental pollutants it seems eminently sensible that standards developed within CEN and ISO should be available to EPA and the USA client base. Further development of these standards towards methods for speciating these elements will also be described.

### ACKNOWLEDGEMENT

Peter B Stockwell is the convenor of working group 52 TC147-SC2.

*Keywords:* atomic spectroscopy, environmental analysis
*Application code:* environmental
*Methodology code:* atomic spectroscopy/elemental analysis

### SPECIATION OF ARSENIC IN CORNISH SOILS

***Derek W. Bryce,* Warren T. Corns, Hylke J. Glass, Chris Hutton, and Loveday Jenkin***

**P S Analytical Ltd, Arthur House, Unit 3, Crayfields Industrial Estate, Main Road, Orpington, Kent BR5 3HP, UK*

Cornwall (UK) has suffered extensive arsenic pollution due to the historic mining and processing of mineral ores. Current standard practice for contaminated land risk assessment is possibly unviable in Cornwall with very large numbers of sites classified as "contaminated." Methods of measuring the speciation and mobility of arsenic, rather than just total soil concentration, are essential for effective and rapid risk assessments of arsenic contamination. Analytical instrumentation for the speciation and analysis of arsenic based on HPLC-AFS will be described, along with the optimization of a microwave-assisted extraction procedure. This procedure was used to study arsenic speciation in a contaminated site. A heavily contaminated agricultural site in Cornwall where total soil arsenic values range between 399 and 4264 ppm was subject to detailed characterization. Soil pore water was sampled using lysimeters and speciated by high-performance liquid chromatography-hydride generation - atomic fluorescence spectroscopy (HPLC-HG-AFS). Results show generally low levels of arsenic (0–86 ppb) in November 2003, with a variable distribution between arsenate and arsenite. Soil samples removed from the lysimeter sampling points were subjected to a novel method of microwave extraction in orthophosphoric acid. This has been shown to preserve in-situ speciation of arsenic following extraction from solid phases. Analysis of this extract by HPLC-HG-AFS shows arsenic in solid phases to consist predominantly of arsenate. The data suggest that arsenic is relatively immobile under the prevailing environmental conditions. Solid-phase extraction data support earlier work showing arsenic to be immobilized in diagenetic iron oxides. Variation in the species distribution within the soluble fraction is probably due to soil heterogeneity, with clay lenses and organic material creating potentially reducing microsites in an otherwise aerobic soil. This suggests that a large fraction of the arsenic appears to be immobile. Data from such methods could provide a better baseline for meaningful risk assessment.

*Keywords:* analysis, soil
*Application code:* others
*Methodology code:* atomic spectroscopy/elemental analysis

### SEPARATION AND QUANTITATION OF ARSENIC SPECIES IN FOOD AND DIETARY SUPPLEMENTS BY HPLC AND ICP-MS

***Sang-Ho Nam,* Stephen G. Capar, and John Cheng***

**Elemental Research Branch, FDA Center for Food Safety and Applied Nutrition, College Park, MD 20740-3835, USA*

Speciation of arsenic in food and dietary supplements is essential in order to provide a meaningful assessment of exposure due to differences in toxicities of the chemical forms. In this study, HPLC (high-performance liquid chromatography) has been coupled with ICP-MS (inductively coupled plasma-mass spectrometry) to separate and quantitatively determine arsenic species. Various sample preparation and extraction procedures were investigated and compared using 2 reference materials, NIST 1568a rice flour and NIST 1570 spinach, and a freeze-dried apple sample. ICP-MS was used for the measurement of total arsenic. Rice flour was extracted by accelerated solvent extraction (ASE) with deionized water, 25%, 50%, and 100% methanol at 40°C and 100°C. Total arsenic extraction efficiency was 64%, 65%, 58%, and 42% with deionized water, 25%, 50%, and 100% methanol, respectively, at 40° C. Evaporating the methanol and concentrating the extracts using a vacuum centrifuge and reconstituting in deionized water had slightly lower results, 58%, 56%, 52%, and 40%, respectively. Trifluoroacetic acid (TFA) was used to extract spinach, freeze-dried apple, and rice flour. Total arsenic extraction efficiency was 88% and 92% for spinach based on the certificate value and our own analysis, 75% for freeze-dried apple, and 83% for rice flour when an elevated extraction temperature of 100° C was used. An enzymatic extraction method with alpha-amylase and sonication was also investigated. Extraction efficiency was 104% for rice flour, 98% for freeze-dried apple, and 7% for spinach. The highest extraction efficiency obtained for rice flour and freeze-dried apple was with the enzymatic extraction method (98%–104%), but it was not effective for spinach (7%). For spinach, the TFA method provided the best extraction efficiency (88%–92%). The chromatograms of arsenic species extracted by the optimum extraction methods for rice flour, freeze-dried apple, and spinach, and the species extraction efficiencies are compared and presented.

*Keywords:* food science, HPLC, ICP-MS, speciation
*Application code:* food science
*Methodology code:* atomic spectroscopy/elemental analysis

### SCANNING ELECTROCHEMICAL MICROSCOPY STUDY OF MOLECULAR TRANSPORT THROUGH THE NUCLEAR PORE COMPLEX

***Jidong Guo* and Shigeru Amemiya***

**Department of Chemistry, University of Pittsburgh, 219 Parkman Avenue, Pittsburgh, PA 15260, USA*

The nuclear pore complex (NPC) is a protein assembly that spans the double membrane of the nuclear envelope (NE) of eukaryotic cells. The large pore (10–40 nm in diameter) of the NPC functions as a unique gate for molecular exchange between the nucleus and cytoplasm compartments. The nucleocytoplasmic transport system attracts increasing attentions for both fundamental interests and future therapeutical treatment such as gene therapy. We applied scanning electrochemical microscopy (SECM) to measure permeability of the NPC. In SECM permeability measurements, an ultramicroelectrode (UME) probe is brought close to the NE, where the flux of redox active mediators such as ferrocene derivatives and $Ru(NH_3)^{3+}$ is detected amperometrically without any contact between the probe and the NE. The resulting current-distance curve, called approach curve, reflects the permeability of the NPCs.

Experimental approach curves were found to fit well with theoretical curves under diffusion-controlled conditions as obtained by numerical simulations. To illustrate the diffusion process beneath NE and understand approach curve better, concentrations and diffusion coefficients of these mediators inside nucleus were determined by chronoamperometry at a short tip-NE distance, confirming the diffusion limitation. These results indicate that mediator transport through the NPC is much faster than the mediator diffusion in the nucleoplasm. The fast transport kinetics corresponds to the large channel size. Indeed, the diameter of an NPC pore was estimated from the approach curves to be greater than 15 nm. Single-channel current was also estimated to be high enough for single-channel detection of NPC by SECM.

*Keywords:* biological samples, electrochemistry, microelectrode
*Application code:* bioanalytical
*Methodology code:* electrochemistry

### HOW TO MAKE THE BEST BETTER? OPTIMIZATION OF A HIGH-THROUGHPUT PURIFICATION PROCESS

***Xu Zhang,[*] Zhang Bailin, Wolfgang Goetzinger, Bi Grace, Peter Tidswell, and Marc Towle***

*[*]ArQule, Inc, 19 Presidential Way, Woburn, MA 01801, USA*

High-throughput purification techniques are an important part in providing high-quality compounds for biological screening. In the drug discovery process, the bottleneck has gradually shifted from lead generation (LG) to lead optimization (LO). This has posted a new challenge for high-throughput purification. Compound libraries from LO can possess wider diversity in physicochemical properties and hence require different approaches in purification. We have developed a purification process that allows for method optimization for each individual compound without compromising the overall purification throughput. In this process, HPLC method selection is determined in an early stage based on library properties. After prepurification screening, retention time, mass flag, and parameters describing chromatographic behavior for each compound are exported into a software tool developed in house, where an "optimized" chromatographic method is automatically generated based on these analytical results. A direct scale-up study was conducted using commercially available compounds in an effort to investigate the advantages and limitations of the above process.

*Keywords:* combinatorial chemistry, liquid chromatography/mass spectroscopy, method development, prep chromatography
*Application code:* drug discovery
*Methodology code:* liquid chromatography/mass spectrometry

### HIGH-THROUGHPUT SCREENING FOR CHIRAL SELECTIVITY WITH A NEW EIGHT-COLUMN PARALLEL SCREENING SYSTEM

***Thomas E. Beesley,[*] Brian He, J. T. Lee, and R. P. W. Scott***

*[*]Advanced Separation Technologies Inc (Astec), 37 Leslie Court, PO Box 297, Whippany, NJ 07981, USA*

The need to evaluate enantiomerically pure drugs has moved to the forefront of the drug discovery process. Given the wide diversity of chiral structures being developed and the high specificity of the chiral separation process it has been important to screen a variety of complementary chiral stationary phases to identify chiral selectivity quickly and efficiently. Macrocyclic glycopeptide phases have played an important role in this process. An instrument has been designed to simultaneously screen six of the glycopeptides plus one derivatized cyclodextrin phase and one polymeric cyclic diamine phase with six sequential mobile phase compositions to cover the vast potential of these phases within this 48 parameter matrix.

Table 2

| Composition | Mobile phase | Mode |
|---|---|---|
| (1) 100/0.2/0.1: | MeOH/HOAc/TEA | Polar ionic |
| (2) 20/80: | MeOH/20 mM $NH_4OAc$ pH 5.0 | Reveresd phase |
| (3) 40/60: | ACN/20mM $NH_4OAc$ pH 5.0 | Reveresd phase |
| (4) 100: | EtOH | Polar organic |
| (5) 40/60: | EtOH/heptane | Normal phase |
| (6) Repeat step (4) | As a wash | XXXX |
| (7) 100: | MeOH | Polar organic |
| (8) Repeat step (5) | As a wash | XXXX |

Columns used (150 × 3.0 mm) are
(1) CHIROBIOTIC V,
(2) CHIROBIOTIC V2,
(3) CHIROBIOTIC T,
(4) CHIROBIOTIC T2,
(5) CHIROBIOTIC TAG,
(6) CHRIOBIOTIC R,
(7) CYCLOBOND I 2000 DNB,
(8) P-CAP/DA.

The mobile phase sequences for the series of phases include two reversed-phase, one polar ionic, two polar organic, and one normal phase compositions. The system uses a two UV lamp construction and eight photo receptor cells. The entire cycle time including conditioning through data acquisition for the eight-column configuration is 300 minutes. Using a variety of compound structures (greater than 100) from published papers on high-throughput sequential screening, the success rate for this system has been in the order of 92%. All chromatograms are stored for future method validation and since selectivity is typically identified in more then one mobile phase condition the results can be applied to different application needs. This presentation will identify the list of structures used in the screen, the analysis of the data output and protocols for optimization of the various mobile phase types.

*Keywords:* chiral, high-throughput chemical analysis, liquid chromatography, pharmaceutical
*Application code:* high-throughput chemical analysis
*Methodology code:* liquid chromatography

### FAST SCREENING OF ZIRCONIA-BASED CHIRAL STATIONARY PHASES FOR CHIRAL SEPARATIONS

***Bingwen Yan,[*] Peter W. Carr, Thomas R. Hoye, and Clayton V. McNeff***

*[*]ZirChrom Separations, Inc, 617 Pierce Street, Anoka, MN 55303, USA*

The separation of enantiomers of active pharmaceutical compounds and their synthetic intermediates has become increasingly important for drug development in the pharmaceutical industry. The synthesis and use of a new class of porous zirconia chiral stationary phase for HPLC is described in this work for fast chiral separation screening. A general method for the flexible attachment of brush-type (ie, Pirkle-type) chiral stationary phases is described. The general method involves two main steps: (1) attach an appropriate anchor group to the zirconia surface through a Lewis acid-base reaction and (2) covalently attach the desired CSP to the anchor group using standard EEDQ amide bond formation chemistry. Here we report the synthesis of ten different chiral stationary phases on porous zirconia using three different Lewis base anchor groups. The stability of an aminopropylphosphonic acid anchored zirconia-based CSP was studied and found to be the most stable of the three anchors, even in very aggressive (50 mM tetrabutylammonium hydroxide in 100% MeOH mobile phase) conditions. A number of chiral separations are compared on analogous zirconia and commercial silica-based CSPs. Most importantly we demonstrate that the novel zirconia-based columns may be used for doing fast screening of CSPs. The total cycle time, an example CSP screening run, is shown to be 1.3 hours.

*Keywords:* chiral, drugs, HPLC, method development
*Application code:* pharmaceutical
*Methodology code:* liquid chromatography

### THE APPLICATION OF MS/MS-DIRECTED PURIFICATION TO THE IDENTIFICATION OF DRUG METABOLITES IN BIOLOGICAL FLUIDS

***Robert Plumb,* Ronan Cleary, Paul M. Lefebvre, and Warren Potts***

**Waters Corporation, 34 Maple Street, Milford, MA 01757, USA*

The identification of drug metabolites following animal or human volunteer studies is essential to the drug discovery and development process and regulatory submissions. Traditionally this has been achieved by the use of liquid chromatography or gas chromatography coupled to mass spectrometry (I. M. Ismail and G. J. Dear, *Xenobiotica*, vol. 29, no. 9, pp. 957–967, 1999, and Dear GJ, Mallett DN, Plumb RS, *LC-GC Europe*, vol. 14, no. 10, pp. 616–624, 2002). The use of MS-directed auto-purification, using semi-preparative scale columns (typically 20 mm id), is now common place within the pharmaceutical industry, especially to support lead candidate purification. This approach has also been applied to the isolation of drug metabolites with some success (R. S. Plumb, J. Ayrton, G. J. Dear, B. C. Sweatman, and I. M. Ismail, "Rapid Communications in Mass Spectrometry," vol. 13, no. 10, pp. 845–854, 1999). The extra sensitivity and selectivity of MS/MS mass spectrometry should allow for the more precise selection of drug metabolites, and the use of neutral loss and precursor ion scanning detection modes will facilitate the collection of drug metabolites without the need for prior knowledge of compound metabolism.

In this paper we will show how tandem quadrupole mass spectrometry has been employed with both analytical and semi-preparative scale chromatography for the isolation of the metabolites of common pharmaceuticals, from urine. The application of MRM, neutral loss, and precursor ion scanning will be demonstrated. We will show how this approach results in extremely pure metabolite fractions. We will also demonstrate how the use of MS/MS-directed purification facilitates the combination of samples from several chromatographic runs.

*Keywords:* drug discovery, isolation/purification, liquid chromatography/mass spectroscopy, prep chromatography
*Application code:* drug discovery
*Methodology code:* liquid chromatography/mass spectrometry

### FAST ANALYSIS OF ACTIVE PHARMACEUTICAL INGREDIENTS AND THEIR PRIMARY DEGRADANTS USING NANO-LC

***Todd D. Maloney,* Peter D. Angus, and Gregory K. Webster***

**Pfizer, Inc, Global Research and Development, 2800 Plymouth Road, Ann Arbor, MI 48105, USA*

An increasing trend in pharmaceutical analysis is the demand for analytical separations yielding more information in a shorter period of time. Techniques such as parallel and multidimensional HPLC along with monolithic columns have met these demands to some extent, but the challenge continues to be combining speed of analysis with efficiency. Nano-LC is a technique often overlooked for these challenges. With capillary columns and nanoliter flow rates, Nano-LC not only provides fast highly efficient separations, but also enables the use of less sample and solvent, and provides simple compatibility with MS detection. Nano-LC is an ideal technique for the analysis of pharmaceuticals in early development where detection and identification of trace impurities and degradants can be realized with the increased mass sensitivity and speed of separation achieved utilizing short capillary columns.

We have implemented Nano-LC as a fast and efficient means of screening pharmaceutical compounds for purity. Samples submitted to purposeful degradation were assayed via Nano-LC to assess their physical and chemical stability. Investigations into impurity identification as well as merits of the analytical instrumentation will be highlighted in this presentation.

*Keywords:* liquid chromatography, pharmaceutical
*Application code:* pharmaceutical
*Methodology code:* liquid chromatography

### RAPID CATALYTIC FORMATION OF NITROXIDES OF PHARMACEUTICAL DRUG MOLECULES FOR HPLC METHOD DEVELOPMENT

***Li Li* and Eric D. Nelson***

**Merck & Co, Inc, 770 Sumneytown Pike, PO Box 4, Wp14-2E, West Point, PA 19486, USA*

A catalytic oxidation system-H2O2/methyltrioxo rhenium (VII) (MTO) was evaluated for the rapid formation of pyridine or purine nitroxides. The system has shown a unique ability to efficiently and selectively generate the nitroxides of pharmaceutical drug molecules under mild conditions. The reaction procedure is very simple and the reaction products are compatible with reversed-phase HPLC analysis. Studies aiming at optimizing the reaction conditions showed significant effects of the reagent concentration, reaction temperature, addition order of the reagents, and solvent composition on the reaction rate. The kinetic studies and some other experimental observations suggested that the key intermediate species formed between $H_2O_2$ and MTO is responsible for the rapid formation of nitroxides, consistent with previous literature reports. The catalytic system was successfully applied to MK-0557 and other Merck compounds, and rapid and selective formation of nitroxides was demonstrated. The system was shown to be a good facilitator for developing stability-indicating HPLC assay methods and ensuring methods are selective for the potential nitroxide degradants.

*Keywords:* drugs, HPLC, pharmaceutical
*Application code:* pharmaceutical
*Methodology code:* liquid chromatography

### ON-LINE ELECTROCHEMISTRY/MASS SPECTROMETRY FOR STUDIES OF CLOZAPINE OXIDATION

***Suze M. van Leeuwen,* Uwe Karst, and Jean-Michel Kauffmann***

**University of Twente, PO Box 217, Enschede 7500 Ae, The Netherlands*

Since a few years, electrochemistry has been explored for its capability to mimic biological metabolism, thus increasing its importance in pharmaceutical chemistry. On-line electrochemistry/mass spectrometry (EC/MS) coupling has been set up to directly identify the electrochemically generated species. Clozapine is an antipsychotic drug with advantages over other antipsychotic drugs for its lack of certain side effects in the body. However, clozapine has been associated with acute agranulocytosis, a severe blood disease. Studies are going on to identify the metabolic pathways of clozapine, indicating a free clozapine radical intermediate to form conjugates. EC/MS has been chosen to give a clearer insight in these and either confirm or contradict hypotheses based on other studies. Solutions of clozapine are transferred to the electrochemical cell in an acetonitrile/ammonium formate buffer (pH3) solvent mixture. Different potentials are applied to the electrochemical cell, and the eluting products are detected by an ion trap MS with electrospray ionization (ESI) source. At increasing cell potential, more oxidation products are seen. First, a species is observed at two m/z units lower than clozapine. Furthermore, N-desmethylclozapine and clozapine-N-oxide (the two main biological metabolites) are observed. No clozapine radical cation is observed. For better identification of the formed species, on-line liquid chromatography (LC) was applied after electrochemistry and prior to MS detection. However, not all electrochemically obtained species are stable enough to overcome the time needed to elute them from the chromatographic column. Tandem MS experiments are carried out for further product identification. Phase II metabolism is studied for the formation of clozapine-glutathione conjugates and experiments at physiological pH are performed.

### ACKNOWLEDGEMENT

Financial support by the "Nederlandse organisatie voor Wetenschappelijk Onderzoek" (NWO) is gratefully acknowledged.

*Keywords:* biopharmaceutical, electrochemistry, liquid chromatography/mass spectroscopy, other hyphenated techniques
*Application code:* pharmaceutical
*Methodology code:* others

### ELECTRONIC NOSE AND TONGUE: INNOVATIVE TOOLS TO SPEED UP FORMULATION DEVELOPMENT

***Vincent Schmitt* and Marielle Garnier***

**Alpha M.O.S. America, 33 North River Street, Hillsborough, NJ 08844, USA*

Good tasting products is a more and more crucial focus for pharmaceutical companies. Whether testing product in phase 1 trials, development of oral delivery methods, or pediatric formulations, product taste is a significant aspect of a product success. The electronic nose and tongue were developed to rapidly test and assess liquid and solid drugs both qualitatively and quantitatively for odor/taste attributes in the formulation stage of development. Different classes of excipients including sweeteners, flavors, and preservatives are evaluated to assist the researcher in choosing the right excipient/ingredients providing the best tasting formula. Several applications will be detailed including bitterness measurement and masking of drugs for various oral formulations (syrups, oral dispersive, orally disintegrating tablets etc).

The results will cover bitterness quantification, taste masking, and identification of the best tasting formulations in both a static mode (as liquid and suspensions are perceived) and also a dynamic mode to define the evolution of taste (as perceived with oral dispersive delivery systems such as lozenges, fast-dissolve technologies, nasal spray and coated products). The successful identification of best tasting formulations has been compared and validated with excellent correlation between analytical data and sensory panel testing; comparison of these techniques will also be presented.

*Keywords:* analysis, pattern recognition, quality, sensors
*Application code:* pharmaceutical
*Methodology code:* sensors

### MONITORING FORMATION OF MAILLARD REACTION PRODUCTS FROM OVER-THE-COUNTER GLUCOSAMINE DIETARY SUPPLEMENTS BY CAPILLARY ELECTROPHORESIS

***Paula F. Sá,* Joel A. Dain, and Xinfeng Zhang***

**ANVISA, Sepn 515 Bloco B, Ed Omega, 2nd Andar, Brasília DF 70770-502, Brazil*

Monitoring the formation of Maillard reaction products (MRP) in over-the-counter (OTC) glucosamine dietary supplements. Solutions prepared with seven glucosamine dietary supplements and a reference glucosamine hydrochloride were prepared in phosphate saline buffer (PBS) and incubated for 48 hours at 37° C. Formation of MRP from the autocondensation reaction of glucosamine was monitored by capillary electrophoresis as described. The nature of the salt used in the formulation clearly affects the MRP profile, which is also affected by the presence of chondroitin and vitamin C. Therapeutic effects of glucosamine preparations may be correlated to some of the MRP products formed during the autocondensation of glucosamine. However, the long-term effect of these compounds in the organism must be evaluated.

*Keywords:* analysis, biopharmaceutical, capillary electrophoresis, monitoring
*Application code:* pharmaceutical
*Methodology code:* capillary electrophoresis

### PREPARATIVE HPLC: FACTORS AND PARAMETERS THAT DIRECTLY AFFECT RECOVERY OF COLLECTED FRACTIONS

***Joan M. Stevens,* Alan Hamstra, Luke Roenneburg, and Gary Scharrer***

**Gilson, Inc, 3000 W Beltline Highway, Middleton, WI 53562, USA*

Purification of synthetic, natural, and biological compounds in any quantity usually requires the use of preparative HPLC. Collecting fractions from the preparative HPLC is the common approach to achieving the purified compound(s) of interest. Collection of fractions however is not trivial and is affected by many conditions and parameters within preparative HPLC. In a perfect world chromatographers would be able to optimize for each compound needing to be purified; however this is not the case. The present study will examine a multitude of factors and parameters that directly affect fraction collection recovery. Some of the parameters to be discussed are column performance, mobile phase constituents, detector setting, and collection parameters such as collection via level versus slope and delay volumes. The data presented will offer guidelines for all chromatographers purifying compounds by preparative HPLC and can be implemented into existing systems.

*Keywords:* HPLC, optimization, prep chromatography, sample preparation
*Application code:* pharmaceutical
*Methodology code:* liquid chromatography

### FAST PCLC: A WIDELY APPLICABLE TECHNIQUE FOR LABORATORY-SCALE PREPARATIVE SEPARATIONS

***Joan M. Stevens,* Luke Roenneburg, and Gary Scharrer***

**Gilson, Inc, 3000 W Beltline Highway, Middleton, WI 53562, USA*

Preparative HPLC is recognized as a prime method for attaining pure compounds from complex mixtures. It is often proposed that prep HPLC can be satisfactorily performed with low efficiency, large-diameter columns, usually operating under overload conditions. This technique, called FAST PCLC (preparative column liquid chromatography), reduces analysis time, so throughput is greater. For example, a 3-micron packed column can handle a throughput of 1 gram in a fraction of the time required on a 15+ micron column. In addition, lower capacity factors mean a higher concentration of solute in the collected fractions, which in turn leads to easier processing of eluted compounds. Solvent consumption is reduced, contributing to a more economical procedure. Short re-equilibration times make the system fully compatible with gradient elution. Complete preparative separations can be developed on the same system with the same column. Having completed method development, the system can operate unattended.

Employing repetitive cycles instead of scale-up substantially reduces labor and column costs. The system can also be used to immediately check the purity of collected fractions and is compatible with analytical-scale HPLC. Implementing FAST PCLC is a high-efficiency approach to preparative HPLC. Data implementing this technique will be presented.

*Keywords:* automation, combinatorial chemistry, HPLC, prep chromatography
*Application code:* others
*Methodology code:* liquid chromatography

### BIOSENSING WITH NANOTUBE MEMBRANES

***Charles R. Martin***

*University of Florida, 218 Chemistry Lab Building, PO Box 117200/218 Leigh Hall, Gainesville, FL 32611, USA*

We have been investigating a versatile method for preparing nanomaterials called template synthesis. This method entails using the pores in a microporous membrane or other solid as templates to prepare, typically, nanowires and hollow nanotubes. Nanostructures of this type composed of metals, semiconductors, other inorganic materials, polymers, and carbons have been prepared. We have shown that membranes containing gold nanotubes with inside diameters of molecular dimensions can be prepared via the template method. These Au nanotube membranes can form the basis for new types of highly sensitive chemicals and biosensors. Immobilization of biochemical molecular recognition agents (eg, proteins, DNA) within the nanotubes is being investigated as a general method for making highly selective biosensors of this type. Nanotubes that are conically shaped are of particular interest.

*Keywords:* nanotechnology
*Application code:* nanotechnology
*Methodology code:* sensors

### MOLECULAR SENSING USING AN ON-CHIP ARTIFICIAL PORE

***Lydia L. Sohn***

*University of California at Berkeley, 5118 Etcheverry Hall, Berkeley, CA 94720, USA*

Biological processes often depend on sparse interactions involving as little as a single molecule. One example is "sensory processing" or the transduction of stimuli such as odor, light, and sound into a cellular response. Such processing centers on transmembrane pore proteins or ion channels are so finely tuned that they can open or close in response to only one molecule. This level of sensitivity is truly remarkable and serves as inspiration for developing artificial nanopores for molecular sensing. In this talk, I will describe a fundamentally different artificial pore my group has developed within an integrated microfluidic chip. Our pore spans the length scales of "micro" to "nano" and is easily and reliably fabricated in either quartz substrates or polydimethylsiloxane (PDMS). I will show our pore's ability to detect the binding of unlabeled antibody-antigen pairs as well as the ability to sense single molecules of unlabeled lambda-phage DNA. Finally, I will discuss current and future applications of our on-chip artificial pore, including immunosensing and multidimensional screening.

*Keywords:* nanotechnology
*Application code:* bioanalytical
*Methodology code:* sensors

### CHALLENGES IN POLYMER CHARACTERIZATION USING MASS SPECTROMETRY

***Charles N. McEwen***

*DuPont, Dupont Experimental Station, PO Box 80228, Wilmington, DE 19880-0228, USA*

Mass spectrometry is a powerful tool for determining the chemical composition of many polymeric materials. Pyrolysis in combination with mass spectrometry, for example, has long been used to thermally decompose and vaporize polymers to enable study by mass spectrometry. On the other hand, newer ionization methods such as matrix-assisted laser desorption/ionization (MALDI) provide a means to directly observe intact molecular ions of polymeric molecules, frequently without the complications of fragmentation. A mass spectrum of the molecular ions of all of the oligomeric components of a polymer can be used to infer the composition of homopolymers, copolymers and sometimes even terpolymers as well as end-group compositions. Any change in the chemical composition of a polymer that alters the mass of its molecular components can potentially be identified using mass spectrometry. In addition to chemical composition information, MALDI mass spectrometry produces a mass/intensity distribution that can potentially be used to determine various moments of molecular weight distributions. Thus, number and weight-average molecular weight distribution information is contained in the same data-set used for structural determination. Changes in composition during a reaction as a function of time can be used in kinetic studies. However, a number of challenges remain in characterizing polymers by mass spectrometry. These include inaccurate molecular weight distribution information for polymers with polydispersity greater than about 1.2, ionization difficulty for polymers lacking suitable sites for ionization, inaccurate or no data from insoluble polymers and those with poor solubility, and fragmentation of labile polymers. Research into methods dealing with these challenges will be presented.

*Keywords:* mass spectrometry, polymers and plastics
*Application code:* polymers and plastics
*Methodology code:* mass spectrometry

### BIOMARKERS FOR CHARACTERIZATION OF GRAM-NEGATIVE AND GRAM-POSITIVE BACTERIA BY COUPLING PYROLYSIS GAS CHROMATOGRAPHY TO ION MOBILITY SPECTROMETRY

***Satendra Prasad,[*] Gary A. Eiceman, and Hartwig Schmidt***

*[*]Department of Chemistry & Biochemistry, New Mexico State University, 1175 North Horseshoe Drive, Las Cruces, NM 88003-8001, USA*

Conventional Gram staining bacterial identification is very often tedious and time consuming. Rapid chemical characterization of bacterial cells using a pyrolysis gas chromatograph coupled to a mobility spectrometer has been demonstrated. Gram-negative cells (*E coli, K pneumoniae, P vulgaris*) and Gram-positive cells (*B megaterium, M luteus*) were pyrolyzed on a platinum ribbon at 650°C. Volatile and semivolatile pyrolysates were separated on a nonpolar DPB-5 column and detected using a mobility spectrometer equipped with a 63 Ni ion source. Chemical standards of known pyrolysates were used to optimize experimental conditions such as detector temperature, electric field, and shutter opening time for bacterial characterization. Qualitative and quantitative chemical information was extracted on basis of chromatographic retention time, orthogonal drift time and intensity. Such characteristic signatures were determined for each strain of bacteria and formed the basis for rapid bacterial characterization and identification as the total chemical profile varied for each species topographically. Detection limits were determined to be in the lower nanogram range. Unknown pyrolysis products of bacterial cells were identified by comparison with chemical standards detected under similar conditions. Potential biomarkers within the topographical profile such as 1-tridecene, dodecanal, 2-tridecanone, and tetradecanoic acid (myristic acid) were observed characteristically in gram-negative cells such as *E coli, K pneumoniae,* and *P vulgaris* and were regarded as breakdown products from characteristic Gram-negative cell biopolymers such as lipopolysaccharides. Myristic acid was only observed in *E coli*. However, in Gram-positive cells of *B megaterium* and *M luteus*, these chemicals were noticeably absent and were thus considered as biomarkers for Gram-negative bacteria.

*Keywords:* bioanalytical, gas chromatography
*Application code:* bioanalytical
*Methodology code:* gas chromatography

### PYROLYSIS GAS CHROMATOGRAPHY WITH MICROFABRICATED DIFFERENTIAL MOBILITY SPECTROMETRY: A POWERFUL COMBINATION FOR A FAST AND SENSITIVE CHARACTERIZATION OF BACTERIA

***Hartwig Schmidt** and Gary A. Eiceman***

**Department of Chemistry & Biochemistry, New Mexico State University, 1175 North Horseshoe Drive, Las Cruces, NM 88003-8001, USA*

The chemical constituents of cell walls of bacteria can be characterized through instrumental methods of chemical analysis which may replace, in some instances, the traditional labor-intensive and time-consuming methods of bacterial culturing, staining, and microscopic analysis. A combination of pyrolysis-gas chromatography (py-GC) and differential mobility spectrometry (DMS) with microfabricated drift tubes was used to chemically characterize bacteria. Chemical information was extracted out of three-dimensional plots on basis of ion intensity, compensation voltage from differential mobility spectra, and chromatographic retention time. The DMS analyzer provided chemical information for positive and negative ions simultaneously from chemical reactions between pyrolysis products in the GC effluent and reactant ions of $H^+(H_2O)_n$ and $O_2^-(H_2O)_n$ in air at ambient pressure.

Chemical standards were prepared and included substances which are formed in the pyrolysis of bacteria. They showed favorable matches with plots from py-GC/DMS analysis and were supported by py-GC/MS results. Comparisons between different Gram-negative bacteria such as *E coli, K pneumoniae,* and *P vulgaris* and between Gram-positive and Gram-negative bacteria helped to identify components which can be regarded as biomarkers due to pyrolysis products of bacterial cells. Amongst other substances, 2-tridecanone, dodecanal, and tetradecanoic acid were found to be characteristic of Gram-negative bacteria evident in positive and partly negative ions and were not observed with Gram-positive bacteria. The minimum number of cell-forming units detected was $\sim$ 10 000 though detection limits and resolution could be further improved by varying the magnitude of the separation voltage in the differential mobility spectrometer as well as by minimizing the distance between pyroprobe and GC-column.

*Keywords:* bioanalytical, capillary GC, gas chromatography
*Application code:* bioanalytical
*Methodology code:* gas chromatography

### A FULLY AUTOMATED PURGE-AND-TRAP GC ANALYZER FOR MONITORING TRIHALOMETHANES IN DRINKING WATER DISTRIBUTION SYSTEMS

***Michael A. Brown** and Gary L. Emmert***

**Department of Chemistry, The University of Memphis, Smith Chemistry Building, Room 213, Memphis, TN 38152, USA*

For over a hundred years, chlorination has been the most widely used form of water disinfection in the world; however, halogenated disinfection by-products (DBPs) like trihalomethanes (THMs) are formed during this process. The THM4, which include chloroform, bromodichloromethane, dibromochloromethane, and bromoform are possible carcinogens; therefore, the USEPA has set a maximum contaminant level (MCL) for these compounds in drinking water at 0.080 mg/L. Traditional methods for THM4 analysis, even though very sensitive and reliable, require extensive sample preparation steps or expensive equipment to perform successfully. In addition, these methods cannot easily be adapted for on-line monitoring of drinking water distribution systems. Collaboration with SRI Instruments has produced an on-line purge-and-trap GC with either a DELCD or ECD used for detection. This instrument is fully automated, portable, easily adaptable to on-line sampling, and costs in the $20 000 range. By using an internal standard injection configuration, calibrating for matrix effects and GC changes is greatly simplified. The THM4 with added internal standard(s) present within a water sample are extracted using a membrane sampling cell and then are sent to traps that are connected by a 10-port injection valve. The traps are heated and the analytes are flowed to a capillary column. After being separated by the column, the THM4 are detected by either a DELCD or ECD, which are both very sensitive to halogenated compounds. This analyzer is fully automated and only requires a typical water tap to conduct on-line monitoring of THM4 concentrations in drinking water. By using a peristaltic pump to draw sample through the sampling cell, samples that are collected off site can also be analyzed. A preliminary monitoring study for Memphis drinking water was conducted using this analyzer and USEPA METHOD 502.2. A comparison of these results will be discussed. MDL, accuracy, and precision studies will also be presented.

*Keywords:* environmental/water, gas chromatography, purge and trap, sample introduction
*Application code:* environmental
*Methodology code:* gas chromatography

### A MICROWAVE-INDUCED PLASMA COUPLED TO A TIME-OF-FLIGHT MASS ANALYZER FOR RAPID ELEMENTAL SPECIATION BY GAS CHROMATOGRAPHY

***Steven J. Ray* and Gary M. Hieftje***

**Department of Chemistry, Indiana University, Bloomington, IN 47405, USA*

The speciation of organometallic compounds of environmental and toxicological interest is typically achieved by hybrid analytical techniques consisting of a chromatographic separation followed by element-selective detection. Atomic spectrometric detection strategies offer high-sensitivity coverage that extends across nearly the entire periodic table over a wide dynamic range, suffer from a minimum of matrix effects, and are tolerant of nonideal separations. When such a method employs a time-of-flight mass analyzer (TOFMS), comprehensive elemental analysis of eluents from chromatographic separations can be achieved with high temporal resolution and without skewing of the mass spectrum that can occur when sequentially scanning mass analyzers are employed.

Here, a novel microwave-induced plasma is investigated as an ionization source for elemental speciation analysis by gas chromatography. The microwave plasma torch (MPT) is an unusual microwave resonator that permits a plasma to be formed without any enclosing support structure, thereby minimizing fouling or memory effects. Like many microwave plasmas, the MPT is also capable of operating with a variety of plasma gases, including helium, which permits the determination of halogens with high sensitivity. Further, as the entire atomic mass spectrum is recorded simultaneously with a TOFMS, all elements of which an eluting molecule is composed can be observed simultaneously, and their molar ratios employed to gain further chemical information or to deconvolute coeluting compounds. Experimental details and current analytical performance of this MPT-TOFMS system will be reported, and future initiatives considered.

*Keywords:* atomic spectroscopy, elemental mass spectrometry, gas chromatography/mass spectrometry, time-of-flight MS
*Application code:* environmental
*Methodology code:* atomic spectroscopy/elemental analysis

### OPTICAL OPTIMIZATION IN THE USE OF ARRAY DETECTORS FOR ICP-OES

***Michael P. Wassall* and Charles V. Perkins***

**Thermo Electron Corporation, Solaar House, PO Box 207, Cambridge, CB5 8BZ, UK*

ICP-OES has become the leading analytical technique of choice for large sample throughput elemental analysis laboratories. Two-dimensional solid-state array detectors have been used in ICP-OES for several years. They have enabled simultaneous elemental analysis to provide fast throughput in remarkably compact instruments. Recent improvements in these detectors now dictate further improvements in the spectrometer optical systems in order to fully realize their potential.

This paper will provide the theoretical background to such optical optimization, outline the improved characteristics of these array detectors, and include experimental, confirmatory data of improvements in detection capability, speed of analysis, and practical improvements in usage.

In the case of the detector, emphasis will be placed on the physical improvements to pixel dimensions, layout, and signal processing infrastructure. The optical improvements will be described by choice of polychromator layout and mathematical modeling of the image characteristics such as quality, stability, and aberration correction.

Finally, by comparison with existing data, the paper will present examples of improvement in real analysis of environmental samples.

*Keywords:* atomic emission spectroscopy, charge transfer devices (CID CCD), ICP, plasma emission (ICP/MIP/DCP/etc)
*Application code:* environmental
*Methodology code:* atomic spectroscopy/elemental analysis

### INVESTIGATION OF STATISTICS FOR IMPROVING THE DISCRIMINATING POWER OF LASER-INDUCED BREAKDOWN SPECTROSCOPY

***Chase A. Munson,* Frank C. DeLucia, Kevin L. McNesby, Andrzej W. Miziolek, and Thuvan N. Piehler***

**US Army Research Laboratory, 2800 Powder Mill Road, Adelphi, MD 21005-5069, USA*

Continuing threats from biological and chemical terrorism have increased the demand for accurate and rapid determination of chemical and biological species. Ideal detection techniques for these species are characterized by little or no sample preparation, negligible use of reagents and consumables, and rapid characterization against a spectral database. One technique exhibiting promise for this application is laser-induced breakdown spectroscopy (LIBS). LIBS provides "real-time" total elemental detection without the need for sample preparation. In addition, LIBS systems are rugged and well suited for field use. However, the success of LIBS as a field-portable point detector for chemical and biological agents requires the development and optimization of stable sample libraries and statistical methods for rapidly analyzing spectra. In this work, we investigate the efficiency of test sample identification by applying various statistical methods and data pretreatments to LIBS spectra. These results are providing insight into improving spectral library matching of nearly indistinguishable samples, such as species of biological and geological origin (eg, anthrax surrogates, pollen, and dust).

*Keywords:* atomic spectroscopy, laser, materials characterization
*Application code:* homeland security/forensics
*Methodology code:* atomic spectroscopy/elemental analysis

### IDENTIFICATION OF THE POLYMORPHS OF SULFATHIAZOLE USING TERAHERTZ PULSED SPECTROSCOPY

***Philip F. Taday,* David A. Newnham, Yaochun Shen, and Clare J. Strachan***

**TeraView Limited, 302/4 Cambridge Science Park, Milton Road, Cambridge Cb4 0Wg, UK*

Terahertz (THz) spectroscopy involves radiation in the THz region of the electromagnetic spectrum (3.3 to 100 $cm^{-1}$). Terahertz radiation induces crystalline phonon vibrations or H-bonding stretches and torsion vibrations in molecular systems. The ability of terahertz pulsed spectroscopy to probe crystalline phonon vibrations makes it an ideal technique to investigate polymorphism and crystallinity.

Sulfathiazole (STZ) is a well-known antibiotic agent whose crystallographic properties have been studied thoroughly. There are five different known polymorphs of STZ making it an ideal case study for a new analytical technique. Measurements were made using a TPI Spectra 1000 spectrometer.

Comparison of the spectra of the five forms of STZ shows pronounced spectral differences. The differences between polymorphs III, IV, and V are clear. These polymorphs are difficult to distinguish by other more conventional techniques such as mid-infrared and NMR spectroscopies. It is also noted that the peaks from polymorph III lay between those of polymorphs IV and V. However the terahertz absorption spectrum of polymorph III is nearer to polymorph IV than polymorph V; this is the opposite of X-ray power diffraction. The spectrum of polymorph I is featureless when compared with the other four polymorphs. This maybe due to the fact that polymorph I is composed of two interlocked lattices, whereas all the other are either simple layer structures (polymorphs III, IV, and V) or a simple three-dimensional lattice (polymorph II). In these studies we have looked at the temperature-dependent spectra of the polymorphs allowing to increase understanding of band assignments. These pharmaceutical examples show that different crystalline forms give rise to remarkably different spectra in the terahertz region. This study shows that terahertz spectroscopy provides an excellent means to quantitatively characterize crystallinity of pharmaceutical compounds.

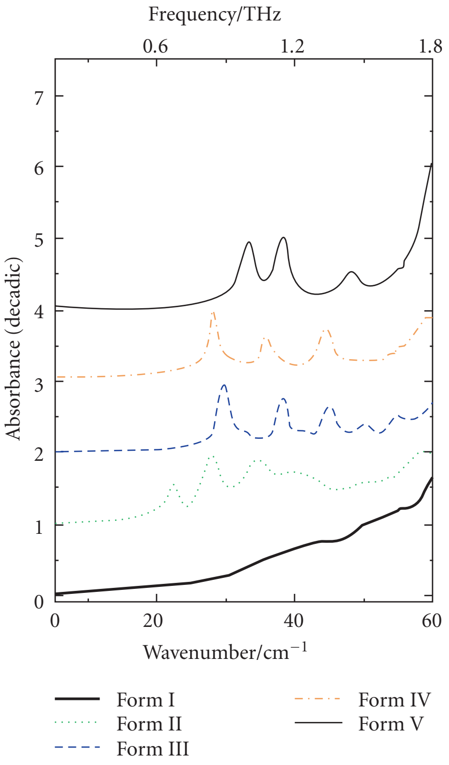

Figure 6

*Keywords:* infrared and Raman, pharmaceutical, spectroscopy
*Application code:* pharmaceutical
*Methodology code:* vibrational spectroscopy

### ACHIEVING AND MAINTAINING REGULATORY COMPLIANCE THROUGH DEPLOYMENT OF ENTERPRISE LIMS

***Edward Ingalls***

*Thermo Electron Corporation, Informatics and Services Group, 18 Commerce Way, Woburn, MA 01801, USA*

Regulatory compliance for life science companies requires adherence to all government regulations for the development and manufacture of safe and effective products including drugs and medical devices. Meeting compliance regulations is mandatory. Understanding that compliance is a multifaceted ongoing process that can be time consuming and expensive encourages the use of automated systems such as LIMS to lessen the burden on laboratory staff. Many global organizations are establishing corporate IT standards across all their labs for increased productivity and cost efficiencies. Deployment of a LIMS that is designed to work with enterprise processes and systems eliminates compliance bottlenecks and significantly reduces resources needed for compliance-related laboratory operations. Tasks such as data acquisition, data review, supervisor approval, audits, investigations, and releases can be dramatically simplified while reducing or eliminating regulatory paperwork. Additional compliance concerns such as security, data integrity, traceability, audit trails, electronic signatures, and electronic records can all be managed across all of your laboratories.

*Keywords:* lab management, laboratory informatics, LIMS, validation
*Application code:* regulatory
*Methodology code:* laboratory informatics

### LIMS AND THE CONTENT REGULATORY COMPLIANCE LANDSCAPE

***Christine Paszko*** *[*] **and Manoucher Shahamat***

*[*]Accelerated Technology Laboratories, Inc, 496 Holly Grove School Road, West End, NC 27376, USA*

As analytical instrumentation becomes more sophisticated and able to generate increasing volumes of data, LIMS have evolved to better manage, analyze, and control this information, with hyperlinks to chromatographs, on-line SOPs, and the generation of pdf documents. In the age of instant access, electronic documentation has come under increasing scrutiny and regulatory control. Several agencies have promulgated legislation to address the integration, access, analysis, sharing, and storage of this electronic data. The compliance landscape is complex and growing, the introduction of regulations such as Sarbanes-Oxley, HIPPA, 21 CFR part 11, Patriot Act, and SEC 17A-4 is forcing organizations across several industries, including laboratories, to address their document management and information processes within them to meet regulatory requirements and to avoid penalties which are inherent in the regulations. Technology is playing a key role in strategic compliance initiates to ensure sustainability of processes, mitigate risk, and control costs. This presentation will discuss the various regulatory requirements and their impact on the laboratory as laboratories moving toward reducing the amount of paper-based documentation toward an electronic document capture and storage.

*Keywords:* computers, data analysis, data base, LIMS
*Application code:* regulatory
*Methodology code:* others

### ESTABLISHMENT OF ELECTRONIC REPORTING ELECTRONIC RECORDS-PROPOSED RULE CROMERRR AND LIMS

***Steve Rayburn*** *[*] **and Manoucher Shahamat***

*[*]Accelerated Technology Laboratories, Inc, 496 Holly Grove School Road, West End, NC 27376, USA*

Currently there are several federal rules covering electronic records and/or electronic reporting. These include, but are not limited to, 30 CFR part 210, 36 CFR part 1234, 40 CFR part 11, and 40 CFR parts 3, 51, and so forth. This last rule, also known as cross-media electronic reporting and record-keeping rule (CROMERRR), has had a start and stop life thus far. However, the potential is there for this rule to be finalized and promulgated during 2005 or 2006. In light of this, laboratories currently using laboratory information management systems (LIMS), and LIMS developers should take this new rule, and the others, under review to be sure the LIMS will be able to meet the requirements set by the rule makers. This presentation will review the general requirements of CROMERRR (and some of the other more well known rules) and discuss the philosophy behind them. Each requirement will be discussed in the context of the specific rule it comes from. Some of the major points to be discussed include making electronic records as reliable as written records, ensuring the traceability of any electronic record, and ensuring the legal defensibility of electronic records. In addition, the presentation will compare these requirements with current LIMS technologies and show what types of solutions these technologies provide to address the requirements of CROMERRR. These technology solutions include both software as well as hardware.

*Keywords:* lab management, laboratory informatics, LIMS, software
*Application code:* regulatory
*Methodology code:* laboratory informatics

### AUTOMATIC NOTIFICATION AND ITS ROLE IN COMPLIANCE MONITORING REQUIREMENTS

***Steve Rayburn*** *[*] **and Anson Ellstrom***

*[*]Accelerated Technology Laboratories, Inc, 496 Holly Grove School Road, West End, NC 27376, USA*

Compliance monitoring can be mandated from either within an organization or from an external agency. Most laboratory personnel are familiar with external agency compliance monitoring requirements. They come from various governmental agencies such as the USEPA, FDA, and others. These agencies administer different programs that are mandated by laws such as the Clean Water Act, Clean Air Act, Safe Drinking Water Act, CFSAN, and others. Other external agencies include clients mandating that materials meet certain specifications such as USP/NF, ASTM, or other industry standards. Internal monitoring requirements could come from quality management systems (QMS), from manufacturing requirements or any other internally generated specification requiring monitoring. Monitoring requirements often go hand in hand with reporting requirements. If the reporting of the results of monitoring testing is not complete, a "reporting violation" may occur. These can lead to regulatory issues, loss of clients, and other "penalties" for the laboratory or laboratory client. This presentation will discuss various types of reporting challenges in a variety of situations including regulatory and business. Solutions to each of the problems will be

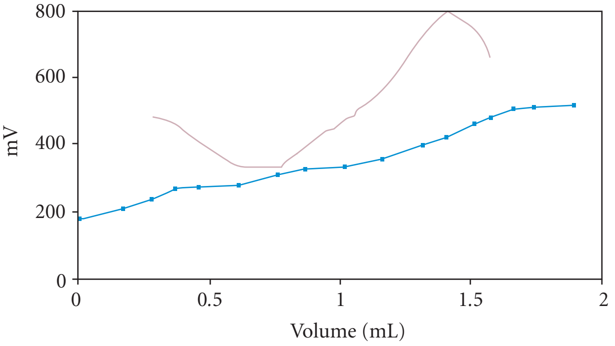

FIGURE 7

presented from available technology found in a computer-based laboratory information management system (LIMS). These technologies will be discussed in relation to how they provide solutions to the reporting challenges defined in this presentation.

*Keywords:* laboratory automation, LIMS, monitoring
*Application code:* regulatory
*Methodology code:* laboratory informatics

### PUBLISHING ANALYTICAL INSTRUMENT DATA OVER NETWORKS WITH ANIML

***Peter J. Linstrom***

*National Institute of Standards and Technology, 100 Bureau Drive, Stop 8380, Gaithersburg, MD 20899, USA*

ASTM Subcommittee E13.15 is developing analytical information Markup language (AnIML) for the storage of analytical instrument data. This language will be able to store a wide range of data types reflecting complex experimental designs. In order for AnIML to be useful, convenient tools for viewing data contained in AnIML files will be required. This talk will discuss approaches for building tools which facilitate the publication of data from AnIML files with web servers. Such systems are appropriate for publishing data globally over the Internet or locally in an intranet. There are several advantages to publishing analytical instrument data on a web server. The most obvious is that the infrastructure for such systems is already in place; the networks and client software (web browsers) required are ubiquitous. Other advantages include the ease of maintaining server-side software and the ability to leverage existing web-based capabilities (eg, search engines and access controls).

This talk will focus on strategies for publishing textural and graphical data using web servers. It will illustrate how the modular nature of the AnIML language aids in these efforts.

*Keywords:* data analysis, laboratory informatics, on-line
*Application code:* general interest
*Methodology code:* laboratory informatics

### MULTIVARIATE TECHNIQUES IN THE OPTIMIZATION OF METHOD FOR DIRECT DETERMINATION OF COPPER IN OIL CONDENSATE USING GRAPHITE FURNACE ATOMIC ABSORPTION SPECTROMETRY

***Walter Nei L. Santos,* Fabio S. Dias, Marcelo S. Fernandes, Sergio Luis C. Ferrreira, and Marcio V. Rebouças***

**Instituto De Química, Campus De Ondina, Universidade Federal da Bahia, Salvador, Bahia 40170-290, Brazil*

In the present paper a method is proposed for direct determination of copper in petroleum condensate using microemulsions and graphite furnace atomic absorption spectrometry (GF AAS). The optimization process was performed in two steps. Firstly, a full two-level factorial design was carried out involving the variables: pyrolysis time, pyrolysis temperature, and atomization temperature. This design demonstrated that in the levels studied the factors pyrolysis and atomization temperature are significant. Considering that, a three-level full factorial design was performed in order to determine the critical experimental conditions for these variables. Using the conditions established in the optimization step, that is, a pyrolysis temperature of 920 °C and an atomization temperature of 2500 °C, this method allowed copper determination at the 327.4-nm line with a detection limit of 0.4 microgram per litre, using 20 microlitre of microemulsion, which corresponds to 1.2 microgram per litre in petroleum condensate.

The characteristic mass was 39 pg. The precision, expressed as relative standard deviation, was 2.0% and 2.8% for copper concentrations of 30 microgram per litre and 5.0 microgram per litre, respectively. The analytical curves should be established using organic standards, prepared as microemulsions. Calibration using inorganic standard cannot be used because the slopes using organic standards and inorganic standards are different. Spike recovery test (105%) of copper added to a petroleum condensate sample proved that this procedure could be applied satisfactorily for a determination in this matrix.

*Keywords:* atomic spectroscopy, chemometrics, optimization, trace analysis
*Application code:* fuels, energy and petrochemical
*Methodology code:* atomic spectroscopy/elemental analysis

### AUTOMATED MEASUREMENT OF BASE NUMBER BY POTENTIOMETRIC TITRATION ASTM D2896

***Tiffany Reid,* Robert V. Menegotto, and Christopher Smith***

**Man-Tech Associates Inc, 2 Admiral Place, Guelph, ON, Canada N1G 4N4*

Base Number or TBN is used to test the quantity of basic components in an oil sample. This test determines the amount of base in a sample dissolved in a solvent mixture.

Base number results are used as a guide in the quality control of lubricating oils. Base additives are added to high-use diesel oils during the manufacturing process. As acids are formed during normal use, the base additives will increase the lifespan of the oil by neutralizing the acids. Therefore, base number will change over time as the base additives are neutralized. Traditionally, the base number is measured by manual titration. This application uses ASTM method D2896 in a fully automated setup. Automated measurement of base number is a simple, effective, accurate, and precise method. It has a large dynamic range, runs smoothly and unattended, and has automated solvent delivery and extraction. This method corresponds to the ASTM method using a well-defined inflection point whenever possible. Versatile software is used for the automation control and data analysis. Quality control charts and custom reports are easily produced, common parameters can easily be adjusted as necessary, and detailed calculations are performed automatically. Dynamic titration control ensures quick and precise titrations, and automated solvent handling provides consistent volume addition plus waste solvent extraction, minimizing the analysts' exposure to hazardous chemicals. Details of the method and statistical results for base number over a range of concentrations using two different solvent matrices will be presented.

*Keywords:* automation, fuels energy petrochemical, petrochemical, titration
*Application code:* fuels, energy and petrochemical
*Methodology code:* chemical methods

### CHARACTERIZATION OF NONVOLATILE ORGANIC COMPOUNDS IN OXIDIZED LANDFILL LEACHATE BY FOURIER TRANSFORM ION CYCLOTRON RESONANCE MASS SPECTROMETRY

***Daniel M. Osborne,* William T. Cooper, Alan G. Marshall, Ryan P. Rodgers, and Tanner M. Schaub***

**Dittmer Laboratory of Chemistry, Florida State University, Tallahassee, FL 32306-4390, USA*

Landfill leachate contains higher concentrations of dissolved organic matter (DOM) than typical domestic wastewater. Many of these organic compounds are resistant to microbial decomposition, but they can be degraded via chemical oxidation techniques such as ozonation to yield products that are bioavailable to microorganisms. Many of the volatile and semivolatile compounds in both raw and ozonated leachate have been identified, but the nonvolatile fraction represents the majority of the DOM and this fraction is largely uncharacterized. Some of the compounds in this fraction are referred to as "humic-like" or "fulvic-like," but there is limited information about their actual composition. Fourier transform ion cyclotron resonance mass spectrometry (FT-ICR MS) provides the high-mass resolving power needed to assign molecular formulas to these unidentified, nonvolatile components. The combination of high-field FT-ICR MS with electrospray ionization (ESI) has proven to be the most useful technique for identifying large organic molecules in natural aquatic environments. However, ESI is highly selective for acidic and basic polar compounds, and ozonated leachate contains additional nonpolar molecules that cannot be observed by this method. Field desorption ionization was thus used in parallel with electrospray to examine both the polar and nonpolar organic compounds in landfill leachate before and after ozonation. Using this combination of ionization techniques and exploiting the ultra-high resolving power and mass accuracy of high-field FT-ICR, we have been able to identify the changes in elemental compositions of individual polar and nonpolar nonvolatile components in leachate after ozonation. This information is currently being used to estimate the biodegradability of these oxidized compounds. This work was supported in part by the NSF National High-Field FT-ICR Mass Spectrometry Facility (CHE 99-09502), Florida State University, and the National High Magnetic Field Laboratory at Tallahassee, Florida.

*Keywords:* electrospray, environmental analysis, ion cyclotron resonance, mass spectrometry
*Application code:* environmental
*Methodology code:* mass spectrometry

### RAPID TOTAL PETROLEUM HYDROCARBON (TPH) ANALYSIS IN SOIL USING FOURIER TRANSFORM INFRARED ATTENUATED TOTAL REFLECTION (FT-IR/ATR)

***John C. Corbett,* David V. Dewey, and Mark L. Norman***

**Smiths Detection, 14 Commerce Drive, Danbury, CT 06810, USA*

Petroleum hydrocarbons (PHCs) are very common soil contaminants. Soil contamination has been a growing concern because it can be a source of groundwater (drinking water) contamination; contaminated soils can reduce the usability of land for development and petroleum residuals can be bound to the soil for years. The sampling methods and the analysis of environmental PHCs are referred to as total petroleum hydrocarbon (TPH) methods. As gross measurements of petroleum contamination, TPH methods simply show that petroleum hydrocarbons are present in the sampled media. Positive TPH results may require action on the part of the land owners, local and state governments, and environmental remediation firms called on to remove or reduce the TPH problem. In most cases, the environmental remediation firms will remove the soil from around the contamination source until the TPH levels are below EPA standard concentration levels. This requires sending samples to a certified laboratory for analysis and waiting one to four days for analytical results to determine whether to continue removing soil. Significant financial expenses are incurred during this procedure because remediation equipment is idle during sample analysis and the cost for analysis determines the sample turnaround time.

Portable FT-IR systems and diamond-attenuated total reflection (ATR) sample interface technology offer excellent opportunities for greater speed and consistency. Small amounts of sample are extracted with a hexane solvent and deposited onto the ATR crystal. A sample spectrum is introduced to a standard calibration dataset and the TPH concentration determined. This method is designed to provide remediation firms with immediate concentration information to determine whether to continue processing soil prior to sending samples to certified laboratories. A discussion of this procedure and the results of laboratory and field validation tests will be presented.

*Keywords:* contamination, environmental, FTIR, instrumentation
*Application code:* environmental
*Methodology code:* vibrational spectroscopy

### MOLECULARLY IMPRINTED POLYMER SENSORS FOR ORGANOPHOSPHORUS COMPOUNDS AND THEIR APPLICATION TO THE DETERMINATION OF ORGANOPHOSPHORUS PESTICIDES

***Sue Y. Bae,* Michael W. Ellzy, and Amanda L. Jenkins***

**US Army, ECBC c/o Geo-Centers, Inc, PO Box 68, APG, MD 21010, USA*

Molecularly imprinted polymers (MIP's) are increasingly being used for selective extraction of biological and environmental samples. They possess high selectivities and affinities and with careful control of the imprinting process these properties may be qualitatively and quantitatively predetermined for a particular analyte of interest, or a class of structural analogues. Unlike their antibody counterparts, the polymers maintain excellent thermal and mechanical stability. The MIP's potentially offer a higher level of sample cleanup than previously obtained using conventional extraction materials. Previous work has provided molecularly imprinted polymers that are selective for the hydrolysis products of organophosphorus species such as the nerve agents sarin and soman (A. L. Jenkins, O. M. Uy, and G. M. Murray, "Polymer-based lanthanide luminescent sensor for detection of the hydrolysis product of the nerve agent Soman in water," *Anal. Chem.*, vol. 71, no. 2, pp. 373–378, 1999). In this study, direct imprinting of organophosphorus compounds such as dimethyl hydrogen phosphonate (DMHP), dicyclohexyl methylphosphonate (DCMP), 1,4-Dithiane (DITH) was used. The resulting DCMP MIP was successfully applied to extract commercially available organophosphorus pesticides such as dichlorvos, diazinon, and phosphamidon. The % recoveries of the organophosphorus compounds were greater than 95% for all cases using GC-MS. Liquid chromatography with electrospray-mass spectrometric detection was also used for the analysis of the organophosphorus compounds. The current DCMP MIP sample can provide an extremely useful tool for extraction and cleanup applications for organophosphorus pesticides in the environment. Limits of detection and interference data will be provided.

*Keywords:* environmental/biological samples, pesticides, polymers and plastics, sensors
*Application code:* environmental
*Methodology code:* sensors

Table 3

| Sample | E-nose response | VOC (mg/m$^3$) | Dy Olf (ODU/m$^3$) |
|---|---|---|---|
| Untreated | 0.56–0.76 | 4.4–6.0 | 6700–10 800 |
| Sample 1 | 0.83 | 5.0–6.1 | 1400–3360 |
| Sample 2 | 0.72–0.76 | 4.8–5.5 | 8000–12 000 |
| Sample 3 | 0.86–0.92 | 5.1–6.1 | 10000–25 000 |
| Sample 4 | 0.78–0.80 | 4.5–5.5 | 8000–13 000 |
| Sample 5 | 0.69–0.72 | 4.8–6.5 | 9000–40 000 |

### OBJECTIVE ODOR MEASUREMENT AND EFFICIENCY

***Louis Vivola,* Eric Chanie, and Vincent Schmitt***

**Alpha M.O.S., 33 North River Street, Hillsborough, NJ 08844, USA*

Offensive odors from industry are a worldwide problem existing for many years. Since its inception in 1970, Environment Protection Agency (EPA) has developed an approach for managing odors in the environment. Important improvements have been made since this time. Actually, more than 40% of the pollution complaints received concern odor. In this context, ability to measure olfactive nuisance with objective information is crucial. Today, it is possible to qualify odor for a known compounds by measuring the concentration of the chemical with traditional techniques as gas chromatography or mass spectrometry. For mixtures of unknown substances, odor emissions are only estimated by a panel of trained human noses using EPA's standard analytical procedure. These methods are expensive and time consuming.

With RQ BOX (electronic nose module), Alpha M.O.S. provides objective odor analysis of atmospheric contaminant in ambient air and from point and surface sources. With an olfactive fingerprint determination coupled to targeted gases detection (as ammonia, hydrogen sulfide, mercaptans) it is now possible to track gas leakage and control deodorizing process efficiency. In this presentation, we will show how the electronic nose module developed by Alpha M.O.S. gave correlated results compared to olfactometry results and VOC data. Discussion will be carried out about the scientific results of such new instrumental fingerprinting techniques for environmental industries.

*Keywords:* environmental air, identification, instrumentation, volatile organic compounds
*Application code:* environmental
*Methodology code:* sensors

### TECHNIQUES FOR IMPLEMENTING HIGH-THROUGHPUT SCREENING (HTS) IN SAMPLE PREPARATION/SAMPLE HANDING APPLICATIONS

***Hall T. Martin***

*National Instruments, 11500 North Mopac Expwy, Austin, TX 78759, USA*

With the advent of HTS screening requirements, the number of samples to prepare and manipulate has exploded. This paper describes several automation techniques for implementing HTS. How to manage large volumes of data will be discussed. Optimizing a system for reliability will be highlighted. Other key issues including timing and synchronization, error checking and system diagnostics, and redundancy of systems will be discussed.

*Keywords:* analysis, instrumentation, sample preparation
*Application code:* high-throughput chemical analysis
*Methodology code:* sampling and sample preparation

### NEW CHROMATOGRAPHIC VALVES FOR PROCESS AND LABORATORY CHROMATOGRAPHS

***Yves Gamache***

*Contrôle Analytique Inc, 1076 Johnson Street, Thetford Mines, QC, Canada G6G 5W6*

Two new methods of manufacturing chromatographic valves will be demonstrated. One is for manufacturing rotary chromatographic valves and the other one is for manufacturing diaphragm type G.C. valves. With these new methods, the G.C. valves are no longer only a simple mechanical part used for sample injection and other sample fluid flow path switching schemes needed by G.C. users but they become an intelligent part of the G.C. system. The performance of existing systems in the industry will be compared with the new method. The benefits of these new valves will be demonstrated. Here are some of them.

(i) Lifetime > 3 years (continuous use).
(ii) Valve could be diagnostic online so use could be warned before loosing system performance (self diagnostic).
(iii) No cross port leak.
(iv) No inboard or outboard leaks.
(v) Vacuum operable to few thousand psi.

The new rotary valves could be used with G.C. or L.C. and the diaphragm valve is G.C. oriented. Furthermore the method could be used to rebuild or retrofit standard G.C. rotary valves extending by 3 times their lifetime expectancy.

*Keywords:* chromatography, sample introduction, specialty gas analysis
*Application code:* others
*Methodology code:* sampling and sample preparation

### MONITORING FERMENTER EXHAUST GAS WITH MINIATURE MODULAR SYSTEMS—ENABLING PAT

***David M. Simko***

*Swagelok Company, 31500 Aurora Road, Solon, OH 44139, USA*

Measurement of pH, ORP, dissolved oxygen, and conductivity is critical to an effective fermentation process. Usually these measurements are made with steam-sterilizable probes installed in the fermenter sidewalls or ports in the head. Analysis of fermenter exhaust gas is also critical. Spectrometers and gas chromatographs may be used to determine the components of the exhaust gas stream and concentrations. These techniques and methods have been employed for many years. Today researchers are working on improvements in analytical instruments, including miniaturization into microanalytical and eventually nanoanalytical devices. The FDA Process Analytical Technologies (PAT) Initiative is intended to promote the use of newer technologies for monitoring and controlling pharmaceutical product manufacturing processes for improved understanding and optimization. Some of these technologies require continuous interface with the process fluid and others extraction of a reliable and repeatable process fluid sample.

Sampling systems to extract and condition the sample prior to introduction into an analytical instrument will be utilized. Space on the fermenter head is at a premium and the number of access ports may be limited. Miniature modular technology has been developed; resulting in sampling systems that are more compact than the complex panels traditionally used in process analyzer systems. This paper explains the miniature modular system technology and discusses its application to the exhaust gas analysis system of a cell bioreactor as an example of how PAT may be employed to help monitor and control a bioprocess.

*Keywords:* biopharmaceutical, instrumentation, process analytical chemistry, sample handling/automation
*Application code:* pharmaceutical
*Methodology code:* sampling and sample preparation

### NEW SAMPLE STREAM SYSTEM AND SELECTION METHOD

***Yves Gamache***

*Contrôle Analytique Inc, 1076 Johnson Street, Thetford Mines, QC, Canada G6G 5W6*

As it is well known, the accuracy and reliability of process analyzers are directly linked to the quality of the valves, manifolds, fittings, and various hardware used in the sampling system. The best trace gas analyzer cannot have better performance than the sampling system it is connected to. In short, an analyzer is only as good as its sample system. By many orders of magnitude, today's trace gas analyzers outperform the analyzers designed 20 or 30 years ago and yet, it is not uncommon to find the same sampling system design philosophy found 30 years ago. For example, the quick connectors are still in use in many Air Separation plants even with gas analyzers having ppb sensitivity.

Our experience has demonstrated that in the process gas industries, or in the semiconductor ones, the cause of analytical system malfunctioning was traced to the sample stream selection system and this for more than 90% of the cases. In the presentation, two new methods of selecting sample will be demonstrated and compared with existing systems and methods in the field. One method is oriented for bulk and industrial gas industries, the other one is oriented for very-high-purity measurement equipment like APIMS.

*Keywords:* sample introduction, semiconductor, specialty gas analysis
*Application code:* others
*Methodology code:* sampling and sample preparation

### COMPARISON OF TWO DIFFERENT TECHNIQUES IN THE EVALUATION OF MERCURY EMISSIONS FROM A MUNICIPAL SLUDGE INCINERATOR

***Meyer Lallouz***

*City of Montreal, Environmental Service, 827 Boulevard Crémazie Est, Montreal, QC, Canada H2M 2T8*

In the ongoing efforts to monitor mercury emissions from the local municipal wastewater sludge incinerator, a gradual and continuous decline in Hg concentrations has been observed over several years. The decline coincided with the promulgation of USEPA method 29 as a sampling and analysis technique for mercury in stationary sources. It became apparent that validation of results obtained for the years prior to the application of method 29 was necessary and urgent in order to confirm the success of the reduction program at the source. Mercury concentrations were measured simultaneously by two methods; the previously applied technique, namely that described in The National Testing Program (NITEP) of the Canadian Environmental Technology Center and method 29 of the USEPA.

Isokinetic sampling was conducted throughout the testing program and analysis was carried out by cold vapor AAS following recovery and sample extraction. Generally, results show good comparison between the two methods. Corrected on the basis of 11%, $O_2$ results varied from 55 $\mu g/m^3$ to 91 $\mu g/m^3$ with overall average concentration of 79 $\mu g/m^3$. Differences varied from −1.4% to +22.7% when comparing method 29 to the NITEP method. This paper describes the main differences between these two techniques and discusses the results obtained and the statistical data generated. A brief review of historical accomplishments in the reduction of mercury emissions at this facility will be presented as well.

*Keywords:* air, atomic spectroscopy, environmental, validation
*Application code:* environmental
*Methodology code:* sampling and sample preparation

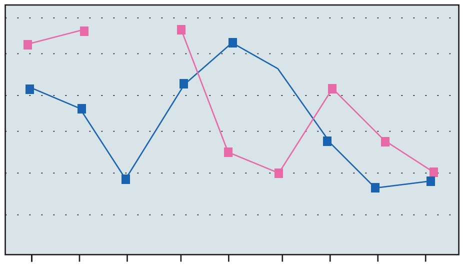

Figure 8

### OPTIMIZATION OF SPECTRAL IDENTIFICATION OF AGENT-RELATED CHEMICALS USING STATISTICAL FACTORS

***Jack C. Demirgian* and Francis D'Amico***

**Argonne National Laboratory, 9700 S. Cass Avenue, Bldg 203/E105, Argonne, IL 60439, USA*

Fourier transform infrared (FTIR) spectroscopy is used by the military for the detection and positive identification of chemical agents in the field. The consequences of false positive or false negative results are severe. Field personnel are not trained spectroscopists who can correctly identify spectra compromised by changing backgrounds and matrices, as well as spectral interference. Hence, automated detection and identification are essential to minimize errors. The objective of this study was to evaluate whether the statistical factors of Mahalanobis distance, F ratio, F distance, and spectral residuals can assist in correct identification of agent simulants. Quantitative releases of the agent simulant dimethylmethyl phosphonate (DMMP) were made in the laboratory by using a specially designed vaporizer. One hundred interferograms were collected during each release. Releases were performed at background temperatures of 25, 30, 35, and 40 °C. All releases were replicated each day and then duplicated on subsequent days. Additional quantitative DMMP releases were performed in the field against a background of grass and blue sky. Data were analyzed by coadding interferograms and Fourier processing to the spectral domain and also by Fourier processing each interferogram and then coadding each spectrum. Some resulting spectra were entered into a partial-least-square algorithm using PLSplus/IQ (Thermo Galactic, Salem, NH). The remaining spectra were analyzed to determine the effectiveness of the statistical factors in correctly identifying the analytes. Additional samples, including some containing interfering chemicals such as ozone, diisopropyl methane phosphonate (DIMP), and methanol, were also analyzed. The ability of the statistical factors to facilitate correct identification will be discussed.

*Keywords:* data analysis, FTIR, quantitative, statistical data analysis
*Application code:* homeland security/forensics
*Methodology code:* data analysis and manipulation

### USE OF NEURAL NETWORKS AND SPECTROSCOPIC METHODS IN FORENSIC ANALYSES

***John N. Driscoll,* Pat Hogan, Raksmey Im, Walter Johnson, Pol Perov, and Rebecca Romasco***

**PId Analyzers, LLC, 25 Walpole Park S. Drive, Walpole, MA 02081, USA*

We have shown previously (Pittcon Paper 11600-200, March 2004) that neural networks can be used in combination with XRF to provide identification of automobile paint samples. An XRF database of Ford, GM, and Chrysler Auto Paints was developed for this purpose. The neural network used was relatively simple with 8 input neurons, 4 hidden layer neurons, and 6 output neurons. In all cases the net was able to correctly identify the unknown sample used in a repeat measurement. The most common forensic methods for identification of organics in automotive paint are infrared and GC (Pyrolysis-MS) analysis. The XRF method does have some limitations as found previously (PC 11600-200). If this method is supplemented with additional spectroscopic methods such as reflectance (diode array spectrometer) and ellipsometry (ellipsometer operates over the wavelength range of 350–850 nm), the technique becomes much more powerful. The latter method is particularly useful for the detection and identification of thin films. A combination of neural networks with spectroscopic methods such as XRF, reflectance, and ellipsometry will be very useful for identification of the year and make of automobiles once the neural net is trained and a database is established. The identification of any vehicle should become a rapid process that can be run without the need for a highly trained specialist.

*Keywords:* array detectors, identification, spectroscopy, X-ray fluorescence
*Application code:* homeland security/forensics
*Methodology code:* UV/VIS

### REMOTE ANALYSIS OF CHROMIUM(VI) USING THE AQUATIC REMOTE MONITORING SYSTEM

***Stuart J. Chalk***

*Department of Chemistry and Physics, University of North Florida, 4567 St Johns Bluff Road S., Jacksonville, FL 32224, USA*

The Aquatic Remote Monitoring System (ARMS) is a remote analytical laboratory designed to test for evidence of bioterrorism or chemical warfare on a city water supply. The instrument uses a central sample intake system for collecting samples every hour, a sample processing system to precondition the sample for a variety of detectors, and detectors subsystems for actual analysis. The device is fully automated and can operate in a remote location for 1 month continuously. The capabilities of the system will be discussed highlighting the analysis of chromium(VI) using a modified diphenylcarbazide reagent.

*Keywords:* flow injection analysis, scientific data management, UV-VIS absorbance/luminescence
*Application code:* homeland security/forensics
*Methodology code:* others

### SYNTHESIS, PATTERNING, AND APPLICATIONS OF HIGH ASPECT RATIO GOLD NANORODS GROWN DIRECTLY ON SURFACES

***Francis P. Zamborini,* Aneta J. Mieszawska, and Zhongqing Wei***

**Department of Chemistry, University of Louisville, Louisville, KY 40292, USA*

This presentation describes the synthesis of gold nanorods directly on surfaces by reducing HAuCl4 onto surface-attached 3–5 nm diameter gold nanoparticle "seeds" with ascorbic acid in the presence of cetyltrimethylammonium bromide. Nanorod length (L) can be controlled between 200 nm and 1200 nm and aspect ratio (AR) between 6 and 22 by varying HAuCl4 concentration (see figure) and time in "growth solution." The yield is low (10%) and dispersity is high (25–35%), however. We describe atomic force microscopy (AFM) experiments closely examining the growth process and the role of the seed and also discuss methods for improving yields and reducing size dispersity. Nanorods were patterned by patterning gold nanoparticle "seeds" on surfaces prior to placement in growth solution. Gold nanorods were mechanically manipulated between electrode gaps and connected electrochemically. This work is important because improved methods for controlling the synthesis and assembly of 1D nanomaterials are needed in order to better study the relationship between structure and function (catalytic, fluorescence enhancement, Raman scattering, etc.) and compare experimental results with theory. Assembly, patterning, and alignment of gold nanorods on surfaces are also important for their use as surface plasmon waveguides and electronic-based sensors.

*Application code:* nanotechnology
*Methodology code:* microscopy

### SIMULTANEOUS SINGLE-MOLECULE OPTICAL AND ELECTRICAL RECORDING OF DNA/PROTEIN ANALYTES INTERACTING WITH NANOPORES IN MEMBRANE BILAYERS

***Daniel L. Burden,* Lisa M. Burden, Emily L. Chandler, John J. Kasianowicz, and Alyssa L. Smith***

**Department of BiNano Chemistry, Wheaton College, Wheaton, IL 60187, USA*

In the last decade, nanoscale pores of both biological and anthropogenic origin have been used for a variety of sensing applications. The alpha hemolysin ion channel (aHL),

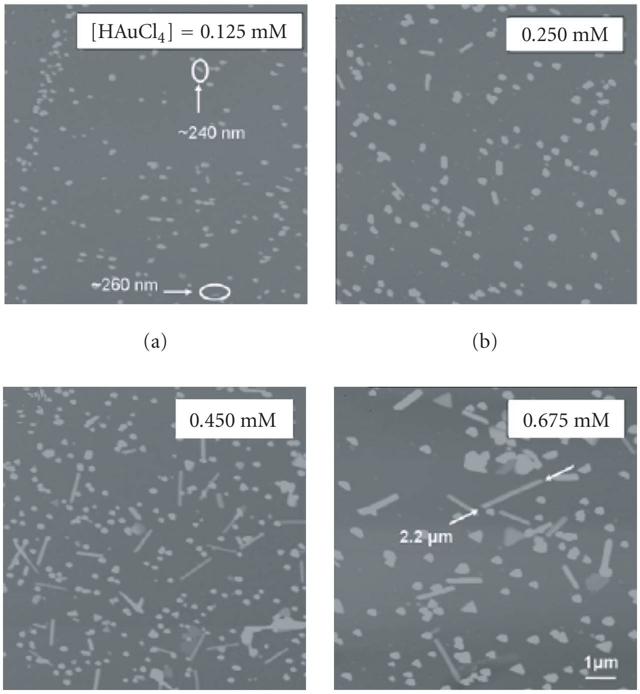

(c) (d)

Figure 9

produced by *Staphylococcus aureus,* is of optimal size and stability for detecting compounds at the single-molecule or single-ion level. Analytical applications based on this small nanopore (~ 10 nm long, ~ 1.5 nm id) include H+/D+ discrimination, heavy metal cation sensing, proteins, small molecules, and polynucleotides. In this presentation, we describe single-molecule fluorescence measurements performed on membrane-bilayer-incorporated aHL as it interacts with protein-capped polynucleotides ranging from 50–100 nucleotides in length. We utilize confocal fluorescence microscopy coupled with ion-channel electrical recording to simultaneously capture single-molecule dynamics from the membrane interface. Fluorescence correlation analysis is used to determine the surface number density and diffusion constants of membrane-incorporated complexes. The simultaneous recording technique enables instantaneous changes in transmembrane current to be correlated with changes in fluorescence dynamics occurring at the lipid membrane interface. Our measurements reveal that significant levels of analyte can remain adsorbed to the surface after a potential reversal is executed to eject material from the pore lumen. The findings impact the design of future nanopore-based sensors. In addition, the signal-to-noise ratio of our optical measurements indicates that the microscope can readily detect membrane-associated species below 10–6 monolayers, a sensitivity that surpasses most other in vitro surface analysis techniques such as surface plasmon resonance and ellipsometry. Funding for this work was provided by the Camille and Henry Dreyfus Foundation, ACS-PRF, Research Corporation, and NIST.

*Keywords:* biosensors, membrane, microscopy, nanotechnology
*Application code:* nanotechnology
*Methodology code:* microscopy

### HIGH-RESOLUTION MAPPING OF COMPOSITIONAL DIFFERENCES AT ELECTRODE INTERFACES BY ELECTRIC FORCE MICROSCOPY

***Grant A. Edwards,[*] Adam J. Bergren, Jeremy D. Driskell, Robert J. Lipert, and Marc D. Porter***

*[*]Department of Chemistry, Institute for Combinatorial Discovery, Iowa State University, Ames, IA 50011-3020, USA*

The characterization of organic thin films is of vast importance to many areas in analytical chemistry and surface science (eg, electrocatalysis, corrosion inhibition, organic electronic devices, and biocompatibility). This presentation describes results that demonstrate the ability of electric force microscopy (EFM) to map terminal group differences of spatially patterned organic monolayers with high resolution. Benzyl mercaptan-derived monolayers (X-$C_6H_4$-$CH_2$-SH) with different substituents (X = H-, Cl-, Br-, F-, $(CH_3)$ 3C-, $CH_3O$-) are interrogated on a smooth gold substrate by EFM. Plots of phase shift (D[phi]) versus dc bias voltage (DE) are presented. The basis for the expected two-dimensional phase-based image contrast is derived from plots of D[phi] versus DE. The images show the EFM has sufficient contrast to function as a compositional mapping methodology for patterns of these monomolecular films. Approaches are also described to estimate the dipole and capacitance of these coatings, along with parallel plate capacitor models to unravel the basis of the contrast mechanism. Support is acknowledged from Phillips Petroleum Corporation graduate research fellowship and the United States Department of Energy under contract W-7405-ENG-82.

*Keywords:* electrodes, microscopy, nanotechnology, surface analysis
*Application code:* nanotechnology
*Methodology code:* surface analysis/imaging

### DETERMINATION OF LEAD(II) IN WATER BY COLORIMETRIC SOLID-PHASE EXTRACTION

***Neil C. Dias,[*] James S. Fritz, and Marc D. Porter***

*[*]Department of Chemistry, Institute for Combinatorial Discovery, Iowa State University, Ames, IA 50011-3020, USA*

A quick, simple analytical method for the low level of lead(II) in water samples is described. The method concentrates lead(II) on a small solid-phase extraction disk, which is then quantified directly on the disk by diffuse reflectance spectroscopy (DRS). This method, termed colorimetric solid-phase extraction (C-SPE), requires only 1–2 minutes for complete workup and is suitable for operation in a wide range of environments. The procedure first adds an excess of potassium iodide to a 10.0 mL sample to produce the anionic

PbI4 2-colored complex at a pH of 3.1 ± 0.2. This complex is then exhaustively extracted onto the disk which was previously impregnated with cetylpyridinium chloride. The amount of complex extracted is then determined at 420 nm by a hand-held DRS instrument. A linear calibration plot was obtained for lead(II) concentrations from 0.02–2.50 ppm, with a detection limit 0.008 ppm. Results from interference tests using Ni(II), Cd(II), Zn(II), Cu(II), and Hg(II) yielded deviations of ± 10%. Potential applications will also be discussed.

*Keywords:* environmental/water, solid phase extraction, spectroscopy, trace analysis
*Application code:* environmental
*Methodology code:* others

### HOLLOW CATHODE PARTICLE BEAM-GLOW DISCHARGE MASS SPECTROMETRY FOR COMPREHENSIVE SPECIATION ANALYSIS

***Jacob L. Venzie*[*] *and R. K. Marcus***

[*]*Department of Chemistry, Biosystems Research Complex, Clemson University, Clemson, SC 29634, USA*

The particle beam-glow discharge mass spectrometer (PB-GDMS) developed in our laboratory, based on a commercial Extrel Benchmark instrument, has demonstrated its value as a liquid chromatography mass spectrometry (LC/MS) detector. The glow discharge source provides mass spectra that contain both elemental and molecular information. This makes the PB-GDMS particularly well suited to applications such as metal speciation where the sensitivity of metal ion detection can be augmented with molecular information to confirm the chemical species. The initial pin-type electrode design of the glow discharge source has drawbacks, moderate sensitivity and pin position effects. In order to improve the sensitivity and reproducibility, a hollow cathode (HC) glow discharge ionization source has been developed. The figure below shows a schematic diagram of the PB-HC coupling. Dry analyte particles enter from the orthogonal to the cathode axis while the discharge gas (Ar or He) is supplied near the anode. A tungsten anode is connected to a positive high-voltage power supply while the rest of the source is held at ground potential. An extraction lens is used to help pull ions from the discharge, with the gas flow through the HC set by choice in orifice diameter. Hollow cathode glow discharges exhibit much higher electron densities which greatly improve the ionization efficiency and hence the overall sensitivity of the instrument (P. J. Slevin and W. W. Harrison, "The hollow cathode discharge as a spectrochemical emission source," *Appl. Spectrosc. Rev.*, vol. 10, pp. 201–255, 1975). The hollow cathode source produces higher ion beam currents than the original pin cathode discharge geometry. The extraction lens (not present in the earlier design) improves the ion sampling efficiency. The hollow cathode has demonstrated its benefits in terms of higher signal intensities and higher excitation energies by producing spectra with much better signal to background ratios.

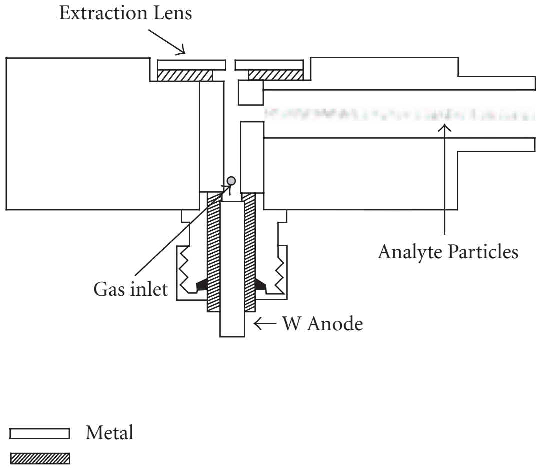

Figure 10

*Keywords:* environmental/biological samples, instrumentation, mass spectrometry, particle beam
*Application code:* environmental
*Methodology code:* mass spectrometry

### IN-SITU FIBER OPTIC RAMAN SENSOR FOR ENVIRONMENTAL ANALYSIS

***Tina M. Battaglia*[*] *and Karl S. Booksh***

[*]*Department of Chemistry and Biochemistry, Arizona State University, Mail Stop 1604, Tempe, AZ 85287, USA*

A field portable fiber optic Raman able to take measurements in terrestrial and natural waters and robust enough to withstand the corrosive environment of the hydrothermal vents is discussed. Presented here are results from employing the system to measure sulfate and various other salts in as hot springs and salinity in and around deep-sea hydrothermal vents. A 785 nm diode laser was used in conjunction with a fiber optic Raman probe, single-board computer or laptop, and a CCD detector. Using the fiber optic probe with a sapphire ball lens at ambient conditions, the detection limits of $SO_4^{-2}$, $CO_3^{-2}$, and $NO_3^-$ were determined to be approximately 0.11, 0.36, and 0.12 g/L, respectively. A novel flow cell using Teflon AF as a waveguide and a sapphire ball lens was designed for enhancement of signal. Using the flow cell the concentration of sulfate in 4 Arizona hot springs were determined. Effects from the cold temperatures of seawater on equipment, addition of minerals commonly found in vent fluid plumes, pressures up to 3600 psi, and temperature of the hydrothermal fluid were also investigated. Results from a dive to the sea floor in November 2004 will also be discussed. Funding was provided by the National Science Foundation.

*Keywords:* environmental/water, raman
*Application code:* environmental
*Methodology code:* vibrational spectroscopy

### AUTOMATED SPECTROMETRIC PHOSPHATE MEASUREMENT

***Aaron S. Beatty,* Robert V. Menegotto, and Tiffany Reid***

**Man-Tech Associates Inc, 2 Admiral Place, Guelph, ON, Canada N1G 4N4*

The measurement of phosphate in water samples is vital for the monitoring of the safety of drinking water and the environmental impact of effluent water. High levels of phosphate in rivers and lakes cause a cycle of runaway growth which ultimately leads to dead water systems.

Due to budget constraints many small laboratories currently test for phosphate using a manual method that is both time and man-power intensive. The automated phosphate method was developed to provide a time- and cost-saving alternative using a fully automated spectrometer. The method has the added benefits of being quick, easy to use, and providing a secure database for defensible and traceable data. The method to be discussed measures free phosphate through the spectrometric EPA method 365.2 as part of an automated pH, alkalinity, color, turbidity, and phosphate system. The advantages of the automated system, details of the method, and statistical results for phosphate over a large concentration range will be presented.

*Keywords:* automation, environmental analysis, environmental/water, spectrometer
*Application code:* environmental
*Methodology code:* chemical methods

### A LOW-COST OPTICAL SENSING PLATFORM FOR BUILDING WIRELESS (CHEMICAL) SENSOR NETWORKS

***Dermot Diamond***

*Aic Adaptive Sensors Group, National Centre for Sensor Research, Dublin City University, Dublin 9, Ireland*

A low-power, high-sensitivity, very-low-cost light emitting diode (LED)-based instrument for intensity-based light measurements is described. In this approach, a reverse-biased LED functioning as a photodiode is coupled with a second LED configured in conventional emission mode. A simple timer circuit measures how long (in milliseconds) it takes for the photocurrent from the detector LED to discharge a capacitor from logic 1 (+5 V) to logic 0 (+1.7 V). The entire instrument provides an inherently digital output of light intensity measurements for a few cents. The light intensity-dependent discharge process has been applied to measuring concentrations of colored solutions and a mathematical model developed based on the Beer-Lambert law. The analytical performance of the device was investigated using bromocresol green before being employed for the analysis of environmentally important species (eg, Cadmium II and Lead II) in water. Results show that the performance of this LED-based device is surprisingly good, in terms of sensitivity and LOD, and is comparable to conventional bench top uv-vis instrument but with the advantages of being ultra low cost, low power, and simple to operate.

This device could therefore function as a fundamental building block of wireless (chemical) sensor networks capable of monitoring many analytes on the basis of colorimetric assays in liquid form or as a chromoreactive film deposited on the LED sensor. Strategies for the introduction of low-cost, low-power, low-bandwidth wireless communication with this platform will be discussed.

*Keywords:* sensors
*Application code:* environmental
*Methodology code:* sensors

### A BACILLUS SPHAERICUS-BASED BIOSENSOR FOR MONITORING NICKEL IONS IN INDUSTRIAL EFFLUENTS AND FOODS

***Neelam Verma* and Minni Singh***

**Department of Biotechnology, Punjabi University, Patiala, Punjab 147002, India*

People have always been exposed to heavy metals in the environment. Monitoring the environment for the presence of such compounds which may adversely affect human health and local ecosystems is a fundamental part of the regulation. In many monitoring situations, biosensors can be expected to be the best available technology. This work aims at the development of a microbial system based on inhibition phenomenon to monitor nickel toxicity. Nickel is associated with causing adverse health effects such as dermatitis and vertigo in humans. It finds applications in electroplating, coinage, electrodes, jewellery, and alloys. The foods rich in Ni(II) are nuts, beans, oats, and wheat. The biocomponent of the biosensor was microbial-based. A urease-yielding microbe was isolated from soil using microbiological techniques and was identified by MTCC, Institute of Microbial Technology (IMTECH), Chandigarh, India, as gram +ve *Bacillus sphaericus* MTCC 5100. Immobilization of the microbe was done by physical adsorption onto Whatman no. 1 filter paper; the paper was dried and coupled to the body of the ion-selective electrode. Under similar conditions the biocomponents showed consistent activities. The transducer was a benchtop potentiometer (Cyberscan 2500) in conjunction with an $NH_4^+$ ion-selective electrode (ISE Code no. EC-NH4-03) that detects the electrode potential developed across the membrane of the electrode when it comes in contact with $NH_4^+$ ions released as a result of urea hydrolysis. This was followed by the calibration of ISE. The detection of Ni(II) was based on inhibition of urease activity. There is increased inhibition with increasing concentrations of Ni(II) ions having a linearity between the concentration of metal ion and the % inhibition caused by it. The range of Ni(II) detection by the developed biosensor is 0.002–0.04 ppb with a response time of 1.5 minutes. For application, the Ni(II) laden effluent was procured from an electroplating industrial unit and was found to have a concentration of 100.0 ppm

Ni(II). In foods, wheat flour sample was acid-digested and Ni(II) was specifically complexed and extracted in the presence of other cations, and had an Ni(II) concentration of 0.044 ppm as determined by the developed biosensor. The operational stability of the biocomponents extended over 8 weeks. The developed system has a reliability of 91.5% and 90.6%, respectively for the samples and could possibly replace the existing conventional techniques of analysis.

*Keywords:* bioanalytical, biosensors, environmental/water, food science
*Application code:* environmental
*Methodology code:* sensors

### NEXXIS ELN: AUTOMATE MANAGEMENT OF SOP FORMS

***Steve Bolton***

*Labtronics Inc, 546 Governors Road, Guelph, ON, Canada N1K 1E3*

Paper-based SOP forms are used extensively for recording laboratory data. These Word or PDF documents are printed out, numbered, and then carried around by technicians who collect data while performing QC analysis. As various sections are completed and approvals are required, the documents move from desk to desk, creating an organizational nightmare for lab managers and supervisors. NEXXIS ELN is a specialized electronic laboratory notebook for the QC lab that eliminates that organizational nightmare by converting SOP documents into an electronic format and then providing full control over their completion and management. Instead of having to track down paper copies of these documents, NEXXIS ELN automatically routes the electronic documents from user to user while management monitors the documents' progress directly from their desktops.

Data can be manually entered or NEXXIS ELN can collect sample data directly from the instruments and transparently add it directly into the document. Data such as sample information or reagent expiry dates can be automatically extracted from other databases and used to update the electronic document.

Entries can be validated, limit checks can be applied, and checks can be made for missing or incorrect data. NEXXIS ELN can automatically enforce multiple layers of review in the sign-off process for each document, ensuring compliance with the SOP. NEXXIS ELN stores electronic SOP documents both as an image and as database entries. This provides the best of both worlds with an ease to review document image as well as the ability to perform complex database queries, including reporting of historical trends and reporting to LIMS or other enterprise systems.

*Keywords:* lab management, laboratory informatics, sample and data management, scientific data management
*Application code:* laboratory management
*Methodology code:* laboratory informatics

### HORIZON WEB PORTAL: SECURE, WEB-BASED PROJECT SETUP, DATA INQUIRY, AND REPORT GENERATION

***J. J. Tomlinson,[*] W. Elliott-Smith, M. Metivier, and T. Radosta***

*[*]ChemWare, Inc, 900 Ridgefield Drive Suite 150, Raleigh, NC 27609, USA*

The HORIZON Web Portal connects laboratories and their clients through secured access to order entry, status on samples, and final analytical results. Eliminating the traditional LIMS bottleneck of waiting for IT staff to write a report, the Web Portal provides extensive self-service business intelligence tools for internal and external users to generate knowledge and insight via rich-content ad-hoc reporting. Web Portal users control extracted information by filtering, sorting, and grouping, and can download extracted information directly to the browser, MS Excel, text, and PDF.

HORIZON Web Portal is a highly scalable, server-based reporting platform that enables reporting and data mining from ChemWare's HORIZON LIMS, Waters NuGenesis SDMS, and other business systems and data repositories.

Web Portal empowers laboratory clients to perform common administrative functions such as requesting quotes, viewing analysis catalogs, ordering shipments, and prelogging samples. It allows clients to bypass typical bottlenecks in communication and workflow, and enables efficient processing of client-specific contracts, samples, data, and reports.

Web Portal provides tools for focused, two-way electronic communication between the laboratory and the client. This reduces the workload on laboratory client services personnel and bypasses the overburdened e-mail and voice-mail channels.

There are two basic types of Web Portal data consumers: LIMS users (lab staff and management) and external users (lab clients). HORIZON maintains a continuous transaction log of users' portal activities, and provides a single sign-on and centralized point of control for user administration. Within Web Portal is a role-based security console that controls which reports a user can view and generate.

*Keywords:* laboratory automation, LIMS, scientific data management, software
*Application code:* laboratory management
*Methodology code:* laboratory informatics

### MULTIVARIATE STATISTICAL PROCESS CONTROL (MSPC) USING STATISTICA

***Robert Eames***

*StatSoft, Inc, 2300 E. 14th Street, Tulsa, OK 74104, USA*

STATISTICA multivariate statistical process control (MSPC) is a software module for applying multivariate data analysis techniques to identify root causes of quality problems and to monitor the most important factors driving product quality. MSPC augments traditional univariate methods for quality control, that are limited by their ignoring the inherent correlations and interactions between process variables.

MSPC works by capturing the underlying relationships between process variables and the signature of the process over time. MSPC is trained on good batches and then trained models are applied for process monitoring of new batches as they mature. MSPC provides a wealth of graphical techniques and diagnostic plots for ongoing process monitoring, deployed within the multiuser, high-performance STATISTICA analytics platform (http://www.statsoft.com).

*Keywords:* data analysis, process monitoring, quality, quality control
*Application code:* quality
*Methodology code:* data analysis and manipulation

### STARLIMS SOLUTION FOR LAB CERTIFICATION/LICENSING

***Dianne C. Hollenbeck***

*StarLIMS, 273 Cherryview Drive, Portage, MI 49024-6892, USA*

This presentation will describe a solution for managing the certification/licensing of laboratories. The solution has been developed by StarLIMS as part of its product offering for Public Health Laboratories. In this presentation I will elaborate on the approach taken to develop the solution. Essentially, the project team followed an adaptation of the waterfall method. The requirements and general design were developed via group conference calls and several individual site calls. Participants included lab certification officers from PHLs in four different states whose areas of expertise spanned clinical, dairy, and environmental certification programs.

I will further detail the issues encountered in deriving the solution. The solution needed to provide enough structure to meet the specifications for lab certifications/licensing outlined in the Requirements for Public Health Laboratory Information Management Systems, and also needed to provide enough generality to support certification programs in multiple domains (eg, clinical, dairy, environmental). The terminology used in these different domains, as well as the varying requirements of the programs, raised several challenges that needed to be addressed in order to arrive at a common solution.

*Keywords:* lab management, LIMS
*Application code:* laboratory management
*Methodology code:* others

### OPTIMIZING PREPARATIVE SCALE PURIFICATION OF BIOSYNTHETIC HUMAN INSULIN BY HIGH PERFORMANCE REVERSED-PHASE LIQUID CHROMATOGRAPHY

***Derek W. Chan,* Grace Vydac,* and Reno T. Nguyen***

**17434 Mojave Street, Hesperia, CA 92345, USA*

Reversed-phase high-performance liquid chromatography is the method of choice to analyze and purify peptides and proteins in research and production. This study documented how pore size (100–120 Å) and particle size (10 $\mu$m) play an important role in preparative separations where purity and yield are critical. Two commercially available reversed-phase resins (VYDAC DENALI C18 reversed-phase column and another C18 vendor column) were evaluated on an analytical scale on the basis of resolution of impurities from the insulin main peak. When larger quantities of peptide and protein are to be purified, the MODcol SPRING column, a patented dynamic axial compression technology, is the preferred choice at the production scale. Hundred mg quantities of the insulin were loaded onto a 25 mm id $\times$ 144 mm preparative SPRING column, with separations yielding a high-purity product with ~ 80% recovery. An internal calibration was established to determine the linearity of the loading and to interpolate the mass recovery. Various fractions were collected from the preparative column for further characterization of the insulin main peak and its impurities utilizing the analytical column as well as a DENALI capillary LC-MS column. One pmol of insulin was loaded onto the 300 $\mu$m id$\times$150 mm C18 column, although upper fmol sensitivity is clearly possible. Excellent peak shape obtained with the DENALI resin ensured the highest sensitivity for the mass spectrometric detection of impurities including variants of insulin.

*Keywords:* HPLC, liquid chromatography, liquid chromatography/mass spectroscopy, peptides
*Application code:* bioanalytical
*Methodology code:* separation sciences

### IDENTIFICATION OF SUGARS BY LIQUID CHROMATOGRAPHY/MASS SPECTROMETRY IN SAMPLES OF FORENSIC INTEREST

***Jeffrey N. Leibowitz,* Jay A. Clark, David A. Henningsen, Marc LeBeau, and Lisa G. Schumacher***

**Federal Bureau of Investigation, 2501 Investigation Parkway Chemistry Unit, Room 4220, Quantico, VA 22135, USA*

Samples submitted for forensic analysis often contain sugars from common items such as food products and drugs. Identification of these sugars can provide very useful information in cases ranging from threatening letters and product tampering, to drug and bomb-making materials. Numerous chromatographic methods have been published on the analysis of sugars. However, few have focused on the analysis and positive identification of common sugars in forensic samples by liquid chromatography/mass spectrometry (LC/MS).

LC/MS identification required the modification of established chromatographic methods in order to obtain a system-suitable separation.

The data from different separation schemes, ionization techniques, and LC/MS/MS will be presented. In addition, examples of encountered matrixes and sample preparation techniques will be discussed.

*Keywords:* carbohydrates, forensic chemistry, liquid chromatography/mass spectroscopy
*Application code:* homeland security/forensics
*Methodology code:* liquid chromatography/mass spectrometry

### ON-LINE SAMPLE PRECONCENTRATION USING A MONOLITHIC COLUMN FOR MEASURING 16 PHTHALATE METABOLITES IN URINE

***Kayoko Kato,* Antonia M. Calafat, Larry L. Needham, and Manori J. Silva***

**Centers for Disease Control and Prevention, 4770 Buford Hwy, Atlanta, GA 30341, USA*

We developed a sensitive, high-throughput, on-line solid-phase extraction method, coupled with high-performance liquid chromatography-tandem mass spectrometry, to simultaneously quantify in human urine 16 phthalate metabolites (phthalic acid, monomethyl phthalate, monomethyl isophthalate, monoethyl phthalate, mono-(3-carboxypropyl) phthalate, mono-iso-butyl phthalate, mono-n-butyl phthalate, monocyclohexyl phthalate, monobenzyl phthalate, mono-(2-ethylhexyl) phthalate, monooctyl phthalate, mono-3-methyl-5-dimethylhexyl phthalate (mono-iso-nonyl phthalate), mono-3-methyl-7-methyloctyl phthalate (mono-iso-decyl phthalate), mono-(2-ethyl-5-oxohexyl) phthalate, mono-(2-ethyl-5-hydroxyhexyl) phthalate, and mono-(2-ethyl-5-carboxypentyl) phthalate). Pretreatment of the urine sample was performed automatically using a Surveyor Autosampler operated using the Xcalibur software. The analytes were extracted from urine and preconcentrated on a silica-based monolithic column and chromatographically separated on a Betasil Phenyl column using a nonlinear 0.1% acetic acid in water and acetonitrile solvent gradient. The total run time from sample injection to analysis was 27 minutes. The method is not labor intensive and involves minimal manual sample preparation. It uses small amounts of urine (100 $\mu$L), and has limits of detection generally between 0.1 and 0.2 ng per mL of urine. It is accurate and precise with inter- and intra-day coefficients of variation less than 10%.

This method combined with the automated sample preparation system provides fast generation of data and it is adequate for large epidemiological studies. The method was validated on spiked pooled urine samples and on urine samples from 43 adults with no known exposure to phthalates.

*Keywords:* environmental/biological samples, high-throughput chemical analysis, liquid chromatography/mass spectroscopy
*Application code:* bioanalytical
*Methodology code:* liquid chromatography/mass spectrometry

### FAST ON-LINE ELECTROCHEMICAL SYNTHESIS OF PHARMACEUTICAL DEGRADANTS AND METABOLITES FOR PROFILING, IDENTIFICATION, AND QUANTITATION

***Darwin J. Asa,* Ian N. Acworth, Rod Cole, Paul Gamache, Mike Granger, Ryan McCarthy, David Meyer, and Marjorie Soloman***

**ESA, Inc, 22 Alpha Road, Chelmsford, MA 01824, USA*

The synthesis of substances derived from investigational compounds is often a major bottleneck in small molecule drug discovery and development. These related substances include degradants and metabolites that are commonly a result of oxidative processes. Several studies utilizing electrochemical (EC) flow cells online with mass spectrometry (MS) have demonstrated that electrochemically derived products often correspond to biological metabolites and chemical degradants. The objective of these studies is to examine the use of controlled-potential electrolysis in flowing solution as a means of small-scale synthesis to facilitate pharmaceutical analyses in discovery and development. EC flow cells and large surface area working electrodes were used on-line with MS. Initial results using ng quantities of material have indicated selective and semiquantitative formation of relevant phase I metabolic products (eg, S and P oxidation, N-dealkylation, and dehydrogenation). Specific phase II metabolites can also be formed by addition of appropriate nucleophile within or downstream from the EC cell. Reaction cells placed before and/or after the HPLC column allow separation, concentration, and purification of reactants and products.

*Keywords:* detection, electrochemistry, mass spectrometry, pharmaceutical
*Application code:* pharmaceutical
*Methodology code:* liquid chromatography/mass spectrometry

### OPTIMIZING HIGH-THROUGHPUT APPLICATIONS USING 20 MM AND 30 MM LENGTH HPLC COLUMNS

***Kenneth Jones,* Robert T. Moody, and Carl L. Zimmerman***

**Advanced Chromatography Technologies, Aberdeen Science and Technology Park, Balgownie Technology Centre, Aberdeen AB22 8GW, UK*

This paper will illustrate a number of method development strategies to enable both the optimization of existing methods and also the development of new methods when using new 20 mm and 30 mm length columns—resulting in significantly reduced analysis times and increased productivity. Nowhere is the requirement for an ultra inert stationary phase more important than in high-throughput analysis or LC/MS. Even subtle differences in silanol activity between columns can markedly affect the chromatography in the very short, fast columns typically used. Additionally, peak tailing due to silanol activity has a profound effect on detection limits in high-sensitivity assays. The importance of stationary-phase inertness will be highlighted by a comparison of a number of commercially available LC/MS columns.

Similarly, the requirement for long lifetime columns is essential for high-throughput analyses, where mid-run column failure must be avoided. Column lifetime using typical high flow, fast gradient conditions will be investigated, and the importance of column lifetime resulting from both the mechanical stability of the silica and the column packing technology employed will be reviewed. This paper should prove attractive to analysts already using conventional 3 $\mu$m and 5 $\mu$m particles who do not wish to invest in the instrumentation required for the use of sub-two micron particles or ultra high-pressure applications.

*Keywords:* high-throughput chemical analysis, HPLC, HPLC columns, liquid chromatography/mass spectroscopy
*Application code:* high-throughput chemical analysis
*Methodology code:* liquid chromatography

### COMPUTER-AIDED DESIGN AND ANALYSIS OF EXPERIMENTS FOR ROBUSTNESS VALIDATION OF AN HPLC ASSAY METHOD FOR FENTANYL

***Jason Jennissen***

*Cima Labs, Inc, 7325 Aspen Lane, Brooklyn Park, MN 55428, USA*

The robustness of an HPLC assay method for fentanyl was evaluated using design of experiment (DOE) facilitated by statistical analysis software. This poster shows the powerful utility of currently available statistical software packages equipped with DOE, and the advantage of using DOE to perform robustness experiments over the traditional, one-parameter-at-a-time approach.

All essential method responses, including peak tailing, precision, and resolution, were shown to be robust to slight variations in method parameters. However, when two mobile phase parameters, buffer pH, and % acetonitrile were varied at the same time, the resolution between fentanyl and a known impurity was compromised. The discovery that two parameters varied simultaneously cause a critical resolution failure that is not apparent when the same parameters are varied independently was only possible through the use of DOE and would not have been discovered by varying one parameter at a time.

The study advanced in this poster describes the use of a resolution III, 2-level, fractional factorial (2III7-4) experimental design in which main effects are confounded with second-order and higher-interaction effects. The subsequent data analysis showed with 95% confidence that standard and sample precision was not influenced by small changes in any of the method parameters.

Percent acetonitrile and stationary-phase batch number directly influenced the retention time and symmetry of the fentanyl peak, respectively. In these cases the corresponding regression model coefficients were studied to determine the predicted response variations, which were found to be within acceptable ranges. The fentanyl assay method was deemed robust to method parameter changes within the ranges studied, except when % acetonitrile and buffer pH are varied simultaneously.

*Keywords:* HPLC, statistical data analysis, validation
*Application code:* validation
*Methodology code:* liquid chromatography

### GUIDELINES FOR ACHIEVING REPRODUCIBLE HIGH-PERFORMANCE PREPARATIVE HPLC SEPARATIONS

***Kenneth Jones,[*] Robert T. Moody, and Carl L. Zimmerman***

*[*]Advanced Chromatography Technologies, Aberdeen Science and Technology Park, Balgownie Technology Centre, Aberdeen AB22 8GW, UK*

Chromatographers with experience in preparative HPLC know that resolution and loadability are of utmost importance. The greater the resolution, the higher the sample load and the faster pure compound can be obtained. The ability to optimize resolution at the preparative scale means starting with high-performance separations at the analytical scale.

As part of a typical method development strategy, this paper will highlight the importance of reproducible scale up from analytical to preparative scale. As loadability is a function of resolution, the importance of choosing the correct bonded phase for optimum preparative resolution will be discussed, as will silica stability and proper packing techniques to assure a long column lifetime.

*Keywords:* chromatography, HPLC, HPLC columns, prep chromatography
*Application code:* others
*Methodology code:* liquid chromatography

### MUTUAL AUTOMATED PEAK MATCHING IN CHROMATOGRAPHIC METHOD DEVELOPMENT

***Michael McBrien,[*] Andrey Bogomolov, Eduard Kolovanov, and Vitaly Lashin***

*[*]Advanced Chemistry Development, Inc, 90 Adelaide Street West Suite 600, Toronto, ON, Canada M5H 3V9*

Computer-based optimization tools streamline the process of chromatographic method development considerably. Peak movement is modeled as a function of one or more variables, allowing for effective visualization and optimization of the experiment across all potential parameter values. The speed and ease of application of tools are limited, however, by the necessity of tracking the location of each analyte across each of the chromatographic runs.

This paper will describe new algorithms designed to take advantage of hyphenated techniques such as LC/UVVis and LC/MS in order to automate the chromatographic peak matching process. The principles of mutual automated peak matching (MAP) will be discussed as well as performance of the algorithm on real samples under conditions of varying signal/noise ratios and solvent conditions.

*Keywords:* array detectors, HPLC, liquid chromatography, method development
*Application code:* general interest
*Methodology code:* liquid chromatography

### HPLC SYSTEM CONTROL AND DATA ACQUISITION USING NET AND EMBEDDED WEB SERVICE TECHNOLOGY IN A LABORATORY NETWORK ENVIRONMENT

***Tsuyoshi Morikawa,* Shuzo Maruyama, Koji Okada, Naoki Osaka, and Teruhisa Ueda***

**Shimadzu Corporation, 1 Nishinokyo-Kuwabaracho Nakagyo-Ku, Kyoto 604-8511, Japan*

As computer networks are spreading into every laboratory, configuration of analytical instruments and data processing systems are changing to make the most of network environment.

Client-server architecture is one example of a productivity-enhancing feature widely used in today's software. However, a conventional client-server approach presumes a PC-based data acquisition server for every HPLC, increasing the complexity of a laboratory network. To make an HPLC network simple and smart, using the instrument embedded data acquisition server is ideal. Although such embedded server is limited in computing performance, it is sufficient for a web-based integrated system, where it serves only as an interface between the instrument and network-based elements. In such integrated analytical system, application software on a client PC easily communicates with analytical instruments and server PC's using web service. In order to demonstrate our new approach, we developed a web service capable HPLC controller which imports methods and sample schedules and exports data files. A .NET application utilizing the web service was also developed to demonstrate the performance of the system. The details of the system will be presented in the poster.

*Keywords:* analysis, computers, HPLC, lab management
*Application code:* laboratory management
*Methodology code:* liquid chromatography

### MODELING CHROMATOGRAPHIC DISPERSION: A COMPARISON OF POPULAR EQUATIONS

***Karyn M. Usher,* John G. Dorsey, and Carolyn R. Simmons***

*Department of Chemistry & Biochemistry, Florida State University, Dittmer Building, Tallahassee, FL 32306-4390, USA*

An analyte that is introduced onto a column as a finite band broadens as it moves along the column. This band broadening is generally attributed to three independent processes, including flow path inequalities, molecular diffusion, and resistance to mass transfer. Many equations have been derived in attempts to mathematically model the data. Some of the more popular of these include the equations of van Deemter, Giddings, Huber and Hulsman, Horvath and Lin, and Knox. Although the basis of each equation is theoretically different, the differences between them are minor, and most of the equations can be used to fit plate height data.

Chromatographers often collect efficiency data to monitor the performance of their columns and then use one of the above equations to fit the data. The choice of which equation to use can be daunting, since the theories are conflicting. Using our extensive collection of data, we have found the equation that offers the best fit. The study was performed using analytes covering a wide range of $k'$ values and mobile phases of different strengths. For this study, special precautions were taken to ensure that the observed broadening was due to only processes occurring in the column. The Foley-Dorsey equation was used to calculate the number of theoretical plates for the efficiency study. Also, the variance from extra-column sources was measured and subtracted from the system variance. From this study it was possible to choose the equation that gave the best fit to the obtained data.

*Keywords:* data analysis, HPLC, HPLC Columns, liquid chromatography
*Application code:* general interest
*Methodology code:* liquid chromatography

### PURIFYING VERY LARGE SAMPLES USING FLASH CHROMATOGRAPHY BY USING PEAK SHAVING AND PEAK RECYCLING

***Adam C. Wilkins***

*Gyan Srl, Unit 9, The Steadings Business Centre, Maisemore, Gloucester, Gloucestershire GL2 8EY, UK*

A flash chromatography system has been developed that enables large samples (20–250 g) to be separated into near pure fractions even if the sample does not achieve baseline separation.

The system works by shaving and recycling the peaks. This enables the high purity initial and final part of the merged peaks to be separated. The system contains a 140 mm ID cartridge packed with silica. The column is compressed both axially and radially to prevent swelling and the formation of channels which may reduce the performance of the cartridge.

Soluble (liquid) samples are loaded directly on to the top of the cartridge using a syringe and a three-way valve. They can also be loaded automatically using the pump. Solid samples and sparingly soluble samples are loaded using the solid sample introduction module (SSIM). The zero dead volume chamber can hold up to 500 g of silica and can also be used as a guard column to protect the main cartridge. Due to the high flow rates (up to 1500 mL/min) used in each separation, a special fraction collector is required for automated collection. The fractions are selected either by time or by using threshold activation whereby the signal from the detector is used as a trigger. The sizes of the collected fractions are unlimited.

*Keywords:* chromatography, combinatorial chemistry, liquid chromatography
*Application code:* others
*Methodology code:* separation sciences

### EXTRACTION OF MERCURY (II) BY SOL-GEL SORBENTS DOPED WITH SULFUR-CONTAINING EXTRACTANTS

***Flora E. Mercader,* Josefina de Gyves, and Eduardo Rodríguez de San Miguel***

**UNAM CENAM, Km 4.5 Carretera A Los Cués, Municipio El Marques, Querétaro 76241, Mexico*

Modern instrumentation for metal (Hg) determination in environmental samples has sufficient sensitivity to be used in routine work. However, preconcentration methods continue to be an interesting alternative for analysts that have limited instrumentation possibilities.

Novel sol-gel sorbents were synthesized and evaluated for the extraction and preconcentration of mercury (II) in digested sediment samples. Sorbents were prepared using the sol-gel technique with tetraethoxysilane (TEOS) as a silica precursor, and triisobutylphosphine sulfide (cyanex 471X) and bis (2,4,4-trimethylpentyl) dithiophosphinic acid (cyanex 301) as mercury extractants. The composition of the sol-gel sorbents was optimized with respect to the type of catalyzer and extractant concentration.

Mercury was extracted in a column packed with the sorbents under investigation and measured by cold vapor atomic absorption spectrometry. Extractant content was studied in the interval from 0.001 to 0.25 mmol of extractant. Reagents like $Na_2S_2O_3$, $NH_4Cl$, KI, and HCl at different concentrations were tested as desorption solutions. The overall extraction and backextraction process of mercury (II) was more efficient with Cyanex 301 and 6 $molL^{-1}$ as eluent.

The Cyanex 301 sorbent was applied for the preconcentration of mercury in a real sediment sample in order to measure its mercury content. The accuracy of the method was examined by the analysis of three different certified reference materials: SRM 2709 (1.40 $mgkg^{-1}$ of Hg), GSD-9 (0.083 $mgkg^{-1}$ of Hg), and GSD-11 (0.072 $mgkg^{-1}$ of Hg).

*Keywords:* atomic absorption, environmental, mercury, solid-phase extraction
*Application code:* environmental
*Methodology code:* separation sciences

### IMPROVING CHEMICAL ANALYSIS WITH ADVANCED ARRAY DETECTOR TECHNOLOGY

***M. B. Denton***

*Department of Chemistry, University of Arizona, PO Box 210041, Tucson, AZ 85721, USA*

Focal plane arrays have vastly increased sensitivity in many areas of low-light spectroscopy, including atomic emission, fluorescence, phosphorescence, and Raman. Recent advances in utilizing these detectors for spectroscopy and spectroscopic imaging will be presented.

New devices are continually being developed to provide even higher performance for critical applications. Several new detectors holding great promise for improving longer wavelength Raman spectroscopy are currently under development.

The impact of recent technological breakthroughs in array detectors, optical components and geometries of optical systems employing both dispersive and imaging optics will be contrasted with more conventional approaches. How these advances are propelling Raman into the mainstream of routine chemical analysis will be discussed.

New approaches for implementing advanced detectors for mass spectrometry and ion mobility spectrometry will be described. These detectors that have been implemented using technologies originally developed for advanced visible CCDs and infrared arrays hold great promise for vastly improving many areas of mass spectrometry. Current trends will be assessed and utilized to predict future application of improved focal plane arrays to modern chemical analysis.

*Keywords:* array detectors, mass spectrometry, multichannel spectrometry (CCD CID array), Raman
*Application code:* others
*Methodology code:* others

### LARGE-SCALE PROFILING OF BIOPOLYMERS BY CAPILLARY ARRAY ELECTROPHORESIS IN BIOINDUSTRIAL SETTINGS

***Greg Richardson* and Julia Khandurina***

**The Gel Company, Suite 240, 665 Third Street, San Francisco, CA 94107, USA*

Increasing needs for large-scale profiling of biopolymers in bioindustrial settings necessitated the development of automated and highly parallel capillary electrophoresis-based separation methods. In this work the adaptation of a 96-capillary array electrophoresis (CAE) system is introduced to implement high-throughput analysis of two major classes of biopolymers: nucleic acids and carbohydrates. This approach enabled rapid screening of double-stranded DNA fragments in a wide molecular weight range, as well as large-scale quality control assessment of single-stranded oligonucleotide probes, using non-covalent fluorophore labeling in both instances. Effect of several DNA staining dyes on separation efficiency has been evaluated. The applicability of internal fluorescent standards with different emission colors has been demonstrated with mathematical spectral overlap correction algorithms. High-performance profiling of monosaccharide and oligosaccharide mixtures by CAE has been also demonstrated and optimized to monitor reaction products from enzymatic polysaccharide digestion with numerous applications in agricultural, chemical, and food industries. Necessary operational protocol modifications, data normalization, processing and visualization tools have been developed, along with quantification approaches. Incorporation of internal fluorescent standards allowed for correction of accidental capillary-to-capillary and run-to-run variations in migration time and signal intensity. The developed methods require small sample amounts and offer quantification capabilities, enabling at the same time a high degree of automation for bioindustrial operation environments.

*Keywords:* bioanalytical, capillary electrophoresis
*Application code:* bioanalytical
*Methodology code:* capillary electrophoresis

### AUTOMATED LEARNING OF PROTEIN SUBCELLULAR LOCATIONS FROM FLUORESCENCE MICROSCOPE IMAGES

***Robert F. Murphy***

*Departments of Biology Science and Biomedial Engineering, Carnegie Mellon University, 4400 Fifth Avenue, Pittsburgh, PA 15213, USA*

Knowledge of the subcellular distribution of each protein is critical for an understanding of that protein's function. Limited information on protein location using controlled vocabularies can be obtained from protein databases, and predictions of low-resolution subcellular location can be made from protein sequence. Cell fractionation can be combined with traditional proteomics methods to determine subcellular location, also with low resolution. As an alternative, my group has developed automated approaches for determining protein location with high resolution for fluorescently tagged proteins. Using immunofluorescence images of ten different subcellular patterns in HeLa cells, we have demonstrated that our systems are able to not only recognize the patterns of all major organelles with nearly perfect accuracy, but also to discriminate between protein patterns that cannot be distinguished by visual inspection. The most critical components of these systems are numerical features that capture the essence of the location pattern without being overly sensitive to the position, rotation, or shape of a cell. These features can be used not only to recognize known patterns but also to measure the similarity between protein patterns with the goal of determining the number of patterns that can be statistically distinguished. We have coined the term location proteomics to refer to the application of this approach on a proteome-wide basis. This involves parallel tagging of many proteins, high-throughput, high-resolution microscopy, and application of unsupervised machine learning methods. Using this approach, we have created the first objective grouping of proteins by their location patterns. This was done using images of over 90 tagged proteins in mouse 3T3 cells collected via spinning disk confocal microscopy. This approach can also be used to detect subtle changes in protein patterns (such as those resulting from the presence of drugs or disease) and to provide critical information for systems-level modeling of protein behavior.

*Keywords:* computers, fluorescence, imaging, proteomics
*Application code:* proteomics and genomics
*Methodology code:* microscopy

### APPLICATION OF FAST SEQUENTIAL AA TO COMPLEX SAMPLE MATRICES

***Doug Shrader,* Christine M. Rivera, and Anna Tisinger***

**Varian, Inc, 201 Hansen Court, Suite 108, Wood Dale, IL 60191, USA*

For over 40 years, flame atomic absorption has provided analysts with a cost-effective method of determining metal concentrations in diverse sample matrices. The introduction of graphite furnace AA allowed low ppb levels to be measured accurately. Automation of both flame and furnace AA has eliminated many tedious steps, once part of solution preparation and analysis. AA is definitely a mature analytical technique. However, recent hardware and software design changes have led to improved productivity and performance in both flame and furnace AA. Fast sequential flame AA achieves the productivity and speed of sequential ICP by measuring all elements in each sample before moving on to the next one. The use of Internal Standards can improve long-term precision and accuracy of analysis. The SIPS Sample introduction pump system can eliminate most manual standard and sample dilutions while extending the analytical working range.

Over the last few months, various complex sample matrix sets have been analyzed by flame AA utilizing the enhanced capabilities described above. Among the sample sets analyzed for various metals were plating baths, petroleum, and agricultural samples. Results obtained will be discussed including analysis parameters and time, QC, precision, and accuracy.

*Keywords:* atomic absorption, instrumentation, metals, sample handling/automation
*Application code:* general interest
*Methodology code:* atomic spectroscopy/elemental analysis

### METHOD DEVELOPMENT STRATEGIES UTILIZING AXIALLY VIEWED ICP-OES WHEN SAMPLES CONSIST OF COMPLEX MATRICES

***Christine M. Rivera,* Michelle E. Cree, Doug Shrader, and Steve Wall***

**Varian, Inc, 201 Hansen Court, Suite 108, Wood Dale, IL 60191, USA*

The ICP-OES technique is appealing for the broad linear dynamic range, good detection limits, and multiple wavelength selection capabilities. However, there are some sample matrices that produce complicated structured backgrounds. These backgrounds can lead to erroneous results. This paper will review correction techniques when these structured matrices are present. Topics of discussion will include sample introduction setup, method parameter optimization, background correction, and standard additions. The matrices represented are potassium hydroxide, ethyl lactate, kerosene, high aluminum concentrations, high cerium concentrations, and high silicon concentrations.

*Keywords:* atomic emission spectroscopy, ICP, plasma emission (ICP/MIP/DCP/etc)
*Application code:* general interest
*Methodology code:* atomic spectroscopy/elemental analysis

### INTEGRATION OF GIANT MAGNETORESISTIVE SENSORS WITH MICROFLUIDIC SYSTEMS: DIRECT ON-CHIP SENSING OF MAGNETICALLY LABELED PATHOGENS

***Nikola Pekas,* Albrecht Jander, Anthony Popple, Marc D. Porter, and Mark Tondra***

**Department of Chemistry, Iowa State University, 40 Spedding Hall, Ames, IA 50011, USA*

Giant magnetoresistors (GMRs) are microfabricated structures that undergo a large change in electrical resistance as a function of magnetic field strength. This presentation shows that GMRs, commonly used as read-out devices in computer hard drives, may be integrated with microfluidic platforms for the purpose of on-chip detection and enumeration of magnetically labeled biological analytes. As a concept demonstration, we have designed and fabricated a microdevice that enabled (1) controlled formation of picoliter-sized droplets of a ferrofluid separated by nonmagnetic oil, and (2) continuous-flow sensing of these ferrofluid droplets. The flow velocity, droplet size, and droplet-formation frequency can readily be determined from the GMR response. These results were successfully validated by comparisons to fluorescence microscopy data. Several strategies to magnetically labeled bacterial and protozoan pathogens have also been explored in terms of surface-epitope density, antibody labeling chemistry, required magnetic properties, sensitivity, and selectivity. Details of these findings will be described.

*Keywords:* biological samples, biomedical, lab on a chip/microfluidics, sensors
*Application code:* bioanalytical
*Methodology code:* microfluidics/lab on a chip

### RAPID ANALYSIS OF BIOMOLECULAR INTERACTIONS USING CAPILLARY ELECTROPHORESIS WITH PHOTOLYTIC INJECTION

***Suminda Hapuarachchi* and Craig A. Aspinwall***

**Department of Chemistry, University of Arizona, 1306 E University Blvd, Tucson, AZ 85721, USA*

Real-time analysis of complex biological mixtures is of paramount importance in the biomedical community. Rapid separation of such mixtures offers a higher level of chemical information than traditional sensors, while maintaining sufficient temporal resolution to map the dynamics within the system. To date such separation has been realized predominantly with capillary electrophoresis (CE) utilizing a range of sample interface technologies such as optical gating and flow gating. Here, we present a novel interface based on photolysis of a fluorogenic label to introduce sub-nL samples that are subsequently analyzed with millisecond to second temporal resolution. This method has been used to analyze neurotransmitters and proteins with LODs below 5 nm with more than 1 000 000 plates/m achieved in less than 3 seconds temporal resolution. We are currently working on online immunoassays for real-time chemical monitoring of peptides such as insulin and glucagon in biological mixtures. Here, we present the design and application of this technology for high-speed, high-sensitivity separations of biologically relevant molecular systems.

*Keywords:* capillary electrophoresis
*Application code:* bioanalytical
*Methodology code:* capillary electrophoresis

### THE SIMULTANEOUS ANALYSIS OF FUEL OXYGENATES AND TRADITIONAL VOCS OF US EPA METHOD 8260 BY PURGE-AND-TRAP GC/MS

***Mark E. Krigbaum* and Tom Hartlein***

**Teledyne Tekmar, 4736 Socialville Foster Road, Mason, OH 45040, USA*

With the increased concern for ground water contamination of MTBE, a common fuel oxygenate additive, environmental laboratories are being asked to analyze water samples for these newer contaminants along with the usual list of volatile organic compounds. However, combining these lists of target compounds into one method brings new challenges. The polar oxygenates require changes in analytical parameters such as increased sample temperature to achieve the required detection levels. This paper will present optimized parameters for a new purge-and-trap GC/MS system as well as method performance data including calibration, method detection limits, precision, accuracy, and recommended hardware.

*Keywords:* environmental analysis, oxygenates, purge and trap, volatile organic compounds
*Application code:* environmental
*Methodology code:* gas chromatography/mass spectrometry

### THE ON-LINE SIMULATED DISTILLATION OF HYDROCARBON DISTILLATES

***Ulrich K. Gokeler***

*Siemens, 7101 Hollister Road, Houston, TX 77040, USA*

To ensure product quality and support automatic process control, simulated distillation is utilized to characterize the boiling point distribution of hydrocarbon mixtures such as naphtha, gasoline, kerosene, diesel, and lube oil. In order to compare results, the measurements are typically done in the laboratory in compliance with ASTM methods that provide calculations and relations between concentrations and boiling points. The simulated distillation measurement is typically done off line by extracting a sample and analyzing it in the plant laboratory. However, by providing on-line hydrocarbon boiling point characterization, automated and faster analysis can be achieved over the entire product boiling point range encountered in production plants.

Consequently a precise and repeatable measurement is ensured. On-line measurement provides significant time advantage for the characterization of hydrocarbon mixtures and therefore permits automatic process control and automatic product certification and reduces the laboratory analysis burden. By investigating sources of variations and discrimination, such as originating from sample injection, split discrimination, detection and integration procedures and consequently by optimizing analytical parameters, significant improvements of precision and repetition were achieved. The presentation discusses the systematic optimization of analytical parameters and presents data of on-line simulated distillation of various distillates compared to ASTM standards.

*Keywords:* gas chromatography, petroleum, process control
*Application code:* fuels, energy, and petrochemical
*Methodology code:* gas chromatography

### MULTIDIMENSIONAL HPLC DETERMINATION OF AROMATIC CONTENT AND MASS DISTRIBUTIONS IN ASPHALTS AND HEAVY DISTILLATES

***Ashraf Z. Khan***

*Intertek Caleb Brett, 327 Erickson Avenue, Essington, PA 19029, USA*

Quantitative measurements of hydrocarbon compositions in solid asphalt and liquid heavy distillate materials have been gaining importance recently in view of their wide applications. Heavy distillates are deep boiling fractions of crude oil and comprise of complex molecules having a wide range of initial boiling points (650 °F–1400 °F). The petroleum-based solid asphalts are much more complex and boil at much higher temperatures. Whereas asphalts are generally used as paving and roofing materials, the heavy distillates are widely used as feedstocks for refinery processes. Because of the importance of asphalts and the specific values of heavy distillates for upgrading the crude residue, improved characterization methods for compositional analyses are always demanded. In spite of the necessity of reliable and affordable techniques, there are only a handful of modern techniques, most notably a multidimensional HPLC, that can effectively examine these materials. In our laboratory we have demonstrated that a multidimensional HPLC, originally designed for heavy distillate analyses, can be effectively applied to asphalt materials. We show that the HPLC, comprising an evaporative light scattering and a photo diode array detectors with two normal-phase columns and operating under isocratic, gradient, and chromatofocussing modes, is an excellent tool for a molecular-level analysis of asphalt materials. Because of the higher boiling ranges, excellent mass balances are obtained for asphalts, unlike the heavy distillates. For the latter, perfect mass balances are not always achieved because of the presence of lighter fractions (< 650 °F). We also show that this technique is relatively free from noticeable matrix interferences and virtually eliminates the need for any tedious sample preparation procedure thereby making it a reliable and an affordable technique for the asphalt and refining industries.

*Keywords:* array detectors, HPLC, light scattering, petroleum
*Application code:* fuels, energy, and petrochemical
*Methodology code:* others

### IMPROVING RELIABILITY OF DETAILED HYDROCARBON ANALYSIS THROUGH AUTOMATED ALIGNMENT OF CHROMATOGRAMS

***Scott Ramos,* Don Crider, Elizabeth Harvey, and Brian Rohrback***

**Infometrix, Inc, PO Box 1528, Woodinville, WA 98072, USA*

In detailed hydrocarbon analysis (DHA), chromatographic methods are routinely used to determine the composition of refinery fractions. The result of this work is a report that quantitates and identifies every hydrocarbon in the sample. This information is critical, particularly in cases where the fraction is used as a feedstock for a chemical plant. As samples are processed, however, common chromatographic and sampling variability results in the need for intervention with manual review of chromatograms to insure precise and reproducible peak identification. Automated profile alignment, via correlation optimized warping, can largely eliminate run-to-run and instrument-to-instrument variability, greatly reducing the need for manual intervention. The alignment approach is not without weaknesses, however. The algorithm relies on the presence of a significant number of similar peaks in both the sample and the alignment target. If many sample peaks are not represented in the target, the alignment can be misleading. To insure an optimal alignment, the process can be repeated against multiple targets, retaining that result which produces the highest similarity between the aligned profile and the target. Alternatively, the alignment target can be chosen by an independent multivariate test, such as SIMCA, from an evaluation of the unaligned chromatographic profiles. After the alignment and DHA steps have been completed, it may be useful to perform another multivariate prediction on the DHA report to confirm the original material identity.

This multistep approach can be incorporated into routine analysis with the result that DHA will be objectively analyzed and consistently interpreted. Examples from different processed fuel types will illustrate the methodology.

*Keywords:* gas chromatography, method development, PAH, pattern recognition
*Application code:* fuels, energy, and petrochemical
*Methodology code:* data analysis and manipulation

### TRACE ANALYSIS OF SULFONYLUREA HERBICIDES IN ENVIRONMENTAL AND BIOLOGICAL SAMPLES USING FULLY AUTOMATED ON-LINE SOLID-PHASE EXTRACTION AND LIQUID CHROMATOGRAPHY WITH MASS SPECTROMETRY DETECTION

***Maria Elena Y. Cabusas,* Timothy J. Devine, and Joseph P. McClory***

**Stine-Haskell Research Center, E.I. DuPont de Nemours & Co, Inc, 1090 Elkton Road, Newark, DE 19711, USA*

Sulfonylurea herbicides are widely used against a range of broad-leaved and grass weeds during crop production. The field usage is at low concentration, typically below 100 g ai/ha. Trace analyses of sulfonylurea herbicides in either environmental or biological samples are often difficult because of the widely varying properties of analytes, for example, polarity, solubility, and stability, which is compounded by the complexity of the matrices. Furthermore, when detection is by mass spectrometry, matrix effect problems (ie, suppression or enhancement) are often encountered so that two or more purification steps are necessary during sample preparation. This presentation will describe a systematic approach for the determination and quantitation of sulfonylurea herbicides at trace levels in environmental and biological samples. The discussion will focus on sample analysis using a fully automated on-line solid-phase extraction and liquid chromatography with mass spectrometry detection. Various types of extraction and purification techniques will also be included in the discussion.

*Keywords:* environmental/biological samples, liquid chromatography/mass spectroscopy, solid-phase extraction
*Application code:* agriculture
*Methodology code:* liquid chromatography/mass spectrometry

### A NEW AIR SAMPLING TECHNIQUE FOLLOWED BY ON-LINE DESORPTION AND LCMS ANALYSIS

***Carlo Crescenzi,* Carlsson Håkan, Davide Tamburro, and Johanna Tollbäck***

**Arrhenius Laboratory of Natural Sciences, Stockholm University, Stockholm S-106 91, Sweden*

Interest and demand for air analysis has increased due to the number and diversity of pollutants presented in ambient air. More than one sampling device may be required to assess a group of target compounds having dissimilar chemical properties. In this presentation the use of SPE membranes as universal sorbent material for organic pollutants and particulate matter is reported.

A major advantage of SPE membranes in comparison to conventional SPE cartridges arises from their higher permeability and surface area-to-volume ratios which allow much higher sampling flow rates. SPE membranes are mainly used in water and biological fluids analysis; in the literature few reports using SPE membranes for air sampling have been published. The main goal of this study was to evaluate commercially available SPE membranes for environmental and occupational air analysis. The capability of Empore disk membrane in sampling particulate matter has been deeply investigated. Recoveries were calculated by analyzing selected PAHs in NIST certified materials by GC-MS. In the case of phosphate esters flame retardant after the sampling procedure, the analysis was performed using on-line desorption and extraction with LC mobile phase prior to MS detection. The use of on-line SPE-LC/MS greatly simplifies sample treatment and reduces the analysis time. Further, the risks of losses and contamination of the samples are reduced. MS detection generates confident identification of the target compounds.

During the air sampling procedure no relevant losses were observed after 24 hours ($16\,m^3$) even for the most volatile compound, that is, trimethylphosphate used in this investigation. Complete desorption was observed for all the organophosphate esters. The effects of particulate matter when using Empore disks for real samples have been evaluated in a comparative study using ultrasonication, microwaves, and temperature- and pressure-assisted extraction. The use of LC-MS allows the analysis of target compounds having a wide range of polarity. Finally the use of Empore disks allows an easy and safe way to store air samples.

*Keywords:* accelerated solvent extraction, environmental air, liquid chromatography/mass spectroscopy, on line
*Application code:* environmental
*Methodology code:* liquid chromatography/mass spectrometry

### PICOGRAM PER LITER LEVEL DETERMINATION OF ESTROGENS IN NATURAL WATERS AND WATERWORKS BY A NOVEL, FULLY AUTOMATED ON-LINE SOLID-PHASE EXTRACTION-LIQUID CHROMATOGRAPHY-ELECTROSPRAY TANDEM MASS SPECTROMETRY METHOD

***Damià Barceló,* Maria J. Lopez de Alda, and Sara Rodriguez-Mozaz***

**IIQAB-CSIC, Jordi Girona 18-26, Barcelona 08034, Spain*

The present work describes a novel, fully automated method, based on on-line solid-phase extraction-liquid chromatography-electrospraytandem mass spectrometry (SPE-LC-ESI-MS-MS), which allows the unequivocal identification and quantification of the most environmentally relevant estrogens (estradiol, estrone, estriol, estradiol-17-glucuronide, estradiol-17-acetate, estrone-3-sulfate, ethynyl estradiol, and diethylstilbestrol) in natural and treated waters at levels well below those of concern (limits of quantification between 0.02 and 1.02 ng/L).

Water samples (250 mL) are preconcentrated in PLRP-s cartridges. Further on-line chromatographic analysis is performed with a reversed-phase Purospher STAR-RP-18e analytical column ($125 \times 2$ mm, 5 micrometer particle diameter, from Merck, Darmstadt, Germany) using gradient acetonitrile/water as mobile phase. MS-MS detection is performed in the selected reaction monitoring (SRM) mode with an electrospray interface operating in the negative ionization (NI) mode, under time-scheduled conditions. Two different SRM transitions, one for quantitation and another one for confirmation, are monitored per compound. The method is highly precise, with relative standard deviations varying between 1.43% and 3.89%, and accurate (recovery percentages > 74%). This method was used to track the presence and fate of the target compounds in a waterworks and to evaluate the removal efficiency of the treatment processes applied. Only estrone and estrone-3-sulfate were detected in the river water used as source (at 0.68 and 0.33 ng/L, resp). After progressive removal through the various treatment steps, none of them was detected in the finished drinking water. In addition to selectivity, sensitivity, repeatability, and automation (up to 15 samples plus six calibration solutions and one blank can be analyzed in an unattended way), this technique offers high throughput (analysis time per sample is 60 minutes), low time and solvent consumption, and ease of use.

### ACKNOWLEDGMENTS

This work has been supported by the commission of the European communities (projects ARTDEMO EVK1-CT-2000-00045 and STAMPS EVK1-CT2002-00119), Ministerio de Ciencia y Tecnología (BQU-2002-10903-E), and the I3P Program (cofinanced by CSIC and the European Social Funds). Spark Holland is acknowledged for the gift of SPE cartridges. Maria José López de Alda acknowledges her Ramon y Cajal contract from the Spanish Ministry of Science and Technology.

*Keywords:* environmental/water, liquid chromatography/mass spectroscopy, on line, tandem mass spectrometry
*Application code:* Environmental
*Methodology code:* liquid chromatography/mass spectrometry

### DEVELOPMENT OF A SCREENING ANALYSIS FOR A WIDE RANGE OF POPs USING GCXGC-ECD

***Frank L. Dorman,* Jack Cochran, Robert Grimminger, Eric J. Reiner, Paul D. Schettler, and Leslie A. Vogt***

**Restek Corporation, 110 Benner Circle, Bellefonte, PA 16823, USA*

Several researchers have reported the development of various analytical methods for the analysis of the different classes of POPs over the last several years. Additionally, GCxGC-TOFMS has been investigated as a possible alternative to more classical analyses for compound classes such as organochlorine pesticides, PCBs, dioxins, and furans. While GCxGC coupled to mass spectrometry may come closer to the ultimate analysis technique for these compounds, the development of an extract-screening method would allow for the determination of which extracts might need additional characterization by either a GCxGC-TOFMS analysis or one of the various classical analytical methods like GC-HRMS. This presentation will discuss the need for the development of this method and the compound classes that are under current investigation. Finally, this presentation will show the current progress of this analytical method that is being developed.

*Keywords:* chromatography, environmental, gas chromatography, separation sciences
*Application code:* environmental
*Methodology code:* gas chromatography

### DEVELOPMENT OF INTEGRATED MICROCHIP INTERFACE TECHNOLOGY FOR AUTOMATED PROCESSING

***Jerome P. Ferrance,* A. Bruno Frazier, Christelle Guillo, Ki-Ho Han, and James P. Landers***

**University of Virginia, Mccormick Road, PO Box 400319, Charlottesville, VA 22904, USA*

One of the major challenges in moving microdevices out of the research laboratory and into analytical and clinical laboratories is developing methods for reliably interfacing the macro environment with the microfluidic architecture of the device in a manner which allows automation. The use of pneumatically activated valves in an integrated system allows automated control of fluid movement within the device. Control of external systems that generate the motive force for different solutions must also be provided, if multiple solutions are to be infused serially or in parallel through select regions of the device. In the design presented, the on-chip valving, utilized to control the flow within the microdevice, and the external fluid connection mechanism are both incorporated into a polymer interface that is independently fabricated then sealed to the glass microdevice. Evaluation of the interface and interface bond showed that they were unaffected during the various sample processing steps required on an integrated device designed for clinical DNA applications.

For this work, the microdevice/interface was designed specifically for use in the extraction of DNA from biological samples. DNA extraction is performed using a solid-phase extraction method that requires sequential flow of load, wash, and elution solutions through the extraction bed. The extraction bed is fabricated using silica beads held in place using a sol-gel matrix. Placement of the solid phase in the glass device can be performed before or after bonding with the interface, with the bead filling step playing a significant role in the design of the device. Fluid control has also been integrated into the outlet region of the SPE chamber. Integration of the SPE functionality into a multiprocessing device would require control of the load and wash solutions so that they are not passed into subsequent processing regions on the device. A control structure and appropriate channels were therefore incorporated into the design such that the load and wash solutions are directed to a waste reservoir as they exit the SPE bed while the eluted material is passed into a second chamber for collection or further processing. DNA extraction on this device, and subsequent PCR amplification of the purified DNA are presented to show the utility of the design with biological samples.

*Keywords:* lab on a chip/microfluidics, sample preparation, solid-phase extraction, SPME
*Application code:* proteomics and genomics
*Methodology code:* microfluidics/lab on a chip

### MICROANALYTICAL SYSTEMS BY COMPOSING MICROFLUIDIC DEVICES, DETECTION DEVICES, AND CONTROL SYSTEMS

***Manabu Tokeshi***

*Kanagawa Academy of Science and Technology, Ksp East 307 3-2-1 Sakado, Takatsu, Kawasaki, Kanagawa 213-0012, Japan*

Recently, there has been great interest in microfluidic devices. These devices have many advantages, including simplified operations, shortened analysis time, and reduced sample, reagents, and waste quantities. These advantages are desirable for applications in a variety of fields such as (bio) analytical chemistry, clinical diagnosis, and chemical manufacturing. In the past decade, many researches have been reported about microfluidic devices. However, most of these reports were focused on the microfluidic devices themselves. In order to realize microanalytical systems, miniaturization of all components composing the systems is necessary. Especially, miniaturization of detection devices is very important.

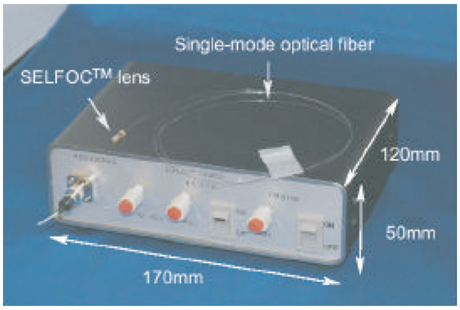

Figure 11

Making the most use of the advantages necessitates highly sensitive detection methods because the quantities of detected targets are very small. We have developed a thermal lens microscope as a highly sensitive detection tool. Although this has good performance, the size is large compared with the microfluidic devices. Very recently, we developed a micro thermal lens detection device based on an optical fiber and SELFOCTM micro lens (Figure 11). The performance of this device was compared with the thermal lens microscope which can determine a single-molecule level of nonfluorescent molecules under optimum conditions. At present, we are in the progress of developing microanalytical systems in which micro thermal lens detection device and all the other components are incorporation. We will show a few examples of microanalytical systems such as a chip-based enzyme-linked immunosorbent assay (ELISA) system and a bioassay system.

*Keywords:* bioanalytical, lab on a chip/microfluidics
*Application code:* bioanalytical
*Methodology code:* microfluidics/lab on a chip

### PRECONCENTRATION, EXTRACTION, SEPARATION, SPECIATION, AND TRACE DETERMINATION OF ARSENIC WITH CALIX(6)-FULLERENE-C60-61-METHANOFORMOHYDROXAMIC ACID

***Jigar J. Shah***

*Department of Chemistry, C.U. Shah Science College, Ashram Road, Ahmedabad, Gujarat 380014, India*

A simple, selective and rapid method for liquid-liquid extraction, separation, preconcentration, speciation, and simultaneous trace determination of arsenic(II) and arsenic(V) with calix(6)-fullerene-C60-61-methano-formohydroxamic acid is described and extracted at pH5 in dichloromethane and determined with a spectrophotometer at microgram level in industrial samples. The extract is directly inserted to plasma for ICP-AES measurement at nanogram level in environment arsenic(V) and reduced to arsenic(III) with hydroxylamine and preconcentrated in dichloromethane. The effect of pH, diverse ions, and reagent concentration were studied. The arsenic(III), arsenic(V), and total arsenic concentration was determined simultaneously in standard, food, biological, and environmental samples.

*Keywords:* atomic absorption, environmental/biological samples, extraction, plasma emission (ICP/MIP/DCP/etc)
*Application code:* environmental
*Methodology code:* separation sciences

### RAPID SPECIATION OF CHROMIUM IN AQUEOUS SAMPLES BY COLORIMETRIC SOLID-PHASE EXTRACTION

***James S. Fritz,*** [*] ***Marc D. Porter, and Steven A. Steiner***

[*] *Microanalytical Instrumentation Center and USDOT Ames Laboratory, Iowa State University, Ames, IA 50011, USA*

A fast and accurate method is described for the selective determination of chromium (III) and chromium (VI) in aqueous samples. Only simple, portable equipment is employed and the analyses can be performed on site. A measured volume of a sample in a syringe is passed through a series of membrane extraction disks contained in a plastic holder. Chromium (VI) is retained on one membrane extraction disk and chromium (III) is extracted simultaneously onto the second disk. Immediately following the extraction, the diffuse reflectance spectrum of the top surface of each membrane disk is measured by a portable instrument and the concentration of each chromium analyte is determined from calibration plots prepared with known standards. Chromium (VI) is determined with excellent selectivity in the approximate range of 0.1 to 10 mg/L in the original sample, and chromium (III) is measured in the range of 1 to 50 mg/L.

*Keywords:* elemental analysis, environmental/water, solid-phase extraction, speciation
*Application code:* environmental
*Methodology code:* separation sciences

### MICROFLUIDICS AND SINGLE-MOLECULE DETECTION: A LOGICAL CHOICE FOR REAL-TIME REPORTING OF BIOMARKERS

***Steven A. Soper***

*Department of Chemistry, Louisiana State University, 232 Choppin Hall, Baton Rouge, LA 70803-1804, USA*

In this post-genomic era, new genes are constantly being discovered and their association with certain diseases elucidated to provide information on either the patient's susceptibility to that disease (preventative medicine) or the course of treatment for that patient (personalized treatment). As an example, the new drug gefitinib (Iressa) can be used to treat nonsmall-cell lung cancers. But only about 10% of patients respond to Iressa and those that respond harbor mutations in the tyrosine kinase domain of the epidermal growth factor receptor gene. This and other examples in cancer biology clearly point to the need for obtaining detailed molecular definitions of tumors for determining the appropriate chemotherapeutic drug regimen or to decide which tissues to remove in sensitive areas, such as the head and neck, during surgical procedures. Real-time reporting of molecular profiles can potentially provide important information to the surgeon during the course of a procedure. Merging microfluidics with single-molecule detection (SMD) is particularly attractive when implemented in genotyping examples, since it allows removing some sample preprocessing steps, significantly reducing analysis time. In this presentation, our work in developing polymer-based microfluidic devices for the detection of low abundant point mutations in certain gene fragments (K-*ras* oncogenes) using single-molecule detection (SMD) will be specifically addressed. This presentation will entail a discussion of our fabrication methods for preparing high-throughput polymer-based microfluidic systems, integrating passive optical elements to these devices and near-IR fluorescence readout for SMD. In addition, development of spectroscopic discrimination techniques appropriate for SMD to identify rare point mutations using molecular beacon technology and FRET will be discussed.

*Keywords:* biotechnology, fluorescence, lab on a chip/microfluidics
*Application code:* bioanalytical
*Methodology code:* fluorescence/luminescence

### CONTINUOUS SINGLE-MOLECULE DETECTION WITHOUT IMMOBILIZATION OR PHOTON COUNT STATISTICS OF INTENSITY TRACES: A NEW PERSPECTIVE

***Zeno Foldes-Papp,*** [*] ***Masataka Kinjo, Mamoru Tamura, and G. P. Tilz***

[*] *Clinical Immunology and Jean Dausset Laboratory, Graz Medical University, Auenbruggerplatz 8, Graz A-8036, Austria*

Many models of molecular interactions are described at the single-molecule level, although our knowledge on structure and dynamics primarily comes from many-molecule systems. For example, amount and fraction of single, individual erroneous oligonucleotide and DNA sequences were quantified by bulk measurements in crude products of chemical and enzymatic multistep syntheses (Z. Foldes-Papp, et al., "Fractals for multicyclic synthesis conditions of biopolymers. Examples of oligonucleotide synthesis measured by high-performance capillary electrophoresis and ion-exchange high-performance liquid chromatography," *J. Chromatogr. A*, vol. 739, no 1–2, pp. 431–447, 1996, Z. Foldes-Papp et al., In: Losa GA, Merlini D, Nonnenmacher TF, and Weibel ER, eds. *Fractals in Biology and Medicine*, vol. II, Massachusetts: Birkhauser; 1997: pp. 238–254, and Z. Foldes-Papp, et al., "The analysis of oligonucleotide preparations by fractal measures," *Biopolymers*, vol. 45, pp. 361–379, 1998).

For the first time an experimental and theoretical framework has been developed that is called the "selfsame single-molecule regime" (Z. Foldes-Papp, U. Demel, and G. P. Tilz, "Ultrasensitive detection and identification of fluorescent molecules by FCS: impact for immunobiology," *Proc. Natl. Acad. Sci. USA*, vol. 98, no. 20, pp. 11509–11514, 2001, Z. Foldes-Papp, et al., "Theory of measuring the selfsame single fluorescent molecule in solution," *Pteridines*, vol. 13, pp. 73–82, 2002, Z. Foldes-Papp, U. Demel, W. Domej, and G. P. Tilz, "A new dimension for the development of fluorescence-based assays in solution: from physical principles of FCS detection to biological applications," *Exp. Biol. Med.*, vol. 227, no. 5, pp. 291–300, 2002, Z. Foldes-Papp, et al., "A new concept for ultrasensitive fluorescence measurements of molecules in solution and membrane: 1. Theory and a first application," *J. Immunol. Meth.*, vol. 286, pp. 1–11, 2004, Z. Foldes-Papp, et al., "A new concept for ultrasensitive fluorescence measurements of molecules in solution and membrane: 2. The individual immune molecule," *J. Immunol. Meth.*, vol. 286, pp. 13–20, 2004, and Z. Foldes-Papp, G. Baumann, U. Demel, and G. P. Tilz, "Counting and behavior of an individual fluorescent molecule without hydrodynamic flow, immobilization, or photon count statistics," *Curr. Pharm. Biotechnol.*, vol. 5, no. 2, pp. 163–172, 2004). With these algorithms, it is possible to distinguish between the observation of single molecules one by one and the observation of one and the same single individual molecule at a time without immobilization, hydrodynamic flow, or photon count statistics. Continuous observation of an individual molecule may also be performed up to hours. Single-phase single-molecule fluorescence auto- and cross-correlation spectroscopy (SPSM-FCS) offers a panel of tools for biomedical applications.

The novel experimental strategy of disease gene identification with unamplified genomic DNA in a solution that is based on special "intelligent" two-color hybridization probes and fluorescence cross-correlation spectroscopy is first presented (Z. Foldes-Papp, et al., 2005). The data will allow a distinction between underlying haplotypes and, ultimately, between high-risk and low-risk alleles at femtomolar allele concentrations and less without amplification or transcription.

*Keywords:* biomedical, biotechnology, data analysis, fluorescence
*Application code:* biomedical
*Methodology code:* data analysis and manipulation

### MODEL VALIDATION IN CHEMICAL IMAGES PRODUCED BY MULTIVARIATE OPTICAL ELEMENTS

***Ryan Priore* and Michael L. Myrick***

**Department of Chemistry & Biochemistry, University of South Carolina, 631 Sumter Street, Columbia, SC 29208, USA*

Imaging multivariate optical elements (IMOEs) combines digital imaging and predictive spectroscopy to produce single-shot concentration or classification images. These IMOEs are thin-film interference filters that enable simple instruments to measure the magnitude of a spectral regression vector in a defined analyte/interferent group. Previous work in our laboratory has shown IMOEs to successfully produce chemical images, but the IMOE predicted analyte concentration has never been validated for samples outside of the calibration model or analyte/interferent group. This presentation summarizes the validation techniques for using imaging multivariate optical elements in the near infrared (NIR).

*Keywords:* chemometrics, imaging, spectroscopy, validation
*Application code:* validation
*Methodology code:* chemometrics

### MULTIVARIATE SPECTRAL CALIBRATION USING REGULARIZATION WITH SMOOTHING

***Forrest R. Stout* and John H. Kalivas***

**Department of Chemistry, Idaho State University, Pocatello, ID 83209, USA*

When the methods of partial least squares (PLS), principal component regression (PCR), ridge regression (RR), and others are used to develop multivariate spectral calibration models, the data is typically used as it is (raw). Sometimes derivatives and other approaches are used to enhance the spectroscopic information prior to modeling. Commonly, the goal in calibration is to obtain a model (a vector of regression coefficients) that provides a good fit to the data. Another aim is to obtain a regression vector that does not display too much rapid fluctuations (noise is reduced and a smooth regression vector is desired). This paper shows that this can be accomplished by using Tikhonov regularization in general form with the vector 2-norm where a compromise is reached between the degree of fit (bias) and a roughness penalty with respect to variance (the bias/variance tradeoff). Our previous work has demonstrated the advantages of using Tikhonov regularization in standard form (no roughness penalty) with the 2-norm compared to PLS and PCR. The new work presented extends this approach to incorporate smoothing into the model building process. Results are compared with PLS, PCR, and RR using spectroscopic datasets.

*Keywords:* calibration, chemometrics, optimization, spectroscopy
*Application code:* general interest
*Methodology code:* chemometrics

### BE PREPARED FOR THE UNEXPECTED: FAST AND FLEXIBLE CHROMATOGRAPHY DATA MINING USING A COMBINATION OF ADVANCED SEARCHING AND REPORTING TOOLS

***Jozsef Medve,* Frank Arnold, and Wulff Niedner***

**Dionex Corporation, Dionex Softron GmbH Dornierstrasse 4, Germering 82110, Germany*

Modern chromatography data systems (CDS) store data in databases. A large amount of data is available that potentially contains valuable information in addition to the initial purpose. Some CDS limit the search to the data that has been explicitly stored in the database, that is, one has to anticipate all future needs well ahead. A better solution is when the CDS also allows searches among instantly calculated results. Combining these advanced search capabilities with flexible and easy-to-use reporting and charting tools facilitates finding the “gold” in the “mine,” that is, finding valuable results, discovery of previously unknown correlations, and creating clear and concise summaries from the vast amount of data. During this presentation you can view some application examples and see how fast and easy this can be done with the right tools.

*Keywords:* chromatography, database, data mining, scientific data management
*Application code:* general interest
*Methodology code:* data analysis and manipulation

### MULTIVARIATE THREE-WAY DATA ANALYSIS TO IDENTIFY SPECIFIC PLANTS IN THE DIETS OF FREE-RANGING HERBIVORES

***Charlotte N. Sisk***

*Department of Chemistry and Biochemistry, New Mexico State University, PO Box 721, Mesilla Park, NM 88047, USA*

The issue of identifying specific plant species in the diets of free-ranging herbivores in the local and global community is of much interest in the context of three-way analysis.

The identification process was analyzed using chemometrics methods applied to the excitation emission fluorescence of a number of plant species. We were able to develop an integrated approach to perform tasks such as multidimensional analysis, process modeling, and statistical process decomposition. A description is given to the advantages of combining several chemometrics tools (parallel factor analysis (PARAFAC), multiway principal component analysis, PARAFAC II, and GRAM) and the principal benefits associated with each strategy for the elucidation of diet composition. Also a general overview of the principal achievements and limitations of the techniques used within the presented methodology is depicted. It is illustrated how three-way principal components analysis as the appropriate generalization of conventional principal component analysis may serve as a powerful method for classification of specific plant species in diets of free-ranging herbivores using the excitation-emission matrices from fluorescence spectroscopy from different species. The factors found appear to correspond to the causal influences manipulated in the experiment, revealing their patterns of influence in all three ways of the data. Several generalizations of the parallel factor analysis model are currently under development, including ones that combine parallel factors with Tucker-like factor “interactions.” In the research, necessary and sufficient conditions for global and local solutions to plant identification are being derived. The results of these investigations will be presented and the implications of the application of these data analysis tools for the identification of specific noxious weeds within the diets of free-ranging cattle will be discussed.

*Keywords:* chemometrics, fluorescence, materials characterization, statistical data analysis
*Application code:* agriculture
*Methodology code:* data analysis and manipulation

### VALIDATING BROWSER-BASED APPLICATIONS: DEBUNKING THE MYTHS

***Fernando Casanova***

*LabVantage Solutions, Inc, 1160 Rt 22 East, Bridgewater, NJ 08807, USA*

Many “validation traditionalists” have specific concerns about utilizing a browser interface for client access to validated environments. In this poster presentation, Fernando Casanova, LabVantage Vice President of Quality, will explain that most of these concerns are based on myths, which he will debunk. Casanova will then proceed to explain the many benefits of browser-based validation over traditional methods. Myths include the following. (1) Browser-based applications cannot be validated. (2) Browser validation concerns are limited to regulated industries. (3) Browser security is less effective than rich client security. (4) A browser-based interface provides little benefit over a rich client interface. (5) Data being transmitted from a browser is not secure. (6) 21 CFR Part 11 compliance cannot be achieved with a browser-based interface. (7) More functionality is available with a traditional rich client/server configuration than with a browser-based configuration. (8) Validation of a browser under the auspices of 21 CFR will not satisfy EMEA regulations.

*Keywords:* LIMS, quality, quality control, validation
*Application code:* quality
*Methodology code:* others

### APPLICATION OF NEAR-INFRARED AND MULTIVARIATE CALIBRATION FOR DETECTION OF INFESTATION IN SMALL FRUIT

***Boyan Peshlov[*] and Darrell W. Donahue***

[*]*University of Maine, 5737 Jenness Hall, Room 200, Orono, ME 04469, USA*

The ability to detect blueberry maggots in berries by using near-infrared spectroscopy (NIRS) could greatly affect insect pest management, blueberry production, and cultural practices. NIRS coupled with chemometric data analysis was examined as a method for detection of maggot infestation in blueberries. Laboratory-raised flies were caged with stems containing mature blueberry fruit to promote artificial inoculation. The individual berries were scanned in diffuse reflectance mode using three different NIR spectrometers. After scanning, all berries were examined under a light microscope to determine larvae presence. Spectral subtraction of averaged data of infested minus non-infested blueberries showed some unique differences similar to the ones found by other researchers working on internal insect infestations of fruit. These differences found in the amino group bands (1300–1700 nm) are particularly interesting because of the chemical nature of the blueberry and the larvae. Multivariate calibration models including PLS regression were built using NIR spectra and reference microscopic data. Results from three seasons were compared and an infestation prediction rate of approximately 87% was obtained. However, model prediction rate slightly improved by applying appropriate pre-processing techniques which correct for light scattering effects due to size and color variation. Data from the 2003 and 2004 seasons were used for estimating season and field variation as well as validating prediction models.

### ACKNOWLEDGMENTS

This research was supported by the USDA and the Wild Blueberry Commission of Maine.

*Keywords:* biological samples, chemometrics, detection, near infrared
*Application code:* quality
*Methodology code:* near infrared

### PROFICIENCY TESTING SCHEME FOR QUALITY CONTROL IN LABORATORIES

***Adriana C. Ferreira* and Sérgio L. Motta***

**SENAI-CETIND, Av Luiz Tarquínio, 938 Aracuí, Lauro De Freitas 42700-000, Brazil*

The aim of the present paper is to evaluate some results of an interlaboratory study of metals in water matrix. In an effort to improve laboratories performing metals and anions analysis in water, the SENAI-CETIND (Brazilian Industrial Learning Service-Industrial Technological Center) has implemented a proficiency testing scheme program since 2001. The PT scheme is indicated to all laboratories that are interested in testing their analytical performance. The conduction of our external quality assurance system is based on the requirements of ISO Guide 43 and thus also corresponds to the international criteria for proficiency testing in chemical laboratories. Currently about 30 laboratories are participating per round.

The water samples were prepared by gravimetric formulation. They consist of synthetic samples, which are assembled from ultrapure water, pure reagents, and pure standard chemicals from NIST (National Institute of Standard & Technology). The estimation of the assigned value was obtained by the consensus value among participants using the robust statistics (median), which is less vulnerable to outliers. Two levels of a test material containing metals were prepared. Reference concentrations were calculated by a combination from the gravimetric data of sample preparation and results obtained from validated methods usually used to measure the analytes. In each series correctness of the reference values, homogeneity and stability of the PT samples were checked.

The participants were evaluated by three methodologies: z-score, En numbers, and confidence ellipse plot. In this proficiency testing most laboratories obtained good results regarding accuracy (z-score and ellipse). For all analytes of Group G1 (Ag, Ca, Cd, Co, Cr, Cu, Fe, Mg, Mn, Na, Ni, Pb, and Zn), the percentage of satisfactory results was superior to 63%.

Concerning Hg this percentage was 19%. The percentage of satisfactory results as normalized error was superior to 50%. This shows that many of the laboratories are calculating the uncertainty of the measurements in a proper way. However, it was observed that the standard deviations of the measurements are not being taken into account in the calculations of the uncertainty of some laboratories. This proficiency testing program must continue to be free of charge. It has to be considered as an education tool.

*Keywords:* chemometrics, environmental/water, quality, trace analysis
*Application code:* quality
*Methodology code:* atomic spectroscopy/elemental analysis

### ELECTRONIC NOSE FOR WAX FRAGRANCE THROW ASSESSMENTS

***Vincent Schmitt,* Laura Hein McElduff, and Joey Viljoen***

**Alpha M.O.S., 20 Avenue Didier Daurat, Toulouse, NJ 31400, France*

The customer's perception of a candle's smell is one of the main selling features of a candle, so evaluating the fragrance throw is important, both when the candle is not burning ("cold" throw) and when the candle is burning (the "hot" throw from the melt pool).

A first investigation in which fragrance throw from different candle blends was evaluated with an electronic nose indicated that a blend's composition can affect the fragrance throw. In a second study, a wider variety of blends were evaluated by I.G.I. using the electronic nose technology in order to expand on this work. Candle smell feature was evaluated via two approaches:

(i) human odor panel at French Color and Fragrance Co,
(ii) electronic nose—Alpha M.O.S. electronic nose (Fox 3000 model).

This abstract will show how a multisensor array system can evaluate qualitative fragrance throw and smell the intensity of candle blends. The results will be presented with different mathematical models: principal component analysis (PCA), discriminant factorial analysis (DFA), and statistical quality control (SQC).

The Alpha M.O.S. electronic nose proved to be an objective and fast tool for fragrance throw assessment with an excellent correlation with sensory panel scores.

*Keywords:* headspace, quality control, quantitative, sensors
*Application code:* quality
*Methodology code:* others

### AN APPROACH TO VALIDATING BARCODE PRINTERS, SCANNERS, AND LABELS

***John L. Peterson***

*DCSS, Inc, 329 Scottsdale Drive, Wilmington, NC 28411, USA*

The use of barcode technology as a tool for improving accuracy and efficiency in the laboratory is growing rapidly. While this technology has been in use for many years, implementing it in the laboratory involves taking special measures to assure label integrity and usage are properly controlled. For example, will the label remain adhered to the container and will the printing remain legible given liquids encountered in the lab and extremes of storage conditions?

This control is achieved through a validation effort that addresses not only the software using the barcodes, but also the capabilities of the printer, the ink, or ribbon used, and the label stock. This presentation will outline examples of requirements that need to be considered and tests that can be used to challenge those requirements.

*Keywords:* computers, laboratory informatics, software, validation
*Application code:* validation
*Methodology code:* laboratory informatics

### THE CRITICAL DOCUMENT FOR COMPUTER VALIDATION

***John Read***

*Thermo Electron Corporation, 18 Commerce Way, Suite 5000, Woburn, MA 01801, USA*

Data system validation involves people, actions, protocols, scripts, testing, and a very large number of documents. A thorough validation plan can ensure successful validation. The various elements of a detailed validation plan will be discussed, as will many of the various activities and other documents required.

*Keywords:* pharmaceutical, standards
*Application code:* validation
*Methodology code:* others

### AUTOMATING THE INSTALLATION QUALIFICATION/OPERATIONAL QUALIFICATION FOR MULTIVENDOR INSTRUMENTATION WITH A LABORATORY CONTENT MANAGEMENT SYSTEM

***Soheil Saadat* and Edward C. Long***

**Scientific Software, Inc, 6612 Owens Drive, Pleasanton, CA 94588, USA*

Regulated laboratories face a daunting challenge to ensure that the laboratory conforms to accepted validation standards. This is particularly challenging with the wide range of multivendor instrumentation that is prevalent in most laboratories. While these tasks can certainly be performed manually by the end user or through outside service organizations and are commonly provided by the original instrument vendor, there is great interest in automating the IQ/OQ process with a single system and having it applied to instrumentation from different manufacturers. An automated, IQ/OQ Validation software package based on multivendor instrumentation with full-featured Enterprise Content Management software will be described. Not only does this provide extensive support for a wide variety of different instrumentation from different manufacturers, but its management of electronic content from the IQ/OQ provides a novel way for laboratories to automate much of the preventive maintenance concerns with instruments.

*Keywords:* automation, data mining, laboratory informatics, validation
*Application code:* validation
*Methodology code:* laboratory informatics

### A SEAMLESS SYSTEM FOR AUTOMATED COMPUTER DATA BACKUP

***Jay S. Osborne,* Roger K. Gilpin, and Joseph G. Solch***

**College of Science and Mathematics, Wright State University, Dayton, OH 45435, USA*

Multi-operating computer system environments that are typical in science-based businesses and research facilities present a series of unique problems. Common challenges include: (1) integrating automatic backup of data from operating systems from different vendors; and (2) adapting to rapidly changing equipment and workgroup configurations. This paper presents the successful implementation of an inexpensive Linux-based system to provide rugged and automatic network data backup that does not require the addition of any software to the client machines. Designed to be instantly deployable in changing laboratory and workgroup situations, it only requires the user to define a specially formatted network share name for resources that need automated backup. Backed up data is then available via read-only shares on the backup computer. A simple password protected web-based system is provided to permit the removal of data no longer required. Firewall services are included to protect the backup computer from any external attacks. Design concerns and the economics of the implementation and operation will be provided.

*Keywords:* computers, database
*Application code:* others
*Methodology code:* computers, modeling and simulation

### APPLICATION OF COMPUTER ALGORITHMS AS A TOOL IN THE ANALYSIS OF MASS SPECTRAL DATA

***Evaldo DeArmas***

*Thermo Electron Corporation, 1400 Northpoint Parkway, West Palm Beach, FL 33407, USA*

Gas Chromatography-Mass Spectrometry (GC-MS) or Liquid Chromatography-Mass Spectrometry (LC-MS) are analytical techniques that can generate large amounts of data. Computers are used both to control the analyzers and for data reduction. One of the problems in this analytical field is that the software used for data reduction does not take full advantage of the information contained in the data and a lot of it is mostly ignored. One of the more common problems in GC-MS is that of coeluting peaks and the ability of the data reduction algorithms to extract clean deconvoluted spectra that can then be used for analyte identification or spectral classification. Spectral classification is more involved and uses chemometric techniques to extract and reduce the dimensionality of the data. In this way it is possible to develop a visual representation of the data.

A more challenging problem is the ability to predict mass spectral fragmentation patterns or to develop mechanisms that show how fragmentations patterns are generated. This can be very helpful for MS/MS prediction and interpretation.

In this presentation, examples will be shown on how advanced computer algorithms can be used to deconvolute mass spectra of coeluting peaks, and to classify mass spectral data using principal component analysis. Also the ability to predict spectral patterns will be demonstrated with some examples.

*Keywords:* data analysis, data mining, gas chromatography/mass spectrometry, software
*Application code:* general interest
*Methodology code:* data analysis and manipulation

### LAB AUTOMATION AND THE HUMAN ELEMENT IN THE CONTINUING EVOLUTION OF THE QUALITY SYSTEMS PROGRAM

***Michael J. Herdlick***

*ATEL, 1776 Marion-Waldo Road, Marion, OH 43302, USA*

The world of the environmental laboratory is constantly evolving and changing its dynamic shape and structure. Environmental analysis is a competitive market driven by quicker turn around times, lower prices, extensive data packages, regulatory demands, and high quality. Although we will never remove the human element entirely from the laboratory, we can continue to improve how the laboratory functions through automation. Thus, the focus of this topic seeks to join the two basic entities together: analyst and technology, in order to evolve the laboratory into a more automated structure and to improve the quality system.

This presentation will be divided into a number of subtopics: (1) Management Techniques; (2) Organic Laboratory Concepts; (3) Inorganic Laboratory Themes; (4) Biological Topics; and (5) Quality Assurance Issues. Each of the above categories will be discussed in detail focusing on how the laboratory automation seeks to assist and improve the output and quality of the analytical data. Furthermore specific examples will be presented to demonstrate how the use of technology through networks, workstations, and workgroups can enhance this dynamic system. Particular emphasis is placed on meeting the demands of a working quality system, especially one that has been fueled by the National Environmental Laboratory Accreditation Conference (NELAC) along with other state regulatory programs.

This presentation will be beneficial for analysts in the laboratory as well as supervisors, senior managers, and quality assurance officers. Those in the environmental community understand that it is vital that the laboratory must continue to demonstrate its commitment to a quality system through automation even though we will always work with human beings who are subject to mistakes and improvements. It is the goal of this presentation to reflect and demonstrate on the positive avenues to pursue in order to meet the regulatory demands and client requirements.

*Keywords:* data analysis, environmental analysis, laboratory automation, quality control
*Application code:* environmental
*Methodology code:* computers, modeling and simulation

### CREATION AND UTILIZATION OF A COST-EFFECTIVE, SEARCHABLE DATABASE CONTAINING NEW AND LEGACY TOXICOLOGY AND CHEMISTRY GLP STUDIES AND DATA

***Denise B. Taylor,[*] Nichelle M. Oberson, George Polson, and Nicholas P. Skoulis***

[*]*Arch Chemicals, Inc, 350 Knotter Drive, Cheshire, CT 06410, USA*

Current legislation requiring registration or approval of antimicrobial actives used in material preservation (wood, building products) and antifouling products has resulted in an abundance of studies to determine the properties, health effects, safety, toxicology, and environmental fate of these substances. These studies (guideline and nonguideline) can be used to support multiple registration efforts worldwide (EPA, HSE, BPD, etc). Final reports often need to be submitted on short notice, in readable formats, with appropriate approvals and signatures intact. Although newer reports have been generated electronically and may even have electronic signatures, the majority exist as paper copies with original handwritten signatures. A cost-effective, yet comprehensive, solution to the preservation, organization, and retrieval of these reports was needed. A study of these issues found that although many elegant document management systems exist, readily available software can be used to create user friendly, effective, and versatile databases. Comprehensive searching of key substance attributes and retrieval of the actual documents via hyperlinks to PDF files created by scanning of the original documents is demonstrated. Where multiple document databases are connected, links between databases are easily implemented. Scanned documents are acceptable to most agencies and good quality images can be made from new and aged documents. Additionally, the entire database can be copied to CD and sent to offices that are not on the internal network or have very slow connections. Our work describes a database created on close to four hundred reports and the versatility of the search, retrieval, and report generation capabilities.

*Keywords:* database, GLP/GALP, sample and data management, scientific data management
*Application code:* regulatory
*Methodology code:* computers, modeling and simulation

### TECHNIQUES FOR DEVELOPING 21 CFR PART 11 COMPLIANT SOFTWARE PROGRAMS

***Eric Reffett***

*National Instruments, 11500 North Mopac Expwy, Austin, TX 78759, USA*

This paper describes how to validate a software application program with code-review, functional testing, and structural testing techniques. In addition, the paper shows how to use traceability to optimize validation time and effort. Finally, this paper will describe how to determine the scope of retesting a software program after making a change to the program and will give examples on how to manage requirements for software program development.

*Keywords:* computers, instrumentation
*Application code:* regulatory
*Methodology code:* data analysis and manipulation

### SOFTWARE PLATFORM SIMPLIFIES DEVELOPMENT OF ANALYTICAL INSTRUMENTATION

***Shelley Gretlein***

*National Instruments, 11500 North Mopac Expwy, Austin, TX 78759, USA*

As the complexity of analytical systems increases, so does the need for efficient design tools that enable vendors to build complete sophisticated systems. A complete software design tool must have the power and functionality expected of professional development languages, as well as the ability to create embedded control solutions that can handle the complexity of the entire instrument. This implies accessing and controlling multiple data acquisition devices, sensors, and actuators that must all work together in a tightly controlled and highly reliable fashion. These systems must also provide a professional user interface and graphical representation of the data for scientists. Finally, these systems must perform data analysis, storage, and presentation of the results in a variety of formats to suit individual customers' needs.

Accomplishing these tasks would typically require a large investment in building custom software and hardware components. This however is not practical, due to increasing demands to reduce system costs. Therefore, engineers must look for solutions to reduce the time and work required to develop analytical instruments. Graphical Development Environments provide easy-to-use tools for acquiring data from any sensor or hardware device, with the power of a programming language capable of performing the most complex analysis algorithms. Using these tools, engineers have the flexibility of creating professional user interfaces and deploy these applications to embedded real-time deterministic targets, as well as configurable FPGA and portable PDA devices, to providing highly-integrated systems at the lowest cost. Attend this session to learn more about these tools and how to use them to build high quality, low cost, analytical software systems.

*Keywords:* analysis, instrumentation, sampling, sensors
*Application code:* general interest
*Methodology code:* computers, modeling and simulation

### ACHIEVING A "PAPERLESS" LABORATORY WITH ENTERPRISE CONTENT MANAGEMENT

***Dale Young* and Edward C. Long***

**Scientific Software, Inc, 6612 Owens Drive, Pleasanton, CA 94588, USA*

The pressure to reduce paper flow, especially for analytical laboratories, remains a widespread goal of many companies. In some respects, the interest in 21CFR part 11 compliance is great with companies as part of an overall goal to become more paperless. Yet while reduction in manual paper communications is achievable in many lab operations, there must be no sacrifice in proper documentation, traceability in results, and workgroup collaboration and communication of the laboratory data.

Outside of the laboratory, many companies have embraced "Content Management" software in order to achieve a reduction in paper flow. For the most part, however, Laboratories have been slow to adopt a paperless mode of operation, partly because of the lack of software tools that can reduce paper flow while still manage the electronic information generated in a lab. The application of a new software framework that integrates laboratory instrumentation with an advanced enterprise content management software as it reduces paper flow will be discussed.

*Keywords:* informatics, lab management, laboratory informatics, scientific data management
*Application code:* laboratory management
*Methodology code:* laboratory informatics

### OPEN AUTOMATION IN A LABORATORY SOFTWARE FRAMEWORK

***Steve Miller* and Edward C. Long***

**Scientific Software, Inc, 6612 Owens Drive, Pleasanton, CA 94588, USA*

A new laboratory software framework has been developed to provide integration of scientific instrumentation and enterprise content management of electronic information. This software framework is based on a truly modular approach to the software components for instrument control, data acquisition, data analysis, printing, and other services so that rather than rely on a single vendor for providing programming applications, the framework will allow interested parties the opportunity to implement their own applications into the framework. The software architecture design, published API, and applications of these automation features will be discussed. In addition, automation through programmatic access, software dialogs, and interactions from the software framework to other 3rd party products will also be reviewed. This extensive layer of automation capabilities provides the software framework with a way to address current and future expansions in the laboratory while still controlling all the laboratory electronic content safely and securely.

*Keywords:* automation, laboratory automation, petrochemical, sample handling/automation
*Application code:* fuels, energy, and petrochemical
*Methodology code:* laboratory informatics

### 21 CFR PART 58: MANAGING PEOPLE AND PROCESSES: HOW LIMS HELPS IN COMPLIANCE WITH GOOD LABORATORY PRACTICE

***Fernando Casanova***

*LabVantage Solutions, Inc, 1160 Route 22 East, Bridgewater, NJ 08807, USA*

To ensure the quality of work done in the laboratory, federal regulations such as 21 CFR part 58 spell out quality checkpoints at various stages of the workflow. Among the requirements of 21 CFR part 58 are specifications for personnel qualifications, management responsibilities, roles of the study director and QAU, protocols, facilities, procedures, substances, systems, report contents and, record keeping. Maintaining consistent compliance throughout the organization can be challenging. In this poster presentation, Mr Fernando Casanova, a career-quality specialist, will discuss how a laboratory information management system (LIMS) can simplify the process by setting up workflows to perform the same steps in the same way. A LIMS tracks qualified personnel, assigns hierarchical roles to individuals, directs tasks automatically, manages protocols, provides data capture and accountability, sponsors e-sig product reviews, tracks test and substance results, creates reports, and records and maintains study data. LIMS also tracks qualified personnel by setting up tables describing details of what a person has been trained on, and sets expiration dates on the training. Thus, only people who are qualified to do something will be able to perform the procedure. Instead of using pencil and paper, a LIMS collates data, and prints out reports electronically. If an audit is required, the system can generate training records. A LIMS is flexible within the confines of the configuration. The software creates the tables used in generating reports to meet the requirements of the federal regulations. It enables the creation of a web page that combines the tables with other information.

*Keywords:* GLP/GALP, LIMS, quality, quality control
*Application code:* regulatory
*Methodology code:* laboratory informatics

### CONTROLLED REPORTS IN REGULATED LABORATORY ENVIRONMENTS

***Chaitanya Diwadkar* and Michael Wong***

**Applied Biosystems, 3833 North First Street, San Jose, CA 95134, USA*

Many laboratories require extensive controlled report execution (controlled report output) to supplement standard reporting functionality. In this presentation we will discuss, through customization of SQL*LIMS software from Applied Biosystems, how the software was extended to develop controlled reports whose reports use is tracked, whose output is secured, and whose output is electronically signed.

Our requirements demand four types of reports that are available to users of the system; untracked, tracked, controlled, and signed reports. Each type places certain restrictions as to how the output will be and what functionality it has. As you progress through the types, the restriction increases. The software's standard security was extended to verify that only privileged users can work with the appropriate output. Untracked reports have no restrictions. Tracked reports have additional history information attached. Controlled reports are stored in the database and can only be printed to the user's default assigned printer. And finally, signed reports can only be printed after they are electronically signed. There are other specific restrictions of each type that will be highlighted during the presentation.

Primarily four modules of the software were extended to provide such functionality. These will be highlighted during the presentation and each will be discussed in what their aspect is and how they fit into this custom functionality. Some of the highlights of such controlled reporting will be presented with actual PDF output. In addition, possible future enhancements will be discussed to perhaps extend this capability even further.

*Keywords:* lab management, laboratory automation, laboratory informatics, scientific data management
*Application code:* laboratory management
*Methodology code:* laboratory informatics

### MANAGING MULTIPLE INSTRUMENT TYPES AND EQUIPMENT MANUFACTURERS WITHIN THE REGULATED LABORATORY ENVIRONMENT

***Dan Furlano***

*Thermo Electron Corporation, 18 Commerce Way, Suite 5000, Woburn, MA 01801, USA*

Managers in regulated laboratories are routinely confronted not only with various types of instruments and equipment, but also instruments of similar types from different manufacturers. The issues that this complexity creates not only directly relate to laboratory productivity and instrument usage, but also with compliance related activities such as instrument qualification and documentation. Developing and implementing processes that can help increase productivity can be realized utilizing various methods including the following.

(i) Test instrument types using a consistent method. Test instruments in the environments in which they will be used on a regular basis without having to modify system configurations or data collection parameters to execute the tests.
(ii) Making documentation easily reviewable by internal quality assurance or regulatory agency representatives. This process eliminates errors and allows for easy comparisons between similar instruments. Review and approval of documentation as well as training is simplified.
(iii) Creating upper-level guide-line documents based on instrument types, that is, HPLC, and lower-level procedures that are make and model specific, that is, Thermo Electron Surveyor HPLC. This process is valuable for numerous reasons, especially in creating instrument methods that are transferable between makes and models of instrumentation.
(iv) Ability to test multiple instrument types to user specifications which will allow the instrument to be tested for its intended use and not manufacturer's specification that may or may not relate to the intended use. Instruments will all be tested to the same specifications not the individual manufacturer specifications.
(v) In today's competitive environment of mixed equipment and instrument types, along with manufacturers testing methods and documentation, procedures have become dispersed. This presentation illustrates the value of utilizing consistent methods for testing similar instrument types from different manufacturers.

*Keywords:* informatics, lab management, laboratory, laboratory informatics
*Application code:* laboratory management
*Methodology code:* laboratory informatics

### OPERATIONAL DATA MANAGEMENT: COMPLETING THE PROTECTION OF YOUR DATA

***Jim Jenkins***

*LabVantage Solutions, Inc, 1160 Route 22 East, Bridgewater, NJ 08807, USA*

Today's laboratories produce vast amounts of electronic data, and there is an urgent need to secure this valuable asset. There are too many occurrences—both in and out of regulated environments, where laboratories are failing to follow GxP guidelines on the management and security of this data. Frequently, laboratories do not perform backups, or they may intermittently copy raw data to CD's or file servers. What would happen if the hard drive on that instrument workstation failed? How do you know that you are working with the correct iteration of the data file(s)? Can you find the data you created today in 1, 2, or 5 years? Clearly, these problems are being regularly highlighted by internal and external auditors, and are not easily addressed by each department manager.

Operational data management systems confront these challenges by automating the movement of data from remote instrument computers to secure, centrally managed locations—with no manual input or additional work required of the scientist. Operational data management fills the void in the management of data—management of the data while it is live, allowing the data to be managed and made readily available in a manner that is transparent to the scientists both in the collection and dissemination of the data. This poster presentation will explore the role of operational data management systems in the laboratory data life cycle, and highlight the advantages gained using an operational data management system to complete the protection of your data.

*Keywords:* data analysis, laboratory automation, scientific data management, software
*Application code:* laboratory management
*Methodology code:* data analysis and manipulation

### ELECTRONIC NOTEBOOKS

***Patrick J. Kelly[*] and Robert D. Walla***

*[*]Synnestvedt & Lechner LLP, Suite 2600 Aramark Tower, 1101 Market Street, Philadelphia, PA 19107, USA*

Research records are a vital component to a corporation's intellectual property (IP). Given the large amounts of data generated in present day laboratory environments, there is increased interest in electronic laboratory notebooks (ELNs). ELNs can provide a paperless laboratory environment where large amounts of data can be created, shared, indexed, and archived. To satisfy a company's IP needs, an ELN

must be able to serve as an evidentiary source in a litigation/interference in the same manner as traditional laboratory notebooks. Unfortunately, there is no clear guidance from the courts or the US Patent Office on the requirements for an ELN. Our discussion will outline rational requirements for an ELN, given that a body of case law has yet to develop.

In determining the requirements for an ELN, we will look to federal regulations (21 CFR) and the business records exception to the hearsay rule. 21 CFR outlines the requirements for the drug industry relating to electronic records and signatures and provides fundamental guidance on how to ensure the security and reliability of electronic data. The hearsay rule exists to prevent unreliable out-of-court statements by declarants from improperly influencing the outcome of a trial or interference. One way in which ELNs may be introduced as evidence is by the "regularly kept records" exception to the hearsay rule. The teachings of 21 CFR and the business records exception to the hearsay rule will be discussed as guideposts for development of company rules for ELNs.

*Keywords:* lab management, laboratory, laboratory automation, laboratory informatics
*Application code:* laboratory management
*Methodology code:* laboratory informatics

### APPLICATION OF SIX SIGMA TO LIMS IMPLEMENTATION

***Donald Kolva*** **and Fred Rider***

*[*]Accelerated Technology Laboratories, Inc, 496 Holly Grove School Road, West End, NC 27376, USA*

A laboratory information management system (LIMS) that automates login, enhances data accessibility, incorporates a full chain of custody and a full audit trail, automatically tracking sample status, providing QA/QC functionality, automation of reporting and management of the vast array of laboratory data generated is one of the most important tools used within the laboratory. The degree of importance involved requires an organized and well thought out approach to its implementation.

One such approach is to apply the principles of Six Sigma to the process. The primary steps include define, measure, analyze, improve, implement, and control. This would start with a formalized effort to define both internal and external goals of the proposed LIMS. It should start with a full evaluation of performance of existing processes. What is the method currently used to document, store, and analyze the test data generated by the lab analysis processes? Both successes and failures need to be identified and the root causes of all deficiencies found. Once this is done, LIMS features can be selected which emulate the successful and mitigate or eliminate the deficiencies.

Once the initial evaluation is complete, the LIMS itself would need to be evaluated in terms of internal and external goals. Measurements of customer needs and specifications would have to be established. Evaluation of the LIMS processes vis-à-vis those specifications would then insure goals were met. Finally, a continuing evaluation program would be established to insure long-term needs are met.

*Keywords:* lab management, laboratory automation, laboratory informatics, LIMS
*Application code:* laboratory management
*Methodology code:* laboratory informatics

### THE SELECTION, INSTALLATION, AND IMPLEMENTATION OF A COTS LIMS AT THE BIRMINGHAM WATER WORKS AND SEWER BOARD

***Christine Paszko,[*] Desiree Alexander, Drusilla Hudson, Anton Jones, and David Schlabach***

*[*]Accelerated Technology Laboratories, Inc, 496 Holly Grove School Road, West End, NC 27376, USA*

As regulatory and reporting demands grew, the laboratory management team identified a need for an upgraded data management solution. Several commercially available systems were evaluated on several criteria including ease of use, standard tools and technology, flexibility, upgradeability, training program, support, and total cost of ownership. The major goals of the project included enhanced real-time sample tracking, improved QA/QC, automation of mundane tasks and data entry from instruments, as well as automated final analysis reporting (via e-mail, fax, and PDF).

The team worked with a consultant experienced in automation solutions to assist them in creating a specification document for the selection of a commercial off-the-shelf COTS LIMS (laboratory information management system) with modules including sample tracking, data entry, sample scheduling, QA/QC, electronic data entry, chemical inventory, resource management, time tracking, and customer relationship management. In addition, the laboratory sought a solution to share sample status, results, and PDF copies of reports with the entire organization via a secure, company-wide Intranet.

This paper will also review associated automation enhancements including bar-coded labels, scanners, instrument integration, and automated reporting.

*Keywords:* lab management, laboratory automation, LIMS
*Application code:* laboratory management
*Methodology code:* laboratory informatics

### KEY ELEMENTS OF A LABORATORY INFORMATION MANAGEMENT REQUEST FOR PROPOSAL

***Kim Paszko***

*Accelerated Technology Laboratories, Inc, 496 Holly Grove School Road, West End, NC 27376, USA*

We discuss the key elements involved in preparing a solid laboratory information management system (LIMS) request for proposal. This paper will look at the important aspects of selecting and implementing a LIMS along with promoting laboratory automation. An outline for creating a comprehensive RFP will be provided along with a sample LIMS RFP for users to modify to meet their specific laboratory data management and reporting requirements. The major categories will include overview and purpose, general specifications, LIMS requirements (system management, database management, sample management and tracking, sample scheduling, collection, identification), sample receiving, test/analysis administration, sample status monitoring, test result management and data validation; chain of custody, sample approval, QC, statistical analysis, ad hoc queries, information reporting, and interface requirements. Additional items that will be covered include, electronic instrument interfaces, transferring information to other enterprise systems, data migration from legacy systems, chemical inventory, personnel and equipment management, and portable data entry units (PDEs). The LIMS RFP should also focus on critical factors to LIMS success; product support and training. The sample RFP will also include a section on vendor qualifications, references, installation, technical support, upgrades, documentation, and validation. The sample RFP will conclude with functional and acceptance testing, the period during which the client reviews all the functional requirements to determine that they are all being met through final system acceptance. This paper will provide users with a step-by-step guide to create a comprehensive LIMS request for proposal.

*Keywords:* lab management, laboratory automation, LIMS
*Application code:* laboratory management
*Methodology code:* laboratory informatics

### SUCCESSFUL USE OF RAPID PROTOTYPING FOR GENERATING AND REFINING LIMS REQUIREMENTS

***Elena S. Peterson* and Gordon A. Anderson***

**Pacific Northwest National Laboratory, PO Box 999, Richland, WA 99352, USA*

A major step in any new experimental research project is deciding what data is required to capture and how to store it. While there are many commercial off-the-shelf LIMS systems available, they all rely on well-established procedures in a well-established laboratory environment. At the start of the Pacific Northwest National Laboratory's (PNNL) involvement in the Department of Energy's (DOE) Genomes to Life (GTL) program, the processes and protocols were the research. We were developing and testing various procedures and protocols that would lead us to be able to create a high-throughput process for analyzing proteins in complex. It was still critical to capture the data from each process to be able to evaluate its effectiveness especially when the processes were continually evolving. We also needed to manage a growing number of samples coming from other DOE laboratories as well as our own samples being stored in various freezers. One of the final stages of the process was to run samples through our well-established proteomics pipeline that already has its own data management facility. Since already existing LIMS systems were not going to be quickly or easily conformed to these needs, we built our own prototype LIMS (pLIMS) using a rapid prototype approach to build client/server applications as well as connect to existing software and capabilities. What we have found is that by using this approach, we are able to not only capture the experimental data as originally required but we are helping to generate and refine the new and more accurate requirements for capturing data and developing protocols.

*Keywords:* laboratory informatics, lims, sample and data management, scientific data management
*Application code:* laboratory management
*Methodology code:* laboratory informatics

### IMPLEMENTING A NEW SOFTWARE FRAMEWORK FOR THE LABORATORY

***Soheil Saadat* and Edward C. Long***

**Scientific Software, Inc, 6612 Owens Drive, Pleasanton, CA 94588, USA*

Laboratories are under increasing pressure to manage a complex mixture of instrumentation and also improve their information in order to provide effective information collaboration and sharing of knowledge. These pressures can be addressed by deployment of advanced software technologies to manage and control the instrumentation, and also manage the information flow without disrupting the way chemists and instruments work. A new software framework based on Microsoft .NET, which incorporates network appliances, instrument control, management of instrumentation information, and enterprise content management will be discussed. This laboratory software framework is designed to provide a highly flexible scale of implementation from single workstations to diverse client/server and thin-client technologies. The impact of this new framework on information sharing and collaboration in the laboratory will also be discussed.

*Keywords:* data mining, laboratory automation, laboratory informatics, scientific data management
*Application code:* laboratory management
*Methodology code:* laboratory informatics

### TECHNICAL CONSIDERATIONS IN REMOTE LIMS ACCESS VIA THE WORLD-WIDE WEB

***David Schlabach* and William Ulma***

**Accelerated Technology Laboratories, Inc, 496 Holly Grove School Road, West End, NC 27376, USA*

The increased dependency on the World-Wide Web by both laboratories and their customers has led LIMS developers to take advantage of thin-client web applications that provide both remote data entry and manipulation, along with remote reporting functionality. A web browser integrated with a LIMS provides both remote administration and real-time analytical result delivery.

There are several primary factors to consider.

(i) Information resource security and data integrity. Implementation of a web-enabled LIMS means opening the doors to your company's database and other resources you wish to provide to your customer. This presentation will outline how, with proper network security practices and hardware/software setup, users can securely accommodate this information with peace of mind. Data integrity is another determinant in moving forward with available technologies for information management applications.

(ii) User-access control. By allowing users from outside your company's facility access to internal information across the Internet, managers need reassurances that security measures are in place. With suitable methods for user-access controls, system administrators can be certain that users will only be able to view and manipulate information with permissions that have been granted or denied to them.

(iii) Ease of use. Companies may not mind investing the time and money to train employees to use the systems in-house but many may be deterred by the thought that their LIMS solution may be too complex for their clients to operate. Through an intuitive user-interface, a web-enabled application can provide users rapid data access.

*Keywords:* lab management, laboratory informatics, LIMS, software
*Application code:* laboratory management
*Methodology code:* laboratory informatics

### INTEGRATING ANALYTICAL LABORATORY OPERATIONS WITH BUSINESS PROCESS MANAGEMENT

***Dan Sudlik*[*] and Edward C. Long***

[*]*Scientific Software, Inc, 6612 Owens Drive, Pleasanton, CA 94855, USA*

Today's analytical laboratories must find new ways to work efficiently and productively. One area of key interest is integration of the laboratory information that comes about through analytical measurements and standard operating procedures with overall business and operational processes. Software applications known as business process management applications (BPM) have been used extensively outside the traditional laboratory to address needs in many business environments but these applications are only now becoming applied to the laboratory. Applying BPM technology in the laboratory can streamline the information sharing process, efficiently control the flow of analytical information, and help labs adopt a long-term strategy for archiving and sharing their information. A novel integration between the laboratory and business process management software will be explored along with a discussion of the specific benefits to any lab.

*Keywords:* laboratory automation, laboratory informatics, LIMS, scientific data management
*Application code:* laboratory management
*Methodology code:* laboratory informatics

### IMPLEMENTING A PAPERLESS LABORATORY BY EXTENDING THE FUNCTIONALITY OF A LIMS

***Robert D. Walla*[*] and Michael S. Zachowski***

[*]*Astrix Technology Group, 175 May Street, Suite 302, Edison, NJ 08837, USA*

Many laboratories have invested considerable resources implementing data processing systems such as chromatography data systems and laboratory information management systems (LIMS) with the goal of eliminating or reducing the amount of paper generated. Despite the efforts, even laboratories that have implemented state-of-the-art systems still generate large volumes of paper. Reducing the amount of paper generated will increase efficiencies and reduce costs.

This paper will present a case study of a project where the current LIMS was extended to eliminate manual paper data collection processes. The paper will describe technical aspects of the system including architecture and topology. The paper will present quantitative data on production, turnaround time, and costs before and after the implementation of the extended system.

*Keywords:* lab management, laboratory informatics, LIMS, scientific data management
*Application code:* laboratory management
*Methodology code:* laboratory informatics

### A SUCCESSFUL APPROACH TO A LIMS ASSESSMENT AND UPGRADE

***Michael S. Zachowski*[*] and Robert D. Walla***

[*]*Astrix Technology Group, 175 May Street, Suite 302, Edison, NJ 08837, USA*

Undertaking a laboratory information management system (LIMS) implementation or upgrade takes considerable resources, that are often underestimated by the client. In addition to the obvious license and support costs, most commercial off-the-shelf (COTS) products will require customization and/or configuration to meet a client's specific requirements and workflows. If these are not documented and compared to the base functionality of the COTS LIMS prior to the installation, it is likely that adequate resources will not be allocated and the project will fall short of its objectives. This paper will present a case study of a disciplined and systematic approach to a LIMS assessment and upgrade. User requirements (functional, technical, business) were collected, segmented, and prioritized. Use-case scenarios were created and incorporated with the requirements into a system specification. The specification was provided to the COTS LIMS vendor to address what functionality would be provided by the base package, configuration, customization or whether or not it was available. This approach provided the client with a better understanding of the full cost of the project and a system upgrade that met their requirements. Future tasks will include integration with instruments from other sources to create an enterprise solution.

*Keywords:* lab management, laboratory informatics, LIMS, scientific data management
*Application code:* laboratory management
*Methodology code:* laboratory informatics

### EXTENDING CHROMATOGRAPHY DATA SYSTEM BY AUTOMATION CAPABILITIES

***Toshinobu Yanagisawa,* Yasuhiro Funada, Yoshihiro Hayakawa, Takayuki Kihara, Okiyuki Kunihiro, Teruhisa Ueda, and Atsushi Yoshida***

**Shimadzu Corporation, 1, Nishinokyo-Kuwabaracho Nakagyo-Ku, Kyoto 604-8511, Japan*

Chromatography data system (CDS) is one of the most sophisticated softwares in analytical laboratory. In addition to automated data acquisition, calculation of results, and report generation, it must provide tools for compliance with GLP, GMP, and 21 CFR part 11. To accommodate all user requirements, CDS has grown huge having too many functions when oftentimes only basic operations are used. Due to this software evolution, laboratory managers have hard time replacing their CDS with new one in order to comply with current GLP/GMP regulations.

One of the solutions for this issue is to offer a simplified user-interface for beginners while providing advanced users with a complete version of the software. However, this approach is not useful for most laboratories since each one operates under different SOPs and 2 modes of operation (simplified and complete) do not nearly accommodate all the diversity of user requirements. Another solution is for the CDS to be easily customizable to meet individual user requirements. OLE automation is the key technology for this purpose. For example, a simplified user-interface can be easily created using an OLE automation feature and some programming in Visual Basic or another language. Our chromatography data systems support application specific software such as quality control, dissolution testing through automation capabilities. Simplified user-interface is supplied as a sample program. It can be customized to meet requirements of each individual SOP. The focus of this presentation is on customization of chromatography data systems by automation capabilities.

*Keywords:* chromatography, laboratory automation, software
*Application code:* laboratory management
*Methodology code:* data analysis and manipulation

### OPTIMIZATION OF NITROGEN DIOXIDE WITH A CHEMILUMINESCENCE AEROSOL DETECTOR

***Maria-Pamela P. Monterola,* Nicolo Omenetto, Benjamin W. Smith, Ronald J. Whiddon, and James D. Winefordner***

**Department of Chemistry, University of Florida, PO Box 117200, Gainesville, FL 32611, USA*

The highly sensitive detection of nitrogen dioxide is an essential requirement not only for air pollution monitoring purposes but for the ultrasensitive detection of explosive vapors as well. Photofragmentation of highly explosive materials such as trinitrotoluene (TNT), 1,3,5-trinitrohexahydro-1,3,5-triazine (RDX), and other highly energetic materials involves the release of $NO_2$ in its excited state which has a 30% probability of dissociating to NO and O radicals. This study developed an efficient chemiluminescent reaction of trace nitrogen oxides with an aerosol of the basic luminol solution. Aerosol is formed by continuous spraying of the reagent solution with a stream of the analyzed gas in a sealed reaction chamber. The chemiluminescent radiation is emitted from the gas/liquid interface boundary by interaction of $NO_2$ molecules in the gas phase with the luminol molecules present on the surface of aerosol droplets. A thermoelectrically cooled photomultiplier tube with high gain and negligible dark count rate served as an efficient detector for this system.Various parameters that affect the intensity of chemiluminescent reaction of $NO_2$ and luminol solution were optimized. These parameters include $NO_2$ to luminol ratio, pH of luminol solution, various solvents for luminol, effects on the signal enhancement of chemiluminescent reagents such as 1-iodophenol and $Na_2SO_3$, and the geometry of the reaction chamber. This study also investigated the influence of various interferent gases such as $CO_2$, NO, and $O_3$ chemiluminescent response of $NO_2$-luminol reaction. Furthermore, the elimination of these interferent gases by physical and chemical means were employed to achieve maximum signal-to-noise ratio for $NO_2$-luminol chemiluminescent reaction. Finally, the sensitivity and limit of detection of $NO_2$ was determined using the optimum conditions obtained.

*Keywords:* aerosols/particulates, chemiluminescence, instrumentation, luminescence
*Application code:* homeland security/forensics
*Methodology code:* fluorescence/luminescence

### DRUG SCREENING USING MICROEXTRACTION IN PACKED SYRINGE/LC-MS UTILIZING MONOLITHIC-BASED SORBENT MATERIAL

***Mohamed Abdel-Rehim,*** *** Zeki Altun, Lars Blomberg, and Jagerdeo Eshwar***

**DMPK&BAC, AstraZeneca R&D, Sodertalje 15185, Sweden*

Miniaturization of separation systems is a growing trend in analytical chemistry. This may provide rapid analysis, at low costs, under environmentally friendly conditions. Porous monolithic packing columns can be prepared by in situ polymerization of monomers in the presence of porogenic solvent within a tube. The packing is a continuous polymer network, composed of highly interconnected pores and aggregated globules. Microextraction in packed syringe (MEPS) is a new sample preparation technique (Current Patent Gazette, week 0310, WO03019149, vol. 77, 2003) that is very easy to use, fully automated, online, and rapid in comparison to previously used methods (M. Abdel-Rehim, "New trend in sample preparation: on-line microextraction in packed syringe for liquid and gas chromatography applications. I. Determination of local anaesthetics in human plasma samples using gas chromatography-mass spectrometry," *J. Chromatogr. B*, vol. 801, no. 2, pp. 317–321, 2004). In this work monolithic packing material was prepared in order to pack syringes (50–100 mL) for sampling, and capillary columns for micro-HPLC. The monolithic material was prepared by in situ radical polymerization of butylmethacrylate (BMA), ethylene glycol dimethacrylate (EDMA), and glycidylmethacrylate (GMA) in the presence of porogenic solvent composed by cyclohexanol and 1-dodecanol (M. Merhar, A. Podgornic, M. Barut, M. Zigon, A. Strancar) or 1,4-butanediol, propan-1-ol, and water.

*Keywords:* sample handling/automation
*Application code:* bioanalytical
*Methodology code:* sampling and sample preparation

### AIR MONITORING AT PARTS PER TRILLION LEVELS FOR RISK ASSESSMENT AND INDOOR AIR QUALITY STUDIES

***Vickie H. Paul,*** *** Thomas P. Wampler, and Charles P. Zawodny***

**CDS Analytical, Inc, 465 Limestone Road, Oxford, PA 19363, USA*

There is an emerging need in the community for improvements in parts per trillion (ppt) analysis capabilities, especially in two areas: risk assessment and quantifying the effects of vapor intrusion on indoor air quality. Building upon proven technology for ppt and sub-ppt analysis of airborne chemical agents in the workplace, this study will apply the same methodology to several volatile chlorinated compounds. Target compounds are vinyl chloride; 1,1-dichloroethylene (1,1-DCE), and trichloroethylene (TCE). The approach consists of high-capacity adsorbent sampling tubes and thermal desorption (TD), transferring 100% of the collected sample to a GC/MS rather than the fractional percentage frequently utilized in TD methods. The study will include a review of breakthrough volumes for various commercially available adsorbents, with testing of several individual and multibed layers to determine strengths and weaknesses and define optimum materials, sampling rates, and the effect of humidity. The study will also define minimum detection limits with GC/MS in full scan as well as selected ion modes.

*Keywords:* environmental air, sample introduction, thermal desorption, ultratrace analysis
*Application code:* environmental
*Methodology code:* sampling and sample preparation

### AUTOMATED LINER-EXCHANGE FOR GC-INJECTORS: NEW CONCEPTS FOR HANDLING DIRTY SAMPLES

***Eike Kleine-Benne,*** *** Dirk Bremer, Volkmar Heinke, Andreas Hoffmann, Karsten Kuhr, and Bernd Rose***

**Gerstel GmbH & Co KG, Aktienstrasse 232-234, Muelheim An Der Ruhr 45473, Germany*

Sample cleanup steps, which are needed in order to prepare, for example, environmental or food samples for pesticide analysis, are time-consuming and a potential source of errors. Simplification—or elimination—of such procedures is often the motivation behind the development of new analytical methods and new instrumentation. Unfortunately, analytical instruments do not normally tolerate introduction of "dirty" samples or even "dirty" extracts. For example, extracts containing suspended matter or high-molecular-weight compounds contaminate a GC inlet after a few injections, causing peak broadening or even a loss of sensitive compounds. Reducing or eliminating cleanup steps will result in dirty extracts and daily—or even hourly—maintenance of the GC system will be required.

A simple and automated liner-exchange system is able to overcome most chromatographic problems caused by dirty samples in GC analysis. A solution is presented that uses a commercially available PTV-injector in combination with an autosampler, which can automatically perform a liner-exchange at any time during a sample sequence. Every liner is equipped with a transport adapter, which also allows liquid injection through a septum. Adapters fitted with liners are transported by means of the autosampler which also performs the liquid injection.

*Keywords:* gas chromatography/mass spectrometry, instrumentation, pesticides, sample handling/automation
*Application code:* general interest
*Methodology code:* sampling and sample preparation

### IMPROVING AUTOMATED LIQUID HANDLER PERFORMANCE THROUGH RELIABLE VOLUME DELIVERY MEASUREMENTS

***John T. Bradshaw***

*Artel, Inc, 25 Bradley Drive, Westbrook, ME 04092, USA*

Automated liquid handling (ALH) systems are highly effective at increasing throughput and decreasing labor expenditure in a number of applications such as drug discovery and development, proteomics, genomics, and molecular diagnostics. Significant technological advancements over the past decade have led to broad implementation of ALH equipment. However, despite the technological sophistication of modern ALH systems, they are still fallible devices requiring regular performance verification. Additionally, optimum performance of volume delivery is attainable for ALH devices only when a reliable measure of the delivered volume can be made, from which appropriate protocol adjustments can be carried out to tune the device performance. The dual-dye photometric approach implemented by the Artel multichannel verification system (MVSTM) provides a tool for measuring both the accuracy and precision of volume delivery from various types of multichannel liquid handling equipment (both automated instrumentation as well as manual pipettes). The traceable measurements provided by the MVS system can be used to reliably analyze ALH performance, as well as adjust delivery protocols to optimize the system. This presentation will focus on case studies wherein ALH performance was measured using the MVS, and optimized for ideal volume delivery. Additionally, recent developmental work for extending the MVS testable volume range down to 0.1 $\mu$L, along with system validation in this enhanced volume range will be discussed.

*Keywords:* drug discovery, laboratory automation, process monitoring, quality
*Application code:* validation
*Methodology code:* UV/VIS

### REAL-TIME MEASUREMENTS OF AMBIENT ULTRAFINE PARTICLE COMPOSITION: HOW ARE THEY PERFORMED AND WHAT DO THEY TELL US ABOUT THE AIR WE BREATHE?

***Murray V. Johnston***

*Department of Chemistry and Biochemistry, University of Delaware, Newark, DE 19716, USA*

Many studies have linked airborne particles to adverse health and environmental effects. The chemical composition of an individual particle will vary according to its source and subsequent transformations in the atmosphere. Chemical composition measurements provide a means to identify particle sources, to assess their impact on human health and the environment, and to implement effective control strategies to reduce air pollution. Recent work in our group has involved two aerosol mass spectrometers: RSMS-3, a real-time single-particle mass spectrometer, and PIAMS, a photoionization aerosol mass spectrometer for characterizing organic components in particles. The RSMS-3 has been deployed at particulate matter measurement sites in five US cities. Ambient number concentrations were determined for major particle composition classes and correlated with particle size, wind direction, and time of day/year. Based on this information, local and regional sources of particles in each class were postulated. For example, almost 40% of the particles in the Baltimore aerosol are internally mixed, consisting primarily of organic carbon, ammonium nitrate, and ammonium sulfate. Most of these particles are likely to be derived from regional sources. The remaining particles appear to be derived mainly from local sources and processes, and include elemental carbon (almost 30%), ammonium nitrate (over 10%), and various metals (over 20%). Particle events were also studied. Each event was characterized by a rapid increase in particle mass and/or number that was also associated with a rapid change in one or more chemical components such as nitrate, sulfate, and/or organic carbon.

The PIAMS has been used to characterize major sources of organic particle emission. Diesel exhaust from an idling bus showed a number of peaks that can be attributed to unburned fuel droplets consisting of high-molecular weight hydrocarbons. Gasoline exhaust from an automobile showed peaks corresponding to a variety of semivolatile alkyl aromatics. Meat cooking aerosol showed peaks corresponding to cholesterol, palmitic acid, and stearic acid among others. Wood smoke showed enhanced signals corresponding to levoglucosan, 4-ethylsyringol, and 4-propylsyringol. Cigarette smoke showed an enhanced signal corresponding to nicotine. All of these results are consistent with previous GC-MS experiments and suggest that PIAMS can be used to monitor rapid changes in ambient organic particle emission.

*Keywords:* aerosols/particulates, environmentalair, laser desorption, mass spectrometry
*Application code:* environmental
*Methodology code:* mass spectrometry

### IN PROCESS METHODS FOR MONITORING RESIDUAL MOISTURE LEVELS DURING FREEZE-DRYING PROCESS

***Nicole Denkinger***

*Boehringer Ingelheim Pharma GmbH & Co. KG, A Bp Process Science, G55-00-06, Biberach 88397, Germany*

A defined residual moisture level is a major stability criterion for freeze-dried products, especially for peptides and proteins. Therefore development should result in a freeze-drying process which leads to an optimal level of residual moisture. The common monitoring measurements during freeze-drying, like pressure and temperature, or even the offline monitoring (of, e.g., residual moisture content, pressure inside vial) after sampling provide important facts. However, several advanced methods can be used to determine the

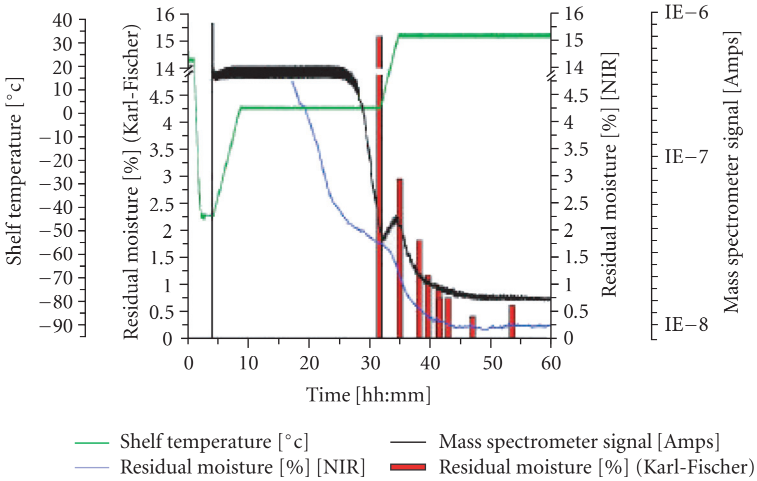

FIGURE 12

status of lyophilized formulations online like near-infrared and mass spectrometry. They are providing significantly more information about actual process conditions concerning the moisture content during the drying process. The demand to improve analytical methods and techniques is crucial and correlates with the introduction of process analytical technology (PAT). The freeze-dryer is linked to a mass spectrometer and a near-infrared system. With this setup, it is possible to record the mass spectrometer signal of the water vapour content in the drying chamber and the residual moisture level of the sample vial within one run without interfering with the drying process. With these data, it is possible to determine the end of secondary drying precisely according to a defined residual moisture level. A direct correlation between the mass spectrometer signal, the near infrared spectroscopy signal, and the residual moisture levels determined by the Karl-Fischer method was found. All data can be collected within one freeze-drying process. After calibration of the mass spectrometer for a specific freeze-dryer and the corresponding product, an aseptic drying process can be controlled until a predetermined moisture level is achieved without disturbing the drying process.

*Keywords:* mass spectrometry, near infrared, water
*Application code:* pharmaceutical
*Methodology code:* other

### THE LAB AS A BUSINESS: MANAGING AND MEETING FINANCIAL, CULTURAL, AND CUSTOMER NEEDS

***Gordon Logan* and Simon N.Wood***

**Labformatics Ltd, 271 Ashley Road Hale, Altrincham, Cheshire WA15 9NF, UK*

Today the laboratory is seen as, and therefore must be run as, a business. However, this can raise issues for lab managers and lab staff who may not be accustomed to this. There is a balance between running the lab as a business and the scientific and technical culture of the lab that has to be maintained if the organisation is to gain the maximum benefit from the lab. One problem that has to be overcome is that the greatest asset of any lab is likely to be the knowledge and experience of the laboratory staff, and from a financial aspect the value of this can be difficult to measure. This means that traditional, and often inappropriate, financial measures may be used to measure lab performance and improvement. However, techniques such as activity-based costing (ABC) and time-is-money (TISMO) analysis can be used to measure costs associated with individual lab activities and therefore accurately identify areas of financially poor performance. Workshops and critical process maps can be used to identify the needs of customers and areas of inefficiency in the laboratory process. In addition, the implementation of balanced scorecards can form the basis of continuous improvement projects that are properly aligned with the strategy of the lab and the organisation, which maximise the intangible assets of the lab. This presentation will discuss the issues outlined above in more detail and will show how the techniques described can be used to address these issues and bring together the seemingly differing demands of financial, cultural, and customer needs. The presentation will draw on the case study of a large water utility lab facing these challenges.

*Keywords:* lab management
*Application code:* laboratory management
*Methodology code:* other

### USING LIMS TO INTEGRATE AND TRACK PERFORMANCE METRICS AT THE BENCH

***François Rodigari***

*EBMUD, PO Box 24055 MS 59, Oakland, CA 94623, USA*

Accurate and timely measurements of laboratory performance goals can only be performed by integrating the metrics into the laboratory information management system (LIMS). The laboratory management and analytical staff need to work closely with the information system staff to integrate the performance metrics into LIMS. Well integrated metrics should provide daily feedback to staff on what is required to be performed in order to meet the laboratory performance goals. Additionally, staff should be provided with real time feedback on actual performance.

*Keywords:* LIMS
*Application code:* laboratory management
*Methodology code:* laboratory informatics

### DESIGN FEATURES OF CONTINUOUS-FLOW VAPOUR GENERATOR FOR USE WITH AA SPECTROMETRY

***Steve F. Morton*** [*] ***and Adrian H. Holley***

[*] *Thermo Electron Corporation, Solaar House PO Box 207, Cambridge CB5 8BZ, UK*

Vapour generation coupled with atomic absorption spectrometry is a long-established technique that offers low-cost parts-per-billion sensitivity for the important arsenic group of elements. The development of devices based on continuous flow principles has permitted fully automated operation, providing fast, convenient, and sensitive analyses for these important elements. The requirements for a high-performance continuous-flow vapour generator will be reviewed. Carrier gas flow rate control and the separation of the gas and liquid phases will be shown to be significant,and design solutions for these critical components will be proposed. Performance characteristics of a vapour generator designed on these principles will be presented, and the application of the device to some typical analyses will be discussed.

*Keywords:* atomic absorption, hydride, mercury
*Application code:* general interest
*Methodology code:* atomic spectroscopy/elemental analysis

### DEVELOPMENT OF A GC-AFS FOR ROUTINE US EPA METHOD 1630 MEASUREMENTS

***Warren T. Corns***

*P S Analytical Ltd., Arthur House, Crayfield's Industrial Estate Main Road, Orpington, Kent BR5 3HP, UK*

For several years P S Analytical has been promoting a GC-AFS system for mercury speciation. The systems supplied comprise modified commercial gas chromatography such as the Agilent GC 6890 coupled with a P S analytical atomic fluorescence through a pyroliser module which converts the separated mercury species to mercury (0) prior to quantifiable using the GC chromatography software. This paper will describe how the GC-AFS was modified to perform US EPA method 1630. Known volumes of extracted or distilled samples are transferred to an impinger bottle. After the addition of acetate buffer to control pH, the sodium tetraethylborate reagent (NaBEt4) was added to ethylate the mercury compounds. Methylmercury and inorganic mercury are converted to methylethylmercury and diethylmercury, respectively. These species were then purged from the impinger bottle and subsequently trapped on a carbotrap. The trapped species are released from the carbotraps by specially designed thermal desorption assembly and transferred onto a GC column. Both packed and capillary columns were studied. Comparative data using both types of columns will be presented. After separation the organo-mercury species are thermally degraded to Hg0 using a quartz cracking tube maintained at 80°C prior to atomic fluorescence detection. Analytical performance characteristics of an optimized arrangement will be presented along with the results for natural water samples from the UK.

*Keywords:* gas chromatography, mercury, speciation
*Application code:* environmental
*Methodology code:* atomic spectroscopy/elemental analysis

### IMPROVING DATA QUALITY AND MEASUREMENT EFFICIENCY IN BIOMEDICAL ELEMENTAL ANALYSIS USING ICP-MS

***Simon M. Nelms,*** [*] ***Martin Nash, Phil N. Shaw, and Bill Spence***

[*] *Thermo Electron Corporation, Ion Path Road Three, Winsford, Cheshire CW7 3BX, UK*

Elemental analysis in biomedical samples is currently achieved using mainly flame and graphite furnace atomic absorption spectroscopy. Flame AAS, although effective for measuring Na, K, and Zn in serum and urine, is not sufficiently sensitive for determining Se in serum and Pb and Cd in whole blood. For this reason GFAAS, with its higher sensitivity, has become accepted as the benchmark technique for trace elemental biomedical analysis. However, GFAAS has its limitations. It is relatively slow, is prone to contamination and can suffer from relatively poor precision, compared to ICP-MS, in some assays. Despite the multielement, low detection limit, excellent precision, and high sample throughput capabilities of ICP-MS, this technique has not yet been widely adopted by the biomedical community, arguably because of the perceptions that it is prone to interferences, complex to use, and expensive. Interference problems and complexity have been significantly improved in recent years through the development of collision cell interference removal technology and simplification of the software and hardware. From an expense perspective, the cost per sample of ICP-MS compared to GFAAS is highly dependent on the sample workload and the number of elements required to be measured. At high sample throughput (more than 30 samples per hour) and for multielement assays, ICP-MS soon becomes more cost-effective than GFAAS. ICP-MS has the additional benefit over GFAAS that it can be easily interfaced with liquid or gas chromatographic separation systems to facilitate sensitive, rapid, and accurate elemental speciation measurements, thereby increasing its value to the biomedical community. This paper will discuss advances in routine elemental analysis in biomedical samples using ICP-MS and will briefly discuss its potential for emerging biomedical assays, such as speciation.

*Keywords:* atomic spectroscopy, biomedical, ICP-MS
*Application code:* biomedical
*Methodology code:* atomic spectroscopy/elemental analysis

### COURSE DEVELOPMENT TOOLS FOR USE WITH AN OPEN SOURCE DISTANCE LEARNING PLATFORM

***Joseph G. Solch*[*] and Roger K. Gilpin***

[*]*Wright State University, Brehm Research Laboratories College of Science and Mathematics, Dayton, OH 45435, USA*

Previously we have reported on the development of a series of advanced spreadsheet-based chemistry simulations that are easily integrated with traditional course content material to create a more dynamic learning experience. This approach was found to be practical because it takes advantage of the ability of commercial web-based distance learning systems such as WebCT to efficiently handle the complex business of managing student registration, assignments, tests, reports, and grading. Unfortunately, a serious disadvantage of commercial web-based distance learning systems is the high software cost that typically limits use to major institutions. During this past year our focus has been on converting our existing self-learning courses to a free web-based distance learning system that is available as open source software (OSS). This OSS, known as Moodle (http://www.moodle.org), is free to download, use, modify, and even distribute (under the terms of the GNU General Public License). It runs on Linux, UNIX, Mac OS X, Netware, and Windows operating systems and is currently available in 50 languages. Our implementation on the free Gentoo Linux distribution (http://www.gentoo.org/) produces a powerful web-based distance learning system with little or no software costs. The goals of this work were to couple in-house developed simulations with a free web-based distance learning system and to provide remote access to laboratory instrumentation. The challenges, solutions, and results will be presented, including the development of tools that make it more easy to use the OSS for simulations and other lecture and laboratory course content materials.

*Keywords:* computers, education
*Application code:* other
*Methodology code:* computers, modeling and simulation

### RAPID OPTIMIZATION OF GRADIENT IC SEPARATIONS THROUGH PREDICTIVE MODELING

***James A. Schibler*[*] and Madden E. John***

[*]*Dionex Corporation, 500 Mercury Drive PO Box 3603, Sunnyvale, CA 94085, USA*

Ion chromatography (IC) is usually performed using “standard” rather than optimized conditions. Although improved resolution and/or faster analyses are often achievable, few people try to optimize their IC separations, for good reasons. Traditional optimization is a trial-and-error process of varying method conditions and hoping for improvements. It requires dozens of injections that consume samples, eluents, instrument time, and operator time. Also, there is no assurance that worthwhile improvements will be achieved. HPLC prediction software turns out to be impractical for IC. The software is unaffordable for many laboratories, and large amounts of retention data must be collected in order to produce accurate predictions for IC, so each optimization project still carries a high cost. Now, using a new software tool, isocratic and gradient IC separations can be optimized quickly, easily, and reliably. The tool uses retention algorithms and known retention data to accurately predict retention of specified analytes under various conditions for selected ion-exchange columns. Interactive resolution maps and virtual chromatograms help analysts quickly learn IC separation behavior. Convenient commands quickly identify the optimum separation conditions for a particular application, based on user-specified criteria (such as column flow rate, eluent system, and minimum acceptable resolution). With the new tool, the optimum IC column and separation conditions for resolving specified analytes can be determined in just a few minutes, without doing any laboratory work. The tool also makes gradient IC as easy to work with as isocratic IC, enabling users of all skill levels to gain the advantages of gradient ion chromatography. This presentation will discuss this new tool and its application to the optimization of isocratic and gradient IC separations.

*Keywords:* ion chromatography, lab management, method development, software
*Application code:* other
*Methodology code:* computers, modeling and simulation

### DEVELOPMENT AND SELECTION OF GENERIC CHROMATOGRAPHIC METHODS

***Margaret Antler,*[*] Alexey Danilov, and Michael McBrien***

[*]*Advanced Chemistry Development, Inc, 90 Adelaide St.W. Suite 600, Toronto, Ontario M5H 3V9, Canada*

Generic chromatographic methods are generally a small set of separation methods that are designed to produce sufficient resolution for the majority of samples in a situation where it is not practical to spend time developing high-quality methods for specific samples. High throughput and walk-up laboratories thus rely on generic, or standard separation methods for structure verification and purity estimation. Software tools can further increase sample throughput by evaluating which method in the set of generic methods will be most appropriate for a particular group of compounds. In addition, data quality can be increased by ensuring that compounds are retained sufficiently on the column and/or can be expected to show resolution from expected/unexpected impurities. The software works in the following manner. For a particular set of methods, the software is first trained.

A number of representative samples are analyzed using the set of generic methods, and the results are entered along with their chemical structures into the software. Once the initial training is complete, the chemical structure(s) of the novel compound(s) are entered into the database. A structure-based retention model is developed for each generic method using the most similar compounds in the database. Selection between each of the candidate methods is done based on the predicted results. This paper will describe the design of typical generic methods, the column selectivity required, and the selection of compounds for the training set. Results will then be shown for some "unknowns" highlighting how the correct choice of above criteria for the training set leads to excellent prediction capabilities from the software.

*Keywords:* high-throughput chemical analysis, HPLC, pharmaceutical, software
*Application code:* high-throughput chemical analysis
*Methodology code:* computers, modeling, and simulation

### THE UTILIZATION ON-LINE OF COMMON PARAMETER MONITORING: A NEW SYSTEM FOR RECOGNIZING AND IDENTIFYING DISTRIBUTION SYSTEM INCURSIONS

***Dan J. Kroll*** **and Karl King***

*Hach Homeland Security Technologies, 5600 Lindbergh Drive, Loveland, CO 80539, USA*

The drinking water distribution system is one of the nation's key infrastructure assets. The ease of attacking the system, combined with the fact that little or no quality monitoring occurs after water has left the treatment plant, makes the danger of such an attack acute. Prior to this, there has not been a system capable of detecting such an event and alerting the system's managers so that effects of an attack or accident can be contained. A system designed to address the problem of distribution system monitoring is described here. The developed system employs an array of common analytical instrumentation, such as pH and chlorine monitors, coupled with advanced interpretive algorithms to provide detection identification-response networks that are capable of enhancing system security. Through the use of laboratory testing, pilot-scale testing on pipe loops, and real world beta site deployment, the system has been shown to be effective in detecting a wide diversity of possible threats including TICs, TIMs, biological and warfare agents. In addition, the system has been shown to recognize common accidental intrusions such as antifreeze and sewage. The response of these various agents is not only adequate to detect the presence of a contaminant, but the unique profile of the responses allows for some degree of identification. Through the use of a searchable library, the system is capable of providing not only an alarm but also an identification of the cause. The profiles of over 80 threat agents and many common contaminants have been compiled. A proprietary baseline estimator dramatically reduces false warnings from regular fluctuations in operational parameters. The deployment of a system such as the one described will be an invaluable tool in maintaining the integrity of the nations drinking water supply.

*Keywords:* data analysis, identification, on-line, water
*Application code:* environmental
*Methodology code:* data analysis and manipulation

### FLOW INFINITE DILUTION ANALYSIS: A NEW METHOD TO IMPROVE ANALYSIS ACCURACY IN FIA

***Stuart J. Chalk***

*University of North Florida, Department of Chemistry and Physics 4567 St. Johns Bluff Road S., Jacksonville, FL 32224, USA*

Flow infinite dilution analysis (FIDA) is a new approach to combatting inaccuracies in analytical flow injection analysis measurements. An FIA system with high accuracy pumps and dilution capability is used to generate a reproducible concentration gradient of the analyte (and reaction product) under investigation. This profile is then used to generate an infinite dilution curve based on a dilution curve from a reference solution. Extrapolation back to infinite dilution gives an accurate estimate of the concentation of the analyte even with high matrix interferences. Example analysis of environmental waters for total iron is used to show the capability of the method.

*Keywords:* environmental/water, flow injection analysis
*Application code:* environmental
*Methodology code:* data analysis and manipulation

### ELECTROCHEMICAL AMPLIFICATION SCHEMES USING SELF-ASSEMBLED MONOLAYERS: DETERMINATION, VARIATION, AND OPTIMIZATION OF AMPLIFICATION AND SELECTIVITY MECHANISMS

***Adam J. Bergren*** **and Marc D. Porter***

*Iowa State University, Institute for Combinatorial Discovery Department of Chemistry, Ames, IA 50011, USA*

Electrochemical signal amplification provides a means to lower detection limits. This presentation describes the mechanistic details of a system based on sacrificial homogeneous regeneration using the selectivity of self-assembled monomolecular films of alkanethiolates on gold electrodes. Details of the selectivity and amplification mechanisms with respect to the properties of electroactive molecules and electrode fabrication are examined, enabling a general description that broadens the fundamental understanding of the interfacial processes central to performance. Electrochemical methods are used to demonstrate, evaluate, and quantify the amplification and selectivity mechanisms. We describe the maximum amplification magnitude using a simple mathematical model and provide experimental verification. Cyclic voltammetric simulations are used to determine the conditions under which the model is valid.

Experimental amplification factors of 250 are attainable using cyclic voltammetry, lowering the detection limit more than an order of magnitude. Analysis of molecular properties determined from electrochemical and chromatographic experiments, literature, and theoretical calculations reveal that the most important factors giving rise to selectivity are electron-transfer kinetics and hydrophobicity of the analyte and monolayer films. Interesting new insights into the structure of self-assembled monolayers are also provided by systematic variation of film properties. Our results indicate that ferrocene derivatives partition to a small extent into disordered alkanethiolate monolayers. The partitioning event (and therefore selectivity and amplification) is very sensitive to the details of the electrode preparation, and therefore the packing density and ordering of the monolayer films. These findings are used to design systems that realize the theoretical maximum amplification.

**ACKNOWLEDGMENT**

Support from Eastman Chemical Company is gratefully acknowledged.

*Keywords:* chemically modified electrodes, electrochemistry, optimization, sensors
*Application code:* general interest
*Methodology code:* electrochemistry

### STUDIES OF THE SPECIATION AND REACTIVITY OF ENVIRONMENTAL ARSENICALS

***Joseph H. Aldstadt,[*] Jason G. Harb, and Aaron R. Roerdink***

*[*]University of Wisconsin-Milwaukee, Department of Chemistry 3210 N. Cramer Street, Milwaukee, WI 53211, USA*

We will describe our recent work in three areas: (1) a summary of our development of optimized methods for the determination of major "feed-additive" arsenicals (3-nitro-4-hydroxyphenylarsonic acid and para-arsanilic acid) at low $\mu$g L-1 levels by solidphase microextraction (SPME) gas chromatography (GC) and long-path absorbance spectrophotometry (LPAS), respectively; (2) development of a field-flow fractionation (FFF) method for determination of inorganic arsenic on colloidal material (50–500 nm); and (3) studies of the thermal decomposition of gas-phase organoarsines by MS using a quartz kinetics chamber. Following derivatization by 1,3-propanedithiol and SPME using a 65 $\mu$m polydimethlysiloxane-divinylbenzene fiber, 3-nitro-4-hydroxyphenylarsonic acid ("Roxarsone") was quantified by using parallel quadrupole ion-trap and pulsed flame photometric detection (LOD = 2.7 ppb). For para-arsanilic acid, a flow injection-absorbance spectrometric method was developed in which p-ASA was derivatized with dimethylaminobenzaldehyde and the product measured using a long-path (1 m) absorbance cell (LOD = 21 ppb). Both methods were applied to authentic environmental samples and high recovery (> 90%) was observed. For physical speciation of inorganic arsenic, a Flow FFF method was optimized and applied to contaminated groundwater. Finally, to better understand the reactivity of organoarsines, we will describe an experimental apparatus for gas-phase kinetic studies based on in situ generation of organoarsines using purge and trap with continuous on-line MS detection of thermal decomposition products.

*Keywords:* environmental/water, flow injection analysis, gas chromatography, mass spectrometry
*Application code:* environmental
*Methodology code:* mass spectrometry

### SAMPLE PREPARATION AND ANALYSIS OF TOXIC METALS IN CONSUMER AND INDUSTRIAL PRODUCTS

***Ranjan Roy***

*SCP Science, 21800 Clark Graham, Baie D'urfe H9X 4B6, Canada*

There is growing pressure on industry to reduce certain key metals in plastic housings and electronic/electrical components for both consumer and industrial products. In the European Union, the waste from electrical and electronic equipment (WEEE) and the reduction of hazardous substances (RoHS) directives have targeted CrVI, Cd, Hg, and Pb. This legislation specifically requires that these metals be monitored due to the quantities of electronics that end up as scrap in landfill sites. A target date of July 2006 has been set whereby these metals must be below accepted levels when producing new electronic equipment. Plastics such as polyvinyl chlorides (PVC) and Polyethylene (HDPE) are also being monitored for the above metals. Using conventional hotplate techniques, these polymers are often difficult to decompose without the accompanying loss of analytes due to the high-temperatures involved. This paper compares the use of a high temperature graphite block digestion technique for sample preparation with a conventional hotplate digestion using European Reference Materials EC680 and EC681 certified plastic reference materials.

*Keywords:* environmental, ICP, ICP-MS, sample preparation
*Application code:* environmental
*Methodology code:* sampling and sample preparation

### DETERMINATION OF TRACE MERCURY IN ENVIRONMENTAL SAMPLES BY HIGH-RESOLUTION INDUCTIVELY COUPLED PLASMA—MASS SPECTROMETRY

***Jerzy Mierzwa***

*Tulane University, Coordinated Instrumentation Facility Tulane University, New Orleans, LA 70118, USA*

The determination of mercury traces by inductively coupled plasma a mass spectrometry (ICP-MS) is still a very complex task both from the sample preparation perspective and

ICP-MS measurement itself. Atomic absorption and atomic fluorescence spectroscopies combined with mercury cold vapor generation are frequently used for the determination of mercury in environmental samples. For the last couple of years, high-resolution magnetic sector ICP-MS (HR-ICP-MS) has become a really powerful analytical tool, not just because of solving some important interference problems, but also because of an excellent detection power of these instruments. A double-focusing sector field HR-ICP-MS instrument "Element-2" (Thermo-Finnigan MAT, Bremen, Germany) was used in this study. Some environmental certified reference materials were employed in this research. The effect of sample introduction system including a new "APEX" (Elemental Scientific Inc., Omaha, NE) desolvating system and microflow nebulizers was studied. Additionally, the influence of organic solvents (methanol, ethanol) on mercury signal was studied. The obtained analytical figures of merit will be discussed and some advantages of the use of high resolution ICP-MS instrument will also be presented.

*Keywords:* environmental analysis, ICP-MS, mercury, metals
*Application code:* environmental
*Methodology code:* atomic spectroscopy/elemental analysis

### IN-SITU SPECTROSCOPIC CLEANING VALIDATION

***Robert A. Lodder* and Aaron Urbas***

**University of Kentucky, Department of Chemistry A123 Astecc Building, Lexington, KY 40506-0286, USA*

In recent years, the appeal of process analytical technologies (PAT) in the manufacturing of pharmaceuticals has grown markedly. Cleaning validation is one area of pharmaceutical processing that could benefit significantly from developments in PAT. This research represents an extension of previous work involving the monitoring of laser light scatter for quantifying surface protein contamination on glass. Previous studies were conducted with a single component system, where potential interferences were absent. A prototype instrument for laser light scattering measurements through glass surfaces was constructed. The current work will focus on extending this instrumentation to analyze polished stainless steel surfaces as well as multicomponent systems. Light scatter alone lacks the selectivity for quantification of the analyte of interest in multicomponent systems. Other spectroscopic phenomena, specifically polarization, will be examined to augment light scattering information to improve prediction accuracy for surface concentrations of the target analyte. Generation of calibration models for the proposed system will also be discussed.

*Keywords:* pharmaceutical, process analytical chemistry
*Application code:* pharmaceutical
*Methodology code:* physical measurements

### REAL-TIME THERMAL DEVOLATILIZATION OF MERCURY AND MERCURY COMPOUNDS FROM CCBS DETECTED WITH ATOMIC ABSORPTION SPECTROMETRY

***David J. Hassett,* Loreal V. Heebink, and Erick J. Zacher***

**UND Energy & Environmental Research Center, 15 North 23rd Street PO Box 9018, Grand Forks, ND 58203, USA*

Thermal release of air toxic trace elements, particularly of mercury, is important from the perspective of long-term use, storage, or disposal of coal combustion by-products (CCBs), especially in some manufacturing scenarios for CCB utilization. Thermal devolatilization of mercury and mercury compounds was investigated in a laboratory-scale apparatus. A small CCB sample was placed in a tube furnace and heated at a linear ramp from ambient to 750°C at a rate of 25°C per minute. Mercury release was measured in real time using an atomic absorption spectrophotometer. A Hewlett Packard 3395 integrator was used for data collection. A large variety of CCBs have been analyzed for the thermal release of mercury. Most of the thermal curves generated were straightforward, containing only one or two major desorption peaks. The curves were rather difficult to interpret since there is no way, at present, using this apparatus, to determine exactly what is happening during the thermal treatment. There are several possible scenarios.

(1) Mercury and mercury compounds, as sorbed, are being released unchanged.
(2) Mercury compounds are being desorbed by a mechanism of thermal decomposition whereby sorbed compounds such as HgO are thermally decomposed to mercury and oxygen.
(3) Mercury or mercury compounds are chemically reacting with the CCB components then thermally desorbing according to the first or second scenario as described above.

### ACKNOWLEDGEMENT

This abstract was prepared with the support of the US environmental protection agency (EPA) through the center for air Toxic Metals (CATM) and the US Department of Energy (DOE) in part through the Coal Ash Resources Research Consortium (CARRCSM).

*Keywords:* atomic absorption, mercury, method development, thermal desorption
*Application code:* environmental
*Methodology code:* thermal analysis

### PROBLEM-BASED LEARNING AND FORENSIC CASE STUDY IN THE INSTRUMENTAL ANALYSIS LABORATORY

***Douglas E. Raynie***

*South Dakota State University, Department of Chemistry and Biochemistry Shepard Hall 121 Box 2202, Brookings, SD 57007, USA*

For the past several years, the necessity of including problem-based learning and case studies in the analytical curriculum has been emphasized. This was especially noted in the 1997 report "Curricular developments in the analytical sciences," based on two NSF-sponsored workshops. The case studies emphasize the role of analytical chemists as problem solvers and serve to challenge students to develop critical-thinking skills. The laboratory exercises also serve to reinforce the communication skills necessary to professional success. This presentation will outline the course development. For example, initial laboratory exercises focus on outlining an approach to problem solving as promoted by the Procter and Gamble short course in problem solving for undergraduate students, "Professional Analytical Chemists in Industry." After an approach to problem solving is developed, laboratory exercises develop from relatively staightforward, like the determination of the cause of a gasoline odor in a flower shop (where the students must convey their findings in the form of a letter or business memo understandable to the shop owner), to more complex, like determining an art forgery or investigating drug degradation to support an FDA new drug application. This laboratory experience culminated in a forensic project. Working in conjunction with a criminal justice class, the instrumental students are presented with "crime scene" evidence. The scenario is designed to allow each student to thoroughly investigate one piece of evidence and to provide both a suspicion of guilt and reasonable doubt. Thus, the students work as individuals in a group setting—in either the prosecution's forensic laboratory or as defense consultants—much like they will encounter in their professional careers. The criminal justice students serve as prosecuting and defense attorneys, which keeps the chemistry students on task. At a mock trial (the jury is also supplied by the criminal justice class), the instrumental students are expected to clearly and concisely explain how their evidence was analyzed, how the results were obtained, and how these results support their conclusions, and defend their statements. During this presentation, our experience with the problem-based learning case-study approach to teaching instrumental analysis will be shared, including resources for developing appropriate exercises.

*Keywords:* education, forensic, forensic chemistry, teaching/education
*Application code:* homeland security/forensics
*Methodology code:* education/teaching

### THE DEVELOPMENT OF AUTOMATED EQUIPMENT FOR DRYING AND CONCENTRATING ENVIRONMENTAL EXTRACTS

***Robert S. Johnson***

*Horizon Technology, Inc., 8 Commerce Drive, Atkinson, NH 03811, USA*

Two steps that have a major impact on the recoveries for both liquid-liquid (LLE) and solid-phase (SPE) extraction techniques are drying and concentrating the extract prior to GC analysis. Residual water must be removed to prevent the extract from separating into multiple phases and back extraction of water soluble analytes. The extract must also be concentrated to improve detection limits by selectively evaporating the extraction solvent. Drying extracts has historically been accomplished manually with sodium sulfate. Recently, hydrophobic membranes have become available that can provide automated removal of residual water. Further, this step can be incorporated into equipment that selectively evaporates the extraction solvent to completely automate sample drying and concentration for GC analysis. The use of such equipment for environmental applications will be discussed. Emphasis will be placed on analyte recovery, carryover, and sample throughput.

*Keywords:* automation, environmental analysis, method development, sample preparation
*Application code:* environmental
*Methodology code:* sampling and sample preparation

### MULTICHANNEL OVATION BIONATURAL PIPETTE: IMPROVING ERGONOMICS FOR PIPETTE USERS REQUIRING MULTICHANNEL FUNCTIONALITY

***Jeff Calhoun***

*VistaLab Technologies, 27 Radio Circle Drive, Mt. Kisco, NY 10549, USA*

Ovation bionatural pipettes are a new class of pipettes designed to address major ergonomic risk factors. Awkward posture, repetition, contact stresses, and high operating forces can predispose pipette users to work-related musculoskeletal disorders. A single-channel ovation pipette was introduced in 2002, offering considerable reductions in stress and force levels. Additional research and development has been conducted to extend the benefits of improved ergonomics to pipette users requiring multichannel functionality. Our research and ergonomic studies showed a significant compounding of risk factors when pipetting in a multichannel environment.

(1) Visual alignment of a multi-tip head requires awkward body positioning.
(2) The forces required for acquiring multiple tips is greatly increased over single-tip operation.

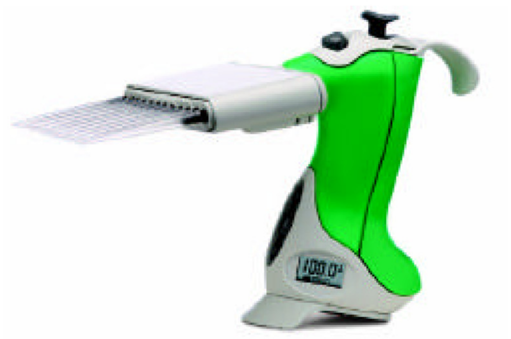

Figure 13

(3) The effort required to discard multiple tips simultaneously can exceed recommended voluntary contraction limits.
(4) Correct forearm and wrist orientation is even more important when using multichannel devices.
(5) The highly repetitious nature of multichannel pipetting increases the unfavorable effects of even minor posture deviations.

Using the principles of ergonomic science, adaptations to meet these challenges have been incorporated into new 8-channel and 12-channel models of the ovation bionatural pipette. Extensive testing and user reviews have revealed that the new multichannel ovation bionatural pipettes provide significant reductions in the ergonomic stress levels when compared to traditional, axial-designed pipettes. Specifications, operation, and features of the new models and data from the testing of ergonomic factors will be described in the presentation.

*Keywords:* sample handling/automation, sample introduction, sample preparation, titration
*Application code:* industrial hygiene
*Methodology code:* sampling and sample preparation

### RAPID SCREENING AND CONFIRMATIONAL ANALYSIS OF RESIDUAL PESTICIDES IN AGRICULTURAL SAMPLES BY GC ECD/FPD AND GC-MS/MS

***Jessie Butler***

*Thermo Electron Corporation, 2215 Grand Avenue Parkway, Austin, TX 78728-3812, USA*

The analysis of organophosphorous pesticides has routinely been done on the flame photometric detector (FPD) and chlorinated pesticides on the electron capture detector (ECD). Since the ECD is a nondestructive detector, it may be configured in tandem with the FPD for simultaneous analysis of both chlorinated and organophosphorus pesticides in a single injection. A smart screening program has been written to query the results of the GC detector runs and create a new sequence to be run for confirmation on the GC mass spectrometer. The TriPlus autosampler was programmed to access two inlets on the same GC. Extracts were screened by injection on the tandem ECD/FPD and only those extracts flagged as positive for detection of a target pesticide were then run by GC-MS/MS. The external source PolarisQ ion trap quadrupole mass spectrometer was used to show confirmational analysis by MS/MS. MS/MS is required for detection of $<$ 10 picogram levels in the heavy vegetable matrix. Vegetable extracts were spiked to determine the accuracy of the method. A linearity study was performed for the ECD, FPD, and GC-MS/MS. The precision of the method was tested by tabulation of the internal standard response.

*Keywords:* agricultural, food science, GC-MS, pesticides
*Application code:* agriculture
*Methodology code:* gas chromatography/mass spectrometry

### FAST, DIRECT AND RELIABLE ANALYSIS OF MINOR COMPONENT IN OLIVE OIL BY ON-LINE REVERSED PHASE LIQUID CHROMATOGRAPHY: GAS CHROMATOGRAPHY USING A PATENTED AUTOMATED THROUGH OVEN TRANSFER ADSORPTION DESORPTION (TOTAD) INTERFACE

***Ariadna Galve-Bosch,* José Manuel Cortes, Roger Gibert, Raquel Sánchez, Ana Vázquez, and Jesús Villén***

**KONIK-TECH, Av. Cerdanyola, 73, Sant Cugat Del Vallès, Barcelona 08190, Spain*

The TOTAD interface coupling an HPLC to an HRGC is based on a modified programmed temperature vaporizer (PTV) injector of a KONIK 4000 HRGC packed with a suitable trapping material, but the way it operates is very different. The TOTAD interface includes a six-port valve and three on-off valves, operated under the control of the K4000 microprocessor. All the system is automated, and consequently, in order to obtain the gas chromatogram of any trapped HPLC fraction, it is necessary only to inject the crude sample of interest into the HPLC. The manual TOTAD interface has been previously described (J. Microcol. Sep. 1999, 11, 582–589). In the present application a method for direct on-line determination of minor components of olive oil is proposed. 20 $\mu$L of virgin olive oil previously filtered, were injected into a 50 $\times$ 4.6 mm i.d. HPLC column packed with 10 $\mu$m silica (C4, Vydac 214 TPB). The composition of the mobile phase (methanol-water 95 : 5 (v/v)) was maintained constant until elution of the minor components. These are well separated from the triglyceride fraction. The methods allow the analysis of all minor components included in this fraction together or the analysis of two different fractions separately (sterols and tocopherols fraction, and erytrodiol, uvaol and scualene fraction). The fraction containing the components of interest is transferred and trapped into the HRGC by using the automated TOTAD® interface. The liner of the interface is packed for this application with 1 cm length of Tenax TA. The speed of sample transfer can be varied from 0.1 to 2 mL/min. The interface has been maintained to 80°C during transfer and heated to 325°C afterwards to produce the thermal desorption of the trapped analytes and their "injection" into the capillary GC column (30 m × 0.32 mm id × 0.25 $\mu$m film of 5% phenyl-methyl silicone). No variation in the retention time is observed. Relative standard deviation (RSD), $n = 5$, from the absolute peak areas varied from 3 to 11%.

*Keywords:* automation, food science, other hyphenated techniques, sample introduction
*Application code:* agriculture
*Methodology code:* sampling and sample preparation

### THE DETERMINATION OF MERCURY IN SEAFOOD: A COMPARISON OF COLD VAPOR ATOMIC ABSORPTION AND FLUORESCENCE TECHNIQUES

***David Pfeil* and Bruce MacAllister***

**Teledyne Leeman Labs, 6 Wentworth Drive, Hudson, NH 03051, USA*

Mercury is a well-known pollutant in the environment. Its affects on humans, and mammals in general, are also well understood and documented. One of the primary sources of mercury for humans is through the consumption of fish. Mercury present in fish is predominately in the form of methyl mercury, a form that is readily absorbed by man. Several varieties of marine fish such as tuna, swordfish, and shark often are contaminated with mercury in excess of 1 ppm, the maximum level allowed by the US FDA. Currently, any consumption of these fish by high risk group, that is, pregnant woman, is currently discouraged. High mercury concentrations found in fresh water fish have resulted in 2000 water bodies in the USA with posted advisories restricting the consumption of indigent fish. Because of bioaccumulation, the concentration of mercury in certain types of fish can exceed 100 000-fold concentration of mercury in the local water. Unfortunately, the larger and older fish that are the preferred catch in sports fishing often have the highest mercury concentration. This presentation will look at the determination of mercury in a variety of fish. Data will be presented using both cold vapor fluorescence and absorption techniques. Commonly employed digestion procedures for each technique vary slightly and will be discussed as well.

*Keywords:* atomic absorption, elemental analysis, food science, mercury
*Application code:* safety
*Methodology code:* atomic spectroscopy/elemental analysis

### AMINO ACID PROFILE AS FINGERPRINT FOR NATURAL PRODUCT IDENTITY AND QUALITY

***Alesya Bradley,* Tivadar Farkas, and Jafar Toulouee***

**Phenomenex, Inc, 411 Madrid Avenue, Torrance, CA 90501, USA*

Amino acid profiles are often used for the identification and confirmation of the origin of various natural products. Specific amino acid levels or their ratios may be indicators of product quality or confirm the purity of a specific varietal origin. Common fraud of natural products involves mixing of common varieties into high-quality products or dilution or rare varieties. There is a general interest in the knowledge of the chemical characteristics of certified natural products like fruit juice, wine, honey, or tea in order to defend them against being modified or falsified. Most of the current methods used for the analysis of amino acids in natural products are laborious, time consuming, and expensive. The method based on a novel amino acid analysis procedure described in this work is fast, reliable, and cost-effective. The total cycle time for the analysis of amino acids in natural products with this procedure is 15 minutes, including all sample preparation. The procedure involves a simple solid phase extraction (SPE) step, followed by a rapid derivatization reaction conducted in aqueous phase at room temperature. We present data on amino acid profiles for different varieties of fruit juice, honey, and tea. The analysis of ten different varieties of honey demonstrate the detection of 24 amino acids in these samples, with excellent sensitivity, reproducibility, and recovery. The data also underlines the specificity of amino acid profiles for natural products of specific origin.

*Keywords:* amino acids, food science, GC, natural products
*Application code:* food science
*Methodology code:* gas chromatography

### APPLICATION OF 0.15 MM FUSED SILICA COLUMNS WITH INDIVIDUAL CONTRIBUTORY INJECTION TECHNIQUES FOR RAPID GC PROFILING OF DISTILLED SPIRITS WITHOUT SAMPLE PREPARATION

***Jaap De Zeeuw* and Kevin MacNamara***

**Varian, Inc, Herculesweg 8, Middelburg 4330 EA, Netherlands*

Analysis of the secondary compounds (congeners) in distilled spirits is important for reasons of quality and production control, development of new flavours, and brand authentication in the market place. The matrix composition of distilled spirits is relatively clean and so injection without sample preparation is possible. Methodologies have been developed in the past using the CP-Wax 57 CB type bonded phase which provides excellent selectivity, stability, and chemical inertness for components of interest measured in water/ethanol matrices. Split injection profiles more abundant congeners eluting just before and after the ethanol peak. Split injection however is not suitable for detection and quantification of later eluting congeners at low mg/L (ppm) levels. A more productive approach is to remove the matrix ethanol and water by split solvent venting in a PTV. Conditions can be tuned so that the only compounds vented with the matrix are those which can be determined by the separate split injection. A 5 to 10 ul injection is sufficient to profile the compounds of interest. Therefore the combination of individual split and solvent venting with splitless modes

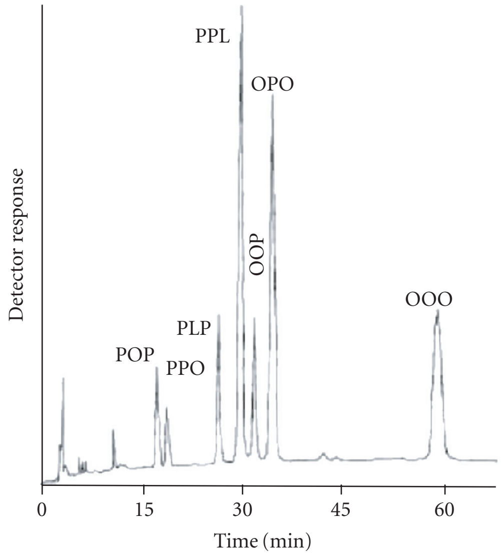

Figure 14

offers a scheme for comprehensive profiling of distilled spirits without sample preparation. The analysis run times using these sample introduction modes can be substantially reduced by using 0.15 mm id capillary columns. The use of 0.15 mm diameter capillary columns results in tremendous gains in analysis speed for both injection modes allowing high throughput of samples. These 0.15 mm id columns fit in every GC and GC-MS and allow typically direct a factor 2 reduction in runtime using with minimal change of conditions.

*Keywords:* beverage, capillary GC, GC columns, trace analysis
*Application code:* food science
*Methodology code:* gas chromatography

### TRIGLYCERIDE ANALYSIS BY AG-HPLC FOR RAPID SCREENING OF FORMULATED MARGARINES AND SPREADS

***Richard O. Adlof** and Gary List***

**USDA, NCAUR, 1815 N. University Street, Peoria, IL 61604, USA*

The amounts and types of triglycerides (TAGs) in the oil phase of margarines and spreads are considered responsible for such properties as spreadability, resistance to water/oil loss, and melting at body temperature (mouth feel). Over the past 25 years, a number of economic, health, and consumer-driven factors have stimulated research aimed at reducing the levels of trans fatty acids formed during the hydrogenation of vegetable oils. Alternatives to hydrogenation include interesterification, blending of tropical and liquid vegetable oils, fractionation and, more recently, development of structurally modified oils by transgenic or conventional plant breeding methods. Within the last decade, silver ion HPLC (Ag-HPLC) has been utilized in such diverse applications as determination of trans fatty acids in vegetable oils, conjugated fatty acids (FAs) in dairy products and, more recently, for screening of triglyceride compositions in seed oils and commercial lipid formulations. We found Ag-HPLC to be a powerful technology to characterize ("screen") complex mixtures of TAGs (Figure 14) and for isolating specific components for further analysis by DSC (relating TAG structure to TAG mp's and crystalline form(s)) or NMR (solid fat content) and, with proper control of solvent composition, for semi-preparative (5–10 mg per run) separations. Ag-HPLC can also be utilized to analyze TAGs differing only in the location of the FA(s) in the molecule (Figure 14) and results are comparable to and more rapid than those achieved by lipolysis/gas chromatography. Specific examples where Ag-HPLC was utilized to screen a variety of commercially available margarines, spreads, and seed oils are included.

*Keywords:* food science, HPLC, method development, modified silica
*Application code:* food science
*Methodology code:* liquid chromatography

Table 4

| Extraction technique | Hop pellets | |
|---|---|---|
| | Alpha acids | Beta acids |
| Pressurized solvent extraction | 6.87 | 3.88 |
| Solid liquid extraction | 7.35 | 4.27 |

### OPTIMIZATION OF THE PRESSURIZED SOLVENT EXTRACTION AND ANALYSIS

***Al Kaziunas,* Martin Fetner, Pavel Karasek, Elena Ostra, Michal Roth, and Rolf Schlake***

**Applied Separations, 930 Hamilton Street, Allentown, PA 18101, USA*

The determination of alpha and beta acids in hops is an important analytical procedure in the brewing industry used to control the organoleptic qualities of beer. Hops extractions are traditionally performed using the soxhlet or solid liquid extraction (SLE) technique. A rapid alternative extraction technique is the pressurized solvent extraction of hops using organic solvents at elevated temperatures and pressures. The pressurized solvent extraction of alpha and beta acids from hops was optimized by evaluating sample preparation, solvent pH, extraction mode, temperature, solvent composition, and solvent-to-sample ratio. Extracts were analyzed by HPLC-UV and compared to a standard solid liquid extraction technique (EBC 7.7) using a diethyl ether-methanolacetic acid solvent mixture. Critical factors in the optimization of the extraction of hop pellets and cones using the pressurized solvent extraction technique are discussed.

*Keywords:* accelerated solvent extraction, beverage, flavor/essential oil, food science
*Application code:* food science
*Methodology code:* liquid chromatography

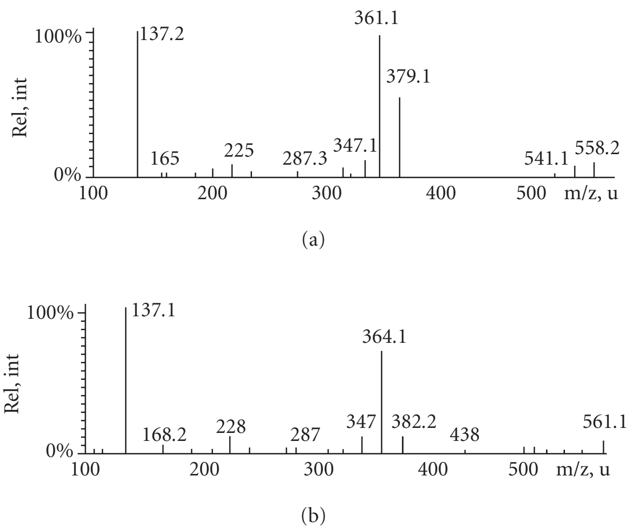

Figure 15

### RAPID ASSAY OF OLEUROPEIN IN VIRGIN OLIVE OIL BY APCI MS/MS AND ISOTOPE DILUTION METHOD

***Leonardo Di Donna,* Fabio Mazzotti, Enzo Perri, and Giovanni Sindona***

**Università della Calabria/Dipartimento di Chimica, Via P. Bucci, Cubo 12/C, Arcavacata Di Rende, CS 87036, Italy*

Virgin Olive oil contains micro-components such as tocopherols and flavonoids and other phenolic compounds such as tyrosol, hydroxytyrosol and oleuropein of recognized antioxidant activity (S. McDonald, P. D. Prenzler, M. Antolovich, K. Robards, Food Chem., 73, 73–84, 2001). Oleuropein (OLP) is a secoiridoid glucoside present in virgin olive oil and drupes; its structural characterization has been accomplished by electrospray ionization (ESI) tandem mass spectrometry (MS/MS). MS/MS has been recently used to assay micro-components in foodstuff by means of suitable internal standards (L. Di Donna, G. Grassi, F. Mazzotti, E. Perri, G. Sindona, J. Mass Spectrom., 2004). The isotope dilution method enhances the reliability of the procedure (D. De Luca, L. Di Donna, L. Maiuolo, F. Mazzotti, G. Sindona, Anal. Chem., 76, 5104–5108, 2004). Oleuropein has been quantified by LCMS/MS through the use of external and internal standard. However, none of the previous works deals with the use of isotope dilution methods. The present works intend to quantify the micro-component OLP in virgin olive oil by means of atmospheric pressure chemical ionization tandem mass spectrometry (APCI-MS/MS) under MRM condition, employing the synthetically labeled OLP as internal standard. The MS spectrum of OLP dissolved in ammonium solution show mainly the adduct ion at m/z 558 [M+NH4]+. The MS/MS spectrum of the ion at m/z 558 provides a fragmentation pattern easily recognizable. The difference between the MS/MS spectra of OLP and OLP-d3 are relative only to few fragments (Figure 15) that retain the carboxy-metoxyl group and these differ in these units; the transitions m/z 558 m/z 137 for the analyte and m/z 561 m/z 137 for the labeled internal standard, respectively, have been used for the quantitative assay.

### ACKNOWLEDGMENT

Funds from FONAB project are acknowledged.

*Keywords:* high throughput chemical analysis, liquid chromatography/mass spectroscopy, tandem mass spec
*Application code:* food science
*Methodology code:* liquid chromatography/mass spectrometry

### IDENTIFYING NONTARGETED PESTICIDE RESIDUES AND THEIR DEGRADATION PRODUCTS IN FOOD USING LC/TOF/MS ACCURATE MASS COMBINED WITH LC/ION TRAP/MS/MS

***Juan F. Garcia-Reyes,* Amadeo R. Fernández-Alba, Imma Ferrer, Antonio Molina-Díaz, and Michael Thurman***

**University of Jaen, Analytical Chemistry Division, Jaen 23071, Spain*

The combination of LC/TOF/MS with accurate mass measurement to generate elemental compositions of ions and LC/Ion Trap/MSn providing complementary structural information represents a powerful analytical approach for the identification of trace levels of organic compounds in complex matrices. In this work, we explore the capabilities of the combined use of these techniques in order to detect and characterize nontargeted pesticide residues and their degradation products as a preliminary screening step, previous to the development of comprehensive quantitative multiresidue methods used in monitoring programs for food safety purposes. The usefulness of the combined use of these two techniques relies on the ability of LC/TOF/MS to provide the accurate mass of the molecular ion to assign a tentative elemental composition, which is then searched against databases (namely, "The Merck Index, The ChemIndex, electronic commercial catalog"), to find out the identity of the suspected species. Once a chemical structure has been assigned, the same process is applied to any of the characteristic fragmentsions (used, if necessary, in source fragmentation) to confirm the proposed identity with the elemental composition of the fragment ion. The final step is the confirmation by the assignment of the different characteristic fragments ions generated in Ion Trap/MSn experiments. We have investigated different extracts of vegetable samples collected in different markets from Andalusia (Spain), and from the study we have unequivocally identified and quantified (when standards were available) various pesticides and their characteristic degradation products. Some examples are shown where different pesticides were identified without the use of standards. A widely used post-harvest fungicide (imazalil) was identified along with its major degradation product, and also other fungicides such as prochloraz (and its metabolite) and procymidone.

*Keywords:* food science, liquid chromatography/mass spectroscopy, pesticides, time of flight MS
*Application code:* food science
*Methodology code:* liquid chromatography/mass spectrometry

### ULTRAFAST GC APPLIED TO HEAD SPACE ANALYSIS OF VOCS IN PACKAGING MATERIALS

***Riccardo Facchetti,* Flavio Bedini, Giuseppe Catino, Paolo Magni, and Thomas Porzano***

*Thermo Electron, Strada Rivoltana Km. 4, Rodano, Milano 20090, Italy*

Determination of volatile organic compounds in packaging material is a typical screening type analysis performed to prevent effects of contamination of enclosed goods from VOC migration (i.e., food contamination). This analysis is typically performed through headspace sampling. Besides tests performed by health regulatory agencies for quality approval on finished goods, a very rapid determination is important for a real time monitoring of the packaging-material production process. Such strict control allows in fact to keep the process under tight control. This work describes how headspace analysis of VOCs in food packaging materials can be coupled to ultrafast gas chromatographic separation. The ultrafast GC instrumental solution features a direct resistive heating of the capillary column capable of very fast temperature programming (up to 20°C/s). The dramatic reduction in analysis time is achieved by combining the very fast temperature programming with the use of short narrow bore columns and by optimizing the sample incubation conditions used by the headspace autosampler. Proper selection of the column stationary phase type and thickness of inner diameter and length allowed to obtain the separation power required for a correct quantitation of the components of interest. Determination of various VOCs, as residual solvents in inks and in printed paper, was achieved in only 1–2 minutes resulting in about a 20–30-fold speed increase with respect of conventional GC methods.

*Keywords:* capillary GC, headspace, quality control, volatile organic compounds
*Application code:* food science
*Methodology code:* gas chromatography

### DISCRIMINATION, AUTHENTICITY AND MATURATION OF TEQUILA BY ELECTRONIC NOSE AND TONGUE

***Eric Chanie***

*Alpha M.O.S., 20 Avenue Didier Daurat, Toulouse, NJ 31400, France*

A shortage of agave cactus, which is used to produce tequila, has had a significant impact on the availability and prices of tequila. In Mexico, among the 136 species of agave, the blue agave and agave tequilana weber azul are the only allowed in tequila production. As a direct result of this shortage, the Alcohol Labeling & Formulation Division (ALFD) has recently noticed changes in the labels and formulation of tequilas and tequila speciality. Control of origin, class, and type of tequila became the major concerns. In this experiment, samples have been studied with two complementary techniques developed by Alpha MOS: Kronos (fingerprint mass spectrometry) used as an electronic nose for the odor analysis and the astree electronic tongue for the taste assessment. Three different qualities (two Blue Agave Tequila with and without maturing and Tequila "Reposado" (one month age)) of tequila and seven unknown samples have been studied by the two techniques. Results indicate that the electronic nose is able to differentiate Tequila product grades and further distinguish competitor product. Astree electronic tongue has succeeded in ranking tequila in terms of taste by maturation levels. The combination of the two instruments results is helpful to benchmark the different tequila brands of the market.

*Keywords:* analysis, process monitoring, quality control, sensors
*Application code:* food science
*Methodology code:* other

### MATURITY MONITORING OF FRUIT USING AN ELECTRONIC NOSE WITH SENSOR ARRAY AND FINGERPRINT MASS SPECTROMETRY

***Jean-Christophe Mifsud,* Shuji Watanabe, and Koichi Yoshida***

*Alpha M.O.S., 20 Avenue Didier Daurat, Toulouse 31400, France*

The characteristic aroma of fruit greatly contributes to their overall acceptance by consumer. Traditionally, food flavor is analyzed through sensory profiling by a trained panel, by gas chromatography (GC), or by GC-mass spectrometry (MS). Although this had led to identification of several new odoriferous compounds, the process is time consuming and less reliable, due to nonuniformity of assessment conditions. The electronic nose, a more recent technology, is designed to specifically measure an entire aroma or odor in a way similar to humans. It can distinguish differences between samples and predict acceptability or consumer response, based on a fingerprint technique using a sensor array or mass spectrometry. This presentation will detail how the analysis of headspace with an electronic nose of a fruit at different stages of maturation enables to define the right maturity step for good consumer acceptance. A specific study has been made for an exotic fruit called snake fruit (pondoh), which is a famous fruit for Indonesian people and unpleasant fruit, when it is too ripe, for nonnative people. The Prometheus system (combining both sensor array technology and fingerprint mass spectrometry) was able to select the best masses and best sensors for the application. We will show how the electronic nose proved to be faster than GC, and more reliable than sensory panel, and how it is a nondestructive method appropriate for fruit maturity monitoring. Discussion will be carried out about the scientific results of such new instrumental fingerprinting techniques for the food market and economic consequences for such perishable products.

*Keywords:* food science, monitoring, quality, volatile organic compounds
*Application code:* food science
*Methodology code:* sensors

### CONTROL OF QUALITY OF MILK-BASED PRODUCTS WITH A GAS SENSOR ARRAY (ELECTRONIC NOSE)

***Wolf Muenchmeyer,* Andreas Walte, and Juergen Warrelmann***

**Airsense Analytics, Hagenower Str. 73, Schwerin 19061, Germany*

In the production of milk-based products a control of the quality clearing the process is needed. This is possible with sensor array systems, also known as electronic noses. This electronic noses usually consist of an array of simple sensors, in this case metal oxide semiconductors (MOS), combined with the required electronics and chemometrical software. The technology can be used for screening applications. Time-consuming separations like in gas chromatography are often not needed. The system is capable to deliver fast results, informing the user if the sample is ok or not. The systems are often used when questions such as “Is the sample contaminated with chemicals?”, “Is the sample contaminated with bacteria or yeast?”, “Is the mixture of ingredients as it should be?”, or “Is the product rancid or even rotten?” have to be answered. The University of Bremen has tested over a period of 6 months different yoghurts. The problem was to detect contaminated samples with wrong yeast. With a simple headspace technique, the system was capable of detecting the contaminated samples. After the testing period the sensor array was installed in a factory. Results of the performance of the sensor array will be shown.

*Keywords:* array detectors, food science, monitoring, quality control
*Application code:* food science
*Methodology code:* sensors

### DETERMINATION OF FLAVOR IN FILTERED ORANGE JUICE USING SOLID PHASE DYNAMIC EXTRACTION, GAS CHROMATOGRAPHY AND ON-LINE SAMPLE FILTRATION

***Ingo Christ* and Ulrike Kuehn***

**CHROMSYS LLC, PO Box 15131, Alexandria, VA 22309, USA*

Orange juice is a valuable daily source of Vitamin C. According to the US Department of Agriculture, the flavor of orange juice alone represents 40% of the Department’s requirements for meeting the standard of Grade A quality. Flavor can be influenced by such factors as concentration, freezing, pasteurization, and absorption by packaging materials. Given the importance of flavor to attaining Grade A quality, this paper will discuss how filtering both pulpy and pulp-free orange juice affects its overall flavor. For GC analysis, it is necessary to have particulate-free samples. Even pulp-free orange juice contains between 8% and 12% particulates. Past studies have typically employed centrifugation for the pulp separation process; however, this is a manual and time-consuming procedure. Using an automated sample filtration system, we were able to eliminate the centrifugation process and thus save time and obtain better reproducibility. We then used a gas chromatograph, autosampler, and solid phase dynamic extraction to determine whether the samples had lost any flavor compounds during the filtration process. We examined the major compounds in both filtered and unfiltered samples, specifically looking at D-limonene, $\alpha$-pinene, sabinene, $\beta$-myrcene, ethylbutyrate, octanoal, and decanal. The filtered samples only experienced a small reduction in some of these flavor compounds; thus, we conclude that filtering orange juice has very little effect on flavor.

*Keywords:* flavor/essential oil, laboratory automation, sample handling/automation
*Application code:* food science
*Methodology code:* sampling and sample preparation

### RAPID GC-ION TRAP-MS-MS FOR SIMULTANEOUS DETERMINATION OF POLYCYCLIC AROMATIC HYDROCARBONS (PAHS) AND ORGANOCHLORINATED PESTICIDES IN SEWAGE SLUDGE USING PRESSURIZED LIQUID EXTRACTION

***Murad I. Helaleh* and Ali A. Al-Omair***

**Kuwait Institute for Scientific Research, Central Analytical Laboratory PO Box 24885, Safat 13109, Kuwait*

It is interesting to determine some of the organic pollutants present in sewage sludges since they are associated with agricultural use of the sludges. The main target of our study is to develop a fast and reliable analytical method for determining both polycyclic aromatic hydrocarbons (PAHs) and organochlorinated pesticides (OCPs) in sewage sludges, using pressurized liquid extraction process. Optimization of the extracted conditions was performed in order to obtain an acceptable level of recoveries. PLE is an efficient method in terms of extraction recoveries as well as repeatability. The sewage sludge samples were collected from four waste water treatment plants (WWTPs) in Kuwait and were subjected to a clean-up process using silica gel and aluminum oxide and then subjected to bio-beads columns in order to remove all lipids. Sulfur was removed by copper filling and finally the samples were injected to GC using ion trap MS-MS in the electron impact ionization.

*Keywords:* accelerated solvent extraction, gas chromatography, mass spectrometry, quantitative
*Application code:* environmental
*Methodology code:* gas chromatography/mass spectrometry

### A FAST GCMS SYSTEM FOR MOBILE LABORATORY ANALYSIS OF TOXIC COMPOUNDS AND CHEMICAL WARFARE AGENTS

***Daniel B. Cardin,* Chris J. Casteel, and Thomas X. Robinson***

*Entech Instruments, Inc, 2207 Agate Ct., Simi Valley, CA 93065, USA*

Rapid identification and quantification of toxic compounds in air, water, and soil is extremely vital during an accidental or terrorism-related chemical release. GCMS remains the most definitive technique for analyzing these compounds due the sensitivity and specificity of this 2-dimensional technique. However, proper sample handling and rapid injections needed to perform fast GCMS have required the development of specialized inlet systems and inert sample collection devices. These sampling devices need to be simple and rugged enough to be used by nonanalytical personnel, while being designed to allow proper decontamination prior to leaving an affected area. A fast GCMS system is presented that quickly quantifies a wide range of toxic and acetyl cholinesterase inhibiting compounds. Gas phase samples are collected using inert Silonite vacuum canisters, while liquids and solids are analyzed by large volume headspace using 500 cc vials. The dynamic range of GCMS is enhanced by altering sample injection volumes from as low as 0.1 mL for PPM-level analysis to as much as 1000 mL for low part-per-trillion detection. For many chemicals including ammonia and hydrogen sulfide, whole air sampling and loop injection provides better recovery during GCMS analysis than any other sampling technique by avoiding even a single adsorption/desorption step. Adsorbent amenable compounds are preconcentrated on two separate traps that alternate between trapping and desorption/bakeout for faster sample throughput. The design of the sampling media and software-controlled inlet makes this approach successful in measuring most "GC-compatible" chemicals that could be considered a threat to human health.

*Keywords:* environmental analysis, forensic, gas chromatography/mass spectrometry, headspace
*Application code:* homeland security/forensics
*Methodology code:* gas chromatography/mass spectrometry

### SIGNATURE CHEMICALS USED BY INSTRUMENTAL AND BIOLOGICAL DETECTORS TO LOCATE ITEMS CONTAINING DRUGS AND DRUG ODORS INCLUDING CURRENCY

***Kenneth G. Furton,* Ya-Li Hsu, Alisha Latham, Stefan Rose, and Bradley Young***

*Florida International University, University Park Int'l Forensic Research Institute, Miami, FL 33199, USA*

This work involves solvent extraction and headspace solid-phase microextraction (SPME) combined with GC/MS to quantify drug residues and to confirm the signature odor chemicals used by detector dogs to locate drugs and drug odors focusing on the drugs cocaine and MDMA. Studies include the analysis and identification of the headspace chemicals above a variety of samples, followed by completion of double-blind dog trials of the individual components in an attempt to isolate and understand the target compounds that dogs alert to. CW/DVB and PDMS SPME fibers proved to be the optimal fiber types for the drugs focused on in this study. Field studies with detector dogs have demonstrated possible candidates for new pseudo scents as well as the potential use of controlled permeation devices as nonhazardous training aids providing consistent permeation of target odors. Many compounds of interest were found to be present in the headspace composition of the MDMA tablets tested, including piperonal, MD-P2P, and methamphetamine with field studies demonstrating that canines are alerting to approximately 10–100 mg of piperonal that is found exclusively in MDMA tablets. The dominant cocaine odor chemical has been confirmed to be methyl benzoate via spiked samples as well as controlled delivery devices with threshold levels of 1–10 g spiked methyl benzoate or 0.1–1 ng/s odor permeation. Extraction studies demonstrate that the currency fiber and ink play important roles in trapping cocaine particles. The recovery of cocaine from both currency and paper decreases exponentially. At 5 weeks, the recovery of cocaine is 10% to 15%. No cocaine was recovered by solvent extraction after 5 weeks. Other researchers have suggested that the ink provides a good bind site for cocaine. In our study, however, the ink appears to be a weaker binding site than the fibers themselves and hindered the cocaine from reaching the currency fibers.

*Keywords:* bioanalytical, forensic, gas chromatography
*Application code:* bioanalytical
*Methodology code:* gas chromatography/mass spectrometry

### IDENTIFICATION OF SIGNATURE MICROBIAL VOLATILE ORGANIC COMPOUNDS FROM TOXIC MOLDS USING SPME/GC/MS AND CANINE DETECTION

***Robert T. Griffith,* Kenneth G. Furton, and Krish Jayachandran***

*Florida International University, CP 345, 11200 SS 8 Street, Miami, FL 33199, USA*

Mold growth is dependent on humidity, temperature, and a supply of nonliving organic material which serves as a nutrient source. When adequate conditions exist, mold is able to flourish, often undetected. The volatile secondary metabolites, MVOCs, are emitted from flourishing molds, and may be species-specific. It may be possible to detect fungal growth down to the species level based on the composition of the microbial volatile organic compounds emitted from a culture. This study is researching what target compounds are being emitted from toxic species of molds including Aspergillus versicolor, Penecillium chrysogenum, and Stachybotrys chartarum and the possible signature MVOCs used by mold detector dogs to accurately locate these species. Samples of *Aspergillus versicolor, Penecillium chrysogenum,* and *Stachybotrys chartarum* were grown in vitro and purified in the laboratory. *Stachybotrys chartarum* was grown and purified on corn meal agar; *Apergillus versicolor* and *Penecillium chrysogenum* were grown and purified on potato dextrose agar. All samples were cultured in triplicate. Headspace analysis was conducted using solid phase microextraction/gas chromatography/mass spectrometry to determine the specific odor signatures of the volatile metabolites for each species. Sample extraction conditions were optimized by varying the fiber types, the time of sample exposure, and the amount of sample being analyzed. This study aims to address the effect of varying concentrations of molds and length of time molds are allowed to grow on the odor signatures obtained via SPME/GC/MS analysis of the pure mold cultures. By determining the compounds and their relative amounts that comprise the odor signatures for each of the mold species, it can be better understood how toxic molds can be detected by instrumental and biological detectors.

*Keywords:* GC-MS, SPME, volatile organic compounds
*Application code:* homeland security/forensics
*Methodology code:* gas chromatography/mass spectrometry

### EXTENDING THE SCOPE OF SCREENING FOR TOXIC ANIONS IN FOODS AND BEVERAGES USING SPME SAMPLING OF ETHYLATION PRODUCTS WITH ANALYSIS BY GAS CHROMATOGRAPHY-MASS SPECTROMETRY

***Kevin J. Mulligan***

*US Food and Drug Administration, 6751 Steger Drive, Cincinnati, OH 45237-3097, USA*

In current times, there is a threat of chemical terrorism to our food. This requires the development of rugged, efficient, rapid methods to screen for the presence of toxic agents so that we can effectively respond to situations in which product tampering is suspected. A group of chemicals that are of concern includes small molecules which would be present as anions if introduced into typical food matrices, for example, sodium azide. As reported at PittCon04, we have developed a procedure which utilizes a robust aqueous ethylation reaction in conjunction with headspace solid phase microextraction (SPME) and GC-MS to indicate the presence of azide, cyanide, or fluoroacetate at the trace level. This method uses the same instrumentation and capillary column that is employed in screening protocols for drugs and pesticides and, thereby, limits the number of gyrations that an analyst needs to endure to extend the scope of their analytical screening methodologies. A 0.1 mL portion of the liquid sample (or a basic aqueous extract) is mixed with 100 mg of a 1 : 1 (mol : mol) mixture of dibasic and tribasic potassium phosphate in a 2 mL GC autosampler vial. Ethylation is accomplished by adding 0.05 mL of a 3:1 (mol:mol) mixture of ethyl p-toluenesulfonate and 18-crown-6. The vial is sealed with a septum cap and incubated for about 30 min at mildly elevated temperature (70°C) to complete the reaction. To sample the headspace, the septum is pierced with a needle and the vial is laid on its side so that contents form a thin layer. A polydimethylsiloxane-coated SPME fiber is introduced through the piercing and exposed to the vapor within for 60 seconds. At the end of this interval, the SPME fiber is transferred to the injection port of a GC-MS system for analysis. The separation is conducted on a conventional capillary column (Rt×5- MS 30 m×0.25 mm id×250 nm df) which is the default column in our laboratory. Selected ions are monitored for each target compound to increase sensitivity and to reduce interferences. This work investigates the applicability of the approach outlined above to substances beyond azide, cyanide and fluoroacetate. These include, nitrite, nitrate, sulfide, iodine (as iodide), thiocyanate, and arsenic (III).

*Keywords:* derivatization, GC-MS, headspace, SPME
*Application code:* homeland security/forensics
*Methodology code:* gas chromatography/mass spectrometry

### COMPOUND CLASSIFICATION WITH MASS SPECTRAL DATA FILTERS FOR COMPREHENSIVE TWO-DIMENSIONAL GAS CHROMATOGRAPHY TIME-OF-FLIGHT MASS SPECTROMETRY (GCXGC-TOFMS)

***Mark F. Merrick,* Donald C. Hilton, Cochran Jack, Jon M. Ruppel, Jonelle Shiel, and Tincuta Veriotti***

**LECO Corporation, 3000 Lakeview Avenue, St. Joseph, MI 49085, USA*

Complex samples analyzed by comprehensive two-dimensional gas-chromatography–time-of-flight mass spectrometry (GC×GC-TOFMS) may contain hundreds or even thousands of peaks. In many applications only compound classifications are necessary rather than individual peak identifications. While GC×GC produces structured chromatograms which allow the grouping of compound classes, these groups can overlap each other and have odd shapes. However, with characteristic mass spectra these classifications can be further refined. Typically, extracted mass chromatograms are displayed with particular ions that are characteristic of a certain class of compounds. However, displaying only selected ions does not necessarily display the information desired. Take for example m/z 57 and alkanes. If m/z 57 is displayed, then any compound containing m/z 57 would be displayed, including those which are not alkanes. In order to be more selective, a script-based tool has been developed which allows data filters based on mass spectra and retention times to be defined by the user for specific classes of compounds. For example, the alkanes would be identified by a filter that identifies spectra having a base peak of m/z 43 or 57 and a second largest peak of m/z 57 or 71, and a second dimension retention time within a specified range. By employing such a tool, classes of compounds can be selectively identified, displayed, and reported. This presentation will describe a script language for identifying chemical classes based on their characteristic spectra and two-dimensional retention times, and an example will be shown demonstrating the power of such a tool.

*Keywords:* gas chromatography/mass spectrometry, GC-MS, identification, software
*Application code:* general interest
*Methodology code:* gas chromatography/mass spectrometry

### UNKNOWN IDENTIFICATION AND REPORTING THROUGH USE OF AUTOMATED SPECTRAL EXTRACTION SOFTWARE AND GAS CHROMATOGRAPHY/MASS SPECTROMETRY

***Trisa C. Robarge,* Jessie Butler, Jason S. Cole, Meredith Conoley, Jim Edwards, and Eric Phillips***

**Thermo Electron Corporation, 2215 Grand Avenue Parkway, Austin, TX 78728, USA*

Today's fast chromatography techniques, used in conjunction with gas chromatography/mass spectrometry (GC/MS), can result in an abundance of data for each sample. These data create analytical challenges for interpretation and reporting, particularly when compounds co-elute or when matrix components coincide with compounds of interest. Similarly, the data are time-consuming for analysts to evaluate manually. A software program that automates sample acquisition, analysis, and reporting was developed and evaluated for performance. This program functions with the instrument control to acquire sample data, extract spectral information, and report results within an automated framework. The program makes use of automatic spectral deconvolution software (AMDIS) to identify spectra and generate reports. To evaluate this program's performance, environmental, toxicological, and essential oils samples were acquired and processed using fast chromatography techniques via the program's user interface. Ease of use was measured by determining the time needed to develop methods and acquire and process data, while robustness was determined through consideration of instrument uptime as a function of sample throughput and report accuracy. The time needed by the program to generate a single report for a complex data file was measured and compared to both that of a manual search of the same file and of an automatic library search of the file. The decreased analytical time can then be extrapolated into a productivity function for the program. Overall, this program offered integrated sample analysis and reporting functions that enhanced productivity and sample throughput, within an easy-to-use framework.

*Keywords:* data analysis, gas chromatography/mass spectrometry, identification, software
*Application code:* general interest
*Methodology code:* gas chromatography/mass spectrometry

### GC/MS DATABASE FOR SEMI-QUANTITATION OF POLLUTANTS AND AGRICULTURAL CHEMICALS

***Yuki Sakamoto,* Kiwao Kadokami, Charles R. Lyle, Haruhiko Miyagawa, Katsuhiro Nakagawa, Kyoko Tanada, and Katsuyuki Taneda***

**Shimadzu Corporation, 1, Nishinokyo-Kuwabaracho Nakagyo-Ku, Kyoto, Kyoto 8040082, Japan*

Although approximately a hundred thousand compounds are used in the world, the number of pollutants that are regulated is limited. The determination of the unregulated compounds is very important for close inspection of environmental pollution by chemicals. It becomes more difficult for each environmental laboratory to obtain these toxic standard chemicals. The database was developed for 583 compounds and consists of the retention time, the mass spectrum, the mass number that is specified for each compound and four-level calibration curve. The GC/MS performance check standard sample was also developed to determine more precise results using this database. After adjusting the GC inlet, the capillary column and the mass pattern, this sample is analyzed and the performance of the GC/MS is confirmed. For the peak identification of unknown samples, the retention time and mass spectrum are used. The retention time depends on the capillary column even if the same specification capillary column is used. The software will automatically correct the retention times in the database using the retention time of *n*-alkanes. Another feature is the increased accuracy of the identification of these compounds using the mass spectrum reverse search function. The identified compounds are determined using the multi-internal standard method. One hundred standard chemicals at 1 ug/mL were analyzed to evaluate the performance of this database. The retention times of the registered compounds were predicted with an error of less than 3 seconds. The similar index of the reverse search is more than 80%. All were detected automatically. Relative standard deviations of the determination values were 20% or less. From these results, the registered pollutants can be simultaneously determined using the database instead of using standards.

*Keywords:* data base, environmental analysis, GC-MS
*Application code:* environmental
*Methodology code:* gas chromatography/mass spectrometry

### DYNAMIC HEADSPACE-GC/MS ANALYSIS OF THE AROMA OF FRESH FLOWERS

***Thomas P. Wampler,* Leticia A. Mancini, and Charles P. Zawodny***

**CDS Analytical, Inc, 465 Limestone Road, Oxford, PA 19363-0277, USA*

Aroma compounds from botanicals like herbs and flowers have been collected, enjoyed, and even analyzed for many years. The collection and analysis of such compounds, however, frequently involves distillation, extraction or other processes which may alter the content and nature of the compounds determined in the actual analysis. Further, if an extract is concentrated by evaporating some of the solvent, volatile constituents may be lost as well. Ideally, one would sample only the volatiles which actually pass into the air around the plant and create the characteristic aroma. Static headspace can collect such volatiles, but is limited in sensitivity since only a small volume may be injected into the gas chromatograph. Dynamic headspace is a technique which takes the volatile compounds and concentrates them onto a sorbent trap, so that the equivalent of several hundred milliliters of air may be transferred to the GC. This substantially increases the sensitivity of the analysis, without introducing solvents from an extraction. This paper presents the mass spectrometric identification of compounds collected from flower fragrances, showing differences between natural flowers as well as comparisons to commercial flower-based aroma products.

*Keywords:* flavor/essential oil, gas chromatography/mass spectrometry, headspace, volatile organic compounds
*Application code:* general interest
*Methodology code:* gas chromatography/mass spectrometry

### AN APPLICABLE DESIGN OF "SPOT-ON-A-CHIP" MALDI-MS SAMPLE SUPPORTS

***Guilin Jiang***

*Brigham Young University, C100 Bnsn, Provo, UT 84602, USA*

An applicable design of "spot-on-a-chip" matrix-assisted laser desorption/ionization mass spectrometry (MALDI-MS) sample supports aimed at improving reproducibility and sensitivity in MALDI-MS analysis was investigated. This design exploits hydrophobic-hydrophilic interactions to focus analyte-matrix droplets onto 200 micron diameter sample supports, which, instead of using complicated photolithographic micromachining methods, were scribed/burnt with a home-built scriber/laser on a silanized hydrophobic silicon substrate. The MALDI-MS analysis results for several test peptides with this design of MALDI-MS sample supports showed increased reproducibility and sensitivity as expected. Because the silanization of silicon substrate can be performed in a common laboratory oven in 15 minutes, and the subsequent scribing/laser burning of the sample supports can be finished in a couple of minutes, it is possible that one can make MALDI-MS sample supports of this design easily and quickly at low cost in a typical laboratory. Thus, this design would be applicable for routine MALDI-MS analysis.

*Keywords:* analysis, mass spectrometry, peptides
*Application code:* bioanalytical
*Methodology code:* mass spectrometry

### CHEMICALLY MODIFIED NANOPORE ELECTRODES

***Henry S. White,* Andrew Bohaty, Ryan J. White, Bo Zhang, Yanhui Zhang, and Ilya Y. Zharov***

**University of Utah, Department of Chemistry 315 S 1400 E Rm 2020, Salt Lake City, UT 84112, USA*

The fabrication and electrochemical characterization of truncated cone shaped nanopore electrodes are reported. A nanopore electrode is a Pt disk electrode embedded at the bottom of a conical pore, the circular orifice of the pore having nanometer dimensions. Our interest in the nanopore electrode is in developing a structurally simple and reliable platform for investigating molecular transport through orifices of nanoscale dimensions. The truncated cone-shape pore electrode possesses a unique transport property—the steady-state flux of molecules into a deep pore is limited by the restriction near the pore orifice and, thus, the steady-state current is independent of the pore depth. This characteristic is potentially useful in studying transport through nanometer-scale orifices. Chemical modification of the pore wall allows selective passage of molecules through the pore to the electrode surface.

*Keywords:* electrochemistry, microelectrode, modified silica, voltammetry
*Application code:* nanotechnology
*Methodology code:* electrochemistry

### MULTIVARIATE ANALYSIS OF ACCURATE-MASS LC-TOF MS DATA OF COUNTERFEIT DRUGS

***Facundo M. Fernandez,* Krystyn Alter, Leonard Arthur, Michael Green, and Paul Newton***

**Georgia Tech, School of Chemistry and Biochemistry 770 State St. Boggs Bldg, Atlanta, GA 30332, USA*

Drug counterfeiters, especially in countries where effective law enforcement is lacking, not only defraud consumers, but also deprive ill patients from therapies that can cure them. The inhuman act of drug counterfeiting is so common in some third world countries such that in some areas there is a greater probability of getting a fake drug rather than a real one. The antimalarial drug artesunate which is the recommended treatment for multidrug-resistant P. falciparum malaria in most of South East Asia has fallen prey to a sophisticated counterfeit drug trade. Fifteen samples of the suspected fake tablets were analyzed by accurate-mass LC-MS using both positive and negative-mode electrospray. In order to explore the similarities between the counterfeit drug samples we applied multiway principal component analysis to the LC-MS intensity data "cube" formed by retention time versus mass-to-charge ratio vs. sample number. The information obtained after multivariate clustering suggests that samples from the same geographical origin tend to have similar chemical composition. One of such clusters was formed by drugs that contained the active ingredient artesunate in substandard concentrations. Another cluster was formed by samples containing a "wrong" active ingredient. Using accurate mass measurements in an orthogonal extraction-TOF MS, combined with in-source CID, NIST database searches and injection of standards, we have positively identified this “wrong” ingredient as erythromycin, a common antibiotic. Samples from Cambodia were found to be chemically different than the majority of samples from Laos. Another very distinct cluster formed by only one sample, showed an unusually high concentration of erythromycin.

*Keywords:* drugs, liquid chromatography/mass spectroscopy, pattern recognition, time of flight MS
*Application code:* pharmaceutical
*Methodology code:* liquid chromatography/mass spectrometry

### NANOSCALE OPTICAL BIOSENSORS

***Richard P. Van Duyne,*** [*] ***Amanda J. Haes, Douglas A. Stuart, Chanda R. Yonzon, and Xiaoyu Zhang***

[*] *Northwestern University, 2145 Sheridan Road, Department of Chemistry, Evanston, IL 60208-3113, USA*

Fundamental and applied research in the field of nanoparticle optics has dramatically increased in recent years. This talk will emphasize applications of nanoparticles and nanostructures fabricated by nanosphere lithography (NSL) techniques. NSL is an inexpensive, simple-to-implement, inherently parallel, high-throughput, materials general nanofabrication technique capable of producing an unexpectedly large variety of nanoparticle structures. Specifically, this presentation will address novel research on triangular nanoparticles and metal film over nanosphere (FON) nanostructures, which exhibit tunable localized surface plasmon resonances (LSPR). The properties of LSPR are sensitive to a number of factors, including the dielectric constant of the surrounding environment, including adsorbed analytes. Sensitive, label-free detection can be accomplished using NSL-fabricated nanoparticles decorated with self-assembled monolayers (SAMs) with specific ligand/receptor interactions. The FON surfaces make remarkable stable, rugged and uniform substrates for surface-enhanced Raman spectroscopy (SERS). These substrates are ideal platforms for sensing applications, such as anthrax detection and glucose sensing.

*Keywords:* biosensors, materials science, nanotechnology, spectroscopy
*Application code:* bioanalytical
*Methodology code:* sensors

### NANOTUBE-BASED ARTIFICIAL VOLTAGE-GATED ION CHANNELS

***Charles R. Martin***

*University of Florida, 218 Chemistry Lab Building PO Box 117200/218 Leigh Hall, Gainesville, FL 32611, USA*

Voltage-gated ion channels are protein pores that span cell membranes; they regulate electrical signaling in nerve and muscle cells by opening and closing in response to changes in the transmembrane potential. When open/on, ions pass through the pore, and hence across the cell membrane, and when closed/off, ion transport is precluded. There is intense research effort aimed at understanding the molecular-level mechanism for this voltage-gating function. While the details are still being debated, it is clear that the mechanism entails physical movement of an ionically charged portion of the channel in response to a change in the transmembrane potential. We have prepared artificial ion channels that were designed to voltage gate via this general mechanism. These artificial channels consist of a polymeric membrane containing a single conically shaped gold nanotube with single-stranded DNA molecules attached to the nanotube surfaces. We propose that this device voltage gates because the anionic DNA chains get electrophoretically driven into (off state) and out of (on state) the small-diameter opening of the nanotube in response to changes in the transmembrane potential.

*Keywords:* nanotechnology
*Application code:* nanotechnology
*Methodology code:* sensors

### NANOMATERIAL-BASED BIOELECTRONIC DETECTION OF PROTEINS AND DNA

***Joseph Wang***

*Arizona State University, Department of Chemical and Materials Engineering Ira A. Fulton School of Engineering, Tempe, AZ 85287-6006, USA*

The unique properties of nanoparticle-based materials, and the versatility of microspheres, in general, offer excellent prospects for electrochemical detection of DNA and proteins. This presentation will describe new multiamplification/multi-tag particlebased bioelectronic assays based on a variety new biomaterial-nanoparticle assemblies. In particular, combining the catalytic enlargement of the metal-particle tags with the effective “built-in” amplification of electrochemical stripping analysis paved the way to remarkably low (fmol) detection limits. New platforms for carrying numerous redox tags, including carbon nanotubes and polystyrene beads, will be discussed in connection to ultrasensitive detection of proteins and nucleic acids. The high sensitivity of the new protocols was combined with an efficient magnetic separation. Such use of magnetic beads has been extremely useful for discriminating against unwanted constituents, including a large excess of co-existing mismatched and noncomplementary oligomers, chromosomal DNA, RNA, and proteins. TEM imaging has indicated that the DNA hybrid links the metal nanoparticles to the magnetic beads. No such aggregates were observed in the presence of noncomplementary or mismatched DNA. A new electrochemical coding bioassay, based on different inorganic-colloid (quantum dots) nanocrystal tracers, whose metal components yield well-resolved highly sensitive stripping voltammetric signals for the corresponding targets, will be described. The use of DNA recognition for designing nanoscale assemblies with tailored properties will also be discussed.

*Keywords:* nanotechnology
*Application code:* proteomics and genomics
*Methodology code:* electrochemistry

### SPECIATION ANALYSIS TODAY: STATE-OF-THE-ART, LEGISLATION AND INDUSTRIAL APPLICATIONS

***Michael Sperling***

*University of Muenster, European Virtual Institute For Speciation Analysis Westfälische Wilhelms-Universität Münster, Muenster D-48149, Germany*

Today, there is little doubt within the community of researchers that the role of speciation is crucial to answer the demanding questions about the activity of trace metals and metalloids, such as their bioavailability and mobility, biological activity and metabolism, toxicity or nutritional value, to name only some. The important role of speciation analysis is well established and visible through the high publication rate in that area that has surpassed 500 publications/year. However, despite all the developments in this area during the last decades, speciation is not yet well established in industrial analysis and other ideal world applications and is far away from being performed on a routine basis. Also, despite the fact that elemental information is not good enough to answer all questions about the activity of elemental species, very few rules, regulations, or laws require to get or use the necessary information about speciation. One of the reasons for this discrepancy is the lack of an efficient link between research scientists and potential users, regulators and policy makers, resulting in insufficiently organized and synthesised information for their decision making process. In order to improve this situation, a project supported by the European community has been launched to fill this gap by combining the expertise of some of the most renowned research laboratories, industrial users, governmental facilities, and manufacturers. The principal aim of the project that manifests itself by the establishment of a European Virtual Institute for Speciation Analysis (EVISA) is to facilitate the transfer of the knowledge collected within the Speciation Scientific Community to potential users facing ideal world problems in industry, food, and environmental issues and to facilitate its integration in far more effective legislative actions. The approach to be taken by EVISA will be briefly presented.

*Keywords:* HPLC, ICP-MS, other hyphenated techniques, speciation
*Application code:* other
*Methodology code:* other

### HOW CAN WE USE SPECIATION METHODS TO CHARACTERIZE AND MINIMIZE TRACE ELEMENT EMISSIONS FROM INDUSTRIAL PROCESSES?

***Dirk Wallschläger***

*Trent University, Environmental & Resource Sciences Program 1600 West Bank Drive, Peterborough, ON K9J 6X5, Canada*

Many large-scale industrial processes, including base and noble metal mining, petroleum refining, fossil fuel combustion, and chemical manufacturing, inadvertently lead to sometimes significant emissions of trace elements into the environment. Several of the elements that carry the highest environmental significance, such as mercury, arsenic, selenium, and chromium, form a number of chemical binding forms ("species") which are stable enough to be separated by chromatographic procedures. These individual species of one trace element usually have significantly different chemical and toxicological properties, thus warranting designated speciation analyses for refining risk and characterizing environmental distribution and fate. Furthermore, different species of one element usually respond differently to treatment procedures utilized to reduce its concentration in industrial waste water streams and/or its mobility in solid by-products. Therefore, knowledge of trace element speciation in these waste streams is important in view of designing and optimizing treatment procedures, sometimes to the point where each species may have to be treated as a separate chemical. Only by careful application of this approach can the maximum removal efficiencies be achieved and guaranteed for every individual discharge source, and thus emissions to the environment and effects on exposed organisms be minimized. In this presentation, I will demonstrate how state-of-the-art hyphenated speciation methods (typically LC-ICP-MS) can give accurate speciation information for trace elements in complex matrices. Then, I will show a number of real-world examples illustrating typical trace element speciation patterns arising from various industrial processes, and finally discuss the performance of common waste treatment strategies for individual species of one element. The success (or failure) of some treatment techniques will be demonstrated using industrial examples.

*Keywords:* environmental/waste/sludge, environmental/water, ICP-MS, ion exchange
*Application code:* environmental
*Methodology code:* liquid chromatography/mass spectrometry

### MAINTAINING (GROWING?) OUR BUSINESS: UNDERSTANDING, MARKETING, SUSTAINING

***Stuart G. Bush***

*Rohm and Haas Company, PO Box 904, Spring House, PA 19477-0904, USA*

Trying to grow a support function in the current industrial climate is somewhere between challenging and insane. This talk will focus on key considerations in maintaining (and yes growing!) our business. It is critical to first understand the "business of analytical" from the global and corporate business environments to the business model. The latter includes both delivering the service/product line and maintaining the ability to serve. We must also understand the details of our cost structure and those of our customers: what can we deliver at what price and what is the opportunity? From this base of knowledge, we can design and implement targeted outreach/marketing to our customers. Corporate culture plays a big role here as we tailor our message and deliver it using the full range of communication options available. While moving forward with our marketing, it is critical to close the loop and not to oversell: nondelivery and other service errors are hard to recover from and can puncture any short-term gains and hurt sustainability.

*Keywords:* lab management
*Application code:* laboratory management
*Methodology code:* other

### BEST PRACTICES IN ASSESSING CUSTOMER SATISFACTION

***Dennis B. Crawshaw***

*International Paper, 6283 Tri-Ridge Boulevard, Loveland, OH 45140, USA*

Analytical laboratories need to have active continuous processes/tools in use to assess how their customers perceive the value, quality, and impact of the laboratory's work. These tools include feedback surveys sent with individual analytical reports, a more formal comprehensive annual survey of major customers, interviews, and website feedback mechanisms. Areas probed include the quality and timeliness of measurements and reports, the laboratory's technical knowledge and problem-solving skills, ability to provide innovative solutions, interactive communications, knowledge of the customer's business, and instrumental capabilities. Information provided by these tools allows the laboratory to identify areas of strength and where improvement is needed, plan staffing and capital projects, monitor utilization of its resources, demonstrate and increase value provided to customers, and recognize contributions by its analysts. Customer satisfaction "scores" may be incorporated into laboratory and analyst work objectives and performance assessments, to drive increased customer focus, service, and satisfaction.

*Keywords:* lab management
*Application code:* laboratory management
*Methodology code:* other

### INVESTING IN EMPLOYEE DEVELOPMENT

***Andrew G. McFarland***

*ATOFINA Chemicals, Inc, 900 First Avenue, King of Prussia, PA 19406, USA*

Yearly employee development plans based upon the management by objectives principle are very effective in understanding the personal and career goals of each staff member as well as ensuring their continual growth in nontechnical and scientific areas. The process of developing the plan and assisting the chemist in achieving each goal is as important as the actual goals that are established. For example, regular coaching and interim semiformal reviews greatly increase the number of goals realized while decreasing the timeframe for their accomplishment. This presentation will provide guidelines for developing the plan and working with each chemist to maximize their achievement, growth, and satisfaction.

*Keywords:* lab management
*Application code:* laboratory management
*Methodology code:* other

### SHOULD I STAY OR SHOULD I GO? THE DO'S AND DON'TS OF KNOWLEDGE WORKER MANAGEMENT

***Jo Dohl***

*Health, PO Box 4220, Nydalen, Oslo 0401, Norway*

The presentation will review the changes, which have occurred in the roles of leaders of technical and skilled people due to various factors such as globalization trends, focus on personal growth and career and employment as a temporary relationship. Suggestions on how to deal with these changes as a means to provide appropriate leadership to the knowledge work will be offered.

*Keywords:* lab management
*Application code:* laboratory management
*Methodology code:* other

### PRIORITIZATION SYSTEMS THAT REALLY WORK TO IMPROVE LAB PRODUCTION

***Joe Wiegel***

*Novidea, Inc, 14260 Middleham Lane, Jacksonville, FL 32223, USA*

It only takes one analytical result that is delayed by a bottleneck to cancel out all the other timely results and make the entire report late to the customer. Bottlenecks are a reality in a laboratory, but there are prioritization systems that really work to manage them and improve lab production. This presentation discusses how analyzing bottlenecks and setting up priority systems that coordinate workflow with demand will help labs improve on-time delivery, reduce work in progress, and increase throughput. The use of LIMS as a tool to coordinate priorities across departments will also be discussed.

*Keywords:* lab management, LIMS
*Application code:* laboratory management
*Methodology code:* laboratory informatics

### ELECTROTRANSFECTION OF MAMMALIAN CELLS USING MULTICHANNEL ELECTROPORATION MICROCHIP

***Jeong Ah Kim,*** [*] ***Jun Keun Chang, Keunchang Cho, Chanil Chung, Sun Hee Lim, Chan Hee Park, and Young Shik Shin***

[*] *Digital Biotechnology Company, Institute of Advanced Machinery & Design, Seoul National University, San 56-1, Seoul 151-742, Korea*

Electroporation is a widely used method to enhance the cellular uptake of little permeable molecules. We developed an electroporation microchip made of polydimethylsiloxane (PDMS) for the gene transfer experiment of mammalian cells. The electroporation microchip has many advantages over conventional electroporation cuvettes as follows. The narrow microchannels of microchip confine the electric field and produce the uniform electric field. The separation of electrodes and reservoirs in microchip can use reusable platinum electrodes for electrical connection. There is neither hydrogen evolution owing to water dissociation nor harmful metal ions dissolved in electroporation buffer during electroporation. Owing to the transparency of PDMS, we could in situ observe the uptake process of propidium iodide or plasmid into cells, which suggests a convenient visualization technique of gene delivery in living cells. Only a small amount of cells are necessary. The various microchip designs can implement the electroporation functionalities unobtainable from conventional systems. Our multichannel electroporation chip gives multiple electric field gradients in a single microchip, which helps researchers to optimize the experimental conditions faster. The microchip has five microchannnels of different length and share two reservoirs at the ends of the microchannels each other. Therefore, different electric fields are generated even with a single electric pulse. We evaluated the effects of various combinations of electric pulses of different voltages and the channel width, concerning the cell permeabilization and the DNA transfection in the mammalian cells. We successfully transferred enhanced green fluorescent protein genes into SK-OV-3, HEK-293, and CHO cells and cultured the cells within the microchip. These results were compared with those of the conventional electroporation system. This electroporation microchip can be a novel tool for gene transfection research.

*Keywords:* bioanalytical, biotechnology, genetic engineering, microfluidics/lab-on-a-chip
*Application code:* bioanalytical
*Methodology code:* microfluidics/lab-on-a-chip

### REAL-TIME TUNING MEMBRANE TRANSPORT OF SINGLE LIVING CELLS USING ELECTRIC FIELDS

***Qian Wan,*** [*] ***Karl Schoenbach, and Nancy Xu***

[*] *Department of Chemistry & Biochemistry, Old Dominion University, 4541 Hampton Baulevard, Norfolk, VA 23529, USA*

Tuning of cellular membrane transports can be used to control the cellular pathways and the cellular function in living cells. It is well known that electric fields can be used to change the cellular membrane transport. However, it still remains unclear how the accumulation of the electric fields affects the membrane transport of subcellular compartments. This is a crucial question to be addressed in order to use the electric field to control the cellular and subcellular functions. In this study, we used an array of electric field strength and pulse intervals to investigate the accumulation effects of electric fields on cellular and subcellular membrane transport using real-time fluorescence microscopy and spectroscopy. The results show that the membrane transport highly depends on the electric field strength and pulse intervals, demonstrating that the cellular and subcellular membrane transport can be changed by timing the electric field strength. This study shows the promise of tuning of cellular and subcellular membrane transport by controlling the timing of pulses.

*Keywords:* bioanalytical, biotechnology, imaging
*Application code:* biomedical
*Methodology code:* fluorescence/luminescence

### REAL-TIME MONITORING OF SUBCELLULAR MECHANISM INDUCED BY ELECTRIC FIELDS

***Qian Wan*** [*] ***and Nancy Xu***

[*] *Department of Chemistry & Biochemistry, Old Dominion University, 4541 Hampton Boulevard, Norfolk, VA 23529, USA*

It is well known that electric field can affect the cellular membrane transport. However, it remains essentially unknown how the electric field induces the change of cellular and subcellular mechanisms, leading to the transformation of membrane permeability. The understanding of such mechanisms offers the possibility of selectively controlling the specific cellular and subcellular functions to eliminate unwanted cells (e.g., tumor), which holds the promising of effective therapy. In our study, we use a new technique, recently developed in our lab, to explore the possibility of selective eliminating tumor cells by tuning electric field strength and pulse intervals. The results show that we can control the fate of cells by timing the membrane transport using electric fields, showing the possibility of effective therapy using electric fields. The experimental approach, updated research results, and prospective applications will be discussed in detail.

*Keywords:* bioanalytical, biotechnology, imaging
*Application code:* biomedical
*Methodology code:* electrochemistry

### DATA PROCESSING OF VOLTAMMETRIC MEASUREMENTS BASED ON THE HILBERT TRANSFORM: THEORY AND EXPERIMENTS

***Costas A. Anastassiou,*** [*] ***Danny O'Hare, and Kim H. Parker***

[*] *Department of Bioengineering, Imperial College, London SW7 2AZ, UK*

A plethora of processes involve species that undergo redox reactions at electrodes. Hitherto, many researchers have been applying amperometry which by default cannot discriminate between different species. Voltammetry has been shown to offer improved selectivity in electroanalysis as well as improved discrimination against nonfaradaic contributions to the signal. Microelectrodes enable the accurate measurement of time-dependent currents and have proven to be a very useful tool for in vivo experiments offering unparalleled spatial and temporal resolution. Electrochemical signals are intrinsically nonlinear and nonstationary. Hitherto, there have been few alternatives to the fast Fourier transform technique for frequency analysis although fundamental assumptions of the theory, namely, periodicity, continuity, and linearity, are not satisfied. The Hilbert transform (HT) offers an alternative tool of data analysis that can overcome these difficulties. It creates instantaneous attributes of the analytical signal to give phase angle and amplitude information and provides a viable method for nonstationary, nonlinear signal processing whilst maintaining time-domain information. Numerical simulations of various limiting processes (mass transport control, heterogeneous reaction control, etc.) as well as intermediate cases on microdisc electrodes will be presented for a number of excitation waveforms. Analysis of the current response with the HT will show that for thin-film processes kinetic and thermodynamic parameters can be estimated with great precision using AC voltammetry and pattern recognition. We will attempt the same characterisation for the more complex diffusion-reaction processes. An additional advantage of this technique is that it is does not involve background subtraction even for high charging currents. We will support our theoretical analysis with experimental measurements showing how our analysis can be applied on electrochemical processes.

*Keywords:* data analysis, microelectrode, pattern recognition, voltammetry
*Application code:* bioanalytical
*Methodology code:* data analysis and manipulation

### MARKUSH STRUCTURE HANDLING, STORAGE, AND SEARCHING

***Markus Hemmer***

*Waters Informatics, Europaallee 27-29, Frechen 50226, Germany*

The handling of Markush structures throws up many problems that are difficult to overcome with conventional chemical structure handling software. The increasing demand from the patent side of the discovery process to not only use Markush structures in a printed deposition process but also to be able to fully integrate Markush-type information into knowledge management systems has made this a very hot topic. Not only is the storage of such structures complex but new search strategies and especially important new visualization tools have needed to be developed to meet the demands of a modern laboratory informatics environment. This talk will review the current state of the art and present a new more user-friendly approach.

*Keywords:* computers, data mining, laboratory informatics, scientific data management
*Application code:* pharmaceutical
*Methodology code:* data analysis and manipulation

### OPENING THE ROAD TO REUSE AND LONG-TERM ASSET PROTECTION FOR SCIENTIFIC AND TECHNICAL DATA

***Richard S. Lysakowski***

*CENSA/GERA, 800 West Cummings Park, Suite 5400, Woburn, MA 01801, USA*

CENSA has initiated a new standards program called the OpenScienceAlliance(TM)(OSA), a fast-track program designed to create defacto (and where necessary de jure) standards for all data and records generated during experiments, so they are reusable, interoperable, XML-message-based archiveable data sets captured and stored in the expML(TM) standard language. expML(TM) codifies all experiment information in XML data object formats and high-quality rendered XML/PDF document formats that are open for any software users to reuse short-term and legal and regulatory professionals to depend upon as long-term reusable records. The CENSA-defined standard expML(TM) language is an XML-based experiment information container markup language that is well defined, comprehensive, flexible, and extensible. This critical interface standard facilitates message-based, loosely-coupled, component-based integrated solution architectures so that for the first time in automation history we can easily construct collaborative eR&D systems from components from multiple suppliers. We will leverage anything that is publicly available and independently validated to create an open, supported standard for short-term reuse and long-term archiving of experiment data sets. This presentation will discuss recent progress on this developing standard and alliances formed during its development. The OSA program and expML(TM) container language includes as deliverables selection criteria for compatible standards, rules to permit facile evolution and validation of expML(TM), compatible expML(TM) web services, training materials, and guides for buyers and suppliers (educational materials, implementation guides, procurement guides and templates, etc.). We will discuss progress and driving forces expected to bring expML(TM) into widespread adoption via the purchasing community within the near future.

*Keywords:* informatics, laboratory automation, LIMS, software
*Application code:* general interest
*Methodology code:* data analysis and manipulation

### MICROBIOLOGICAL FUNCTIONS IN LIMS

***Christine Paszko,* Robert Cattolica, Rick Danielson, and David Schlabach***

**Accelerated Technology Laboratories, Inc, 496 Holly Grove School Road, West End, NC 27376, USA*

Traditionally LIMS (laboratory information management systems) have been deployed in analytical laboratories that performed primarily chemical testing. As a result most commercially available LIMS are not equipped to handle the often subjective nature of the results of microbiological analyses. This presentation will focus on the incorporation of several microbiological tests incorporated into the LIMS. The first test is the most probable number (MPN) calculation for the detection and quantification of microorganisms. This test has been automated so that end-users no longer need to consult reference charts, the calculations are performed within the LIMS. Automating these features reduces the potential for errors that result form the manual interpretation of charts and increases productivity. Another automated function that was incorporated included the automation of Giardia and Cryptosporidium calculations, uses can enter in raw microscopic counts along with the information on the volume of sample that was analyzed and what was filtered and the LIMS automatically calculates the concentration of cysts or oocysts for the analysts and places this information on the final analysis report. This presentation will discuss the process and the numerous advantages to laboratories that have automated integration of MPN, Giardia and Cryptosporidium calculations in the LIMS.

*Keywords:* automation, data analysis, informatics, LIMS
*Application code:* bioanalytical
*Methodology code:* data analysis and manipulation

### TOTAL LABORATORY INFORMATICS FROM DISCOVERY THROUGH DEVELOPMENT TO BATCH RELEASE AND PAT

***Antony N. Davies***

*Waters Informatics, Europaallee 27-29, Frechen 50226, Germany*

The rapid advancement in computing power available to the laboratory informatics field has finally made it possible to envisage bringing all relevant laboratory information content online and available in real time. It is irrelevant whether the information is instrumental or human in origin and this resource can be keep alive for the support of the whole corporate organization whether in discovery/research, development, or production. This talk will show how this can be achieved in practice with real working examples from industry and highlighting how these systems support the drive towards real-time batch release through the intelligent implementation of process analytical technology.

*Keywords:* laboratory informatics, process analytical chemistry, sample and data management, software
*Application code:* laboratory management
*Methodology code:* data analysis and manipulation

### PROCESS ANALYTICAL TECHNOLOGY (PAT): WHAT'S IN A NAME?

***Chris Watts***

*FDA/CDER/OPS, Rockwall Building, HFD-003, 5515 Security Lane, Rockville, MD 20852, USA*

Conventional pharmaceutical manufacturing is generally accomplished using batch processing with laboratory testing conducted on collected samples to evaluate quality. This conventional approach has been successful in providing quality pharmaceuticals to the public. However, significant opportunities exist for improving pharmaceutical development, manufacturing, and quality assurance through innovation in product and process development, process analysis, and process control. Unfortunately, the pharmaceutical industry generally has been hesitant to introduce innovative systems into the manufacturing sector for a number of reasons. One reason often cited is regulatory uncertainty, which may result from the perception that our existing regulatory system is rigid and unfavorable to the introduction of innovative systems. In August 2002, recognizing the need to eliminate the hesitancy to innovate, the Food and Drug Administration (FDA) launched a new initiative entitled "Pharmaceutical cGMPs for the 21st century: a risk-based approach." The agency's PAT guidance was released as part of this initiative. The FDA's PAT guidance explains a science-based, risk-based framework, "process analytical technology," or "PAT," founded on process understanding to facilitate innovation and risk-based regulatory decisions by industry and the agency. This presentation will discuss various elements of PAT, including the agency's approach to regulation, as well as examples of implementation.

*Keywords:* pharmaceutical, process control, process monitoring
*Application code:* pharmaceutical
*Methodology code:* other

### PROCESS ANALYTICAL TECHNOLOGY (PAT): AN INDUSTRY PERSPECTIVE

***Sigmund M. Waraszkiewicz***

*AstraZeneca, 50 Otis Street, Westborough, MA 01581, USA*

In many aspects, process analytical technology (PAT) today is like Y2K was in 1999 and 21 CFR Part 11 (Electronic Records/Electronic Signatures) has been since its publication in 1997, it is included as a topic at every recognized seminar, conference, or workshop for the pharmaceutical industry. While addressing Y2K was relatively straightforward, PAT, like 21 CFR Part 11, has not been as straightforward. The concept of PAT was identified by the Swedish R&D Group in 1985 and some limited at-line process analysis was performed in 1987. Low-level PAT activities continued until 1999. Today PAT applications have been implemented for approximately 50% of solid oral dosage form manufacture, but almost exclusively at 2 major manufacturing sites in Europe. The initiative to implement PAT at the US manufacturing sites was announced in July 2001. Following the decision to implement PAT at the US manufacturing sites, a product was selected as the "pilot". The product was a high-volume immediate release tablet, manufactured using a traditional wet granulation process. Today, approximately 3 years after the announcement of the US PAT initiative, and 2 years after the "kickoff" in the US, we are in the data-gathering phase. The slow progress in the US has not been as a result of the technology associated with PAT, but predominantly due to the time associated with conveying a basic understanding of PAT throughout the organization and the need to coordinate the resources and activities of many different functional areas.

*Keywords:* chemometrics, near infrared, pharmaceutical, process analytical chemistry
*Application code:* process analytical chemistry
*Methodology code:* near infrared

### TAKING PROCESS ANALYTICAL TECHNOLOGY FROM RESEARCH TO MANUFACTURING AND VICE VERSA

***Andrew J. Lange***

*Pfizer, Inc, Global Research and Development, 10777 Science Center Drive, San Diego, CA 92121, USA*

In the chemical industry, process analytical measurements of solids play a relatively smaller role than do analyses of gases and liquids. In pharmaceutical production, the reverse is true. Another key difference in focus between these two industries is emphasis on continuous versus batch production. In this talk, a range of examples of process analytical technology (PAT) as applied to chemical and pharmaceutical unit operations will be discussed, including approaches to sample presentation. Philosophical and strategic differences between research and manufacturing approaches to the use of PAT in the pharmaceutical industry will also be discussed.

*Keywords:* near infrared, pharmaceutical, process analytical chemistry, process monitoring
*Application code:* process analytical chemistry
*Methodology code:* other

### STRATEGIES FOR SUCCESSFUL IMPLEMENTATION OF PAT IN PHARMACEUTICAL MANUFACTURING

***Walter Dziki***

*Abbott Laboratories, Inc, Dept. R4R9/ Bldg R13, 1401 Sheridan Road, North Chicago, IL 60064-6293, USA*

Successful implementation of PAT in pharmaceutical manufacturing involves formation of a multidisciplinary PAT Team with members including analytical chemists, process chemists/scientists, process engineers, a statistician, the Information Technology Group and QA, as well as application engineers and chemists from instrumentation manufacturers to decide on the right technology for an application. Implementation should be broken down into manageable concepts of analytical component of PAT or "PaT" (process analytical technology) and the control component of PCT or "PcT" (process control technology). PaT can be defined as on-line, in-line, at-line or off-line (lab rapid) technologies used to predict the results of an analytical test. PcT can be defined as automated on-line or in-line technologies that use process critical control parameters to control a manufacturing process through a feedback loop. In addition, it is important to have management support and be able to fit PAT into the business model, and educate key individuals involved with analytical and product development to the importance of PAT. A focused, multistep approach can be most beneficial for implementation of PAT for those new to it.

*Keywords:* pharmaceutical
*Application code:* pharmaceutical
*Methodology code:* other

### THE ROLE OF A CENTER OF EXCELLENCE IN DEVELOPING PAT APPLICATIONS

***Nils-Erik Andersson,** *Staffan Folestad, and Arne Torstensson***

**AstraZeneca, R&D Mölndal S-431, Mölndal 83, Sweden*

The development of an innovative competence and the successful introduction of process analytical technologies (PATs) within an organization require a coordination of the activities from the R&D stage right through to the commercial manufacturing stage. One successful approach is to employ the concept of a centralized resource—a "center of excellence" (COE) for PAT. The COE approach includes invariable consideration of PAT to meet business needs in drug substance/product development, and manufacturing. PAT plays a role in bridging over between traditional scientific disciplines, for example, between traditional analytical chemistry and their customers, be it in process chemistry, pharmaceutical technology, or in pharmaceutical production. The COE is the scientific driving force in technology development and provides support for building local cross-functional PAT teams. The overall purpose is to support optimization of product quality and safety, process control and quality assurance, manufacturing economy and capacity, and to enable faster drug product development.

*Keywords:* pharmaceutical
*Application code:* pharmaceutical
*Methodology code:* data analysis and manipulation

### REALIZING THE BENEFITS OF AND CHALLENGES TO PROCESS ANALYTICAL TECHNOLOGY (PAT) IMPLEMENTATION: A GENERIC INDUSTRY PERSPECTIVE

***Su-Chin Lo***

*Barr Laboratories, Inc, 2 Quaker Road, PO Box 2900, Pomona, NY 10970, USA*

The implementing process analytical technology (PAT) is a hot topic within the pharmaceutical manufacturing industry today. This new approach encompasses a variety of technologies, methodologies, and tools designed to shift the industry's quality focus from postproduction inspection to building quality into its products. The outcomes will provide a more robust process, consistent process performance, cost reduction, and efficiency production for the manufacturer. However, numerous challenges prevent the generic pharmaceutical companies from fully implementing PAT into the manufacturing processes. The current challenges of PAT implementation in a generic drug company include

(1) shorter new product developing time that may not be accumulating more data for the process understanding or generating robust model in the early development stage,
(2) multiproduct facilities and equipments that require a universal unit operation-based technology instead a permanent installed analyzer for a specific application,
(3) shorter existing product life cycle that requires more resources and specialized skill for new PAT development cycle,
(4) regulatory uncertainty on both compliance and change of process on existing products.

This presentation will be focused on the practical issues and challenges of PAT in generic drug industry. A multistep approach and strategy on continuous improvement of existing product will also be illustrated.

*Keywords:* pharmaceutical, process analytical chemistry, process monitoring, validation
*Application code:* process analytical chemistry
*Methodology code:* vibrational spectroscopy

### MEETING THE REGULATORY CHALLENGES ASSOCIATED WITH PROCESS ANALYTICAL TECHNOLOGIES

***Thomas P. Garcia***

*Pfizer, Inc, Pfizer Global Research & Development, 78 Brookview Court, Groton, CT 06340, USA*

Process analytical technologies (PATs) have been utilized for many years across various industries with one notable exception—the pharmaceutical industry. However, in the last several years, the pharmaceutical industry's interest in PAT methodologies has increased substantially. The use of on-line, in-line, and at-line analyses provides an opportunity to gather substantially more information about a process behavior than may have been available using off-line and end of manufacture product release testing alone. The benefits of this increased knowledge come with many challenges in approaches to regulating products. The major hurdle often identified with preventing the adoption of these new technologies has been the regulatory framework overseeing the pharmaceutical industry. The regulatory challenges associated with the implementation of PATs and options to address them are presented.

*Keywords:* pharmaceutical
*Application code:* pharmaceutical
*Methodology code:* data analysis and manipulation

### PAT METHODS: INTEGRATION OF EQUIPMENT QUALIFICATION AND METHODS VALIDATION

***Carl A. Anderson***

*Center for Pharmaceutical Technology, Duquesne University, 600 Forbes Avenue, Pittsburgh, PA 15282, USA*

This session will focus on the qualification of analytical equipment for use in a method supporting process analytical technology (PAT). In particular, the relationship between qualification testing of sensors and the analytical requirements for the methods employing these sensors will be examined. Techniques for continuous sensor performance monitoring will be considered in addition to initial qualification testing.

*Keywords:* near infrared, pharmaceutical, process analytical chemistry, validation
*Application code:* pharmaceutical
*Methodology code:* other

### IMPACT OF PAT IMPLEMENTATION ON PRODUCT AND PROCESS SPECIFICATIONS

***Jean-Marie M. Geoffroy***

*Abbott Laboratories, Inc, D-049F, Bldg A4-4, 1401 Sheridan Road, N. Chicago, IL 60064-6235, USA*

Process analytical technology (PAT) is a logical extension of today's regulatory and scientific environment. PAT initially involves increasing the amount of in-process testing in order to gain a more fundamental understanding of product and process performance. PATs can help measure a myriad of physical and chemical attributes including traditional and nontraditional pharmaceutical properties. This presentation will discuss case studies where PAT has been applied and processes controlled as a result of these measurements. The implications on how product specifications should be approached will also be discussed.

*Keywords:* process analytical chemistry, process control, process monitoring
*Application code:* pharmaceutical
*Methodology code:* other

### THE ULTIMATE GOAL OF PAT: REAL-TIME RELEASE

***Thorsten Herkert*** **and Siegmund Kunz**

*AstraZeneca GmbH, Otto-Hahn-Strasse, Plankstadt 68723, Germany*

In September 2003, the FDA published the guideline "PAT—A Framework for innovative pharmaceutical manufacturing and quality assurance" which focuses on principles of the use of PAT (process analytical technology). The goal of PAT is to ensure final product quality by the use of a system for designing, analyzing, and controlling manufacturing. The development of a PAT system is based on in-depth process understanding combined with a product-specific risk assessment gained by development and manufacturing expertise. Using a complete PAT system in operations means that all production steps (critical for the product quality and/or process performance) are monitored online and the process equipment is controlled by direct feedback from analytical techniques out of the PAT toolbox in real time. Due to direct product measurements, the product itself—at each time of data acquisition—determines the process parameters between given ranges. For example, a validated fixed blending time will be obsolete, because the blending time is a function of particle size, shape, water content, and so forth. and therefore will vary due to the variation in raw materials. In a PAT system, the process will be stopped at the endpoint, detected by an analytical method. As a logical consequence, a more consistent product due to expertise in manufacturing is produced and a higher level of quality assurance verified by many PAT information elements about the product quality is reached. Process understanding and process control in combination with PAT information elements can provide all the required information to assure the product quality. Therefore traditional quality control methods will become redundant and PAT opens the door for RTR (real-time release) with all its benefits.

*Keywords:* near infrared, process analytical chemistry, process control, process monitoring
*Application code:* other
*Methodology code:* near infrared

### OPTIMIZING SOLID PHASE EXTRACTIONS: STRATEGIES AND USEFUL TIPS FOR GETTING THE BEST PERFORMANCE

***Stephen J. MacDonald*** **and Robert S. Johnson**

*Horizon Technology, Inc, 8 Commerce Drive, Atkinsion, NH 03079, USA*

Solid phase extraction (SPE) is the method of choice for many environmental samples. It is safer, faster, and more cost effective compared to liquid-liquid extractions (LLEs) because it can be automated and generates much less solvent waste. EPA methods such as 525.2, 8081, 8082, 8270, and 1664 are commonly performed by fast automated SPE. EPA Method 3535A coupled with the 8000 series of EPA methods is performance-based and provides a great deal of flexibility. The analyst is free to select the extraction disk, filtering media, solvents, conditions, as well as the drying and concentrating techniques used to prepare the extract for GC analysis. This presentation will use real-world experiences to provide practical information that can streamline SPE operations in the laboratory and lead to optimal recoveries for many EPA methods.

*Keywords:* environmental, optimization, solid phase extraction
*Application code:* environmental
*Methodology code:* sampling and sample preparation

### LONG-TERM STORAGE OF AIR-SAMPLED MERCURY ON GOLD-COATED QUARTZ TUBES

***David J. Hassett*** **and Erick J. Zacher**

*UND Energy & Environmental Research Center, 15 North 23rd Street, PO Box 9018, Grand Forks, ND 58203, USA*

The properties of mercury and mercury compounds have made the storage of low-level mercury samples a problem. The Energy & Environmental Research Center has developed a novel method of storage for mercury on gold-coated quartz traps. Samples are taken in the usual manner by drawing air or other gases through a quartz tube containing gold-coated quartz granules. The tubes are then placed into a bottle that has been lined with a gold-plated copper screen. A cap with a Teflon liner is then placed on the bottle. This provides a mercury-free environment for the traps and allows them to be shipped to or from a laboratory. Gold-coated quartz traps stored in bottles lined with gold-plated copper screen did not show appreciable accumulation of mercury for up to 2 months. Unlined covered and open storage containers were also evaluated and showed sorption of mercury from the surrounding environment. Mercury vapor samples stored on gold-coated traps should be analyzed as soon as possible. However, the above method shows that the mercury is stable, allowing ample time to transport the samples for analysis in the laboratory rather than immediately testing the samples in the field. This abstract was prepared with the support of the US Department of Energy (DOE) through the Coal Ash Resources Research Consortium® (CARRCSM).

*Keywords:* atomic spectroscopy, fluorescence, mercury, method development
*Application code:* general interest
*Methodology code:* sampling and sample preparation

### THE DEVELOPMENT OF AUTOMATED EQUIPMENT FOR DRYING AND CONCENTRATING ENVIRONMENTAL EXTRACTS

***Stephen J. MacDonald*** **and Robert S. Johnson**

*Horizon Technology, Inc, 8 Commerce Drive, Atkinson, NH 03079, USA*

Two steps that have a major impact on the recoveries for both liquid-liquid (LLE) and solid phase (SPE) extraction techniques are the drying step and the concentrating step. Residual water must be removed to prevent the extract from separating into multiple phases and back extraction of water soluble analytes. The extract must also be concentrated to improve detection limits by selectively evaporating the extraction solvent. Drying extracts has historically been accomplished manually with sodium sulfate. Recently, hydrophobic membranes have become available that permit automated removal of residual water. Further, this step can be incorporated into equipment that selectively evaporates the extraction solvent to completely automate sample drying and concentration for GC analysis. The use of newly developed equipment for drying and concentrating environmental extracts for both LLE and SPE will be discussed. Emphasis will be placed on analyte recovery, carryover, and sample throughput.

*Keywords:* environmental, instrumentation, sample preparation, solid phase extraction
*Application code:* environmental
*Methodology code:* sampling and sample preparation

### DETERMINATION OF CALIBRATION CONSTANTS OF PERMEATION PASSIVE SAMPLERS BASED ON THE LINEAR TEMPERATURE-PROGRAMMED RETENTION INDEX OF THE ANALYTES

***Suresh Seethapathy,*** **Tadeusz Górecki, Jacek Namiesnik, and Bozena Zabiegala**

*University of Waterloo, 200 University Avenue West, Waterloo, ON, Canada N2L 3G1*

Permeation-type passive samplers utilize the principle of permeation of gases or vapours through a polymer membrane which controls the analyte uptake into the sampler. The conventional method of calibration of passive samplers involves the generation of a standard gas mixture of all the analytes of interest, and exposure of the passive samplers to this mixture for a predetermined period of time. This requirement for experimentally determining the calibration constant for each individual analyte is time-consuming, and is one of the major disadvantages of the permeation passive sampling technology. This disadvantage could be overcome by establishing relationships between the physicochemical properties of the analytes and their calibration constants. In the case of permeation-type passive samplers utilising membranes made of thin films of polydimethylsiloxane (PDMS), the calibration constants of the analytes can be estimated from their linear temperature-programmed retention indices (LTPRI) on PDMS-coated capillary columns. This correlation is possible as the permeability of PDMS films towards various analytes is determined largely by the solubility of the analytes in the PDMS film and depends very weakly on their diffusion coefficients in this material. Since the retention index of a substance is also a function of the solubility of the analyte in the coating inside the capillary column, there should be a correlation between LTPRI and the calibration constant of the sampler towards this analyte. This correlation also makes it possible to estimate the calibration constants for unidentified analytes, which otherwise is not possible with conventional procedures. The excellent permeability properties of PDMS and the possibility of estimating the calibration constants of the analytes based on their physico-chemical properties make this type of sampler very attractive for practical use in the environmental monitoring field. The results of research into calibration of permeation passive samplers for various thicknesses of the PDMS membrane will be presented. Flux rates of various analytes in PDMS membranes reported in the literature will be correlated with their LTPRI to further analyze the permeability—LTPRI correlation.

*Keywords:* air, environmental analysis, sampling, volatile organic compounds
*Application code:* environmental
*Methodology code:* sampling and sample preparation

### METHOD DEVELOPMENT FOR THE SAMPLING AND ANALYSIS OF BLOOD FROM SEA TURTLES AND MARINE MAMMALS FOR ORGANOHALOGEN COMPOUNDS

***John Kucklick,*** **Paul Becker, Steven J. Christopher, Jocelyn Flanary, Jennifer Keller, Rebecca S. Pugh, Michele M. Schantz, Robert Swarthout, Stacy Vander Pol, and Stephen A. Wise**

*Hollings Marine Laboratory, National Institute of Standards and Technology, 331 Fort Johnson Road, Charleston, SC 29412, USA*

Monitoring protected marine species, such as sea turtles and bottlenose dolphins, for organohalogen contamination requires the use of nonlethal sampling techniques. The use of blood as a monitoring tool has collection advantages over other samples, such as biopsies, as blood collection is less invasive, can provide biomarker and health data, is fairly easily obtained from live-captured animals, and samples can be archived for future analysis. The National Institute of Standards and Technology in conjunction with the National Oceanic and Atmospheric Administration, Mote Marine Laboratory, and the State of South Carolina has developed standard protocols for collecting sea turtle and bottlenose dolphin whole blood and plasma samples. The protocol covers aspects of sample handling, including collection, storage, sample metadata, and field blanks. The protocol is currently employed in all wild bottlenose dolphin health assessments performed in the US Methods for the analysis of polychlorinated biphenyl congeners and organochlorine pesticides have been developed for whole blood and serum. The method utilizes liquid/liquid extraction followed by solid phase extraction (SPE) and size exclusion chromatography (SEC) cleanup steps. Analysis has been performed using dual column GC-ECD. Methods are currently being developed to extract samples using automated polymeric SPE, automated SPE cleanup, and semiautomated semipreparative SEC, followed by analysis using GC-MS with large-volume injections.

*Keywords:* environmental/biological samples, mass spectrometry, solid phase extraction, trace analysis
*Application code:* environmental
*Methodology code:* gas chromatography/mass spectrometry

### IDENTIFICATION AND QUANTIFICATION OF CARBOHYDRATES IN GLYCOPROTEINS OF TRANSGENIC CORN BY HIGH PERFORMANCE LIQUID CHROMATOGRAPHYFLUORESCENCE DETECTION AND LIQUID CHROMATOGRAPHY-SONIC SPRAY IONIZATION MASS SPECTROMETRY

***Tongwen Wang,* Yinfa Ma, and Paul K. Nam***

**Chemistry Department, University of Missouri-Rolla, 345 Schrenk Hall, Rolla, MO 65409-0010, USA*

Glycoproteins have drawn increasing attention in fields of biotechnology, clinical chemistry, pharmaceutical, and others. Transgenic corn offers a cost-effective means for large-scale production of therapeutic glycoproteins suitable for pharmaceutical purposes. In this study, both high performance liquid chromatography-fluorescence detection (HPLC-fluorescence) and liquid chromatography-sonic spray ionization mass spectrometry (LC-SSI MS) were used for characterization of carbohydrates in glycoproteins derived from transgenic corn. Compositional carbohydrates released by enzymatic and acid digestion of glycoproteins were chemical derivatized for HPLC-fluorescence analysis. Identification of heterogeneous monosaccarides present in the transgenic corn glycoproteins was confirmed by LC-SSI MS. The detailed experimental conditions and results obtained from a new approach for glycoprotein characterization will be discussed.

*Keywords:* carbohydrates, liquid chromatography, liquid chromatography/mass spectroscopy
*Application code:* bioanalytical
*Methodology code:* liquid chromatography/mass spectrometry

### PREPARATIVE LIQUID CHROMATOGRAPHY: THE REVERSE AUTOCOMPRESS COLUMNS OVERLOADING CONDITIONS

***François Dardoize* and Gilles Clodic***

**UPMC, Laboratoire Li2C, Universite P&M Curie 4, Place Jussieu, Boîte 51, Paris 75005, France*

Preparative chromatography is now a common tool in research laboratory as well as in production plants. A few years ago, the reverse auto-compression system was presented. This new system was quickly manufactured and distributed worldwide, the efficiency and the ease of use have contributed to this success. Two factors are important for the efficiency of a preparative column: the homogeneity of the packing of the material and the distribution of the sample. These two factors were studied and a solution is proposed to decrease their effects: the annular distributor system. The improvement of the efficiency brought to the system is demonstrated. Moreover, the main goal of preparative chromatography is to produce the maximum of pure product, and the best way for this task is to work under overloading conditions. Several important papers have explained the shape of the peaks under these conditions when conventional columns are used. These tests are repeated with a RACS column fitted with the ADS distributor. The results compared with those of the publications demonstrate the efficiency of the new system.

*Keywords:* HPLC columns, prep chromatography
*Application code:* process analytical chemistry
*Methodology code:* liquid chromatography

### EFFICIENT CHROMATOGRAPHIC PURIFICATION OF COMBINATORIAL LIBRARIES WITH NEW SOFTWARE ALGORITHMS FOR AUTOMATED PREANALYSIS, SAMPLE CULLING, PURIFICATION, AND FRACTION ANALYSIS

***Wulff Niedner,* Frank Arnold, and Thomas Piecha***

*Dionex Corporation, Dionex Softron GmbH, Dornierstrasse 4, Germering 82110, Germany*

Chromatographic purification of combinatorial libraries is a bottleneck in pharmaceutical discovery. Many laboratories purify all products obtained from parallel syntheses, even when only 50% to 70% contain the desired compound in a high enough yield. They accept the additional cost and time loss for purification of useless samples, because analytical preanalysis with subsequent culling of samples is time-consuming. Available software solutions for automation of this process have so far not been flexible and sophisticated enough to allow intelligent culling of samples based on user-definable criteria. This presentation shows a novel software solution that overcomes these limitations and allows complete automation of the purification workflow. Users can set up their system to inject samples into an analytical HPLC, select the samples worth purifying, inject them into a preparative HPLC, and reanalyze collected fractions. For automated culling of unwanted samples, they can define criteria based on the UV, MS, and ELSD signals of the analytical pre-injections. These new features allow accelerating the process and reducing the cost of chromatographic purification.

*Keywords:* automation, combinatorial chemistry, drug discovery, prep chromatograph
*Application code:* drug discovery
*Methodology code:* liquid chromatography

### LIQUID CHROMATOGRAPHY-MASS SPECTROMETRY AS A TOOL FOR CLASSIFICATION AND IDENTIFICATION OF BACTERIA USING AMINO ACID SEQUENCES OF TRYPTIC PEPTIDES DERIVED FROM CELLULAR PROTEINS

***Jacek P. Dworzanski,* Rui Chen, Samir V. Deshpande, Rhabih E. Jabbour, Liang Li, A. Peter Snyder, and Charles H. Wick***

*[*]Geo-Centers, Inc, PO Box 68, Aberdeen Proving Ground, MD 21010-0068, USA*

The ability to classify and identify bacterial pathogens is of paramount importance in many areas of public health. The growing number of fully sequenced bacterial genomes, combined with spectacular progress made in automated sequencing of peptides obtained from gene products, that is, proteins, opens new possibilities for exploring bacterial genomes for classification and identification purposes. We present a mass spectrometric approach to link these genomic resources with amino acid sequence information of proteins extracted from analyzed bacterial cells. The results of searching tandem mass spectra of peptide ions against a comprehensive bacterial proteome database are analyzed using probability-based scoring. This is followed by the automated taxonomic classification and potential identification of bacteria with an algorithm that uses phylogenetic relationships between bacterial species represented in the database as a part of a hierarchical decision tree process. Tryptic peptides derived from proteins extracted from lysed bacterial cells were analyzed with reversed phase liquid chromatography coupled with tandem mass spectrometry (quadrupole ion trap, LCQ Deca). Product ion mass spectra were searched with SEQUEST against a comprehensive bacterial protein database assembled using amino acid sequences translated from protein coding open reading frames of all bacteria with fully sequenced genomes. Matches between potential peptide sequences and bacterial proteomes were analyzed using discriminant analysis, and assignments were accepted or rejected based on probability criteria. An in-house developed bacteria identification algorithm processed the accepted matches. This algorithm removes degenerate peptide sequences and uses phylogenetic relationships among database organisms for taxonomic classification and identification of analyzed sample.

*Keywords:* bioanalytical, bioinformatics, liquid chromatography/mass spectroscopy, peptides
*Application code:* bioanalytical
*Methodology code:* liquid chromatography/mass spectrometry

### RAPID ANALYSIS OF PEMOLINE IN EQUINE PLASMA BY LIQUID CHROMATOGRAPHYTANDEM MASS SPECTROMETRY

***Cornelius E. Uboh***

*West Chester Pennsylvania Equine Toxic and Research Center, University of Pennsylvania, 220 East Rosedale Avenue, West Chester, PA 19382, USA*

A rapid and sensitive method for high throughput analysis of pemoline in equine plasma by LC/TSQ-MS/MS is described. Analysis was performed by positive electrospray ionization. Extraction efficiency of various solvents was determined. Ethyl acetate (EtoAc) was the best solvent for extraction and cleanliness of the extract. Plasma (0.5 mL) was augmented with pemoline (0.1–100 ng/mL). Clonixin served as the internal standard. Sample was mixed with 1 mL of 0.1 M phosphate buffer (pH 5.0), and extracted by 5 mL EtoAc for 10 min and centrifuged (3000 $\times$ g $\times$ 10 min). Extract was concentrated at 60°C and reconstituted in 100 $\mu$L mobile phase (2 mM ammonium acetate, pH5.0:acetonitrile; 50 : 50, v/v), and 10 $\mu$L was used for analysis. Entire analysis lasts 4 minutes. Retention time for pemoline was 1.77 $\pm$ 0.10 min and 1.89$\pm$0.10 min for clonixin (IS). Product ion (m/z 106.0) was chosen as the target ion for pemoline screening whereas that for clonixin was m/z 245. Confirmation of pemoline in plasma was achieved by data dependent scan based on parent ion. Recovery of pemoline from plasma was 93.67% to 105.01% with CV of 9.13% to 6.29%. Intraday accuracy and precision at 0.5 ng/mL, 5 ng/mL and 50 ng/mL were 100.0%–100.6% and 2.40%–0.96%, respectively, whereas those for interday were 102.0%–101.5% and 1.72%–0.92%, respectively. LOD with LOQ was 100 pg/mL (S/N = 5). Quantification was linear at 0.1–100 ng/mL (r2 > 0.995). LOC was 0.5 ng/mL. The method is fast, simple, sensitive, and reliably reproducible.

*Keywords:* bioanalytical, drugs, liquid chromatography, tandem mass spectrometry
*Application code:* bioanalytical
*Methodology code:* liquid chromatography/mass spectrometry

### FAST MAPPING OF GUNSHOT RESIDUES ON CLOTH AND SKIN TO DETERMINE SHOT DISTANCE

***Dirk Wissmann***

*Spectro Analytical Instruments GmbH & Co. KG, Boschstrasse 10, Kleve 47533, Germany*

The determination of a shot distance is one of the time-consuming tasks in a forensics science laboratory. Typically the cloth or skin sample is treated with special chemicals to make the residue visible. By this a pattern is created, which is compared to samples, which are prepared in the forensics lab. Those samples are created with comparable ammunition and cloth or skin from different shot distances. From this comparison an experienced scientist estimates the shot distance. Modern EDXRF with mapping facilities can shorten the time of the investigation dramatically. As the sample is not modified or destroyed by the analytical method, it still can be investigated by other means later. The task of this investigation is to do the analysis in a very sensitive way, but also in a short period of time, as organic samples as skin will change their structure with time in the ambient conditions of an analytical lab. The final task has to be to analyze one real sample and up to 5 comparison samples per working day. In addition the analysis does not only determine the shot distance but also includes the characterization on the ammunition used on base of some typical elements in the gunshot residue.

*Keywords:* elemental analysis, forensic, X-ray fluorescence
*Application code:* homeland security/forensics
*Methodology code:* X-ray techniques

### A NEAR-REAL-TIME AIR MONITORING SYSTEM FOR CHEMICAL WARFARE AGENTS

***James H. Crabtree* and Bruce D. Quimby***

**Agilent Technologies, Inc., 2125 East Katella Avenue, Suite 300, Anaheim, CA 92806-6074, USA*

One of the analytical needs generated by the recent focus on homeland security is the ability to monitor for the presence of airborne chemical warfare agents (CWAs). Of particular concern are the nerve agents and mustard (HD). Due to their extreme toxicity, monitoring systems must reliably detect the agents at very low concentrations. The short-term exposure limits (STELs) for chemical demilitarization projects in the US, for example, range from 0.9 ppt for VX to 449 ppt for HD. The challenge is to detect the agents reliably at or near these levels while minimizing false positives and false negatives. This talk will describe a GC system configured to monitor for all of the significant V- and G-type nerve agents and HD from existing global stockpiles at STELs in 10-minutes NRT. The system consists of an air concentrator/thermal desorption sampling system connected to a gas chromatograph with two columns coated with different stationary phases and both connected to flame photometric detectors (FPDs). An air sample (usually several liters) is drawn through a sorbent trap to concentrate any CWAs. The CWAs are thermally desorbed from the trap and injected onto both columns, where they are selectively detected by the FPDs. The presence of an agent is confirmed by a peaks at the correct retention times on both columns. This minimizes false positives. The talk will describe system performance in stockpile monitoring and chemical demilitarization operations and discuss critical issues for extension into homeland security applications, such as calibration and passivation.

*Keywords:* detection, gas chromatography, monitoring, thermal desorption
*Application code:* homeland security/forensics
*Methodology code:* gas chromatography

### NEW TECHNOLOGY IN DISCRETE ANALYSIS FOR THE AUTOMATED TESTING OF INORGANIC IONS IN WATERS

***Ninglan Liao,* Michael L. Duffy, and William Lipps***

**O. I. Analytical, PO Box 9010, College Station, TX 77845, USA*

Automated testing of inorganic ions (as well as other commonly tested compounds, such as phenols) in waters by discrete analyzers is rapidly becoming an alternative technology for environmental testing laboratories due mainly to ease of use. The first generation of discrete analyzers for environmental use has been modified from clinical discrete analyzers. Clinical analyzers are typically aimed at the analysis of very high concentration levels of compounds of interest, such as 200 ppm phosphorous in human blood. With such high levels of analyte in these samples, all in a clearly defined matrix, the low-level sensitivity and issues with interfering matrix effects on the analysis, was not a key issue for designing a reliable system. However, for environmental samples, the system needs to be capable of testing down to ppb and sub-ppb levels in rather complex, and changing, sample matrices, as has been achieved by continuous flow analyzers for the past 20 years. It is extremely challenging for existing discrete analyzers to measure low-level samples to meet EPA regulatory requirements due to limited sensitivity and matrix effects. This presentation will discuss the current limitations for existing discrete analyzers to achieve lower detection limits and higher precision, review how interferences affect the sample analysis, and how a new discrete analyzer by O.I. Analytical has combined new technology developed to allow users to overcome interferences for more accurate results on environmental samples. Additional features focused on achieving higher sensitivity and precision per EPA methods will be discussed. Performance data will be presented.

*Keywords:* automation, environmental analysis, instrumentation, wet chemical methods
*Application code:* environmental
*Methodology code:* chemical methods

### ADVANCEMENTS IN LOW-COST DISCRETE ANALYSIS OF INORGANIC IONS IN WATERS

***David Riese***

*Westco Scientific Instruments, 12 Precision Road, Danbury, CT 06810, USA*

Westco Scientific Instruments will present extensive data on a new bench-top discrete analyzer designed to meet the low-cost needs of today's smaller environmental laboratories.

Until now, other low-cost discrete analyzers have used a flow cell to measure the final reaction product. The flow cell design, while inexpensive to implement, brings along technical issues inherent to full-scale flow analyzers; potential for carryover and complicated troubleshooting due to a sophisticated flow system. The new Westco system features true second-generation technology, typically only available in higher-cost discrete analyzers. The second-generation design provides individual cuvettes for each individual reaction product. This design minimizes any possibility of sample carryover, as each sample is handled in a truly discrete manner. An optional Intelligent wash station washes, rinses, and checks each cuvette for cleanliness to ensure carryover-free operation. An alternative to flow analysis, discrete analysis moves closer to “keyboard chemistry,” eliminating the need to manually change manifolds, filters, and tubing between analytical methods. EPA-approved methods are pre-programmed and ready for use in today’s environmental laboratory.

*Keywords:* automation, environmental analysis, environmental/waste/sludge, environmental/water
*Application code:* environmental
*Methodology code:* other

### AUTOMATED MERCURY ANALYSIS: INCLUDING DIGESTION

***Jason P. Gray,* Alvin Chua, and Koji Tanida***

**AGS Scientific, Inc, 1511 Texas Avenue South, Suite 270, College Station, TX 77840, USA*

Mercury (Hg) is one of the most highly monitored environmental pollutants in the world today. Inexhaustible world population growth and decreasing water supplies are factors that continue to drive the need for reduction of industrial pollution. With such high demand for monitoring mercury contamination levels, automation of mercury analysis methods is of increasing importance for industrial compliance laboratories, government regulatory laboratories, and environmental contract laboratories. One of the more troubling steps required in many of the traditional analytical methods is the sample preparation step, which requires acidic digestion of the samples prior to analysis. Such sample preparation is labor intensive, consumes valuable time, and can be hazardous due to the handling of concentrated acids at high temperatures. Many automated mercury analyzers are available today, however few, if any, have been able to fully automate methods such as EPA 245.1, 245.2, 245.7, and 1631E from digestion through analysis. In this presentation, a new mercury analysis system that is capable of total automation of such methods, including the digestion and reagent addition will be described. This mercury system functions on the principles of reducing vaporization, optional gold amalgamation, and cold vapor atomic absorption spectroscopy (CVAAS). Illustrations and supporting data will be provided.

*Keywords:* atomic spectroscopy, environmental analysis, mercury, trace analysis
*Application code:* environmental
*Methodology code:* atomic spectroscopy/elemental analysis

### MONITORING THE IN VITRO ADVANCED GLYCATION ENDPRODUCT (AGE) FORMATION OF GLYOXYLATE WITH LYSINE, ARGININE, AND GLUCOSAMINE USING CAPILLARY ELECTROPHORESIS

***Udayan Dutta* and Joel A. Dain***

**University of Rhode Island, 51 Lower College Road, Kingston, RI 02881, USA*

Glucosamine (GlcN) is an unregulated over the counter dietary supplement that has been reported in many clinical studies to be highly effective in the treatment of osteoarthritis with few side-effects. In addition to forming autocondensation products, GlcN like other reducing sugars and their metabolites reacts nonenzymatically with free amino acids and proteins to form a complex and heterogeneous group of compounds commonly referred to as either advanced glycation endproducts (AGEs) or maillard reaction products. Glyoxal (GO) is a carbonyl intermediate of glucose autooxidation under physiological conditions that is found in AGE pathways involving glucose as the reducing sugar. GO is also formed as a byproduct of lipid peroxidation and glycine metabolism and acts as a major contributor to AGE formation in diabetes. Lysine (Lys) and arginine (Arg) are essential amino acids and important nutritional supplements, which undergo glycation and form various common AGE intermediates and derivatives. The objective of the present study is to develop a model system of GO as an AGE precursor by monitoring its reaction with the amino acids lysine and arginine and the amino sugar GlcN by the use of capillary electrophoresis (CE) for a potential study in diabetic patients. Lys and Arg undergo rapid glycation with GO and exhibit greater AGE formation with increasing concentration and time. GO rapidly forms AGE with GlcN and inhibits its autocondensation. Formation of AGE was monitored by UV and fluorescence spectroscopy. Comparison of the AGE profile for each reaction was performed by CE.

*Keywords:* amino acids, bioanalytical, capillary electrophoresis, carbohydrates
*Application code:* bioanalytical
*Methodology code:* capillary electrophoresis

### SEPARATION OF RECOMBINANT HUMAN ERYTHROPOIETIN GLYCOFORMS BY IONENE-COATED CAPILLARY ELECTROPHORESIS AND ON-LINE CAPILLARY ELECTROPHORESIS- ELECTROSPRAY IONIZATION-MASS SPECTROMETRY

***Huwei Liu* and Bing Yu***

**Department of Chemistry, Peking University, Beijing 100871, China*

A series of ionene polymer was synthesized and used to coat fused silica capillaries for the separation of recombinant human erythropoietin (rHuEPO) by capillary electrophoresis (CE). The influence of the charge density of coatings and the concentration and pH of separation buffer on the separation were investigated, demonstrating that when the capillary was permanently coated with 6,6-ionene and a buffer containing 300 mM of acetic acid-ammonium acetate (HAc-NH4Ac) at pH 4.8 was used, a significantly reproducible separation was achieved for rHuEPO glycoforms. Because the CE conditions used in this study were highly compatible with electrospray ionization mass spectrometry (ESI-MS) analysis, we employed the coupling technique of CE-ESI-MS to attain the online total ion current (TIC) signal of intact rHuEPO successfully and reproducibly. These results confirm the robustness of the methodology and the usefulness as a good alternative for analyzing and detecting intact rHuEPO.

*Keywords:* bioanalytical, capillary electrophoresis, mass spectrometry
*Application code:* bioanalytical
*Methodology code:* capillary electrophoresis

### MONITORING GABA IN THE RODENT BRAIN IN VIVO USING MICRODIALYSIS COUPLED TO CE-LIF

***Kristin N. Schultz***

*University of Michigan, 930 North University Avenue, Ann Arbor, MI 48109, USA*

One way to study brain function is to correlate changes in neurotransmitter levels (both naturally and chemically induced) with behavior, pharmacological manipulation, and physiological state. In such measurements temporal resolution can be important to achieving good correlations because neurochemical concentrations fluctuate rapidly. Coupling microdialysis online to capillary electrophoresis is a method that can measure changes in neurotransmitter levels every 12 seconds. In this approach, sample collected from the extracellular space of the rat brain is derivatized with ortho-phthaldialdehyde (OPA) and 2-mercaptoethanol, which form a fluorescent product with primary amines. A flow gate injects the sample onto the column, where sample components are separated under the influence of an electric field. A sheath-flow cuvette minimizes background fluorescence when the components are detected through laser-induced fluorescence. In this work, we focus on the measurement of gamma-aminobutryic acid (GABA), the main inhibitory neurotransmitter in the brain. In one project, the effect of estradiol on GABA levels is examined. Studies show that estradiol decreases GABA levels in dialysate. This result supports the hypothesis that estrogen enhances dopamine function, and therefore reinforcement of addictive substances such as cocaine, by inhibiting GABA release, which in turn disinhibits dopamine release. These results illustrate the potential of CE for chemical monitoring applications.

*Keywords:* amino acids, bioanalytical, capillary electrophoresis, drugs
*Application code:* neurochemistry
*Methodology code:* capillary electrophoresis

### UTILITY OF MIXED MICELLES FOR SIMULTANEOUS CHIRAL SEPARATION OF STRUCTURALLY SIMILAR COMPOUNDS IN MICELLAR ELECTROKINETIC CHROMATOGRAPHY: AN APPROACH TOWARDS HIGH-THROUGHPUT CHIRAL ANALYSIS

***Sheron Franklin Tate,* Rashid Iqbal, Syed A. Rizvi, and Shahab A. Shamsi***

**Center of Biotechnoogy and Drug Design Department of Chemistry, Georgia State University, Atlanta, GA 30303, USA*

In general, it is unlikely that more than one chiral drug is prescribed for one disease. However, simultaneous separation of structurally similar chiral compounds has several advantages. First, the combination of various mixed micelles can simply be used to simultaneously analyze these chiral compounds for which various chiral assay methods with different conditions are listed in US Pharmacopoeia. Hence, a single separation condition using mixed micelles is sufficient to assay all of structurally similar drugs in a single run. Second, the use of mixed micelles can be used to simultaneously analyze chiral drugs and their primary and/or secondary metabolites in biological samples. Third, physicochemical properties such as log P can be determined in a high-throughput fashion for combinatorial mixtures of chiral drugs. Thus, the use of mixed chiral micelles for simultaneous chiral and achiral separation offers unique possibility in micellar electrokinetic chromatography (MEKC). In this study, enantiomeric resolution of several classes of structurally similar chiral compounds is first compared using leucine, valine, and leucine-valine derivatives of polymeric alkenoxy and polymeric acyl amino acid and dipeptide surfactants. Next, various combinations of amino acid and dipeptide derivatives of these polymeric surfactants are prepared at various molar concentration ratios before and after polymerization to evaluate the enhancement of enantioselecitivity. Various classes of structurally similar chiral drugs such as binaphthyl derivatives, benzoin derivatives, phenylethylamines, benzodiazepines, PTHamino acids, and b-blockers are compared in a single MEKC run using various mixed micelles. Physicochemical properties such as partial specific volume, aggregation number, and polarity of these mixed micelles are planned to fully understand the utility of these mixed micelles for chiral separations in MEKC.

*Keywords:* capillary electrophoresis, chiral separations, drugs
*Application code:* pharmaceutical
*Methodology code:* capillary electrophoresis

### EVALUATION OF COATINGS AND ALLOYS TO EXTEND THE LIFETIME OF EQUIPMENT USED IN CORROSIVE ENVIRONMENTS

***Gary A. Barone*** * ***and David A. Smith***

*Restek Corporation, 110 Benner Circle, Bellefonte, PA 16823, USA*

Manufacturing control systems and components are most often exposed to environments, process streams, and/or analytical streams that cause corrosive wear. Industrial corrosion is responsible for several billion dollars of additional costs related to periodic maintenance, process inefficiencies, process downtimes, and hazardous environmental and human health impacts. Chemical corrosion of system components has many potential solutions, including unique corrosive-resistant substrates (e.g., stainless steels, high-performance alloys, plastics, PTFE). Another corrective approach involves electrochemical protection through sacrificial materials. Yet, another method for the prevention of corrosion is the application of a protective coating. Each of these methods has benefits and drawbacks. The most desirable characteristics of a solution to corrosive wear are low cost, ease of use/application, and ultimate performance during use. Value-add is the key where the cost of the solution pays dividends in system performance, decreased downtime and decreased periodic maintenance. A variety of substrates and comparative corrosion performances under ASTM and NACE-based testing protocols will be presented. Stainless steels, coated stainless steels and high-performance alloys will be evaluated and compared through performance and costing to better understand the value of each potential solution.

*Keywords:* fuels energy petrochemical, monitoring, process control, wet chemical methods
*Application code:* fuels, energy and petrochemical
*Methodology code:* physical measurements

### SIMPLIFYING THE ANALYSIS OF PERMANENT GASES, LIGHT HYDROCARBONS, AND A VARIETY OF OTHER GASES USING A UNIQUE MICROPACKED COLUMN

***Barry L. Burger***

*Restek Corporation, 110 Benner Circle, Bellefonte, PA 16823, USA*

The ability to separate light gases requires a high surface area solid support medium in excess of 1000 $m^2$/gm. A new type of carbon molecular sieve gas-solid chromatographic (GSC) support medium with a surface area of ~ 1500 $m^2$/gm is ideally suited for the analysis of light gases. This material is robust, thermally stable at 330° C, and compatible with sensitive detectors such as the helium ionization detector (HID). Data will be presented illustrating the separation of light hydrocarbons, permanent gases, permanent gas mixtures, and also a variety of other gases on this new material.

*Keywords:* chromatography, fuels energy petrochemical, petrochemical, specialty gas analysis
*Application code:* fuels, energy and petrochemical
*Methodology code:* gas chromatography

### ANALYSIS OF SATURATED PARAFFINS IN PETROLEUM BY FAST GAS CHROMATOGRAPHY

***Lei Cao,*** * ***Yuki Hashi, Ying Ming Li, and Jie Wu***

*Shimadzu Hong Kong Ltd, 14th Floor, China Life Tower, No 16 Chaoyangmenwai Street, Beijing 100020, China*

A fast capillary gas chromatographic method was described for the analysis of saturated paraffins in crude oil and rock extracts. By using a 20 m narrow-bore capillary column, the analytical time has been shortened to 15 minutes, 5 times faster in comparison with 80 to 90 minutes by conventional capillary gas chromatography, which resulted in higher throughput, higher efficiency, and better separation. The fast method is fully in compliance with petroleum industrial standard SY/T5120-1997 and paves the way for the fast analysis of paraffin isomers, such as steroidal paraffins and terpanes by GCMS and that of aromatics by GC.

*Keywords:* analysis, gas chromatography, method development, petrochemical
*Application code:* fuels, energy and petrochemical
*Methodology code:* gas chromatography

### A NOVEL MICROFLUIDIC CHIP-ELECTROSPRAY MASS SPECTROMETRY INTERFACE FOR LOW ELECTROOSMOTIC FLOW SYSTEMS AND SENSITIVE, POSITIVE ION MODE MASS SPECTROMETRY DETECTION OF HIGH pH TRYPTIC DIGESTS

***Trust T. Razunguzwa*** * ***and Aaron T. Timperman***

*Department of Chemistry, West Virginia University, 217 Calrk Hall, Prospect Street, Morgantown, WV 26506-6045, USA*

The purpose of this work is to develop a microfluidic-ESI-MS interface for low electroosmotic flow (EOF) systems, as well as pH reduction for high pH electrokinetic separation of tryptic digests. Low EOF systems are important because coatings such as polyethylene glycols used to minimize adsorption of proteins typically reduce EOF. The interface is designed to decouple the CE and ESI voltages, which allows for better control of the ESI voltage. This interface has been applied to the electrokinetic delivery of tryptic digests (pH = 8), with positive ion mode mass spectrometry detection. The separation and pH reduction of the tryptic digests, as well as the application of the electrospray voltage are all integrated onto the microfluidic device. High pH separation of tryptic digests can be performed upstream on the microfluidic device, and a low pH make-up solution is used to reduce the pH of the digest. The ESI voltage is applied to the reservoirs of two opposing channels intersecting the separation channel. The applied voltage is rapidly switched between these two reservoirs via a relay switch at frequencies up to 40 kHz. The performance of the interface has been characterized by the separation at high pH of a standard peptide mixture, as well as a tryptic digest of bovine serum albumin (BSA), in channels coated with polyethylene glycol (PEG) terminated self-assembled monolayers. The pH of the separated peptides is reduced prior to detection by ESI-MS. This work is funded by NIH/NCRR Center of Biomedical Research Excellence Grant.

*Keywords:* capillary electrophoresis, lab-on-a-chip/microfluidics, mass spectrometry.
*Application code:* proteomics and genomics
*Methodology code:* microfluidics/lab-on-a-chip

### DEVELOPMENT OF MINIATURIZED ELECTRIC FIELD GRADIENT FOCUSING FOR PROTEIN ANALYSIS

***Ryan T. Kelly,* Paul H. Humble, Milton L. Lee, and Adam T. Woolley***

**Department of Chemistry and Biochemistry, Brigham Young University, C-100 Bnsn, Provo, UT 84602, USA*

Proteomic analysis, which is becoming an increasingly important area of research for preclinical drug screening and early detection of diseases, currently relies on slow, labor-intensive separation techniques. Electric field gradient focusing (EFGF) uses a combination of pressure-driven flow and an electric field gradient to separate and focus on charged species according to their electrophoretic mobilities. EFGF has several advantages over standard protein analysis techniques, such as increased sample loading capacity and the ability to simultaneously separate and concentrate analytes. We have successfully developed and tested capillary-based and micromachined EFGF devices that use a semipermeable acrylic copolymer of changing cross-sectional area to establish an electric field gradient along a protein-focusing channel. With the capillary-based devices, standard mixtures of both natively fluorescent and fluorescently labeled proteins have been separated, and dilute samples have been enriched up to 10 000-fold. The microchip EFGF devices utilize a solvent bonding approach in their construction and have demonstrated increased resolution compared with capillary-based devices, due to reduced laminar flow-induced dispersion. Continued developments in this field, such as surface modifications to reduce electroosmotic flow, should help to make EFGF a valuable tool for proteomics research.

*Keywords:* fluorescence, protein
*Application code:* bioanalytical
*Methodology code:* microfluidics/lab-on-a-chip

### A MICROCHIP-BASED BLOOD BRAIN BARRIER MIMIC FOR MONITORING THE FATE OF ENDOTHELIUM-DERIVED NITRIC OXIDE

***Matthew K. Hulvey,* R. Scott Martin, and Dana M. Spence***

**Department of Chemistry, Saint Louis University, 3501 Laclede Avenue, Saint Louis, MO 63103, USA*

Previous work involving the study of transport across mimics of the blood brain barrier (BBB) has primarily involved cells cultured on permeable membranes placed in a diffusion cell. This static system fails to mimic the interface of the circulatory system at the BBB. Here, we will describe studies toward the creation of a microchip-based blood brain barrier mimic. This mimic will ultimately involve bovine brain microendothelial cells (BBMECs) coated on a polycarbonate membrane, which will separate two separate channel networks made of poly (dimethylsiloxane) (PDMS). The fabrication of silicon master molds using soft lithography methods and the incorporation of bilayer PDMS devices involving valves and flow channels for this mimic will be discussed. Fluorescence microscopy and amperometric detection were used to examine the ability of the valves to stop flow at the molecular level, with this being the first report of incorporating electrodes with on-chip valving. Secondly, we will discuss the culture of bovine pulmonary arterial endothelial cells (BPAECs) in the flow channels using fibronectin. BPAECs are initially being used in place of BBMECs for convenience purposes. It was found that the use of hydrodynamic flow to introduce cells into these devices severely hampered the immobilization of the BPAEC's. Steps towards using on-chip valving to direct the location of cell immobilization as well as to eliminate any secondary flow effects while the cells are attached to the fibronectin-coated surface will be discussed. Lastly, we will discuss the future work and ultimate goal of this project, which is to amperometricly detect endothelium-derived nitric oxide upon stimulation with adenosine triphosphate.

*Keywords:* bioanalytical, biological samples, lab-on-a-chip/microfluidics
*Application code:* bioanalytical
*Methodology code:* microfluidics/lab-on-a-chip

### TOWARDS MICROCHIP LIQUID CHROMATOGRAPHY USING ELECTROCHEMICAL MICROPUMPS

***Hernan V. Fuentes,* Jason W. Munyan, and Adam T. Woolley***

**Department of Chemistry and Biochemistry, Brigham Young University, C-100 Bnsn, Provo, UT 84602, USA*

In recent years, the integration and miniaturization of biochemical analysis onto a small platform, which is often called a micro-total analysis system ($\mu$-TAS) or lab-on-a-chip, has attracted much attention. Electrically driven separation methods particularly, have greatly benefited from the use of microfabrication technologies. However, miniaturization of other analytical techniques such as liquid chromatography although highly desired have been hindered mainly by difficulties in making the pressure connection onto a microchip and the introduction of the stationary phase into the microchannel. Here, we describe the fabrication of electrically actuated micropumps for microfluidic applications. Micropumps have been fabricated in both poly-(dimethylsiloxane) and poly-(methylmethacrylate) (PMMA) substrates. Micropumps were evaluated by measuring the flow rate, based on the time to transfer 5 $\mu$L of water from one reservoir through a microfabricated channel to an exit reservoir. We will present different micropump designs and approaches that have been evaluated. Pumping rates of tens of $\mu$L/min have been achieved against back pressures as high as 150 psi using an integrated PMMA micropump microchannel. Also, preliminary experiments using two micropumps in a PMMA substrate were performed to evaluate pressuregated injection of samples onto a microchannel. Recently, we have applied conventional photolithography and wet-chemical etching to fabricate micropumps integrated with microchannels in a single glass substrate. Chemical modification of the channel walls should allow us to perform chromatographic separations in these devices. Our results demonstrate that electrochemical micropumps hold great potential to generate pressure-driven flow in microchannels, and thus may be applicable for miniaturization of liquid chromatography.

*Keywords:* lab-on-a-chip/microfluidics
*Application code:* high-throughput chemical analysis
*Methodology code:* microfluidics/lab-on-a-chip

### CHEMISTRY ON A MICROCHIP: FLUORESCENCE DERIVATIZATION OF FATTY AMINES

***Kristin M. Adams,* Tara S. Carpenter, and Mitchell E. Johnson***

**Department of Chemistry and Biochemistry, Duquesne University, 600 Forbes Avenue, Pittsburgh, PA 15282, USA*

There are many significant compounds whose resting levels in biological systems are at nanomolar concentrations or below. At these low concentrations, detection is problematic for many current methods of analysis. Derivatization of the analyte can improve detection limits, but solution derivatization is lengthy and can produce interfering byproducts at these concentrations. In previous work, phenylalkylamines were reacted with 5-fluorescein isothiocynate (FITC) online using a C18 chromatographic stationary phase. Preconcentration in the stationary phase helps improve the ability to react amines with fluorescent dye. However, the previous method, while offering sub-micromolar detection limits, required relatively high mass loads (nanomoles). By utilizing a microchip packed with a solid phase, we can use this technique to perform derivatization reactions at even lower detection limits and with much lower mass loads. The use of mass spectrometry provides a means to detect these low concentrations. Results will be given for the derivatization of fatty amines with FITC on a microchip packed with a solid phase allowing for picomole detection. Experimental results and procedures including injection technique, reaction, elution, and detection conditions will be given. This work is supported by NIH/NINDS R15NS038443.

*Keywords:* derivatization, lipids, online
*Application code:* bioanalytical
*Methodology code:* microfluidics/lab-on-a-chip

### A MINIATURIZED FIA ANALYZER FOR MONITORING TOTAL TRIHALOMETHANES AND TOTAL HALOACETIC ACIDS IN DRINKING WATER

***Gija Geme,* James J. Aschberger, Gary L. Emmert, and Lucy J. Thurston***

**Department of Chemistry, The University of Memphis, Smith Chemistry Building, Room 213, Memphis, TN 38152, USA*

The emerging techniques of microfabrication and microfluidics, though broader in scope, are in many ways analogous to the more traditional field of flow injection analysis (FIA). Today, many researchers use a variety of microfabrication techniques to prepare specialized microconduits for many miniaturized applications. As with FIA, once these microfabricated structures are prepared, many are used for fundamental and practical studies that depend or relate to fluid flow in small channels microfluidics. Current research is aimed at the miniaturization of FIA (micro-FIA). Recently, a new method using nicotinamide- (NCA)-fluorescence-FIA is being developed to detect total trihalomethanes (THMs) and total haloacetic acids (HAAs) in drinking water. The method is based on a Fujiwara-like reaction between the THMs or the HAAs and NCA in the presence of base (0.2 M NaOH) that results in a fluorescent product. The goal is to miniaturize this method. A miniaturized FIA manifold has been constructed on a single chip. A single-syringe pump is used for sample injection and to drive the fluid inside the channels. This is done by connecting the syringe pump at the end of the conduit line. The aspiration of the syringe creates vacuum inside the channels to flow the sample and reagents toward the end of the conduit line and then passes through the detector flow cell. In general, micromechanical pumping devices such as silicon or plastic diaphragm pumps and electroosmotic flow are two important and basic methods of the fluid movement in microfluidic systems. However, the fluid movement on this proposed system depends upon the negative pressure coming from the one single-syringe pump. An off-chip "serpentine-type" mixing coil is employed for heating the reaction between NCA and the THM or HAA species. Two types of detectors will be investigated—a Waters fluorescence detector with a lab-built PDMS flow cell and also a miniaturized stand-alone LED-fluorescence detector. The optimized analyzer is expected to be about the size of a typical shoebox and would be capable of monitoring both total THMs and total HAAs in drinking water. Additionally, a laptop computer and a dual-channel data collection box would be incorporated into the design.

*Keywords:* environmental/water, flow injection analysis, fluorescence, lab-on-a-chip/microfluidics
*Application code:* environmental
*Methodology code:* microfluidics/lab-on-a-chip

### ON-LINE EVALUATION OF TRAINING NEEDS AND CUSTOMIZABLE MODULAR COURSES TO MEET THEM

***Christina S. Gilpin,*** [*] ***Roger K. Gilpin, and Joseph G. Solch***

[*] *Select-o-sep, LLC, P.O. Box 164 75841 Dry Ridge Road, Freeport, OH 43973, USA*

As chemical and information technologies continue to develop at an increasing rate, there is a growing industrial need for an effective way of evaluating their employees' skill levels and, if needed, providing training to improve detected weaknesses. Traditionally this has been through the uses of short courses and workshops held either in-house or off-site. Alternatively, with the advent of web training, each of these can be easily met via the use of an interactive, online testing platform to evaluate professional strengths and weaknesses in targeted areas and by the use of web-based learning modules that can be easily combined to form unique learning experiences/training courses. This presentation will demonstrate important aspects of setting up a user friendly environment to host the initial skill level evaluation, and the subsequent use of interactive tutorials that provide constant feedback in the learning process. Emphasis will be in areas of traditional and instrumental methods of chemical analysis and information management of student progress. Two types of courses will be used as examples, a beginning chemistry course and an advanced fundamentals of chromatographic analysis course.

*Keywords:* education
*Application code:* other
*Methodology code:* education/teaching

### DEVELOPMENT OF AN ON-LINE METHOD FOR THE DETERMINATION OF AMMONIUM BICARBONATE IN AN ADVANCED AQUEOUS AMMONIA-CO2 SCRUBBING SYSTEM

***Stuart C. Burris,*** [*] ***Holt Bui, Lingyu Meng, and Wei-Ping Pan***

[*] *Department of Chemistry, Western Kentucky University, 1 Big Red Way, Bowling Green, KY 42101, USA*

A suite of samples containing ammonium carbonate, ammonium bicarbonate (ABC), and ammonium carbamate were studied using near-IR spectrometry. The spectra were used to form a regression for the determination of ABC in the products from an advanced aqueous ammonia-CO2 scrubbing system. The suite contained three subsets of samples: one with bicarbonate and carbonate, one with bicarbonate and carbamate, and one with bicarbonate, carbonate, and carbamate, all in varying percentages by mass. The NIR spectra (1400–1550 nm only) for all samples were imported into a chemometrics software package for analysis. A principal components analysis (PCA) was completed to separate the three subsets of samples. The PCA indicated that samples with high percentages of ABC were grouped together and were not dependent on the remaining composition of the sample. However, as the percentage of carbonate or carbamate increased, the samples separated into two additional groups. The same spectra, along with the mass percentage data for the samples, were then used to perform a partial least-squares regression that employed three latent variables to explain 100% of the spectral and 98% of the constituent variation for bicarbonate. The predicted-versus-measured plot for cross-validation is almost identical to calibration, indicating a robust model (SEC = 4.1 and SEP = 4.7). Two test samples from the advanced aqueous ammonia-CO2 scrubbing system were analyzed using this regression. The results for the NIR method agreed closely with a separate determination of the ABC in the test samples. The RSD in the percentage of ABC for all analyses was less than 0.28%. An additional suite of 81 calibration samples containing only bicarbonate and carbonate was investigated to refine the method. This refined model was also quite robust (SEC = 3.8 and SEP = 4.1). It was used to predict the percentage of ABC in ten additional test samples from the scrubbing system, again giving close agreement with a separate determination of the ABC. The RSD in the percentage of ABC for the additional test samples ranged from 0.19% to 1.33%. The higher RSD and accompanying uncertainties in the data are primarily due to the larger particle size in the test samples compared to the calibration samples. In the online application, a large number of samples from the scrubbing system will be included in the calibration to compensate for this.

*Keywords:* detection, method development, near infrared, online
*Application code:* fuels, energy and petrochemical
*Methodology code:* near-infrared

### EXPANDING THE ROLE OF NIR IN IDENTIFICATION THROUGH ANALYSIS OF NEW MATERIALS AND SOFTWARE TOOLS

***Ronald L. Rubinovitz***

*Buchi Analytical, Inc, 19 Lukens Drive, Suite 400, New Castle, DE 19720, USA*

Although near-IR (NIR) has been quite successful as a raw material identification tool, particularly in the pharmaceutical industry, its application in this area has somewhat "stabilized." In this study, the potential for NIR spectroscopy to be used as an identification method for pharmaceutical raw materials of an inorganic nature is evaluated, since this class of materials is generally considered to be a poor candidate for NIR spectra. In addition, the effectiveness of identifying solids through the plastic container liner typically used in shipment, compared to measuring spectra of the solids directly, is compared. Being able to successfully identify even inorganic materials with weak spectral features through plastic liners has strong practical consequences for the use of NIR in the pharmaceutical industry. Finally, the increasing use of NIR as a "container comparison" tool to quickly reduce non-NIR testing is explored, through the use of new software tools available.

*Keywords:* near-infrared, pharmaceutical, quality control
*Application code:* pharmaceutical
*Methodology code:* near-infrared

### IN-SITU CHEMICAL ANALYSIS ON THE 2007 PHOENIX MARS SCOUT MISSION

***Samuel P. Kounaves***

*Department of Chemistry, Tufts University, 62 Talbot Avenue, Medford, MA 02155, USA*

The NASA Phoenix Mars Scout mission, launching August 2007 and landing May 2008 will conduct stationary surface and subsurface studies of Martian soil/ice. It will quantify the volatile inventory, determine the chemistry of the soils, search for habitable zones and biosignatures, identify potential chemical energy sources for supporting life, and identify the geochemical potential to preserve paleontological evidence. In addition to cameras, meteorological sensors, and atomic force (AFM) and optical microscopes, the Phoenix payload includes two instruments designed to provide insitu inorganic and organic chemical analyses, the thermal evolved gas analyzer (TEGA) and the MECA Wet Chemistry Laboratory (WCL). TEGA uses differential scanning calorimetry (DSC) to heat soil samples, release volatiles, and measure the phase change enthalpy. The TEGA mass spec obtains compositional information, isotope ratios, analyzes volatiles, correlates composition/release temperature, and samples atmospheric composition, isotopic ratios, and humidity. The WCL consists of an upper dispensing unit with stirrer and lower cell containing a sensor array. Each WCL accepts a 1 $cm^3$ sample and delivers it to 25 $cm^3$ of water containing standards and acting as leaching solution. The array of sensors includes ion selective electrodes (ISE) for $Ca_2^+$, $Na2^+$, $K2^+$, $Mg2^+$, $Cl^-$, $Br^-$, $NO_3^-$, $ClO_4^-$, pH, and dissolved CO2 and O2, electrodes for conductivity, redox potential (ORP), anodic stripping voltammetry (ASV) for selected heavy metals, and cyclic voltammetry (CV) to analyze redox couples. The WCL for the 2007 Phoenix Mission provides a low-mass/energy analytical device to obtain unique information about potential habitability and the history of the aqueous and geochemical Martian environment.

*Keywords:* electrochemistry, mass spectrometry, robotics, wet chemical methods
*Application code:* other
*Methodology code:* other

### CHIP-SIZED OPTICAL SPECTROMETERS IN PROCESS ANALYSIS

***Richard A. Crocombe***

*Axsun Technologies, 1 Fortune Drive, Billerica, MA 01821, USA*

Over the past 30 years, optical spectrometers have shrunk dramatically in size: from the room-filling behemoths of the 1970s, to benchtop instruments of the 1980s, and portable units in the 1990s. However, until now, the available analytical tools have been too delicate, too big, and too costly to deploy effectively throughout most industrial process lines. A new generation of MEMS-based miniaturized spectrometers has recently emerged, and these new instruments—spectrometers-on-a-chip—can now be mounted directly on a process probe, bypass loop, or pharmaceutical dryer and blender. This greatly facilitates installation and widespread deployment of process spectrometers, and leads towards their use as dedicated spectroscopic sensors. These breakthroughs in miniaturized spectroscopic instruments are enabling true process-analytical spectroscopy and will ultimately change pharmaceutical and chemical manufacturing so that quality is designed as an integral part of the production process—from raw materials through manufacturing to final packaging.

*Keywords:* instrumentation, near-infrared, pharmaceutical, process analytical chemistry
*Application code:* process analytical chemistry
*Methodology code:* vibrational spectroscopy

### SPECTROSCOPIC MONITORING OF UNIT OPERATIONS

***James Rydzak,[*] Christian Airiau, Ricard Escott, and Jill Parris***

*[*]GlaxoSmithKline, 709 Swedeland Road, Mailstop Uw2940, King of Prussia, PA 19406-0939, USA*

Process analytical technology (PAT) applications within any industry can be broken down into specific unit operations. PAT in the pharmaceutical industry has begun to more fully embrace the concepts of process monitoring and control. The specific requirements of individual unit operations such as reaction monitoring, dryer monitoring, and reactor cleanouts lend themselves well to spectroscopic measurements for real-time monitoring and control. This presentation will discuss applications of spectroscopy to some of these unit operations.

*Keywords:* chemometrics, near-infrared, pharmaceutical, process analytical chemistry
*Application code:* process analytical chemistry
*Methodology code:* near-infrared

### RAPID APPLICATIONS DEVELOPMENT FOR ON-LINE PROCESS ANALYSIS

***W. Michael Doyle* and Michael M. Power***

**Axiom Analytical, Inc, 17751-B Sky Park Circle, Irvine, CA 92614, USA*

The recent emergence of high-performance near-IR spectrometers that are small, robust, and fast is helping to accelerate the deployment of process analysis within the pharmaceutical industry. However, the right spectrometer is only part of the picture. A complete process analysis installation must include other elements such as robust and compact sample interfacing equipment and software capable of controlling one or more instruments, providing process modeling capability, providing process variable outputs in various formats, archiving the data, and interfacing to diverse data systems—all in a regulated environment. In addition, both the hardware and software must be compatible with rapid and economical applications development. This paper discusses some of the tools and methods currently available both for rapid applications development and for accomplishing efficient transition to the stringent requirements of online deployment. The hardware discussed will include probes and flow cells which combine the variable path length often required during applications development with the extreme robustness necessary for online analysis. The software portion of the paper will cover the use of a high-level scripting language—specifically tailored to the needs of analysis—to link diverse software resources so as to accelerate application development. Once an application has been developed, the needed security required for online deployment can be provided in the form of a series of user screens having only the required functionality.

*Keywords:* near-infrared, pharmaceutical, process analytical chemistry, spectrometer
*Application code:* process analytical chemistry
*Methodology code:* near-infrared

### REAL-TIME MONITORING OF ADSORPTION AND RETENTION OF DNA ON PATTERNED SELF-ASSEMBLED MONOLAYERS (SAMS) USING TOTAL INTERNAL REFLECTION FLUORESCENCE MICROSCOPY (TIRFM)

***Hye-Young Park,* Hungwing Li, Marc D. Porter, and Edward S. Yeung***

**Ames Laboratory, Department of Chemistry, Institute for Combinatorial Discovery, Iowa State University, USDOE, Ames, IA 50011-3020, USA*

Molecular interactions of biomolecules on compositionally functionalized surfaces at the solid-liquid interface are central to the development and application of DNA, protein, and small-molecule microarrays. Patterning self-assembled monolayers (SAMs) on a gold surface is one of the most widely used methods in creating these microarrays, and there have been numerous investigations of the preferential adsorption and localization of proteins, cells, and DNA on these materials. However, very little work has been done to examine interactions at the single-molecule level at these patterned interfaces. This presentation describes an investigation using YOYO-labeled DNA and patterned SAMs on optically transparent gold and total internal reflection fluorescence microscopy (TIRFM) for the real-time detection of single-molecule DNA adsorption. The experiments monitor the binding of YOYO-DNA to patterned SAM surfaces that are excited by an evanescent wave generated by $Ar^+$ (488 nm) laser on the surface of a silica prism. We therefore monitored the interaction of DNA at compositionally patterned SAM surfaces that were formed from alkanethiols with $-COOH$, $-OH$, and $NH_2$ terminal groups in solutions of varied pH and buffering capacity. Results show that the localization and magnitude of the interaction between DNA and the surface can be manipulated by changing pH.

*Keywords:* biotechnology, fluorescence, spectroscopy, surface analysis
*Application code:* bioanalytical
*Methodology code:* fluorescence/luminescence

### MULTIVARIATE MODELING OF DUAL SCANION AND DIFFERENTIAL MOBILITY SPECTRA

***Ping Chen* and Peter B. Harrington***

**Clippinger Laboratories, Center for Intelligent Chemical Instrumentation, Ohio University, Athens, OH 45701, USA*

Portable, low cost, and sensitive instruments are important for homeland security and have applications to onsite analysis. Ion mobility spectrometry (IMS) and differential mobility spectrometry (DMS) furnish instruments with these attributes. Multivariate curve resolution methods have proved useful because the signal acquired from these instruments is a mixture between the analyte ions (e.g., monomer and dimer ions) and the reactant ions (i.e., the background peak). Modern instruments such as the Lightweight Chemical Detector, the Itemiser3, and the DMS have the capability of acquiring positive and negative ion spectra simultaneously. A synergistic advantage exists for modeling positive and negative spectra simultaneously. Thermodynamic modeling characterizes charge and mass balance of the chemical equilibria that occur during atmospheric pressure ionization into the multivariate model. A comparison between SIMPLISMA, alternating least squares with nonnegativity constraints, and thermodynamic modeling will be presented.

*Keywords:* chemometrics, forensic chemistry, monitoring, sensors
*Application code:* homeland security/forensics
*Methodology code:* chemometrics

### CHEMOMETRIC DISCRIMINATION OF HONEYS AS A FUNCTION OF ORIGIN USING VISIBLE, MID-AND NEAR-INFRARED SPECTRA AND THEIR COMBINATIONS

***Nathalie Estephan,* Gerard Downey, and Doug Rutledge***

**Institute National Agronomique Paris-Grignon (INA P-G), 16 Rue Claude Bernard, Paris 75231, France*

Chemometrics applied to the analysis of spectroscopic signals has become a very significant asset in the food industry. Discriminant procedures are particularly important, for example for confirmation of authenticity. Combining the signals obtained from the application of techniques on some samples can facilitate the determination of the chemical functions responsible for any observed discrimination, by highlighting the relation between the two signals. The combination of two signals may also improve the discriminant power of the models created using multivariate chemometric methods. This simultaneous analysis can be carried out using either "outer product analysis" or concatenation. The outer product calculates the product of intensities at all combinations of frequencies in the two domains for each sample. Concatenation consists in attaching the two matrices of results side-by-side. In this study, these different combined matrices were analyzed using several discriminant methods—factorial discriminant analysis (FDA), FDA on principal components (PC-FDA), FDA on independent components (IC-FDA) and FDA on polar projection coordinates (PPC-FDA). These techniques were applied to mid-infrared, near-infrared, and visible spectra for the classification of honey samples as a function of their geographical origin. Profiles of loadings, scores or discriminating functions aid in the interpretation of the simultaneous variations in the combined signals, as well as the links between the spectral features and the classification criterion.

*Keywords:* chemometrics, food science, ftir, near-infrared
*Application code:* food science
*Methodology code:* chemometrics

### MEMBRANE ON A CHIP: APPLICATIONS OF TETHERED BILAYERS

***Ingo Koeper,* Randolph S. Duran, and Wolfgang Knoll***

**Max Planck Institute for Polymer Research, Ackermannweg 10, Mainz 55128, Germany*

Tethered lipid bilayer membranes are a powerful model for biomimetic membranes. They have been shown to be able to mimic biologic properties of a membrane, including the incorporation of functional membrane proteins. We were successful in the synthesis of a membrane architecture with good electrical properties (especially high resistance) that is tethered to a Si-surface. Functional incorporation of membrane proteins has been observed. The membrane can then be transferred to the gate oxide of a field-effect transistor (FET). We have built a new, compact readout systems, where different types of FETs can be used. Using incorporated ion channels, that have been genetically modified, this device is then a new kind of bioelectronic device for the detection of toxins.

*Keywords:* biosensors, biotechnology, sensors, surface analysis
*Application code:* drug discovery
*Methodology code:* sensors

### INTEGRATING INSTRUMENT CONTROL INTO A LABORATORY SOFTWARE FRAMEWORK

***Frank J. Tontala***

*Scientific Software, Inc, 6211 Owens Drive, Pleasanton, CA 94588, USA*

A modern laboratory data system must provide a wide variety of functionality beyond analytical instrument control. Features like security, audit trailing, data analysis, reporting, and data management are essential components of any laboratory software package. In a traditional software solution, this functionality is written as part of the application. This approach is easy to implement and provides tight integration. However, this architecture is closed, meaning that other applications have little or no access to the functionality and changes in any component impact the entire package. Open lab is a laboratory software framework that addresses this issue by decoupling the various components of a laboratory data system from one another. The components are packaged as a family of web services which can be independently accessed. This architecture has two big advantages. First, this functionality is available to any web-aware application which understands the interface. Secondly, enhanced or completely new web services can be easily substituted for an existing service, with the need to rewrite and the need to modify the calling application. This paper describes the integration of analytical instrument control with the open lab enterprise content management System. A number of development and implementation issues involved in providing generic software services will be described, including common access into the data base, analysis and reporting services, and the management of users and permissions. Support for GLP and 21 CFR 11 issues will also be discussed. Implementation of this integration will be described, showing how software interfaces are used to generically provide services to an instrument control subsystem.

*Keywords:* computers, instrumentation, laboratory informatics, software
*Application code:* laboratory management
*Methodology code:* laboratory informatics

### EMERGING MARKET FOR ELECTRONIC LABORATORY NOTEBOOKS

***Michael H. Elliott***

*Atrium Research & consulting, 5 River Road, Suite Number 216, Wilton, CT 06897, USA*

The market for electronic laboratory notebooks (ELN) is currently expanding with new products and technologies. However, there is a significant confusion in the market about what an ELN is, the types of technologies available, and the legal requirements for an ELN used for the support of patents. Based on information derived from an extensive market research project, this presentation is designed to provide an educational resource on the current market for ELNs, their integration with other laboratory informatics solutions, best practices for ELN implementation, and the future of the technology. An ELN as a foundation technology for scientific informatics will also be discussed.

*Keywords:* laboratory informatics, LIMS, sample and data management, scientific data management
*Application code:* laboratory management
*Methodology code:* laboratory informatics

### GLOBAL LIMS AND CDS PROJECTS: ARE THEY WORTH IT?

***Stuart M. Miller***

*Taratec Development Corporation, 1170 US Highway 22 East, Bridgewater, NJ 08807, USA*

Standardizing on a single laboratory information management systems (LIMS) or chromatography data system (CDS) product for all global laboratory operations is arguably the most common central objective of almost every major lab informatics project at virtually every large company that is actively planning, or implementing, one of these systems for their quality control or research labs. However, some recent experiences from the field on several global projects call into question the soundness of the concept and wisdom behind, such broad global standardization of LIMS and CDS. When viewed from the perspective of the lab, the potential pitfalls in global projects are significant and pose great risk to the individual lab's ability to adopt and efficiently use any new global system. First-hand experience on several projects shows that corporate lab management expectations are centered around establishing a new, harmonized system and new work practices across all their sites. These expectations are, more often than not, driven by their corporate IT partner's objectives of standardizing technology to reduce support and integration costs, a few to mention. However, standardization on a single product and global configuration will take significant effort from the business as well. It is the labs that will shoulder the burden of organizational change and business process improvement required to adopt the new standardized system. Perhaps this is precisely why it is IT and corporate lab management driving these global standardization projects instead of the labs themselves advocating for updated, globally standardized informatics systems from their IT departments. This presentation will critically examine the potential benefits and pitfalls, with particular emphasis on making recommendations and suggested solutions for the lab stakeholders in order to ensure that the inevitable transition to a global lab informatics system is as smooth and successful as possible.

*Keywords:* chromatography, laboratory informatics, LIMS
*Application code:* validation
*Methodology code:* laboratory informatics

### A PRACTICAL GUIDE TO TRUE REQUIREMENTS GATHERING FOR A SUCCESSFUL LIMS IMPLEMENTATION

***Don Crossett* and Todd Pollack***

**Thermo Electron Informatics & Services, 18 Commerce Way, Woburn, MA 01801, USA*

Without the appropriate laboratory requirements gathering and business analysis, companies stand to waste large volumes of time, effort, and money implementing LIMS solutions. As vendors, it is amazing to see the frequency with which customers fail to realize the importance of properly gathering and documenting the lab processes before moving forward with LIMS selection and implementation. This paper will not only focus on why this process is important, it will also outline best practices involved to ensure that the LIMS can maximize lab efficiency and work hand in hand with the lab's core competencies.

*Keywords:* lab management, laboratory informatics, LIMS, pharmaceutical
*Application code:* laboratory management
*Methodology code:* laboratory informatics

### LIMS: DUMP IT OR DEAL WITH IT—THE CASE FOR INTEGRATED SYSTEMS

***John G. Huntington***

*Gateway Enterprises, 2655 Winding Trail Drive, Boulder, CO 80304, USA*

Laboratory Information Management Systems (LIMS) software packages have been available for many years. For the smaller laboratory in particular, the success of these systems has often been limited. Frequently, the scope of the LIMS has been confined to simple reporting and sample management. But today's LIMS should not be just a software package, but rather an integrated system of software, people, and other tools, supporting and permeating all parts of the laboratory business, including technical, QA, sales, and administrative components. If such an integrated system (I-LIMS) is not part of your future planning, you will find yourself behind in the competitive marketplace. In this discussion, we will briefly touch on the common errors made by laboratories after the purchase of a LIMS that prevent the evolution to an I-LIMS. Then, we will summarize the main corrective actions that must be taken to move the laboratory in the right direction. Finally, we will focus on the key action that must be in place for any of these needed corrections to occur. Without this step, the I-LIMS will not develop and the company will fail to take advantage of the profound performance enhancements that are possible. The I-LIMS concept encompasses the business as a whole. When implemented over time, it will provide both profitability and smoother, more controlled operations management.

*Keywords:* data analysis, environmental, environmental/ water, LIMS
*Application code:* environmental
*Methodology code:* laboratory informatics

### THE NEW AGE OF CONFIGURABILITY AND FLEXIBILITY IN LIMS DESIGN FOR LIFE SCIENCES AND HIGH THROUGHPUT SCREENING

***Don Crossett***

*Thermo Electron Informatics & Services, 18 Commerce Way, Woburn, MA 01801, USA*

Years of trial and error in the LIMS industry have ushered in a paradigm shift in terms of moving away from highly customized solutions to more configurable, out-of-the-box functionality. The term “configuration” has been worn-out, overused, and oftentimes abused. Everyone claims to be configurable; nobody wants to admit that their product requires customization. At the same time, configurability should not come at the expense of flexibility in system administration. This paper will define the true benefits of configurability and flexibility in LIMS design as it relates to increased productivity, efficiency, and collaboration. It focuses on features and best practices which promote not only a successful implementation, but an effective long-term informatics solution which will allow the customer to keep up to speed with scientific advances.

*Keywords:* bioinformatics, drug discovery, laboratory informatics, LIMS
*Application code:* laboratory management
*Methodology code:* laboratory informatics

### THE EMERGENCE OF INTERNET-ENABLED INSTRUMENTATION

***Eric Reffett***

*National Instruments, 11500 North Mopac Expwy, Austin, TX 78759, USA*

This paper describes the emergence of a new class of instrumentation that utilizes an Internet Protocol (IP) address to provide connectivity to Laboratory Information Management Systems. The simplification of the instrument interface requires the LIMS to provide greater data management and control of the instrument. This paper discusses the issues of shifting control from the instrument to a LIMS system including both benefits and costs.

*Keywords:* instrumentation, LIMS
*Application code:* laboratory management
*Methodology code:* physical measurements

### ENTERPRISE STANDARDIZATION TO LOWER THE TOTAL COST OF OWNERSHIP OF LIMS

***Cathy Brown***

*Thermo Electron Informatics & Services, 18 Commerce Way, Woburn, MA 01801, USA*

Most companies contemplating a LIMS deployment today recognize the importance of standardizing one solution across their QA/QC or R&D labs. With the goal of harmonizing business processes in order to optimize efficiency, these companies seek solutions that can accommodate the needs of a diverse user group and geographically dispersed labs. By harmonizing processes and standardizing one LIMS solution, companies gain numerous operational and financial benefits, which ultimately will drive down the total cost of ownership (TCO) of LIMS. From a business perspective, one global solution facilitates better and more standardized enterprise reporting, enables easier access to data from across the organization, and lowers the cost of training. From an IT perspective, a standard configuration applied to all sites allows IT resources to develop deep expertise in one solution, making them better equipped to identify and solve problems before they occur. In addition to improved technical expertise and product reliability, key benefits of LIMS standardization include reduced vendor complexity, improved purchasing power, more efficient upgrades and integration to other systems, and streamlined IT management. Collectively, these benefits enable companies to lower the TCO of an enterprise LIMS deployment. This paper will explore the benefits of standardization as well as the potential challenges related to harmonizing processes and achieving buy-in from local labs already accustomed to a particular LIMS solution. In addition, balancing the needs of individual labs during the implementation of a global LIMS solution in order to ensure a successful deployment will be discussed.

*Keywords:* lab management, laboratory informatics, LIMS, pharmaceutical
*Application code:* laboratory management
*Methodology code:* laboratory informatics

### INSTRUMENT INTEGRATION IN PHARMACEUTICAL QUALITY ASSURANCE LABS: IS IT WORTH IT?

***Kyle McDuffie***

*CSols, Inc, 220 Continental Drive Suite 405, Newark, DE 19713, USA*

Quality assurance laboratories provide the final verification of quality for the manufactured drug product. As such, their role is crucial to the success of the company from the standpoint of product identity, efficacy, strength, purity, and safety. The impact of mistakes and/or noncompliance can be catastrophic in terms of product recall or regulatory action. This makes quality assurance laboratories very high-risk areas for regulatory scrutiny as related to cGMP and 21 CFR Part 11 compliance. Typically, the types of analyses performed by pharmaceutical laboratories are similar (defined by regulation) for similar dosage forms and do not change regularly for any given product. The most commonly utilized analytical techniques are HPLC (high performance liquid chromatography), UV-Vis (ultraviolet-visible spectroscopy), and automated dissolution equipment. Instrument integration and automation from the bench through lot release eliminates transcription and calculation errors. This simplifies the lot release process by helping to facilitate QA review. Instrument integration also makes it easier to demonstrate compliance with regulatory requirements. Surprisingly, most laboratories handle the data flow between the analytical instruments and other key systems (ie, LIMS, SDMS, and ELNs) manually. Automating the data flow can have significant benefits both within the laboratory and in the wider organization. In this presentation, the author discusses integrating analytical instrumentation to other key systems. The obstacles which historically have prevented pharmaceutical laboratories from implementing instrument integration will be discussed as well as the drivers for moving forward. Successful case studies will also be discussed; for example, dissolution testing, content uniformity, and stability analysis.

*Keywords:* laboratory automation, laboratory informatics, LIMS, pharmaceutical
*Application code:* regulatory
*Methodology code:* laboratory informatics

### BUILDING A BUSINESS CASE FOR LIMS

***Mark Fish***

*Thermo Electron Informatics & Services, 18 Commerce Way, Woburn, MA 01801, USA*

Implementing a Laboratory Information Management System (LIMS) or upgrading an existing system can require a significant investment and must often compete with other IT initiatives for funding. A compelling proposal that justifies the cost of the LIMS and demonstrates value to the organization is key to gaining approval for such a major project. A sound business case to justify a LIMS investment needs to take into account the full benefits and costs of the investment, and requires a detailed review of processes and practices both within and outside of the laboratory. Benefits may include hard savings, such as reduced labor, and material and operating expenses, as well as soft savings, such as improved sales, operating efficiency, customer satisfaction and competitive advantage. Costs are often easier to quantify and estimate, and include both one-time and ongoing expenses, such as hardware, licenses, project staffing, support, and maintenance. This paper will detail the process for developing a business case and includes suggestions and strategies for identifying relevant costs and benefits. Both quantitative and qualitative aspects required to justify a LIMS investment will be discussed, with a specific emphasis on benefits and costs that are "hidden" and commonly overlooked, but are equally important to reinforcing the overall business case. Sample data will then be presented in the form of a cash flow statement and will be used to demonstrate a return on investment (ROI) analysis.

*Keywords:* lab management, laboratory informatics, LIMS, pharmaceutical
*Application code:* laboratory management
*Methodology code:* laboratory informatics

### THE ROLE OF NEAR INFRARED SPECTROSCOPY TO VERIFY LABEL INFORMATION IN AGRO-FOOD PRODUCTS

***Ana Garrido-Varo***

*University of Córdoba, Faculty of Agriculture and Forestry Engineering Avda, Menendez Pidal S/N, Córdoba 14080, Spain*

Foods and feeds play an important part in the food chain. At the European level there is an extensive legislation on the labelling of feeds and foods. Without the capability of measurement and the availability of suitable instrumentation, the enforcement of that legislation is doubtful. During the past 15 years, the author and his colleagues have been developing robust NIRS calibrations which may be implemented in practise in the Spanish agro-food industry and, in particular, in the animal feeds, the Iberian pig, the dairy, and the olive oil industries. As part of that research, several NIRS instruments, cups, and fibre optics for the precise analysis of unground/intact agro-food products, avoiding the tedious task of sample preparation before scanning have been evaluated. The use of spectral data for purposes of authentication, identification, traceability, and labelling of foods and feeds has been an important part of the research done. Through a number of selected examples, the lecture will present data about the use of near-infrared spectroscopy as an affordable technology for fulfilling mandatory food and feed labelling and also for voluntary labelling aimed to fulfil consumer expectations on purity and authenticity.

*Keywords:* near-infrared, quality
*Application code:* food science
*Methodology code:* near infrared

### IDENTIFICATION OF GLUCOSE- AND UREA-SPECTRAL SIGNATURES IN IN-VIVO RAT SKIN TISSUE WITH NEAR-INFRARED SPECTROSCOPY

***Lingzhi Liu,* Mark A. Arnold and Jonathon T. Olesberg***

**University of Iowa, 296 LATL, Iowa City, IA 52242, USA*

Near-IR spectroscopy has been proposed as a means for measuring in-vivo blood glucose values noninvasively. The central analytical question in such measurements is whether calibration models generated using multivariate calibrations are based on glucose absorbance, secondary effects, or chance correlations. Identification of the presence of glucose-specific information in in-vivo IR absorbance spectra was studied in this work. Comparison was made with similar experiments where urea is infused rather than glucose. In-vivo glucose and urea infusion experiments were performed by collecting near-infrared absorbance spectra in the combination region (5000-4000 cm-1) with rats under anesthesia. The blood glucose or urea values of rats were allowed to stabilize at the initial levels for two hours and then increased substantially by venous infusion. Noninvasive tissue absorbance spectra were collected continuously with a Nicolet 670 Nexus FTIR spectrometer equipped with a fiber-optic interface. Blood glucose and urea values were monitored using samples taken from an arterial catheter with a HemoCue 201 glucose sensor and a Stat 2300 analyzer, respectively. Spectra were analyzed to identify spectral changes due to glucose and urea infusion. A pure glucose absorbance spectrum was used to calculate the glucose net analyte signal (NAS) by removing the projection of the spectrum onto the background factor space, which was built with the spectra collected when the glucose level was constant. The spectral residuals, after removing the projection from the spectra with high glucose concentrations, were strongly overlapping with the glucose NAS. Significant similarities between regression coefficient vector of a PLS model and glucose NAS were found. Similar results were obtained when the same procedures were applied for urea. All the evidence suggested that analyte specific information is available for non-invasive near-infrared detection.

*Keywords:* chemometrics, near-infrared, sensors
*Application code:* bioanalytical
*Methodology code:* near-infrared

### A NEW MODELING APPROACH TO PERFORMING RAW MATERIAL IDENTIFICATION BY FT-NIR

***Jeremy A. Linoski***

*ABB, Inc, 433 Northpark Central Drive, Suite 100, Houston, TX 77073, USA*

FT-NIR raw material identification is a well-proven and increasingly used technique in the pharmaceutical industry. FT-NIR in combination with powerful chemometric algorithms is a fast and reliable method that can be performed in the lab or warehouse. Commonly, three algorithms are available to build identification models: wavelength correlation, maximum wavelength distance, and principle component analysis (PCA). This presentation will discuss the use of discriminate partial least squares (D-PLS) as an alternate and superior choice for raw material identification. With the D-PLS approach, the construction of factors takes into account class membership with which PCA uses total-variability, making D-PLS models a superior alternative for discrimination. The use of D-PLS enables the selection of an individual instead of a common model library structure. An individual model library uses a separate model to discriminate each material group from the others. When an individual model is built for a new group and added to a list of existing models composing the entire library, these other groups are not influenced by its insertion thereby minimizing the library validation procedure. The advantage of an individual model structure over a common model will be reviewed.

*Keywords:* chemometrics, method development, near infrared, pharmaceutical
*Application code:* pharmaceutical
*Methodology code:* near infrared

### PORTABLE AND STABLE NEAR-INFRARED SPECTROSCOPIC SYSTEM FOR CHEMOMETRICS-BASED IN VIVO MONITORING

***Babs R. Soller,* Sherry Grobstein, Michael Parker, William Perry, Patrick Phillipps, Michael Phillipps, and Olusola Soyemi***

**Department of Anesthesiology, University of Massachusetts, Medical School, Worcester, MA 01655, USA*

Near-infrared spectroscopy (NIRS) can be used for the simultaneous determination of multiple medical parameters since the light penetrates skin and bone to probe underlying blood and tissue. In combination with chemometrics, NIRS can be used to simultaneously measure muscle pH and oxygen tension with blood hematocrit. These three parameters can be used to triage trauma victims and guide treatment by emergency responders. The use of chemometrics requires a spectroscopic instrument with a high level of optical stability, while long-term monitoring of critically ill patients limits the opportunity to recalibrate the instrument. We have designed and built a portable spectroscopic system for the field measurement of tissue oxygenation parameters required for this in-vivo application. A three-legged fiber optic cable connects the patient to the monitor. The 8 lb monitor contains unique electronic circuitry to rapidly power up the lamp and maintain its stability. A compact shutter allows routine collection of the dark signal as well as signal directly from the lamp for real-time correction of reflectance spectra collected from patients. System stability was characterized on a dysprosium oxide (DyO2) diffuse reflectance standard with absorption bands in the near infrared region used for our measurements (700–975 nm). Sample reflectance is converted to absorbance spectra using a 99% spectralon reflectance standard. Variation in DyO2 absorption was calculated as the relative standard deviation of the area under the spectral curve. Over 16 hours the absorption spectra were found to randomly vary by less than 1.5%, providing minimal impact on the accuracy of the in-vivo measurements. This system would also find use in other applications of remote spectroscopic monitoring. This work is supported by the US Army Medical Research Command and the National Space Biomedical Research Institute.

*Keywords:* instrumentation, medical, monitoring, near-infrared
*Application code:* biomedical
*Methodology code:* near infrared

### ACOUSTO-OPTIC TUNABLE-FILTER-BASED NEAR-INFRARED SPECTROMETER FOR ORGANIC VAPOR DETECTION

***John E. Carr* and Jon W. Carnahan***

**Department of Chemistry and Biochemistry, Northern Illinois University, Faraday Hall, Dekalb, IL 60115, USA*

Acousto-optic tunable filters (AOTFs) are digitally accessible, compact, solid-state wavelength selection devices that afford the construction of small, cost-effective spectrometers with no moving parts. This project investigates the potential applicability of an AOTF-based device utilizing the near-infrared (NIR) region. A NIR spectrometer has been assembled employing a noncollinear paratellurite AOTF as the wavelength selection device. The device operates in the range of 1.03 to 1.87 $\mu$m. Initial characterization of the instrument demonstrates adherence of the AOTF crystal to theoretical models governing frequency and wavelength relationships. Previous experiments were accomplished without an integrated computer program that simultaneously control the wavelength selection and detection systems. Wavelength selection was accomplished through manual control which involved altering several knobs during the course of an experiment. Thus, experiments were time consuming and initial results lacked averaged data sets as fewer experiments were performed. A program has been prepared, using LabView that allows computer control over wavelength selection and synchronous gathering of data, allowing for greater efficiency in the lab and increased S/N ratios as more experiments can be rapidly completed. Applicability of this spectrometer to the detection of several organic solvents, such as 1,2-dichloroethane and chloroform, is currently being investigated and results are being compared with commercial NIR spectrometer results. Experiments are preformed where the analyte solvent is introduced into an evacuated cylindrical sample cell (4.5 cm diameter, 10.5 cm length). This allows for detection of the analyte as a vapor. Construction of a portable spectrometer capable of detecting dangerous gas/spore clouds is envisioned as a result of this research.

*Keywords:* acoust-optic tunable filter, near infrared
*Application code:* general interest
*Methodology code:* near infrared

### EVALUATION OF INFRARED TECHNIQUES FOR THE ANALYSIS OF ANHYDROUS HF MIXTURES

***Valerie Bossoutrot,* Josyane Bruat, Eric Coffre, and Stéphane Richard***

**Air Liquide, Centre De Recherche Claude Delorme 1, Chemin De La Porte Des Loges, Jouy En Josas 78354, France*

Hydrogen fluoride is a toxic gas which is emitted from processes operating in waste and coal combustion, cement factories, and also in the glass industry. As a consequence, frequent emission monitoring is required by regulators. The feasibility and reliability of analytical techniques based on infrared detection has been studied to analyse HF mixtures in balance nitrogen. The figures of merit for Fourier transform IR and nondispersive IR has been evaluated in terms of selectivity, detection limit, and calibration range. A two-stages dilution device has been used to vary the HF concentration. In order to perform instrument calibration, optimized operating conditions have been established. Associated uncertainties have been calculated using a conventional approach for both instruments. This presentation will discuss the analytical figures of merit and their implications on the method detection limit as well as the associated uncertainty calculations.

*Keywords:* analysis, environmental, FTIR, gas
*Application code:* environmental
*Methodology code:* Near Infrared

### THE PAT FRONT END: STREAMLINING RAW MATERIAL INSPECTION BY FT-NIR AND FEEDING FORWARD INTO MANUFACTURING PROCESSES

***Scot Ellis* and Dave Edwards***

**Thermo Electron Corporation, 5225 Verona Road, Madison, WI 53711, USA*

The regulatory and quality-driven push for 100% inspection of raw materials for secondary (dosage form) pharmaceutical manufacturing demands changes in efficiency and control of the testing process. The process analytical technology (PAT) initiative makes "check-every-container" policies both more demanding and more useful. The inspection process itself can be streamlined by eliminating QC lab dependency, automating integration with enterprise data systems, and by eliminating human decision and data entry points where error can occur. A FT-NIR raw material inspection system facilitates the achievement of just-in-time manufacturing, eliminates lab dependency, and improves the overall efficiency and product quality. By further tying analytics to informatics systems, inspection results not only can improve quality and yields by ensuring material correctness but also can be used to feed forward quality and grade indicators into manufacturing processes. These in turn can be tuned for more predictable performance based on material properties.

This paper examines how a raw material inspection process streamlined for PAT can be facilitated through the use of FT-NIR analyzer and software system design. This may include barcode data integration, consideration for cleaning validation issues, system mobility, and automated LIMS or enterprise data system integration. It will also discuss examples of pharmaceutical manufacturing controls both at the point of inspection and by using fed-forward data.

*Keywords:* near infrared, pharmaceutical, process analytical chemistry, process control
*Application code:* pharmaceutical
*Methodology code:* near infrared

### VISIBLE NIR-MIR INDUSTRIAL STRENGTH SPECTROSCOPY: THE STATIC INSTANT MEASURE MULTIPLEXING SPECTROMERS (SIMMS). WILL IT MEASURE UP?

***Richard A. DeVerse,*** [*] ***Ronald R. Coifman, Andreas C. Coppi, William G. Fateley, Frank B. Geshwind, and Robert M. Hammaker***

[*] *Plain Sight Systems, Inc, 74-5599 Luhia Street, Unit D-6, Kailua-Kona, HI 96740, USA*

We will show the results of the build and test of a new solid-state multiplexing spectrometer that collects all of the multiplexed data resolution elements simultaneously. Short-lived and transient events can now be recorded within a single detector integration time and with the advantages of a multiplexed instrument. A new approach to highly multiplexed spectral measurements that can implement and or complement principal component regression vectors, and other encoding and chemometric methods directly enables end users to collect real-time high-throughput spectrometric measures in a compact, no-moving-part device. The measures of interest can be computed prior to impinging upon the detector at light speed while still in the optical domain, thus avoiding or eliminating post-data collection processing of the data. Only the data of interest and relevant to the measure are collected. The attributes of no moving parts and no scanning times create a completely new application space for spectrometry in extremely harsh environs typically not agreeable to spectrometric instrumentation. Conventional multiplexing methods that work to increase signal-to-noise ratios (SNRs) or decrease integration times by increasing throughput of spectrometric devices require measuring a series of encodements or multiplexed resolution elements over time $T$ which in turn dictates a maximum detector integration time of $T/N$ where $N$ is the number of resolution elements. The required scan time can be problematic if the source energy or sample changes over the time $T$ of the scan. The improvement in multiplexing spectrometry we demonstrate here eliminates noise contributions of scanning instruments when measuring fluctuating sources and transient events as each of a multitude of detectors views the source or sample fluctuations simultaneously.

*Keywords:* instrumentation, near infrared, process control, spectroscopy
*Application code:* process analytical chemistry
*Methodology code:* near infrared

### ON-LINE METAL PRECONCENTRATION USING A MODIFIED CHLOROMETHYLATED RESIN

***Valfredo A. Lemos,*** [*] ***Rennan G. Araujo, Patricia X. Baliza, Sergio L. Ferreira, and Juracir S. Santos***

[*] *UESB, Av José Moreira Sobrinho, S/N Bairro: Jequiezinho, Jequié, BA 45200-000, Brazil*

The synthesis, characterization, and application of an Amberlite XAD-2 resin functionalized with 3,4-dihydroxybenzoic acid (DHB-XAD) in an on-line system for metal preconcentration and determination by flame atomic absorption spectrometry (FAAS) is proposed. Ni (II), Cd (II), Pb (II), Co (II) and Cu (II) ions were sorbed in a minicolumn containing this material, from which it could be eluted using an acid solution. Eluent solution was carried by water and signals were measured as peak height by using an instrument software. DHB-XAD was synthesised by chloromethylation and Friedel-Crafts reactions. Ligand was coupled on the polymeric sorbent through a methylene group ($-CH2-$). The product was characterized by infrared spectra and elemental analysis. Achieved sampling rate was 48 samples per hour. Analytical parameters were evaluated for determination, such as sample pH, eluent concentration, sample and eluent flow rate, and so forth. Analytical characteristics and interferences were determined for metals, and the results demonstrated that the method could be applied for metal determination in several matrices.

*Keywords:* atomic absorption, flow injection analysis, separation sciences, solid phase extraction
*Application code:* polymers and plastics
*Methodology code:* separation sciences

### A RAPID AUTOMATED APPROACH TO THE GENERATION AND VISUALIZATION OF INVITRO METABOLISM, SOLUBILITY, AND LOG D USING LC/MS/MS AND UPLC/MS/MS UPLC/MS/MS

***Warren Potts,*** [*] ***Robert Plumb, and Kate Yu***

[*] *Waters Corporation, 34 Maple Street Mailstop Gc, Milford, MA 01757, USA*

The rapid screening of drug candidates for their physicochemical and metabolic properties is an essential part of the drug discovery process. The ability to rapidly profile thousands of compounds and display the data in a manner which allows compound ranking and selection for further development is critical to rational drug discovery. As most of the assays, such as solubility, Log D, CaCO2, and metabolic stability are now performed in a parallel manner using 96 well plates moving the throughput bottleneck to the analysis of the samples. In an attempt to address this issue LC/MS and LC/MS/MS with rapid gradient separations have been employed in this field. In this paper we will show how fast separations with MS/MS detection employing a multimode APCI and ESI probe are performed. We will also show how the throughput can be increased and ion suppression can be reduced by the use of small sub 2 $\mu$m particles and high pressure separations. Here we will show how the analysis times will be reduced from 5 minutes to less than 1 minute. We will also show how the multimode ionization probe coupled to an automated MS/MS optimization process can be to reduce the need for multiple analyses. The derived data will be analyzed using a single software package allowing the rapid quantification of the samples from many different assays and displayed in one simple spreadsheet.

*Keywords:* characterization, high throughput chemical analysis, liquid chromatography/mass spectroscopy, pharmaceutical
*Application code:* high-throughput chemical analysis
*Methodology code:* liquid chromatography/mass spectrometry

### SOFTWARE-ASSISTED SELECTION OF GENERIC SEPARATION METHODS IN THE HIGH-THROUGHPUT LABORATORY

***Michael McBrien,* Margaret Antler, Charlotte Blythe, Rhiannon Jones, and Mark Woodruff***

**Advanced Chemistry Development, Inc, 90 Adelaide Street, West Suite 600, Toronto, ON, Canada, M5H 3V9*

Generic or standard chromatographic methods have become a widely-used tool for high-throughput separations. A small set of generic chromatographic methods can be designed to produce sufficient resolution for the majority of samples. These methods can be used when it is not efficient to design high-quality separation methods for each sample. When a sample is submitted for analysis, this set of generic methods is screened to select the most appropriate separation method. Sample throughput can be further increased by using software to evaluate which generic method will be the most appropriate for a particular sample. In this work, we designed a limited number of analytical scale methods that address the vast majority of drug-like compounds. A training set was designed, containing a number of drug-like compounds. Each compound was analyzed with each generic separation method using MS detection. The retention times for each compound and method were then used to train the software. A set of drug-like compounds (not included in the training set) was then created to test the software. For each compound in the test set, the software chose the most similar compounds from the training set to create a prediction model. The best generic method for the test compound was then selected using the prediction model. This poster focuses on the experimental design of typical generic methods, and the selection of compounds for the training and test sets. Results for the test samples will be demonstrated, illustrating the quality of software-assisted generic method selection.

*Keywords:* high-throughput chemical analysis, HPLC, HPLC columns, software
*Application code:* high-throughput chemical analysis
*Methodology code:* liquid chromatography

### ON-SITE DETECTION OF CHEMICAL WARFARE AGENTS BY MONITORING TAPE METHOD

***Mieko Kanamori-Kataoka,* Satomi Abe, Gaku Ishiguro, Tetsuya Kawabe, Nobuo Nakano, Isaac Ohsawa, Yasuhiro Sano, Yasuo Seto, Kouichiro Tsuge, and Shigeharu Yamashiro***

**National Research Institute of Police Science, 6-3-1 Kashiwanoha, Kashiwa, Chiba 277-0882, Japan*

In the incidents of chemical terrorism and chemical weapon disarmament, on-site detection of chemical warfare agents (CWA) is important. The monitoring tape method has been utilized for the quantitative monitoring of hazardous gases in various factories. In the previous meeting, we reported the usefulness of this technique for monitoring of blistering agents such as mustard gas (HD) and lewisite 1 (L1). In the present work, the monitoring tape method was further improved for monitoring blistering agents and nerve gases by the use of pyrolyzing pretreatment. L1 was detected by three types of the pH indicator tapes with the detection limit of 0.5 mg/m$^3$. HD was detected by the Methyl Red tape with the detection limit of 0.2 mg/m$^3$ using pyrolyzing pretreatment adjusted to 500° C. Tabun and sarin were also detected by the cyanide sensitive tape and the Methyl Red tape, respectively, with pyrolyzing pretreatment.

*Keywords:* detection, environmental air, gas, monitoring
*Application code:* homeland security/forensics
*Methodology code:* other

### AUTOMATED COLD-ON-COLUMN INJECTION AND THE ANALYSIS OF EXPLOSIVE RESIDUES BY NEGATIVE CHEMICAL IONIZATION GC/MS

***Trisa C. Robarge,* Jessie Butler, Jason S. Cole, Meredith Conoley, and Eric Phillips***

**Thermo Electron Corporation, 2215 Grand Avenue Parkway, Austin, TX 78728, USA*

Analysis of explosive residues by gas chromatography/mass spectrometry (GC/MS) is complicated due to the unstable nature of the compounds themselves, which tend to decompose in the injection port. The use of a pressure-temperature programmable vaporizing inlet can overcome many of these limitations; however, a recent study was unable to optimize a method that allowed analysis of all of the nitramine, nitrate ester, and nitroaromatic compounds listed in the US EPA Method 8095. Therefore, an alternate injection technique was investigated, using true automated cold-on-column injection. A GC/quadrupole mass spectrometer that operated in electron capture negative chemical ionization (NCI) mode was coupled with an autosampler that provided automated cold-on-column injections. The technique was optimized for all components of EPA Method 8095, and a deuterated internal standard was used for quantitation purposes. Fast chromatography provided short analysis times, and linear fits for calibration were greater than 0.99 (r2) for all compounds. The calibration range extended from 1 to 1000 pg/$\mu$L in NCI SIM. Method limitations and applicability for alternate matrices will also be discussed.

*Keywords:* chemical ionization MS, environmental analysis, forensic chemistry, GC-MS
*Application code:* homeland security/forensics
*Methodology code:* gas chromatography/mass spectrometry

### IMPROVING ELECTRONIC NOSE TECHNOLOGY FOR APPLICATIONS IN DETECTING

***John F. Schneider* and Carrie M. Thomas***

**Argonne National Laboratory, 9700 S. Cass Avenue, Argonne, IL 60439, USA*

The detection of agricultural threats in incoming international mail and at ports of entry in the United States of America is the responsibility of the Animal and Plant Health Inspection Service (APHIS) of the United States Department of Agriculture (USDA). The USDA requires a detection process that is easy to implement and use, cost effective, and very sensitive. Currently specially trained beagles, their handlers, human inspectors, and X-ray machines accomplish the bulk of this work. In an attempt to augment the beagle brigade, Argonne National Laboratory is developing electronic nose devices by detecting and identifying characteristic or associated odors to address the agricultural threats.

The electronic nose is a device that uses an array of chemical sensors to mimic the way a human nose samples and processes odors. Each sensor produces independent responses for a given odor that are then converted from chemical signals to electrical ones with a connected data processor. The collection of all the sensors responses produces a "pattern" for a given odor. The data processing system compares the pattern of an unknown odor with a library of patterns previously databased. If a pattern matches one in the library, the odor is identified. The sensitivity of most electronic noses is in the low ppm ($\mu$L/L) range and not sufficient to detect contraband in the environment. In order to improve the sensitivity, methods for improving the sampling of contraband were explored and will be presented.

*Keywords:* agricultural, detection, sensors
*Application code:* homeland security/forensics
*Methodology code:* sensors

### FINGERPRINT IDENTIFICATION OF COCAINE ADULTERANTS BY HPLC AND GC

***Kristi J. Sellers* and Rick Morehead***

**Restek Corporation, 110 Benner Circle, Bellefonte, PA 16823, USA*

Illicit cocaine is commonly "cut" with adulterants or diluents that mimic the stimulant or local anesthetic effects of cocaine. Incorporating these additives into cocaine also increases the volume or weight of product available for sale, which results in increased profits for drug dealers. Because illicit cocaine composition (type of adulterant and diluents used) can be specific to a dealer, adulterant and diluent identification of seized cocaine is critical in determining the possible routes of distribution and sales. Mock samples of illicit cocaine were prepared using a variety of adulterants and diluents. Stimulants including caffeine, local anesthetics including procaine and lidocaine, and over-the-counter analgesics like phenacetin, were added to cocaine hydrochloride in varying concentrations. A simple "dilute and shoot" sample preparation scheme was used to dissolve the samples before analysis. High-performance liquid chromatography (HPLC) and gas chromatography (GC) methods were developed for identifying each adulterant or diluent added to cocaine. The method developed focused on maximizing the resolution of all of the compounds in the study while minimizing the total analysis time in order to increase sample throughput. "Fingerprint" identification of different cocaine samples could be achieved through the identification of the type and number of additives in the analysis using either analytical technique. Semiquantitative analysis of the concentration of each additive relative to the cocaine concentration in each sample was also possible.

*Keywords:* forensics, gas chromatography, HPLC
*Application code:* homeland security/forensics
*Methodology code:* liquid chromatography

### A LOW-COST REAGENTLESS MULTIANALYTE METABOLIC MONITOR

***Leah Tolosa,* Amelita Bartolome, Yordan Kostov, and Govind Rao***

**Chemical and Biochemical Engineering Department, University of Maryland, Baltimore County, Baltimore, MD 21250, USA*

We are developing an optical sensor array for glucose, lactate, glutamine, and fatty acids using highly sensitive and highly specific binding proteins. Glucose, lactate, glutamine, and fatty acids are important markers in determining metabolic profiles for various applications: diagnostics, sports medicine, military training, space medicine, and critical care. The protein biosensors used in the device are not enzymes. Thus, no reagents are required and no products are formed. Rather, the substrate/analytes bind to the corresponding genetically altered binding proteins. These proteins are labeled at a specific site with a polarity-sensitive probe (Em ~ 525 nm) and a long-lived metal-ligand complex at the N-terminal (Em ~ 620 nm). The 525 nm emission changes in intensity with analyte concentration while the 620 nm emission remains constant. The miniaturized optoelectronics readout system designed for dual-frequency modulation sensing will be described. The performance of this device is comparable to more expensive desktop instrumentation. A microfluidics device designed to contain the binding proteins will be shown. Initial data on the stability of the labeled proteins under various conditions will be presented.

*Keywords:* biomedical, biosensors, biotechnology, fluorescence
*Application code:* biomedical
*Methodology code:* fluorescence/luminescence

### HAND-HELD ELECTROCHEMICAL BIOSENSORS FOR DETECTION AND QUANTIFICATION OF MARINE MICROORGANISMS

***Michael J. LaGier,* Jack W. Fell, and Kelly D. Goodwin***

**University of Miami CIMAS, 4600 Rickenbacker Causeway, Miami, FL 33149, USA*

The field of biotechnology has advanced rapidly, paving the way to capitalize on such advances by the oceanographic community. Biosensors can provide a robust biotechnology for use in a variety of environmental monitoring applications. For instance, biosensors can provide early warning to close fisheries or recreational waters by real-time monitoring of coastal areas for toxic microorganisms. Electrochemical biosensors identify microbes by monitoring an electric current resulting from the oxidation or reduction of molecular markers. This study describes the use of hand-held electrochemical instruments (Alderon Biosciences, Inc Durham, NC) for detection of nucleic acids from the harmful algae *Karenia brevis* and fecal indicator bacteria (*Enterococcus* species). Detection was accomplished by tracking the reduction of a redox marker, tetramethyl-benzidine, at disposable carbon electrodes. The assays were optimized for identifying *K brevis* and fecal indicator bacteria (*Enterococcus* species). Detection was accomplished by tracking the reduction of a redox marker, tetramethyl-benzidine, at disposable carbon electrodes. The presence and abundance of *K brevis* or *Enterococcus* was consistent with microscopic observations or cell culture results, respectively. The work presented lays the foundation for the implementation of in-situ, electrochemical biosensors capable of simultaneously monitoring coastal environments for harmful algae, human pathogens, invasive species, and microbial indicators of pollution.

*Keywords:* biosensors, detection, electrochemistry, environmental analysis
*Application code:* environmental
*Methodology code:* electrochemistry

### DIAGNOSTIC TOOLS TO CHARACTERIZE NEAR-INFRARED SPECTRA FOR MULTIVARIATE ANALYSIS

***Dong Xiang,* Mark A. Arnold, and Wenjiao Lin***

**Optical Science and Technology Center, 114 IATL, The University of Iowa, Iowa City, IA 52242, USA; Department of Chemistry, 305 Chemistry Building, The University of Iowa, Iowa City, IA 52242-1294, USA*

Quantitative analysis from near-infrared spectra typically involves the construction of a multivariate calibration model from a set of calibration or training spectra. These training spectra must encompass the full range of variance expected for subsequent sample spectra. Sources of this spectral variation include changes in the sample matrix, changes in the instrumentation, and changes in the environmental conditions. Advances in near-infrared spectroscopic methods of analysis often come from the development of novel procedures and instrumentation designed to reduce the magnitude of spectral variance from these sources. Success is generally judged by lower standard errors of calibration and prediction. Unfortunately, few benchmark statistics are available to characterize the degree of spectral variance within a set of calibration or prediction data. As a result, few reports provide meaningful measures of overall spectral variance, which makes it impossible to compare datasets both within and between research groups.

We propose a set of benchmark statistics designed to characterize the spectral variance across a set of near-infrared spectra. These statistical parameters are generated from analyses of 100% lines and include root-mean-square noise values computed from different polynomial fits and the corresponding signal-to-noise levels. Examples of these statistical values will be presented for a set of buffer spectra collected with a Nexus 670 Fourier transform near-infrared spectrometer. Results are compared for spectra collected under conditions of (1) constant temperature and incident light intensity; (2) variable temperature and constant incident light intensity; and (3) constant temperature and variable incident light intensity.

*Keywords:* characterization, near infrared, statistical data analysis
*Application code:* bioanalytical
*Methodology code:* near infrared

### SINGLE-MOLECULE IMMUNOELECTROPHORESIS IN SUBMICROMETER PIPETTES AND IN LAB-ON-CHIP TECHNOLOGY

***Rene J. Pueschl,* Bernd W. Wenclawiak, and Christoph Zander***

**Adolf-Reichwein Str. 2, Siegen 57068, Germany*

A novel assay to identify proteins such as antibodies by using a single-molecule electrophoresis is described. The method applies special designed fluorescent tracer molecules, for example, antigen molecules. To separate the tracer from the tracer-target complex, the charge of the tracer molecules is chosen to be opposite to the corresponding tracer-target complex (antigen/antibody). The hydrodynamic flow was established by an electric field, a flow system served a cone-shaped micropipette with a diameter at the very end of about 0.5 $\mu$m. The tracer-target complexes were detected by laser-induced fluorescence where a diode laser at a wavelength of 635 nm served as an excitation source. The described method proved to be as sensitive that even antibodies at a concentration of 10–15 M could be registered within several seconds. A potential application for tumor diagnostics is demonstrated. Another point of interest is to transfer the method to the lab-on-chip technology. Therefore a microchip with a capillary electrophoresis application was designed and produced at the University of Siegen.

*Keywords:* capillary electrophoresis, lab-on-a-chip/microfluidics
*Application code:* biomedical
*Methodology code:* capillary electrophoresis

### AUTOMATED SPECIFIC DETECTION OF RNA SEQUENCES USING SEQUENTIAL INJECTION ANALYSIS

***Katie A. Edwards* and Antje J. Baeumner***

**Department of Biological and Environmental Engineering, Cornell University, Ithaca, NY 14853, USA*

Rapid detection of specific RNA sequences from environmental or clinical matrices can provide significant economic and health benefits. Early detection can minimize disease progression and allow for proper resource allocation in the event of suspected bioterrorism threats. Careful selection of nucleic acid probes results in the sandwich hybridization of RNA from the target organism, while minimizing false positives resulting from harmless bacteria. This principle has been used successfully in this lab with the development of single-use portable strip assays for the detection of various pathogens, such as *Bacillus anthracis*, *e coli,* and *dengue virus*. These assays have been adapted for use in a sequential injection analysis (SIA) system. SIA is a relatively new technique used typically to automate wet chemistry analyses while reducing reagent use and waste generation. While the aforementioned strip assays are useful in clinician's offices and for field testing, they are not ideal for repetitive sampling. Using a renewable bead surface within the SIA, the novel strip assay chemistry has been adapted for sequential measurements. The assay relies on the sandwich hybridization of a fluorophore-tagged DNA oligonucleotide (reporter probe) with RNA that may be present within the sample. This mixture is injected into the SIA system through a lab-on-valve (LOV) device where beads tagged with another DNA oligonucleotide (capture probe) can bind to the passing DNA-RNA complex. The specificity is a function of the sandwich hybridization. Detection is accomplished using

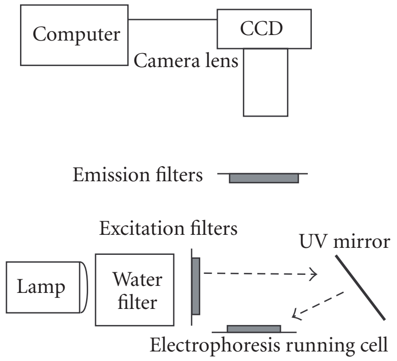

Figure 16: Schematic diagrams of the detection setup.

a photomultiplier-based flow-through fluorometer. We will present data on the detection of *B anthracis* RNA with a SIA system, and investigation of the hybridization kinetics of the target RNA binding to the capture and reporter probes.

*Keywords:* biosensors, flow injection analysis, fluorescence, nucleic acids
*Application code:* bioanalytical
*Methodology code:* fluorescence/luminescence

### A PROTOTYPE OF AUTOMATED TWO-DIMENSIONAL GEL ELECTROPHORESIS OF PROTEINS WITH NATIVE FLUORESCENCE DETECTION

***Aoshuang Xu* and Edward S. Yeung***

**Roy J. Carver Co-Laboratory and Ames Laboratory, Iowa State University, Ames, IA 50011, USA*

Two-dimensional gel electrophoresis practitioners have long waited for a fully automated system. We here present an integrated platform that is capable of complete automation from sample introduction to spots detection. The strip gel for the first-dimensional separation is fixed on the edge of a discrete planar stage before separation. A pair of platinum pin electrodes for isoelectric focusing (IEF) makes contact from underneath the stage. IEF is performed directly after rehydration and protein loading. After the first-dimensional separation, SDS equilibration is done on the same stage without moving the gel. The IEF stage is then moved horizontally to couple with a precast second-dimensional gel. The gap between the two gels less than 0.5 mm, is filled with poly (ethylene oxide) solution. After SDS-PAGE separation, a CCD camera is used to detect spots via protein native fluorescence excited by a Hg (Xe) lamp with the gel inside the running cell. Potential for full automation is demonstrated with 0.5 $\mu$g of *Esherichia coli* proteins on this miniaturized platform. More than 240 spots are detected in a total experiment time of less than 2.5 hours.

*Keywords:* automation, electrophoresis, fluorescence, proteomics
*Application code:* proteomics and genomics
*Methodology code:* fluorescence/luminescence

### MULTIDIMENSIONAL FLUORESCENCE DETECTION FOR FAST ON-CHIP DETECTION

***Sebastian Götz*** **and Uwe Karst***

*Tnw Department of Chemical Analysis, University of Twente, Enschede 7500 AE, The Netherlands*

Along with the total size of analytical systems, the light paths and detection volumes become smaller and smaller as well. Many analytical techniques have been applied to microfluidic systems, but due to its very high sensitivity and selectivity fluorescence detection has become the predominant detection method in these fields. However, when samples become more complex, standard laser-induced fluorescence (LIF) systems sometimes turn out to be not flexible enough and do not always provide as much information about the analytes as needed. We describe a new powerful and flexible detector setup consisting of a fluorescence microscope, a spectrograph, and a light intensified CCD camera. The microscope is used to focus the excitation light, generated by a xenon burner, onto the capillary or the microchip, and for collecting the emission light as well. In the spectrograph, the light is dispersed and the generated spectrum is projected onto the CCD. This setup enables the recording of online fluorescence emission spectra for various liquid separation techniques (HPLC, CE, on-chip CE). Detection frequencies of up to 60 Hz also cope with fast separations that can be carried out in microfluidic devices.

Three rhodamine dyes on a CE microchip could be separated in less than 7 seconds, and baseline separation occurred within 3 mm after the injection crossing of the microchip. A complete online emission spectrum was recorded every 0.02 seconds. The on-chip detection setup was used to determine NBD-derivatized taurine and g-aminobutyric acid (GABA) in beverages.

*Keywords:* capillary electrophoresis, fluorescence, multichannel spectrometry (CCD CID array)
*Application code:* environmental
*Methodology code:* fluorescence/luminescence

### MONITORING BIOMOLECULAR INTERACTIONS IN LIVING CELLS USING FLUORESCENCE ANISOTROPY IMAGING

***Zehui Cao,* Chih-Ching Huang, and Weihong Tan***

*University of Florida, Gainesville, FL 32611, USA*

Biomolecular interactions are often accompanied by changes in the molecular weights, such as bindings between two or more biomolecules, and digestion and synthesis of nucleic acids or proteins. This provides the basis for detection of such interactions using fluorescence anisotropy (FA). By labeling the interacting molecules with a fluorescent tag, changes in molecular weights can be monitored in real time using FA. While anisotropy measurements are usually conducted in homogeneous solutions, we have developed an imaging technique capable of providing localized anisotropy information. Based on a confocal microscope, the fluorescence emission from the sample is separated into two polarization states and two images are formed simultaneously. An anisotropy image is then calculated using these two images. As an application of anisotropy imaging, digestion of DNAs by nucleases has been monitored inside living cells.

Fluorescently labeled single-stranded DNA is injected into the cells and a decreased anisotropy is observed as the DNA is being cleaved by nucleases. In contrast, DNAs with a phosphorothioate-modified backbone have been found to be stable against cellular nucleases. Using the same approach, we have studied the stability of the telomere-like DNA sequences inside cells. DNAs which form a G-quadruplex structure similar to that of human telomeres are able to resist nuclease digestion, providing direct evidence that stability of single-stranded telomeres in cell nuclei is mainly due to the G-quadruplex structure. The newly developed anisotropy imaging technique may find interesting applications in intracellular biointeraction study.

*Keywords:* bioanalytical, fluorescence, imaging
*Application code:* bioanalytical
*Methodology code:* fluorescence/luminescence

### LEVERAGING THE POWER OF AN ENTERPRISE LIMS SOLUTION

***Cathy Brown***

*Thermo Electron Informatics & Services, 18 Commerce Way, Woburn, MA 01801, USA*

The full potential of an LIMS can best be realized when it serves as the foundation for an enterprise-wide IT strategy. LIMS are no longer lab-centric solutions designed merely for sample management and reporting, but instead are solutions capable of being integrated with key processes such as procurement, manufacturing, and quality management. An integrated approach ensures faster and more accurate data flow between the lab and the rest of the organization and, consequently, better, more efficient access to data. Making laboratory data available to managers and decision-makers greatly speeds decision-making and knowledge sharing across the organization. To enable seamless enterprise-wide integration, companies must select an LIMS capable of being more than a stand-alone system within a lab. An ideal solution is an LIMS that serves as an integrated platform capable of supporting key enterprise processes and systems. Key LIMS requirements include XML web services, allowing open communication with lab and enterprise applications, and client/server functionality, providing greater access to information and insight into data across all parts of the organization.

This paper will explore the important role an LIMS can play in a company's enterprise IT strategy and the challenges of selecting an implementing an LIMS solution that meets the needs of the broader organization.

*Keywords:* informatics, lab management, laboratory informatics, LIMS
*Application code:* laboratory management
*Methodology code:* laboratory informatics

### USING MOLECULAR BIOLOGY EXAMPLES TO ANALYZE LIMS IN THE LIFE SCIENCE LABORATORY

***Don Crossett***

*Thermo Electron Informatics & Services, 18 Commerce Way, Woburn, MA 01801, USA*

As researchers become more computer savvy and software becomes increasingly easy to use and administer, scientists are becoming a more of a predominant feature in the implementation process. IT staff are becoming less burdened by mundane LIMS administrative tasks and are freed up to focus on more difficult programing issues. This talk focuses on LIMS implementation from the scientist's perspective. This talk revolves around practical applications of LIMS in a molecular biology setting. It will focus on those features of information management that are useful to today's researchers. Data collection, data storage, dynamic process changes, communication with collaborators, instrument management, location tracking, scheduling, and workflows are a few of the items which will be covered.

The success or failure of a particular experiment relies on good data tracking. This talk includes real-world examples taken from molecular biology experiments, with subject matter relevant to all of life sciences. It will focus on aspects of LIMS that allow for better collaboration and protocol tracking and enhancement, ultimately resulting in better research.

*Keywords:* Laboratory Informatics, LIMS, Pharmaceutical
*Application code:* Laboratory Management
*Methodology code:* Laboratory Informatics

### BEST PRACTICES AND LESSONS LEARNED IN MULTISITE DEPLOYMENT OF LIMS: A TECHNICAL PERSPECTIVE

***Mark Evans***

*Thermo Electron Informatics & Services, 18 Commerce Way, Woburn, MA 01801, USA*

The trend toward standardization of LIMS for global enterprises has led to LIMS vendors adopting new system architectures and developing their capability to offer local support on a world-wide basis. In this presentation we explore a selection of deployment scenarios and how they compare in terms of system performance and scalability, ease of management, and ease of setup and deployment. We will also discuss a variety of important rollout considerations including options for multisite strategy, the handling of time zones and local language support.

*Keywords:* lab management, laboratory informatics, LIMS, pharmaceutical
*Application code:* laboratory management
*Methodology code:* laboratory informatics

### LABORATORY SYSTEM INTEGRATION IN BIOPHARMACEUTICALS

***Kyle McDuffie***

*CSols, Inc, 220 Continental Drive Suite 405, Newark, DE 19713, USA*

Biopharmaceuticals is an emerging area which promises to revolutionize drug manufacturing and deliver higher efficacy with lower doses. The development and deployment of these biologics-based systems requires faster, more efficient, and, in some cases, more complicated analytical laboratory testing. Instrument integration and laboratory automation of these "nonclassical" analytical techniques offer improved safety, efficacy, and manufacturing quality with lower cost. Commonly these techniques (ELISA, bioactivity, LC-MS, CE eg) have not been the mainstream candidates for automation and integration relative to HPLC (high-performance liquid chromatography). Many LIMS have also not serviced these areas well in the past. In this presentation, the author will describe case studies of novel approaches to laboratory system integration and automation in the biopharmaceutical environment. He will address the obstacles which historically have prevented most pharmaceutical laboratories from implementing instrument integration, the drivers for moving forward now and for successful case studies.

*Keywords:* biopharmaceutical, laboratory automation, LIMS, scientific data management
*Application code:* pharmaceutical
*Methodology code:* laboratory informatics

### ENTERPRISE INTEGRATION OF DATA ACQUISITION SYSTEM AND LIMS

***Donald Kolva* and Joanne Dussourd***

**Accelerated Technology Laboratories Inc, 496 Holly Grove School Road, West End, NC 27376, USA*

Often one of the primary reasons for laboratory information management system (LIMS) integration with enterprise systems is to enhance laboratory automation, reduce transcription errors, and increase productivity and efficiency. Primary advantages include the time savings in transcribing results from instruments to LIMS, bidirectional loading of run sequences and integrating complex calculations.

Laboratories often integrate instrumentation, and label printers, scanners, enterprise systems (ERP, CRM, chemical inventory, chromatography systems, and many others).

This talk will cover the business case for integration and review the potential cost savings as well as the total cost of ownership. This presentation will also address the challenges faced when integrating diverse data management and information systems and the need for standardization.

*Keywords:* Computers, database, instrumentation, lab management
*Application code:* laboratory management
*Methodology code:* laboratory informatics

### USING LIMS AND VBA TO MEET REGULATORY COMPLIANCE AND OPERATIONAL REPORTING NEEDS OF A WATER SYSTEM

***Michael Lehtola* and Sisay Mengistu***

**LABWORKS LIMS, PerkinElmer, 2135 Stonebriar Drive, El Dorado Hills, CA 95762, USA*

The information technology needs of modern day laboratories have undergone a major change in both complexity and scope, over the past decade. This change is driven in part by the complexity of the regulatory requirements and in part by the continually increasing sophistication and computer savvy of the customers that these labs serve. The need for an accurate and clear report cannot be overemphasized. Laboratories need to evaluate the presentation and data analysis capabilities of an LIMS system before making acquisition decisions. Furthermore, the ease with which a system can be customized to meet evolving customer reporting and presentation needs should be one of the criteria that tips the scale when selecting a new LIMS system. Starting with the release of MS Office 97ä, MicrosoftÒ has added features that used to be available in its highly popular Visual Basic program to its Office products macrofacilities. Microsoft called this new program Visual Basic for Applications (VBA). This creative integration of two widely used automation tools has greatly enhanced the ability of users to manipulate and present their data. The Portland Water Bureau purchased an LIMS in 2001. One of the required features was the ease with which the system could be customized to handle the reporting needs of the utility.

The main questions that needed to be answered were the following:

(i) Can the system handle the current regulatory and operational reporting needs?
(ii) Will it be possible to accommodate future reporting requirements?

This paper will discuss how Portland Water Bureau Lab was able to leverage the LIMS to meet the utility's complex regulatory and operational reporting and data presentation needs.

The paper will present materials on (i) LIMS' ability to seamlessly export analytical data to Microsoft Access database, and (ii) VBA's inherent capability to program Microsoft Office's applications object models and how they were used to achieve this.

*Keywords:* environmental/water, lab management, LIMS
*Application code:* laboratory management
*Methodology code:* laboratory informatics

### LIMS BEYOND THE LABORATORY WALLS

***Devender Gandhi***

*PerkinElmer, 7801 Monument Lane, Raleigh, NC 27615-3020, USA*

Some multitier LIMS have evolved to reach beyond the walls of the modern laboratory framework. Enterprise-wide requirements for timely decision support require that information be available immediately. The intricate and interdependent global economies demand that products be available quickly without maintaining large inventories. Federal, state, and local municipalities are requiring that results be turned around expeditiously. A laboratory information management system (LIMS) offers high uptime utilizing an infrastructure that makes it available across a local area network (LAN), a wide area network (WAN), or a web enabled environment. The modern laboratories are no longer limited to the confines of four walls instead; they could be spread throughout a campus or multiple locations, even on multiple continents. In order to take a proactive role in assuring the quality of products or services, an LIMS should allow the user community to produce automatic exception reports for specification violations. These reports may be set up to automatically email the responsible party, speeding up the correction of problems and reducing production costs. Reports may be scheduled for automatic printing or faxing using MS Fax or automatic email using formats such as RTF, XLS, or PDF file images. Exceptions to results with specifications can even be broadcasted as text messages on pagers directly to production or manufacturing personnel expanding the reach of the LIMS.

*Keywords:* lab management, LIMS, scientific data management, software
*Application code:* laboratory management
*Methodology code:* laboratory informatics

### RISK-BASED APPROACH TO VALIDATING LABORATORY SYSTEMS

***George Gallatig***

*Taratec Development Corporation, 1170 US Highway 22E, Bridgewater, NJ 08807, USA*

While considerable time and effort are generally expended validating laboratory systems, approaches can vary significantly. Some companies apply hordes of testers to challenge every aspect of new LIMS or chromatography data systems. They formally document, witness, and review every actual result. Other companies take a more laid-back approach trying to get by with as little as possible. The difference is typically driven by the interpretation of FDA guidelines by their quality people. What is really required to validate laboratory systems? How much testing is required? Is it necessary to test every function as part of the operational qualification? What advantage can be taken of knowledge gained from a vendor audit? How can one best take advantage of vendor-provided IQ/OQ? Is it necessary to create functional specifications for COTS applications? What can be omitted where multiple systems perform the same functions either at the same or at different sites? How does one apply this new risk-based thinking to validating laboratory systems? This presentation will address these questions and present some strategies for a practical approach to validation of laboratory informatics systems. The approach uses a formal risk analysis of the process and system to determine and document the scope and extent of validation necessary. This risk-based approach ensures that the system not only meets the needs of the business and complies with regulations, but also takes into consideration current regulatory thinking related to risk management and avoidance of unnecessary validation overhead. Examples will be drawn from case studies validating global LIMS and CDS.

*Keywords:* laboratory informatics, LIMS, validation
*Application code:* validation
*Methodology code:* laboratory informatics

### ENHANCED MONITORING PROGRAM THROUGH AUTOMATED SAMPLE SCHEDULING AND PALM UNITS

***Mathew Abraham,* Steve Rayburn, Fred Rider, and Russ Vranken***

**Accelerated Technology Laboratories Inc, 496 Holly Grove School Road, West End, NC 27376, USA*

The importance of monitoring programs (ie, drinking water) is high due to the repercussions of not having these programs in place. Federal regulations exist to enforce the application of the contaminant limits which are spelled out in the Safe Drinking Water Act and the associated monitoring programs and to ensure that the water is being treated appropriately before distribution. Monitoring programs are critical in managing processes often to meet regulatory requirements. These programs offer challenges to the laboratory in terms of ensuring that sampling (monitoring) events are not missed, that samples are collected in proper containers, received, and to ensure that the proper test and associated methods are performed. It is critical that samples are not missed during the collection or receiving process and that all samples have been collected and have a complete chain of custody form. These challenges can be met and monitoring programs can be enhanced through the use of the sample scheduling function in a laboratory information management system (LIMS) and integration of Palm hand-held units which allow the remote data collection. This presentation will cover the LIMS sample scheduling function and how it can improve the efficiency and accuracy of sample collection and receiving and the integration of Palm hand-held units to automate the process.

*Keywords:* lab management, laboratory automation, LIMS, monitoring
*Application code:* laboratory management
*Methodology code:* laboratory informatics

### THE OPTIMAL MAINTENANCE SOLUTION

***John Demeo***

*Thermo Electron Informatics & Services, 18 Commerce Way, Woburn, MA 01801, USA*

Several methods are available to fund maintaining instrumentation and equipment. This paper will explore the four main strategies for reducing, managing, and controlling maintenance costs. These strategies include service contracts, in-house repair, “going bare,” or a managed time-and-materials approach. The pros and cons associated with each strategy will be reviewed and analyzed. Insights to each will allow the attendee to determine the best strategy to be utilized depending on such factors as the category type of equipment or instrumentation, the primary use of the technology (such as research or production), and so forth.

Proven techniques to manage repairs on time-and-materials obtain the most out-of-service contracts and identify techniques used by vendors to increase time and material charges, will be discussed. Also included will be a best practices assessment outlining specific actions which can be taken to improve the attendees’ financial position.

*Keywords:* lab management, pharmaceutical
*Application code:* laboratory management
*Methodology code:* other

### THE NEW QUALITY ASSURANCE: THE CASE FOR I-QAS

***John G. Huntington***

*Gateway Enterprises, 2655 Winding Trail Drive, Boulder, CO 80304, USA*

The classical view of quality assurance (QA) is that it must be independent of pressures that arise from operational and profitability goals. Certainly, this is necessary and laudable. However, the unintended consequence is often that the QA program management may be isolated and uninvolved with daily activities. This results in higher costs to the laboratory for an ineffective program that fails to actually assure quality. The fundamental need for most laboratories is to mesh the QA program with the integrated laboratory information management system (I-LIMS) so that it becomes an integrated QA system (I-QAS). This system will then pervade the organization, improving its technical, administrative, sales, and business aspects. We will briefly touch on the five common errors made by laboratories in the development of a QA system. These errors effectively prevent the evolution to an I-QAS. Next, we will summarize courses of action that will enable the development of a true IQAS. The laboratory must judge the impacts of a change to a component of the system and respond with additional adjustments to that and other systems. Of critical importance is the impact on the economic viability of the laboratory. This requires ongoing management participation and involvement at all levels.

Finally, we will focus on the key transformational step that is required if these needed corrections are to occur. This step essentially focuses on company access to appropriate tools that enable the development of the systems required. Without this step, I-QAS will not develop and the laboratory will fail to take advantage of the performance and efficiency enhancements brought by effective QA. The I-QAS concept encompasses the business as a whole. When implemented over time, it will not only provide quality assurance, but also contribute to profitability and efficient operations. At the conclusion of this presentation, we will provide a real-world example of how this approach can impact laboratory operations. This example involves integration of the standard operating procedures (SOPs) used by the laboratory with the LIMS to create a more effective and economically viable control system.

*Keywords:* environmental, ISO 14000, ISO 9000, LIMS
*Application code:* laboratory management
*Methodology code:* data analysis and manipulation

### METHODS FOR SELECTING THE OPTIMUM LABORATORY GAS

***Steve S. Scheuring***

*Airgas, Inc, 250 North Radnor-Chester Road, Suite 100, Radnor, PA 19087, USA*

This presentation helps specialty gas users understand how to specify the optimum gas for their laboratory applications. When specifying gases, many buyers specify names and grades of gases, but really do not know what they are requesting due to the lack of industry standards. For example, there is no universal standard for defining “zero grade” gas, making it difficult to select the right gas.

Adding to the difficulty is confusion surrounding the definition of purity. Gas users may think they need the purest gas—gas that is “five 9s” or 99.999 percent pure. Rather than the level of impurity, they need to examine the actual impurity in the gas. It is these impurities that can affect analytical performance. When selecting gas, users need to examine the contaminants that could give false readings or cause equipment breakdowns and downtime. Different analytical apparatus are susceptible to different impurities. Once users understand these impurities, then they need to ensure the gas they specify has the lowest level of the impurities that their system can handle. This will enable users to produce the results they are looking for at the lowest possible cost. Looking at the specific analysis of the gases they are selecting also will allow the best comparison of various suppliers. The presentation covers differences in gas specifications, how contaminants are measured, what is actually measured, and sampling techniques.

*Keywords:* gas, gas chromatography, lab management, specialty gas analysis
*Application code:* laboratory management
*Methodology code:* gas chromatography

### AUTOMATED BASELINE REDUCTION IN HIGH-PERFORMANCE LIQUID CHROMATOGRAPHIC DIODE ARRAY SIGNALS

***Michael McBrien,* Andrey Bogomolov, Alexander Ivanov, and Eduard Kolovanov***

**Advanced Chemistry Development, Inc, 90 Adelaide Street West Suite 600, Toronto, Ontario, Canada M5H 3V9*

Diode array detectors (DAD) perform both qualitative and quantitative roles in high-performance liquid chromatography, as well as provide orthogonal detection in LC/MS experiments, allowing detection of peaks with little or no MS signal. The spectra that result from LC/DAD are useful for automated compound matches as well as extracting peak purity information. All of these functions can be hampered to varying degrees by the baselines that are especially typical in the nonroutine situations that dictate gradient experiments. This paper will detail a new algorithm for eliminating variable baseline signals chemometrically. This gives more accurate peak purity information, more accurate compound matches, lower detection limits, and more accurate quantitation. The performance of the algorithm will be shown using both real and simulated data.

*Keywords:* array detectors, chemometrics, HPLC, HPLC detection
*Application code:* general interest
*Methodology code:* liquid chromatography

### OPTIMIZED INSTRUMENT PARAMETERS FOR A HIGH-TEMPERATURE HPLC SYSTEM WITH FLAME IONIZATION DETECTION

***Jody Clark,* Dale Felix, Brian A. Jones, Brian A. Jones, and Stephanie J. Marin***

**Selerity Technologies, 2484 W Custer Road, Salt Lake City, UT 84104, USA*

The lack of sensitive universal detection in high-performance liquid chromatography (HPLC) is considered to be a serious obstacle in method development when analyzing non-UV-absorbing compounds. With the development of temperature-programed liquid chromatography (TPLC), it is now possible to use the flame ionization detector (FID)

in conjunction with pure water. Water is an attractive mobile phase for these types of separations because it shows no significant response in the FID and has the added benefit of being an environmentally friendly solvent.

Parameters have been studied to increase the stability and sensitivity of the flame when using the FID and superheated water as the mobile phase. A postcolumn split with varying backpressures was studied to optimize the stability of the flame and the amount of sample being introduced into the detector. Other critical parameters for flame stability are the type of restrictor, distance of the restrictor into the flame jet, and temperature of the FID. In this study we found that the flame was most stable at temperatures above 300°C at a distance between 2 and 3 cm below the tip of the flame jet. The restrictor that we found to perform very reliably was a stainless steel frit restrictor. The frit restrictor delivered the sample into the flame as a mist, eliminating the sputtering action observed when sample introduction is in droplet or stream form.

*Keywords:* GC detectors, high temperature, HPLC, instrumentation
*Application code:* pharmaceutical
*Methodology code:* liquid chromatography

### EXPLORING THE USE OF HIGH-TEMPERATURE HPLC FOR THE DEVELOPMENT OF HIGH-THROUGHPUT PURITY METHODS IN THE PHARMACEUTICAL ANALYTICAL LAB

***Ivelisse Colon*[*] *and Juan R. Gozalez***

[*]*Pfizer, Global R & D Eastern Point Road, Groton, CT 06340, USA*

The development of stability-indicating methods is a major focus of an analytical laboratory in a pharmaceutical research environment. While these methods are highly selective as shown by forced-degradation samples early on in the development stage, they usually employ extended run times (more than 45 minutes) and gradients involving high percentages of organic solvents. Even though these methods are appropriate for quality control and stability assessment tasks, they might not be attractive or time/cost effective for other research activities within the analytical laboratory and/or partnering with other R&D groups. These activities might include impurities isolation/characterization, purity measurements for synthetic route development, drug safety/discovery analytical assessments, prototype/accelerated stability for development purposes, among others. The advantages of conducting chromatography at elevated temperatures have been previously documented, and with recent instrumental advances (including mobile-phase preheating and postcooling), the potential for development of selective high-throughput methods for purity assessments has become viable. A collection of examples will be discussed, where fast methods have been developed with similar selectivity to that of the traditional method in one-third of the run time. This is attractive for fast development activities and collaboration with research partners in the pharmaceutical environment. The additional advantages of chromatography at higher temperatures, particularly lower backpressures, sharper peaks, and less organic solvent usage, will be shown with specific examples on how these have demonstrated superior results over existing methods. The dramatic increase in signal-to-noise ratio at higher temperatures has also allowed the development of methods with increased sensitivity.

*Keywords:* high temperature, HPLC, liquid chromatography, pharmaceutical
*Application code:* pharmaceutical
*Methodology code:* liquid chromatography

### NEAR-INFRARED SPECTROSCOPY: A UNIVERSAL TOOL FOR QUALITY AND PROCESS CONTROL

***Heinz W. Siesler***

*Department of Physical Chemistry, University of Duisburg-Essen, Schuetzenbahn 70, Essen 45117, Germany*

The growing demand for product quality improvement and production rationalization in the chemical, pharmaceutical, polymer, cosmetic, food, and agricultural industries has led to the increasing substitution of time-consuming or unspecific analytical procedures by faster, more specific, and environmentally compatible techniques. In this respect, of the different methods of vibrational spectroscopy, primarily the near-infrared technique has emerged over the last decade as an extremely powerful tool for industrial quality control and process monitoring. The lecture will specifically address the experimental flexibility and high-throughput character of NIR spectroscopy as well as the extreme diversity of its applications and will highlight the rationalization effect with the intention to further support its acceptance and encourage the enhanced implementation in industrial environments.

*Keywords:* chemometrics, near infrared, process control, quality control
*Application code:* quality
*Methodology code:* near infrared

### INLINE NEAR INFRARED FOR CONTROL OF FATTY ACID DISTILLATION: FEASIBILITY STUDY

***Marchel Snieder,*[*] *Wei G. Hansen, and Sophie S. Wiedemann***

[*]*Uniqema, Buurtje 1, Gouda 2802Be, The Netherlands*

Close monitoring of composition is needed to reach cost-effective operation in fatty acid distillation. However, introduction of inline gas chromatography (GC) requires heavy investment, constant maintenance, and remains risky and limited as it involves cutting-edge technology (especially with medium- and long-chain fatty acids). Conversely, near-infrared (NIR) spectroscopy has recently gained a wider acceptance in process analytical chemistry. Both instrumentation and chemometric tools have reached a level of maturity that allows the technique to revisit some more challenging areas. Its main advantages over GC are a very short response time and a much lower overall cost.

This paper investigates the possibility to use inline NIR for determination of medium- to-long-chain fatty acids during distillation. It reports some detailed spectra investigations (including the identification of the $CH_n$ bands), and a study of the effect of scanning temperature on the spectral area of interest (CH second overtone and OH first overtone bands). Based on this spectra understanding, it also presents various modeling options. Finally, some limitations of the NIR technique compared to GC analysis are discussed.

*Keywords:* near infrared, process analytical chemistry
*Application code:* process analytical chemistry
*Methodology code:* near infrared

### DIMENSIONALITY REDUCTION OF NIR SPECTRAL DATA USING GLOBAL AND LOCAL IMPLEMENTATIONS OF PCA FOR NEURAL NETWORK CALIBRATIONS

***Igor V. Kovalenko,* Charles R. Hurburgh, and Glen R. Rippke***

**Iowa State University, 1551 Food Sciences Building, Ames, IA 50011, USA*

Artificial neural network (ANN) learning algorithm has established itself as a strong alternative to traditional linear calibration methods used in near-infrared (NIR) spectroscopy. One of the limitations of this method comes from the fact that its generalization capacity could be effectively employed only when the ratio of available training samples to a number of neuron interconnection weights and biases (unknown regression parameters) is sufficiently large. Traditionally, this ratio is increased by reducing dimensionality of ANN input space by compressing X data using principal component analysis (PCA). However, several other dimensionality reduction methods have been shown to outperform it. An attractive data compression method that combines two multivariate data analysis techniques, namely clustering and PCA, has been described in the literature. This approach, known as local PCA, overcomes PCA's global linearity by performing dimensionality reduction task in two steps: division of the data space into clusters and local compression of each cluster using PCA. Therefore, the objective of this study was to compare applicability of global and local implementations of PCA compression to NIR calibration problems solved with ANN regression. In this experiment, two datasets were used for development of control (based on PCA) and experimental (based on local PCA) ANN calibrations. Predictive ability of two types of models was compared for both datasets. The results demonstrated that local PCA could significantly outperform traditional global PCA compression. However, the choice of preferred dimensionality reduction method was case dependent. In addition, the study showed that performance of local PCA-based calibrations degraded rapidly, while global PCA allowed achieving higher compression rates at minimal cost of prediction accuracy.

*Keywords:* calibration, chemometrics, near infrared, neural network
*Application code:* agriculture
*Methodology code:* near infrared

### BLEND UNIFORMITY MONITORING USING NIRS: QUALITATIVE AND QUANTITATIVE ANALYSIS

***Promit Das,* Ross Herrold, and Terrance R. Kinney***

**Control Development, Inc, 2633 Foundation Drive, South Bend, IN 46613, USA*

Blending of powders is a critical step in the manufacturing of certain products in the pharmaceutical industry. The process analytical technology (PAT) initiative by the FDA encourages manufacturers to incorporate sensor technologies that enable real-time monitoring of production processes. This will eventually lead to an increase in process knowledge and higher product yield. Near-infrared spectroscopy (NIRS) has been identified as one of these enabling technologies. In this paper we will concentrate on the use of NIRS for blend uniformity monitoring of powders. We will present data, results and different methods of analysis, both qualitative and quantitative. A brief section will describe diode array instrumentation and its suitability for this type of measurement.

*Keywords:* array detectors, near infrared, pharmaceutical, spectrometer
*Application code:* pharmaceutical
*Methodology code:* near infrared

### A PROCESS ANALYTICAL NEAR-IR CHEMICAL IMAGING INSTRUMENT

***Kenneth S. Haber,* Fiona Clarke, Linda H. Kidder, Eunah Lee, and E. Neil Lewis***

**Spectral Dimensions, Inc, 3416 Olandwood Court, Suite 210, Olney, MD 20832, USA*

One of the more fundamental process analytical technology (PAT) measurements currently being contemplated in the pharmaceutical industry is the in-situ monitoring of blend uniformity during the course of a manufacturing run. While near-infrared chemical imaging is routinely used to measure blend quality on multiple spatial scales in the laboratory, it has not previously been applied to in-situ blend measurements in a manufacturing environment. We describe the first process near-infrared chemical imaging system designed explicitly to analyze pharmaceutical mixtures during the course of a blending run, without extracting samples from the blender. We characterize the performance of the instrument in a trial blending run performed on a modified commercial V-blender.

The instrument consists of an optical head, outfitted with filtered broadband sources and collection optics, coupled to a modified stainless steel NEMA type 4X enclosure which houses additional optics, a tunable filter, and a TE-cooled mercury-cadmium-telluride (MCT) focal-plane array detector. The filter can be continuously tuned from 1000 to 2450 nm and has a spectral bandpass of 9 nm at 1900 nm. The coupling is accomplished with a 1/2 m length of coherent, IR-transmissive fiber bundle, whose distal end is reimaged through the filter onto the focal-plane array with a 1 : 1 correspondence between fibers and pixels. The optics head is bolted in one of 5 positions directly onto a hygiene flange on the blender which has been modified to accept a 6 mm thick sapphire window. In each position a 5 mm × 6 mm portion of the sample is imaged onto the focal plane with 20 micron/pixel resolution. The filter may be scanned sequentially or in a random access mode to generate spectral image cubes.

Results from a trial blending run of saccharin, lactose, and avicel will be presented, showing that in this case spatial homogeneity of the ingredients requires approximately 5 minutes of blending.

*Keywords:* imaging, near infrared, pharmaceutical
*Application code:* pharmaceutical
*Methodology code:* near infrared

### EXPLOITING PROCESS ANALYTICAL TECHNOLOGY BENEFITS THROUGH DEEP BUSINESS INTEGRATION

***Brian Davies,* John Dickson, Scot Ellis, and Zafar Kamal***

**Thermo Electron Corporation, 5225 Verona Road, Madison, WI 53711, USA*

Measuring, understanding, and ultimately controlling manufacturing processes offer pharmaceutical companies a route to greatly enhanced effectiveness and efficiencies in their factories. Utilizing process analytical technology (PAT) product quality will be enhanced and assured, manufacturing waste reduced, and production assets better utilized for both business and patient benefit. However these benefits will not be realized by simply putting analytical measurement systems into manufacturing processes; they will come from a full and informed integration of PAT into the manufacturing business process.

This paper will describe the PAT business integration process from establishing the appropriate point in the process for the analytical measurement, the necessary capabilities of a process analytical system, measuring and modeling manufacturing processes through to the effective use of the process information to take control of processes, make business decisions, and be a long-term business asset via informatics and knowledge management tools.

The necessary technologies, skills, and capabilities to accomplish this deep integration will be discussed, with examples drawn from both pharmaceutical and other complementary industries used to illustrate how PAT benefits can be realized practically and effectively.

*Keywords:* laboratory informatics, materials characterization, process analytical chemistry, process control
*Application code:* pharmaceutical
*Methodology code:* near infrared

### INTEGRATION OF A NEAR-IR ANALYZER INTO THE PROCESS CONTROL LOOP-OPC-BASED SOLUTIONS FOR PAT

***Jeffrey Hirsch***

*Thermo Electron Corporation, 5225 Verona Road, Madison, WI 53711, USA*

Industrial fourier transform near-IR (NIR) analyzers provide a dedicated solution for both routine and complex QA/QC measurements. Common tasks like moisture analysis or chemical discrimination can be accomplished quickly and nondestructively with proven time and cost savings. Near IR also has the capability to go in line where it can be used for endpoint determination and process parameter control, making it an ideal candidate as a process analytical technology (PAT) for pharmaceutical manufacturing. One of the biggest hurdles to putting an FT NIR system in line is the lack of ability to communicate with other equipment using a standard communication and feedback protocol. OLE for process control (OPC) is widely recognized as the new standard for process communication because of its simplicity and applicability to a wide range of equipment and systems. OPC can be used to tie together systems or equipment utilizing seemingly disparate communications protocols such as modbus, profibus, and 4–20 mA. It provides an ideal interface between a process analyzer, control systems, and information systems. The current study will discuss the use of real-time output from an FT NIR analyzer into an OPC framework and its impact on monitoring and control of manufacturing processes, such as blending and drying.

*Keywords:* near infrared, process analytical chemistry, process control, vibrational spectroscopy
*Application code:* process analytical chemistry
*Methodology code:* near infrared

### NEW GENERATION OF AOTF-NIR ANALYZERS FOR ON-LINE APPLICATIONS

***Igor Nazarov***

*Brimrose Corporation of America, 5024 Campbell Blvd, Suite E, Baltimore, MD 21236, USA*

We will present the use of a new generation of rugged and miniaturized AOTF-NIR spectrometers as cost-effective solutions for the pharmaceutical and other industries. The working combination of all these analyzers along with software is known as the Brimrose Process Quality Management System. We will present the Luminar 5030, a sturdy portable hand-held analytical mini-spectrometer, and the Luminar 4060 mini-multiplexer that are used in multiple locations for conducting nondestructive and contact/noncontact analytical testing and inspection in warehouses, production floor, laboratories, and so forth. The next generation of Luminar 4030 miniature process on-line analyzers will also be presented. The Luminar 4030 analyzer can be used for conducting real-time on-line applications such as blend uniformity analysis, monitor on-line dryers (spray, FBD, etc), reaction monitoring, and so forth.

*Keywords:* acoustic-optic tunable filter, near infrared, process control, process monitoring
*Application code:* pharmaceutical
*Methodology code:* near infrared

### CORRELATION OF WATER CONTENT IN POWDERS, COATED AND CORE TABLETS BY NIR AND KARL FISCHER

***Seema Tomer,* James E. Mullins, James Pazdan, Venkat S. Tumuluri, and Richard Vivilecchia***

**Novartis Pharmaceuticals Corporation, One Health Plaza, East Hanover, NJ 07936-1080, USA*

Near-infrared (NIR) spectroscopy is an instrumental technique, which detects the excitation of C−H, O−H, and N−H bonds. One of the most common applications reported is quantitative moisture analysis due to the specific absorption of near-infrared light by the O-H bond. Drug substances and lyophilized cakes have been successfully analyzed for moisture content by NIR using Karl Fischer (KF) titration as the reference method. The correlation between the two techniques was investigated using powders and tablet cores. The correlation of moisture content in coated tablets is ongoing. The NIR calibration curve was created by hydrating the tablets and powders in the range of 0.5% to 15% water content, at constant temperature and humidity levels in a VTI vapor sorption analyzer. These samples were then analyzed by NIR followed by KF to obtain the moisture levels for each sample. Then a partial least squares (PLS) regression model was employed to create the calibration curve and obtain the correlation value. Once the curve was created, random samples were used to validate and prove its predictive capabilities and robustness. These validation samples were also used to determine the standard error of prediction (SEP) of the method for powders and cores; excellent values were obtained for SEP, in the range of 0.13% to 0.15%. This method of measuring moisture is nondestructive and much faster than conventional Karl Fischer method of measuring moisture content.

*Keywords:* process analytical chemistry
*Application code:* process analytical chemistry
*Methodology code:* near infrared

### ANALYSIS OF THE ADSORPTION EVOLUTION OF WATER MOLECULES ON SILICA GEL SURFACE BY NEAR-INFRARED SPECTROMETRY AND CHEMOMETRICS: AN ALTERNATIVE WAY TO DETERMINE SILANOL NUMBER

***Alfred A. Christy* and Per K. Egeberg***

**Faculty of Mathematics and Sciences, Agder University College, Serviceboks 422, Kristiansand 4604, Norway*

The adsorption properties of silica gel particles depend on the surface silanol groups and these groups are exploited for chemical modification in several different fields including catalysis, separation science, and polymers. Because of the importance of silica surface functional groups in several different fields, a lot of research has been done to find ways for the quantitative determination of the silanol and other functional groups on silica surface.

Silanol number is the number of OH groups present per nm2. Wet chemical methods have been used in the silanol number determination. These methods are tedious and time consuming.

In this paper we report a revolutionary way of studying adsorption of water molecules on silica gel surface by following near-infrared profiles of the dry silica gel and the weight gained over a certain period. These data would provide evolutionary profiles and these evolutionary profiles can be treated by chemometrics to identify the type of water molecule adsorption on the surface. The weight difference would give the number of water molecules on the surface and the weight difference at an appropriate time during the evolution would give monolayer water molecular adsorption on the surface. The weight of the monolayer water molecules can be used in the determination of silanol number.

*Keywords:* adsorption, chemometrics, modified silica, near infrared
*Application code:* materials science
*Methodology code:* near infrared

### BIOINFORMATICS AND MASS SPECTROMETRY FOR NEUROPEPTIDE DISCOVERY IN THE HONEYBEE, *Apis mellifera*

***Andinet Amare,* Michael A. Ewing, Amanda B. Hummon, Timothy A. Richmond, and Jonathan V. Sweedler***

**Chemistry Department, University of Illinois at Urbana-Champaign, 505 South Mathews Avenue, Urbana, IL 61801, USA*

Neuropeptides are vital for cellular communication and are difficult to infer directly from genomic data. We have developed a method to identify putative neuropeptide genes and confirm their products by employing a combination of mass spectrometry and bioinformatics tools. The genome of the honeybee, *Apis mellifera*, has recently been sequenced; however, very few of its neuropeptides are confirmed by immunoreactivity or by mass spectrometry, respectively (S. Eichmuller, M.Hammer, and S.Schafer, "Neurosecretory cells in the honeybee brain and suboesophageal ganglion show FMRFamide-like immunoreactivity," *J. Comp. Neurol.*, vol. 312, no.1, pp. 164–174, 1991. and H. Takeuchi, A. Yasuda, Y. Yasuda-Kamatani, T. Kubo, and T. Nakajima, "Identification of a tachykinin-related neuropeptide from the honeybee brain using direct MALDI-TOF MS and its gene expression in worker, queen and drone heads," *Insect Mol. Biol.*, vol. 12, no. 1, pp. 291–298, 2003). We perform tandem MS on brain homogenates using ESI-Q-TOF MS. We also carry out homology searches using known neuropeptides from other insects on the *A mellifera* EST and genome libraries and predict putative genes by employing a suite of bioinformatics tools. Using our neuropeptide prohormone processing prediction algorithm (A. B. Hummon, N. P. Hummon, R. W. Corbin, L. Li, F. S. Vilim, K. R. Weiss, and J. V. Sweedler, "From precursor to final peptides: a statistical sequence-based approach to predicting prohormone processing," *J. Proteome Res.*, vol. 2, no. 6, pp. 650–656, 2003), we predict the final neuropeptides from the genes and confirm by MS. We and our collaborators have used this approach to discover over 30 putative genes and confirmed the primary structure of over 40 predicted neuropeptides. This work illustrates the importance of combining mass spectrometry and bioinformatics for successful discovery of new neuropeptides.

*Keywords:* bioinformatics, mass spectrometry
*Application code:* proteomics and genomics
*Methodology code:* mass spectrometry

### ONLINE MONOLITHIC PRECONCENTRATOR-CZE FOR THE ENRICHMENT OF PROTEINS IN HUMAN PLASMA

***Jenny M. Armenta,* Binghe Gu, and Milton L. Lee***

**Department of Chemistry and Biochemistry, Brigham Young University, C100 Bnsn, Provo, UT 84602, USA*

The analysis of proteins in biological fluids by capillary electrophoresis (CE) has gained interest in clinical chemistry. However, protein analysis by this technique is challenging due to low analyte concentration and poor concentration limits of detection (CLOD). Coupling preconcentration techniques with capillary electrophoresis greatly improve the CLOD. An online preconcentration-CZE method that selectively preconcentrates proteins would be very useful for the analysis of low-abundance proteins and would establish CE as a major tool in biomarker discovery. To address this, the development of an online protein G monolithic preconcentrator-CZE device is reported. To generate active groups for protein immobilization, glycidyl methacrylate (GMA) was used to prepare the monolith.

A 2 cm long GMA monolith was cast inside a 75 $\mu$m id fused silica capillary that had previously been coated with alternating thin films of physically adsorbed negatively (dextran) and positively (polybrene) charged polymers. Protein G was covalently bound to GMA. A 3.3 nM IgG solution was successfully preconcentrated using this device. Online preconcentration-CZE of IgG was accomplished in four steps. First, the protein G monolithic preconcentrator was rinsed with PBS before a 160 $\mu$L volume of 3.3 nM IgG solution was pumped through. Next, the preconcentrator was conditioned with 50 mM ammonium formate-formic acid, pH 7.6. Then, IgG was desorbed from the protein G monolith by injecting a small plug (equivalent to three times the monolith length) of 50 mM formic acid. Finally, IgG was separated by CZE using a Crystal CE 300 system (ATI, Madison, WI) equipped with an online Crystal 100 variable wavelength UV/Vis absorbance detector.

Monoliths from different formulations were prepared and evaluated for binding capacity to optimize the monolith formulation for protein preconcentration. The physical properties of the column considered best for preconcentration were determined using an Auto Pore IV 9500 mercury intrusion porosimeter. The total pore area was 4.795 $m^2/g$, the average pore diameter was 3.2947 $\mu$m, and the porosity was 82 percent. Analyte concentration prior to CE separation can be accomplished by the use of a monolith placed at the inlet of the CE separation capillary.

### ACKNOWLEDGMENT

This work was funded by the National Institutes of Health (R01GM06547-01A1).

*Keywords:* capillary electrophoresis, protein, separation sciences
*Application code:* proteomics and genomics
*Methodology code:* capillary electrophoresis

### HIGH-THROUGHPUT, GENOME-SCALE PROTEIN PRODUCTION FREE EXPRESSION SYSTEM

***Tatsuya Sawasaki,* Yaeta Endo, Masatoshi Fujiwara, Ryo Morishita, and Motoo Watanabe***

**Cell-Free Science and Technology Research Center, Ehime University, Matsuyama 790-8577, Japan*

Current cell-free protein expression systems are capable of synthesizing proteins with high speed and accuracy; however, the yields are low due to their instability over time. *Escherichia-coli*-based systems are not always sufficient for expression of eukaryotic proteins. This report reviews a high-throughput protein production method based on the cell-free system prepared from eukaryote, wheat embryos. We first demonstrate a method for preparation of this extract that exhibited a high degree of stability and activity. To maximize translation yield and throughput, we address and resolve the following issues: (1) optimization of the ORF flanking regions; (2) PCR-based generation of DNA for mRNA production; (3) expression vectors for large-scale protein production; and (4) a translation reaction that does not require a membrane. The combination of these elemental processes with robotic automation resulted in high-throughput protein synthesis.

*Keywords:* automation, method development, protein
*Application code:* proteomics and genomics
*Methodology code:* others

### AN ACCURATE AND SENSITIVE METHOD FOR THE DETERMINATION OF METHYLMERCURY IN BIOLOGICAL SPECIMENS USING GC-ICPMS WITH SOLID-PHASE MICROEXTRACTION

***Clay Davis,*[*] *Colleen E. Bryan, Steven J. Christopher, and Russell D. Day***

[*]*Hollings Marine Laboratory, National Institute of Standards and Technology, Charleston, SC 29412, USA*

Various levels of information are required for proper assessment of trace element species including total elemental composition, oxidation states, and bound ligand/molecule identification. While chromatographic methods exist for separating the various species of a trace metal present in an environmental or biological matrix, many times the analyte of interest is present in such a low concentration that instrumental sensitivity becomes the limiting factor in the analysis. Compared with other detection methods, inductively coupled plasma mass spectrometry (ICPMS) has the unique advantages of element-specific detection, wide dynamic range, low limits of detection, and the ability to perform isotope dilution analysis. The aforementioned advantages make ICPMS a powerful instrumental technique for the determination of trace element species in chromatographic effluents. Development of an acid-assisted, microwave extraction method and its application to the quantification of methylmercury (MeHg) in biological samples is described. Capillary gas chromatography with inductively coupled plasma mass spectrometric (GC-ICPMS) detection was utilized for the identification and quantification of MeHg in some current-issue NIST Standard Reference Materials (SRMs). The method was validated for the determination of MeHg concentrations at trace levels (less than 20 ng/g) in blood samples using SRM 966 toxic metals in bovine blood. Measured concentration values for the GC-ICPMS method for MeHg are compared with data derived from complementary analytical methods. Additionally the method was used to determine the MeHg concentration in a limited number of blood samples collected from bottlenose dolphins (*Tursiops truncatus*) and loggerhead sea turtles (*Caretta caretta*) during various health and population monitoring assessments.

*Keywords:* atomic spectroscopy, environmental/biological samples, ICP-MS, speciation
*Application code:* environmental
*Methodology code:* atomic spectroscopy/elemental analysis

### SPECIATION OF METHYLMERCURY AND INORGANIC MERCURY IN ENVIRONMENTAL AND BIOLOGICAL SAMPLES BY FLOW INJECTION COLD VAPOR GENERATION FROM TETRACHLOROTIN(II) AND TETRAHYDROBORATE-FORM ANION-EXCHANGE COLUMNS WITH ATOMIC ABSORPTION SPECTROMETRY

***Wipharat Chuachuad*[*] *and Julian F. Tyson***

[*]*University of Massachusetts Amherst, 710 N. Pleasant Street, Amherst, MA 01003-9306, USA*

In an on-going study of the possibilities of immobilized reagents for chemical vapor generation, procedures for the determination of mercury species have been evaluated. The advantages of this approach are that purer reagents are generated, less reagents are consumed, and less waste is generated in comparison with procedures in which homogeneous solution reactions are used. The effects of column dimensions, types of resins, concentration of generating reagents, loading time, loading flow rate, carrier reagent flow rate, carrier gas flow rate and acidity of sample by the flow-based system were investigated. Inorganic mercury was determined by passing the acidified sample (in 0.10 M HCl) through the immobilized tin(II) chloro anion column loaded with 0.5% (w/v) tin(II) chloride in 0.5% (v/v) HCl. For both inorganic mercury and methylmercury, the acidified sample was passed through an immobilized tetrahydroborate column loaded with 0.3%(w/v) $NaBH_4$ in 0.1% (v/v) TMAH. Methylmercury was determined after subtracting the contribution from inorganic mercury. The limits of detection (3 seconds) of inorganic mercury and methylmercury were 5 ng/L and 92 ng/L, respectively. From the analyses of natural water and seawater, good accuracies based on percentage recovery of methylmercury and inorganic mercury were obtained. Better tolerance for some coexisting elements compared to that of the conventional cold vapor generation technique was found. The method was validated by the determination of inorganic mercury and methylmercury in biological standard reference material samples DORM-2 (dogfish muscle), DOLT-2 (dogfish liver), and SRM 2670 (urine), and was applied to the analysis of whale liver and goat blood.

*Keywords:* atomic absorption, environmental/biological samples, mercury, speciation
*Application code:* environmental
*Methodology code:* atomic spectroscopy/elemental analysis

### A METHOD FOR DETERMINING MICROBIOLOGICALLY MEDIATED RELEASE OF ELEMENTAL AND ORGANOMERCURY COMPOUNDS FROM CCBS USING SPME, GAS CHROMATOGRAPHY, AND ATOMIC FLUORESCENCE

***David J. Hassett,*[*] *Loreal V. Heebink, and Erick J. Zacher***

[*]*UND Energy & Environmental Research Center, 15 North 23rd Street, PO Box 9018, Grand Forks, ND 58202-9018, USA*

Mercury release from coal combustion by-products (CCBs) is a current topic of interest. Microbiologically mediated mercury release can result from methylation reactions as well as from changes in redox potential and pH in CCB applications such as agricultural use and soil stabilization. The Energy & Environmental Research Center has developed a method of determining organomercury and elemental mercury vapor releases and liquid fraction organomercury species in experiments conducted in which bacterial cultures were grown in the presence of CCBs known to have relatively high mercury concentrations. Mercury vapors were captured on gold-coated quartz and *Supelco Carbotrap* sorption traps and analyzed with atomic fluorescence spectroscopy (AF). The organomercury species in the liquid fraction were determined by a derivitization method using boroethylation, boropropylation, or borophenylation followed by capture with a solid-phase microextraction (SPME) fiber coated with polydimethylsiloxane, gas chromatographic separation, and AF detection.

The addition of microbes to the CCBs did generate organomercury species. Dimethylmercury, diethylmercury, and methylmercuric chloride were identified in the liquid fraction of a CCB slurry after the addition of bacteria. These experiments are being repeated to confirm or refute the reproducible formation of these compounds. It is likely that the diethyl mercury, which was somewhat unexpected, was formed from ethanol produced by anaerobic fermentation of the glucose used to feed the bacterial culture.

### ACKNOWLEDGMENTS

This abstract was prepared with the support of the US Environmental Protection Agency (EPA) through the Center for Air Toxic Metals (CATM) and the US Department of Energy (DOE) in part through the Coal Ash Resources Research Consortium(CARRCSM).

*Keywords:* gas chromatography, mercury, speciation, SPME
*Application code:* environmental
*Methodology code:* atomic spectroscopy/elemental analysis

### RAPID ANALYSIS OF MERCURY IN FISH TISSUE USING DIRECT MERCURY ANALYSIS

***Michael A. Levine,* Peter M. Grohse, Keith E. Levine, and Frank X. Weber***

**RTI International, PO Box 12194, Research Triangle Park, NC 27709-2194, USA*

A study was performed to (1) estimate the mercury content in tuna from a variety of sources and (2) assess the efficiency and accuracy of a direct mercury analyzer during such a screening process. This measurement approach involved the thermal decomposition, gold amalgamation, and detection of mercury from tissue samples by atomic absorption spectroscopy. Prepacked and freshly caught seafood samples were lyophilized, ground to a powder, and homogenized. The powdered tissue was then directly analyzed for mercury content.

The analyzer was calibrated using animal tissue CRMs and demonstrated a detection limit of less than 0.1 ng Hg and a quantitative range of between 0.5 ng and 500 ng . The analysis of a wide range of reference materials, including animal and plant tissue CRMs and SRMs as well as coal and sediment SRMs, demonstrated recoveries consistently between 85% and 115% at concentrations between 0.080 $\mu$g Hg/g and 4.6 $\mu$g Hg/g. No dissolution step was required and approximately 80 samples can be measured unattended in an 8-hour period. The average amount of Hg found in canned tuna packed in water was 20.5 $\mu$g/serving (suggested serving size 3 ounces); canned tuna packed in oil was 16.3 $\mu$g/serving; and tuna packed in a pouch with water was 26.9 $\mu$g/serving. The method was found to be highly sensitive, accurate, matrix independent, and efficient. Manufacturers and regulatory agencies concerned with monitoring the lot-to-lot tuna quality may find this method an attractive alternative to the more classical acid dissolution/cold vapor atomic absorption (CVAA) methodology.

*Keywords:* environmental/biological samples, mercury, quality control, trace analysis
*Application code:* environmental
*Methodology code:* atomic spectroscopy/elemental analysis

### DETERMINATION OF MERCURY IN AN ASSORTMENT OF DIETARY SUPPLEMENTS USING AN INEXPENSIVE COMBUSTION ATOMIC ABSORPTION SPECTROMETRY TECHNIQUE

***Keith E. Levine,* Peter M. Grohse, Michael A. Levine, Jason M. Perlmutter, and Frank X. Weber***

**RTI International, PO Box 12194, Research Triangle Park, NC 27709-2194, USA*

The mercury concentrations of 40 commercially available dietary supplements were determined using a new, inexpensive analysis technique. The method involved thermal decomposition, amalgamation, and detection of mercury by atomic absorption spectrometry with an analysis time of approximately 6 minutes per sample. The primary cost savings from this approach was that sample digestion was not required prior to analysis. As a result, manufacturers and regulatory agencies concerned with monitoring lot-to-lot product quality may find this approach an attractive alternative to the more classical acid-decomposition, cold vapor atomic absorption methodology. Dietary supplement samples included astragalus, calcium, chromium picolinate, echinacea, ephedra, fish oil, ginger, *Ginkgo biloba*, ginseng, goldenseal, guggul, senna, St John's wort, and yohimbe products. Quality control samples analyzed with the dietary supplements indicated a high level of method accuracy and precision. Ten replicate preparations of a standard reference material (NIST 1573a, tomato leaves) were analyzed, and the average mercury recovery was 109% (2.0% RSD). The method quantitation limit was 0.3 ng, which corresponded to 1.5 ng/g sample. The highest mercury concentration (123 ng/g) found was measured in a concentrated salmon oil sample. When taken as directed, this product would result in an approximate mercury ingestion of 7 $\mu$g per week.

*Keywords:* environmental/biological samples, mercury, quality control, trace analysis
*Application code:* environmental
*Methodology code:* atomic spectroscopy/elemental analysis

### FULLY AUTOMATED MERCURY ANALYSIS: JUST PRESS 'START'

***Jason P. Gray,* Alvin Chua, and Koji Tanida***

**AGS Scientific, Inc, 1511 Texas Ave S, Suite 270, College Station, TX 77840, USA*

Mercury (Hg) is one of the most highly monitored environmental pollutants in the world today. Inexhaustible world population growth and decreasing water supplies are factors that continue to drive the need for reduction of industrial pollution. With such high demand for monitoring mercury contamination levels, automation of mercury analysis methods is of increasing importance for industrial compliance laboratories, government regulatory laboratories, and environmental contract laboratories. One of the more troubling steps required in many of the traditional analytical methods is the sample preparation step, which requires acidic digestion of the samples prior to analysis. Such sample preparation is labor intensive, consumes valuable time, and can be hazardous due to the handling of concentrated acids at high temperatures. Many automated mercury analyzers are available today; however few, if any, have been able to fully automate methods such as EPA 245.1, 245.2, 245.7, and 1631E from digestion through analysis. In this presentation, a new mercury analysis system that is capable of total automation of such methods, including the digestion and reagent addition, will be described. This mercury system functions on the principles of reducing vaporization, optional gold amalgamation, and cold vapor atomic absorption spectroscopy (CVAAS). Illustrations and supporting data will be provided.

*Keywords:* atomic spectroscopy, environmental analysis, mercury, trace analysis
*Application code:* environmental
*Methodology code:* atomic spectroscopy/elemental analysis

### ADDRESSING THE CHALLENGES OF TOTAL ARSENIC DETERMINATION IN VARIOUS ENVIRONMENTAL SAMPLES USING HYDRIDE GENERATION- ATOMIC FLUORESCENCE SPECTROMETER (HG-AFS)

***Olujide T. Akinbo,* Megan Bergauff, and Jon Clark***

**Butler University, 4600 Sunset Avenue, Indianapolis, IN 46208, USA*

HG-AFS has been widely used for the determination of total arsenic in different types of water samples. In addition to being cheaper, HG-AFS sensitivity and detection limits are comparable to those of ICPMS. However the adverse effect of sample matrix on the hydride generation reaction and the stability of the hydrogen diffusion flame of AFS limits the realization of its full potential with other environmental samples, particularly those that require acid digestion. For example, introduction of undiluted nitric acid digestate of hair sample into the AFS at conventional flow rates causes a vigorous reaction that results in poor signal reproducibility, flame instability, and, in some cases, extinguishment. Also, introduction of acid digestates of edible oil samples resulted in very broad and suppressed signals. In addition to these problems, the hydride generation reaction requires high-concentration reagents (eg, 30% HCl) at relatively high flow rates (6–10 Ml/min) thus resulting in high rates of reagent and sample consumption, and waste generation. In this presentation we will discuss how these challenges were addressed in our lab during arsenic determination in hair, milk, vegetable, and edible oil samples.

*Keywords:* atomic spectroscopy, biological samples, elemental analysis, environmental analysis
*Application code:* environmental
*Methodology code:* atomic spectroscopy/elemental analysis

### QUANTIFICATION OF ORGANIC AND INORGANIC ARSENIC AND SELENIUM SPECIES IN STANDARD REFERENCE MATERIALS AND CLINICAL MARINE SAMPLES BY HIGH-PERFORMANCE LIQUID CHROMATOGRAPHY COLLISION CELL INDUCTIVELY COUPLED PLASMA MASS SPECTROMETRY

***Clay Davis,* Steven J. Christopher, and Rebecca S. Pugh***

**Hollings Marine Laboratory, National Institute of Standards and Technology, Charleston, SC 29412, USA*

It is well known that the chemical form, and not the total concentration, determines the toxicity or bioavailability of metallic compounds. Of particular interest is not only the quantitative determination of the amount of a particular element present in a system, but the identification of the associated elemental complex in order to successfully assign toxic species. An ideal solution to the problem of species-specific detection is the combination of powerful separation techniques such as liquid chromatography (LC) with compatible detection modes such as inductively coupled plasma mass spectrometry (ICPMS).

A lack of reference materials certified for elemental species has been a major limiting factor in speciation analysis. Method development and validation requires reference materials, in a variety of matrixes, in order to produce precise and accurate results.

To this end, a chromatographic method was developed to simultaneously separate both organic and inorganic arsenic and selenium compounds while monitoring the most abundant isotopes of arsenic at m/z 75 and selenium at m/z 80. Total arsenic concentrations as well as individual arsenic species concentrations were determined in reference materials DORM-2, standard reference materials (SRM) 1566b Oyster Tissue, SRM 2976 Mussel Tissue (trace elements and methylmercury), as well as archived liver samples of ringed seals (*Phoca hispida*) from the National Biomonitoring Specimen Bank (NBSB). Major goals of this work were the identification of NIST SRMs that could be certified for arsenic species, preferred SRM form (freeze dried or fresh frozen), determination of species stability during long-term storage of tissue samples in the NIST NBSB, and monitoring long-term trends in environmental quality.

*Keywords:* atomic spectroscopy, environmental/biological samples, ICP-MS, speciation
*Application code:* environmental
*Methodology code:* atomic spectroscopy/elemental analysis

### THE ABC'S OF ARSENIC, BANGLADESH AND CANCER

***Jerry DeMenna* and Eid Al-Khatib***

**Buck Scientific, 58 Fort Point Street, East Norwalk, CT 06855, USA*

The nation-wide problem of arsenic contamination in Bangladesh is actually a world-wide problem. Most health agencies recognize the damage that chronic exposure to arsenic can cause in all people, but particularly women. Scientific studies in the US, and around the world, have shown a statistically significant increase in the appearance and severity of breast cancers in women. Such validated evidence has been cause for most states in America to lower their drinking water levels for arsenic from the federal 50 ppm to 5.0 ppm. The high arsenic levels in Bangladesh, an effect of naturally occurring deposits and overdevelopment of formerly untouched regions, has created a national epidemic for the Bangladeshi.

Efforts are being made to develop both a "quick and easy" test that can be performed in every town to ascertain the levels of contamination, and to design an efficient and readily available method to clean up such contamination. Classical methods range from hydride generation atomic absorption spectroscopy (based on the ancient "Marsh" test for arsenic) to UV-Vis molecular spectrophotometry (arising from indirect "displacement" reactions). Add to this ion chromatography, plasma emission spectrometry and even colorimetry, and . . . you have a complex "competition" of general analytical disciplines and specific techniques.

What need to be considered for implementing any protocols are the detection limits of the method, interferences and their magnitude (if any), working range, stability of chemical reactions, sample preparation and operation labor, the durability of the instrumentation, to . . . start!

This presentation will provide short but detailed overviews of all the analytical methodologies available for Arsenic, with operating specifications, procedures and actual data from ground-water samples. Covered topics include hydride-AAS, GFAAS, UV-Vis, HPLC, I-C, DCP, and polarography, with detection limits and interferences outlined for this analysis.

*Keywords:* atomic absorption, atomic spectroscopy, environmental/water, UV-VIS absorbance/luminescence
*Application code:* environmental
*Methodology code:* atomic spectroscopy/elemental analysis

### TRACE METAL ANALYSIS ON BORON-DOPED DIAMOND ELECTRODES FOR WATER QUALITY MONITORING

***Elizabeth A. McGaw,* Prerna Sonthalia, and Greg M. Swain***

**Department of Chemistry, Michigan State University, East Lansing, MI 48824, USA*

On-board the International Space Station (ISS) potable water is used for drinking, food preparation, and hygiene. There are several potential sources of contamination, both organic as well as inorganic, that are potentially toxic to the crew. Several heavy metals have been detected in the ISS water (eg, Ag(I), Pb(II), and Cd(II)). The latter two were found at concentrations much higher than the EPA action levels. Onboard monitoring of these contaminants is essential to crew health. However, there is currently no on-board technology used for water quality monitoring (organic and/or inorganic contaminants).

Electrochemical methods lend themselves to use in space as they are versatile, energy efficient, easy to be automated, environmentally compatible, and cost effective. Traditionally, anodic stripping voltammetry (ASV) with an Hg electrode has been employed for trace metal ion analysis. Alternate electrodes, however, are being investigated because of the stability, toxicity, and volatility of Hg. Our group has shown that boron-doped diamond electrodes are a viable alternate electrode and can be used successfully for terrestrial ASV analysis of heavy metals found in water, sludge, and soil samples. Results regarding method optimization for water quality monitoring on board ISS will be studied. The effects of solution pH, dissolved oxygen, and salt content will be presented. Additional studies regarding the electrode-to-electrode response precision and the long-term response stability will also be presented.

*Keywords:* electrochemistry, metals, stripping analysis
*Application code:* environmental
*Methodology code:* electrochemistry

### SIMULTANEOUS DETECTION OF ETHANOL AND SMOKING BY-PRODUCTS WITH FUEL-CELL-BASED SENSORS

***Johna Leddy* and Luke Haverhals***

**Department of Chemistry, University of Iowa, Iowa City, IA 52242, USA*

Fuel cells are predominantly used for power generation, but they are also used as sensors that measure ethanol in aspirated breath. These sensors serve in ignition locks mandatorily installed in the vehicles of drivers convicted of driving under the influence (DUI). The sensors must be both sensitive and selective; it is the selectivity that is remarkable in a matrix as complex as human breath. Sensors respond to very few components other than ethanol; the most common interferent is a by-product of cigarette smoking. Data collected are voltage drops generated across the cell and recorded as function of time. Data show an initial rise followed by a decay. Wave shapes appropriate to ethanol and smoke by-products are similar but not the same. Samples are examined in the biologically relevant region between 0.01 and 0.30 blood alcohol concentrations (BAC - g ethanol / 100 mL of blood or 250 L of breath) where the legal limit is 0.08 BAC in most states. Cigarette smoke is detected up to 0.5 to 1 hour after smoking. An algorithm is presented to quantify ethanol and smoke by-products. When samples are generated for simultaneous ethanol and smoke by-products, the algorithm allows the ethanol and smoke by-product levels to be determined independently. Ethanol can be quantified when the signal for the smoke by-products is 50x that of the ethanol. Ethanol levels can be determined to 0.01 BAC.

Coupling of fuel-cell-based sensors and the appropriate algorithm may allow breath-based sensors to be developed for other analytes.

*Keywords:* electrochemistry, electrodes, sensors, statistical data analysis
*Application code:* bioanalytical
*Methodology code:* electrochemistry

### MONITORING BETA-GLUCOSIDASE ENZYMATIC HYDROLYSIS OF CARBOHYDRATES BY QUANTITATIVE IN VITRO MICRODIALYSIS

***Swati J. Modi*[*] and William R. LaCourse***

[*]*University of Maryland, Baltimore County, 1000 Hilltop Circle, Baltimore, MD 21250, USA*

Enzymes play an important role in numerous industries (eg, clinical diagnostics, food processing, and industrial bioprocessing). Crucial to understanding and optimizing enzyme-based systems is the measurement of fundamental enzyme parameters. A sensitive and reliable method is described for determining Michaelis-Menten enzyme-kinetic parameters with almond $\beta$-glucosidase as a model enzyme. Using in vitro microdialysis for on-line sampling, HPLC followed by UV absorbance detection and pulsed electrochemical detection was used to monitor carbohydrate enzyme reactions. This method has been applied to the determination of salicin and its conversion to saligenin. Limits of detection of salicin and saligenin were found to be 100 ppb (8 pmol, 20 $\mu$L) and 60 ppb (10 pmol, 50 $\mu$L), respectively. Accurate quantitation was achieved via internal standard methodology. Km and Vmax were obtained for salicin by microdialysis sampling using polyacrylonitrile membranes and were compared to spectrophotometric data. In addition to high sensitivity, microdialysis-HPLC allows for the simultaneous detection of substrate and product and the ability to monitor the kinetics of these conversions, as is shown for several other substrates. The online microdialysis setup reduces sample preparation and produces simpler chromatograms in a variety of matrices. Finally, this method was modified for monitoring the enzymatic hydrolysis of the nonchromophoric carbohydrates (eg, cellobiose) using high-performance anion-exchange chromatography and pulsed electrochemical detection, showing that this versatile method has far-reaching applications to monitoring a variety of carbohydrates in enzymatic processes.

*Keywords:* bioanalytical, carbohydrates, enzyme assays, membrane
*Application code:* bioanalytical
*Methodology code:* electrochemistry

### USING AUTOMATED DECONVOLUTION SOFTWARE TO FIND NEEDLES IN A HAYSTACK

***Chinkai Meng,*[*] *Mike Szelewski, and Philip L. Wylie***

[*]*Agilent Technologies, Inc, 2850 Centerville Road, Wilmington, DE 19808, USA*

The term deconvolution is used here in the broad sense of extracting one or more compound spectra from a complex total ion chromatogram (TIC). This is the goal of AMDIS (automatic mass spectral deconvolution and identification software) developed by NIST (National Institute of Standards and Technology). AMDIS looks at every ion's rising and falling pattern through out the TIC to group related ions together into a deconvoluted spectrum. The matrix background or interference ions would then be left out of the deconvoluted spectrum. Each deconvoluted (cleaned) spectrum is then searched against a target library for hits. The timesaving using deconvolution is about 30 minutes per sample, compared to manual manipulation (peak averaging and background subtraction of each potential target compound) of the data by an experienced analyst.

Another important feature of the deconvolution is the consistency of the report. Since AMDIS will do a good job in pulling out "deconvoluted/cleaned" spectra from complex matrix background, human factors (mood, concentration, and skill level) will not affect the validity of the results. Examples in pesticide residue, allergens, and forensics will be presented.

*Keywords:* environmental analysis, gas chromatography/mass spectrometry, GC-MS, pesticides
*Application code:* general interest
*Methodology code:* gas chromatography/mass spectrometry

### AN HPLC AUTOSAMPLER OF ULTIMATELY REDUCED CARRYOVER EQUIPPED WITH AN ULTRASONIC CLEANING DEVICE

***Osamu Shirota,* Tsuneaki Kaneko, Masashi Mita, Yuki Togashi, and Xiaomi Xin***

**Shiseido Company, 2-2-1 Hayabuchi Tsuzuki, Yokohama 224-8558, Japan*

An attempt to install an ultrasonic cleaning device to an autosampler for HPLC is presented in this paper. Carryover, or a memory effect, from previous sample runs is becoming a serious problem in LC or LC-MS, especially in the field of pharmacokinetics and clinical analysis, where concentrations of unknown samples often range widely. While various practical strategies to clean an autosampler have been attempted in the real field, a problem common to them is the time required to decrease a carryover peak. Ultrasonic cleaning is one of the most common cleaning techniques used extensively in laboratory experiments for industrial production. It will be discussed how quickly and efficiently the autosampler will be cleaned by the ultrasonic cleaning device through carryover-evaluating experiments with many compounds supposed to be "difficult."

*Keywords:* HPLC detection, liquid chromatography/mass spectroscopy
*Application code:* bioanalytical
*Methodology code:* liquid chromatography

### HIGH-THROUGHPUT DETERMINATION OF THE PHARMACEUTICAL ACTIVE DIAZEPAM BY ION MOBILITY SPECTROMETRY (IMS)

***John J. Carroll* and Reno DeBono***

**Smiths Detection, 30 Technology Drive, Warren, NJ 07059, USA*

The purpose of this paper is to develop a fast, precise method for the determination of the pharmaceutical active diazepam using ion mobility spectrometry (IMS).

Ion mobility spectrometry (IMS) is a fast, sensitive, and simple-to-use alternative to HPLC for pharmaceutical analyses, especially cleaning validation. High-performance injection (HPI) has recently been developed as a means of sample introduction for IMS analyzers. HPI incorporates a variable-temperature, variable-flow injector in place of thermal desorption from a solid substrate. This innovation improves the quantitative performance and extends the range of pharmaceutical applications of IMS. The data were obtained using an IONSCAN-LS with HPI system from Smiths Detection (NJ, USA)). A robotic autosampler made 1 $\mu$L injections of diazepam standards prepared in isopropyl alcohol into the HPI. Instrument response was based on the diazepam molecular ion peak at $K0 = 1.2124$ detected in positive ion mode.

Each sample took 8 seconds to be analyzed. The cycle time of 48 seconds per sample was limited by the autosampler. The detection limit for diazepam was estimated to be 2 pg, or 0.002 $\mu$g/mL for a 1 $\mu$L injection. The instrument response versus concentration was plotted from 0.005 to 0.250 $\mu$g/mL. A linear range was identified between 0.005 and 0.050 $\mu$g/mL with $R2 = 0.9957$. Four sets of five replicate measurements of a 0.05 $\mu$g/mL standard were made over a two-hour period to assess the short- and long-term precision. The mean of the RSDs within each set, which indicate the short-term repeatability, was 2.1%. The RSD between the means of the four sets, which indicates the degree of long-term drift, was 1.5%.

A method for the quantitation of diazepam with high sample throughput and excellent sensitivity, linearity, and short- and long-term precision was successfully developed using IMS with HPI.

*Keywords:* high-throughput chemical analysis, method development, pharmaceutical, validation
*Application code:* pharmaceutical
*Methodology code:* mass spectrometry

